# A Boratafulvene

**DOI:** 10.1002/anie.202107968

**Published:** 2021-08-06

**Authors:** Tobias Heitkemper, Leonard Naß, Christian P. Sindlinger

**Affiliations:** ^1^ Institut für Anorganische Chemie Georg-August-Universität Göttingen Tammannstrasse 4 37077 Göttingen Germany

**Keywords:** borane, borata fulvene, boroles, C-H acidity, main group chemistry

## Abstract

Structurally authenticated free B‐alkyl boroles are presented and electronic implications of alkyl substitution were assessed. Deprotonation of a boron‐bound exocyclic methyl group in a B‐methyl borole yields the first 5‐boratafulvene anion—an isomer to boratabenzene. Boratafulvene was structurally characterized and its electronic structure probed by DFT calculations. The p*K*_a_ value of the exocyclic B−CH_3_ in a set of boroles was computationally approximated and confirmed a pronounced acidic character caused by the boron atom embedded in an anti‐aromatic moiety. The non‐aromatic boratafulvene reacts as a C‐centered nucleophile with the mild electrophile Me_3_SnCl to give a stannylmethyl borole, regenerating the anti‐aromaticity. As nucleophilic synthons for boroles, boratafulvenes thus open an entirely new avenue for synthetic strategies toward this highly reactive class of heterocycles. Boratafulvene reacts as a methylene transfer reagent in a bora‐Wittig‐type reaction generating a borole oxide.

## Graphical abstract

The first boratafulvene was accessed by deprotonation of a B‐methyl borole. The boratafulvene is a new C‐nucleophilic synthon for substituted boroles and reacts with carbonyls in a bora‐Wittig‐type reaction transferring the methylene group.

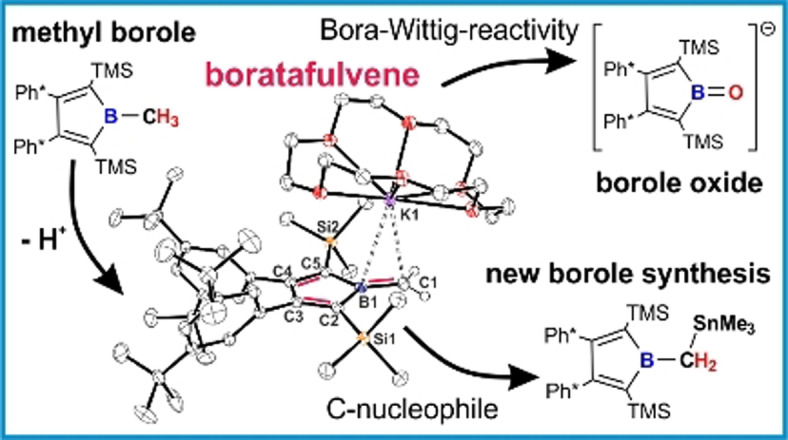

## Introduction

**Scheme 1:**
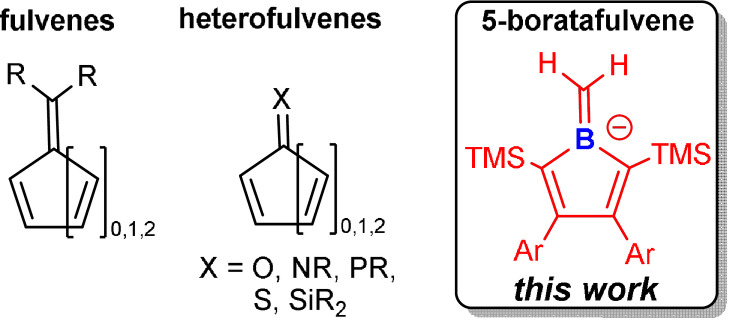
Examples for fulvenes and heterofulvenes.

Replacement of carbon by isoelectronic heteroelement fragments in classic hydrocarbons has long been a fruitful synthetic challenge which led to fundamental structural motifs of E/C−E bonding interactions.^[1]^ A plethora of molecules and materials with altered properties resulted from these efforts, particularly when more electropositive boron atoms are introduced.^[2]^ Among classic hydrocarbon molecules, the parent (penta)fulvene is a reactive isomer of benzene featuring an unsaturated five‐membered ring with a “cross‐conjugate” exocyclic methylene group.^[3]^ Compounds conventionally also considered heterofulvenes usually feature exocyclic electronegative oxygen or imine nitrogen atoms (Scheme 1).^[4]^ Heterofulvenes with endocyclic heteroatoms, except for ubiquitous N‐atom containing rings as in dipyrromethene‐based compounds, are much scarcer and often transient.^[5]^ Erker and Nöth reported on borata‐(di)benzofulvene derivatives with exocyclic =BR_2_ moieties.^[6]^

We now present an anionic 5‐boratafulvene, accessed by deprotonation of B‐methyl 1*H*‐boroles, with an exocyclic methylene group as a new entry into heterofulvene chemistry (Scheme 1). 1*H*‐Boroles are unsaturated five‐membered boron heterocycles with four cyclic conjugate π‐electrons and reveal (weakly) anti‐aromatic properties.^[7]^ This results in high reactivity of the butadiene and pronounced Lewis acidity of the organoborane moiety.

## Results and Discussion

**Figure 1:**
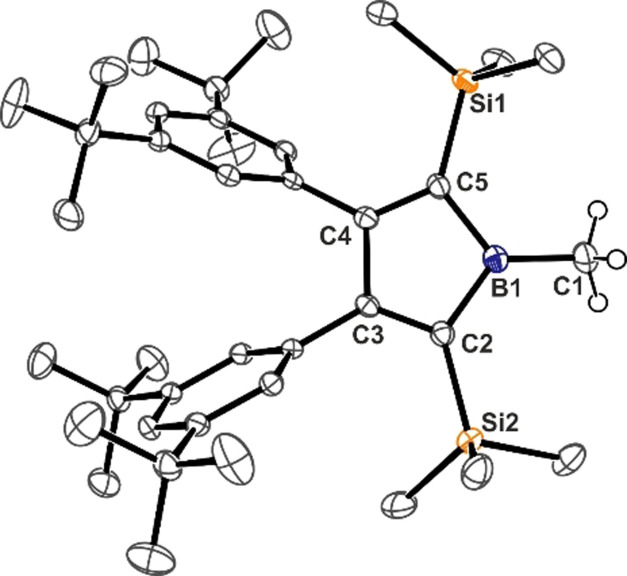
ORTEP plot of the molecular structure of **1**. Atomic displacement parameters are drawn at 50 % probability level. Hydrogen atoms except for C1‐bound H are omitted for the sake of clarity. Selected bond lengths [Å]: B1–C1 1.559(2), B1–C2 1.595(2), C2–C3 1.357(2), C3–C4 1.539(2), C4–C5 1.354(2), C5–B1 1.587(2), C2–Si2 1.871(2), C5–Si1 1.866(2).

Only few substitution patterns that sufficiently stabilize free boroles have been reported and our group has recently established reliable protocols towards 1‐chloro‐2,5‐(TMS)_2_‐borole (**A**).[^7b^, ^8^] When **A** was treated with ethereal methyl Grignard solutions, 1‐methylborole **1** is formed and is obtained in ca. 80 % crystalline yield as a brightly orange solid. Boron‐bound alkyl groups in free boroles are rare: (PhC)_4_BCH_3_, prepared by Sn/B exchange from (PhC)_4_SnMe_2_ and MeBX_2_, is the only derivative described in the literature.[^7b^, ^7c^] Related B‐alkyl (di)benzoboroles,^[9]^ and Me‐borole derivatives, sufficiently stabilized in transition metal complexes or as base adducts, are documented.^[10]^ We were able to structurally characterize **1** and its molecular structure is depicted in Figure 1.

Localized single and double bonds are found within the central borole ring in **1** as to be expected for an anti‐aromatic system. The exocyclic methyl group is slightly bent out of the borole plane by ca. 7° (H_3_C‐B‐(C_β_C_β_)_centroid_ ca. 173°) with a B−CH_3_ bond length of 1.559(2) Å in the typical range of B−C(sp^3^) single bonds. The characteristic π→π* transition of borole‐based frontier orbitals is found at *λ*_max_=458 nm, slightly blue shifted to comparable B‐aryl derivatives (*λ*_max_≈475 nm).[^7b^, ^8d^] As to be expected for tri‐coordinate boron, the ^11^B NMR resonance is found lowfield‐shifted at 80.0 ppm and the B‐methyl group resonates at 1.32 ppm (^1^H) and 11.9 ppm (^13^C).

α‐CH acidity of boranes is known.[^6a^, ^11^] However, suitable diorgano alkyl boranes R_2_B(CHR′_2_) and conditions that allow selective deprotonation are scarce.^[6b]^ Successful deprotonation of Ar_2_BCH_3_ with suitable amides to yield borataalkenes is restricted to sterically demanding aryl groups (such as mesityl) and bases that prevent from adduct formation and quaternation at the boron atom.[^11b^, ^12^] This route granted access to the yet sole example of a structurally characterized borataalkene with an unsupported terminal methylene unit in [Mes_2_BCH_2_]^−^.^[11b]^ Erker proposed intermediate formation of borataalkenes by tautomerization in an indane‐bridged FLP.^[11a]^

**Figure 2:**
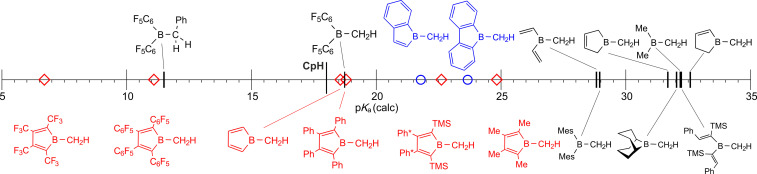
Computationally approximated p*K*_a_ values of various B‐methyl boranes (black dashes), B‐methyl boroles (red squares) and B‐methyl(di)benzoborole (blue circles). RI‐BP86‐D3BJ/def2TZVPP with a solvent model for DMSO and exp. p*K*_a_=18.0 as reference.^[11a]^

As a further example, Herberich reported twofold deprotonation of endocyclic α‐CH in 1‐amino‐3‐borolene to yield the Hückel‐aromatic borole dianion.^[13]^ We reasoned that the exocyclic methyl protons of **1** bound to a Lewis‐acidic boron atom, which is embedded in an anti‐aromatically destabilized borole moiety, might reveal an increased acidic character that would facilitate deprotonation. Along the lines of a recent computational approach by Erker and coworkers to estimate p*K*_a_ values for α‐CH bonds in boranes (with p*K*_a_(CpH)=18.0 as a reference),^[14]^ we found the p*K*_a_ of **1** with polar DMSO solvent model to be 22.6, slightly higher than, for example, (C_6_F_5_)_2_BCH_3_ (18.7, Figure 2).[^11a^, ^15^] Notably, a series of substituted B‐methyl boroles were probed and all revealed general, significantly increased acidity along with strong dependency on the substituents [(MeC)_4_BMe 24.8; (PhC)_4_BMe 18.8; (HC)_4_BMe 18.6; (Ph^F^C)_4_BMe 11.1 (Ph^F^=C_6_F_5_); (F_3_CC)_4_BMe 6.7]. Methyl boranes with comparably inductively active vinyl substituents, yet lacking the cyclic conjugation, reveal significantly higher computational p*K*_a_ (vinyl: 28.8; 1‐silyl‐2‐phenylvinyl: 32.2) than the respective boroles, clearly pointing at the remarkable acidity enhancement which results from the thermodynamic incentive that is the removal of anti‐aromaticity upon deprotonation. Comparison with predicted p*K*_a_ of five‐membered B‐methyl 2‐ or 3‐borolene (32.6; 31.7) also advocates against ring‐strain effects to account for the increased C−H acidity in methylborole compared to acyclic divinyl derivatives. Compared to parent methyl borole (HC)_4_BMe, benzannulation as in 1‐boraindene (21.8) or 9‐borafluorene (23.7) increasingly reduces the C−H acidity, presumably due to reduced anti‐aromatic character. We propose this (computational) acidity assessment to be a useful measure for anti‐aromaticity‐driven reactivity enhancement in boroles. Previous approaches to quantify this effect include shifts in CN‐stretching modes of nitril adducts to boroles,^[7b]^ and computational measures such as aromatic stabilization energy (ASE) or NICS.^[16]^

**Scheme 2:**
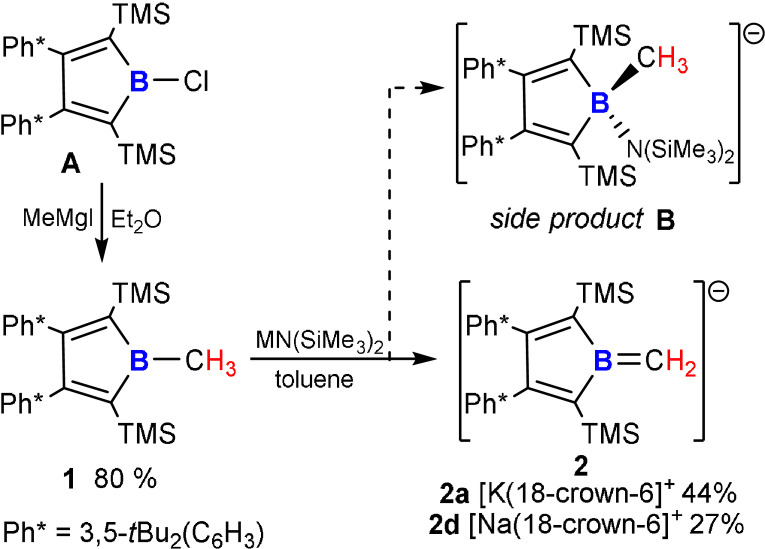
Synthesis and deprotonation of methyl borole **1**.

Successful deprotonation of **1** (Scheme 2) and reliable isolation of boratafulvene anion **2** are very sensitive to base and solvation conditions. In benzene, treatment with LiTMP (TMP=2,2′,6,6′‐tetramethylpiperidine) leads to decomposition and intractable mixtures, while in [D_8_]THF immediate clean conversion with LiTMP is indicated by NMR monitoring. However, isolation attempts fail as, again, intractable mixtures form. Treatment of orange solutions of **1** in toluene with K[N(SiMe_3_)_2_] for 18 h yields a sparingly soluble yellow solid. This crude solid contained boratafulvene **2** and varying amounts (0 to ca. 30 %) of two side‐products, of which one was identified as the colorless amide adduct **B** (Scheme 2, see SI for structure).^[17]^ Computational assessment (BP86/def2TZVPP and benzene solvation model, see SI) of the reaction indeed reveals the adduct formation to be more exergonic (−28.8 kcal mol^−1^) than the deprotonation reaction (−15.7 kcal mol^−1^). The Lewis‐acidic boron atom in **1** is significantly less sterically shielded than in previous cases of successful R_2_B−Me deprotonation (as in Mes_2_B−CH_3_ with p*K*_a_=29.0), where adduct formation is sterically impaired. In that respect, the successful deprotonation of **1** likely benefits from its increased C−H acidity (p*K*_a_=22.6).

**Figure 3:**
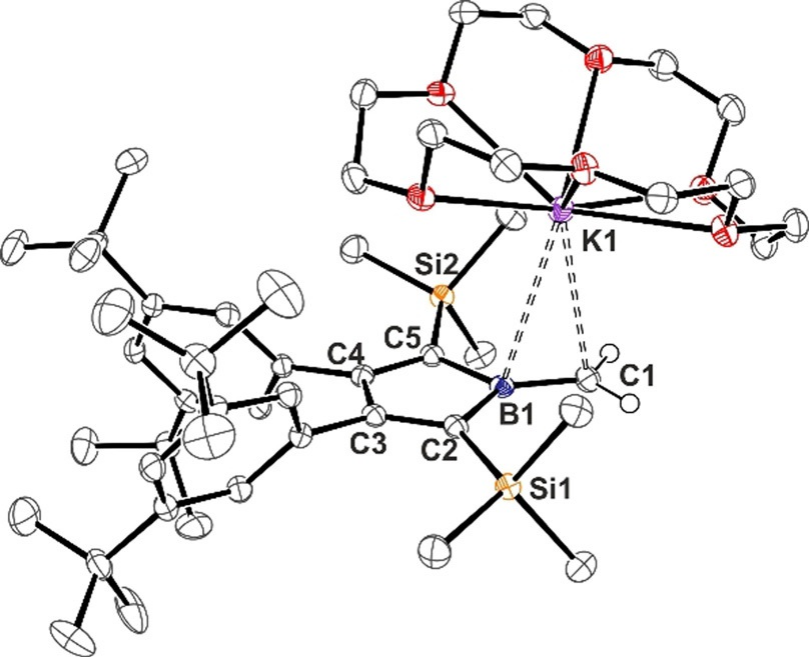
ORTEP of the molecular structure of boratafulvene **2**[K(18‐crown‐6)] (**2 a**). ADP are drawn at 50 % probability. Non‐methylene hydrogen atoms, disorder in *t*Bu groups, and lattice toluene are omitted for clarity. Selected bond lengths [Å]: B1–C1 1.457(2), B1–C2 1.602(2), C2–C3 1.367(2), C3–C4 1.501(2), C4–C5 1.370(2), C5–B1 1.601(2), C1–K1 3.210(2), K1–B1 3.322(2).

After work‐up, boratafulvene **2**[K(thf)_2_] can be isolated in small to moderate yields from THF by fractional crystallization. These crystals have been probed several times by X‐ray diffraction but only provided poor data which only allowed identification of the connectivity pattern as a coordination polymer of {**2**[K(thf)_2_}_∞_ (see SI). Adding 18‐crown‐6, **2 a** (**2**[K(18‐crown‐6)]) can be reliably isolated from toluene as crystalline material suitable for X‐ray diffraction in moderate yields (ca. 44 %). The molecular structure of **2 a** is shown in Figure 3.[^18^, ^19^] The structure reveals B=C contacts to a crown‐ether‐coordinated K‐cation. Compared to **1**, the B1−C1 bond lies in the borole plane and is significantly shortened to 1.457(2) Å as to be expected for authentic C=B in borataalkenes (C=B in [Mes_2_B=CH_2_]^−^: 1.444(8) Å).[^1^, ^11b^, ^20^] The ^11^B NMR resonance is found at 40.3 ppm highfield‐shifted relative to **1** indicating involvement of the previously empty boron p‐orbital in a B=C π‐bond. The methylene signals are found at 4.48 ppm (^1^H) and 96.1 ppm (^13^C).

**Figure 4:**
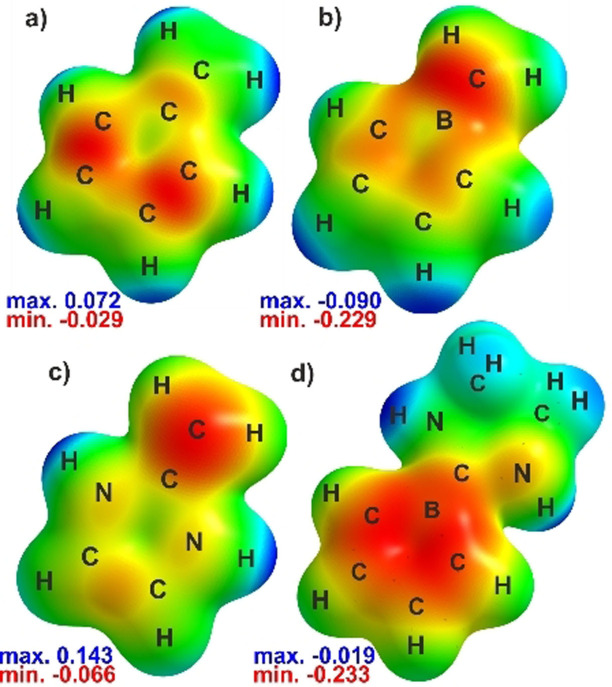
Electrostatic potential maps of parent hydrogen‐substituted a) fulvene, b) boratafulvene, c) N‐heterocyclic olefin, d) NHC‐supported borole anion at an isolevel of 0.006.

**Scheme 3:**
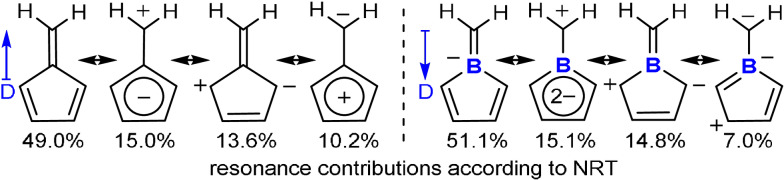
NRT comparison of fulvene versus boratafulvene.

Anion **2** is isoelectronic to (penta)fulvene and thus a rare case of a heterofulvene with an endocyclic heteroatom. Due to resonance stabilization of a negative charge in aromatic cyclopentadienyl moieties, fulvenes are polar molecules with the dipole moment aligned along the polar exocyclic C=C double bond.^[21]^ NBO analysis of boratafulvene reveals similar polarities for both, the B−C σ‐ and π‐bonds with dominant contributions (65 %) of the more electronegative carbon atom. NRT analysis further suggests very similar resonance structure contributions compared to fulvene (Scheme 3).^[22]^ Despite similar contributions of a cationic exocyclic CH_2_ group attached to an aromatic, dianionic borole ring, the dominant polar double bond resonance structure results in an inverted directionality of the dipole moment in boratafulvenes compared to fulvenes. The electrostatic potential surface map of boratafulvene thus reveals a nucleophilic site along the B=C bond and in that respect, boratafulvene more resembles the profile of N‐heterocyclic olefins (Figure 4), which have evolved as versatile C‐nucleophilic ligands in coordination chemistry.^[23]^ Respectively, (N‐heterocyclic) borataalkenes were most recently shown to be suitable ligands.[^12b^, ^24^]

Fulvene is a high energy isomer of benzene (by ca. 33 kcal mol^−1^) and accordingly, parent boratafulvene is 31 kcal mol^−1^ higher in energy than boratabenzene, of which substitution and transition‐metal complex derivatives are known.[^25^, ^26^]

**Scheme 4:**
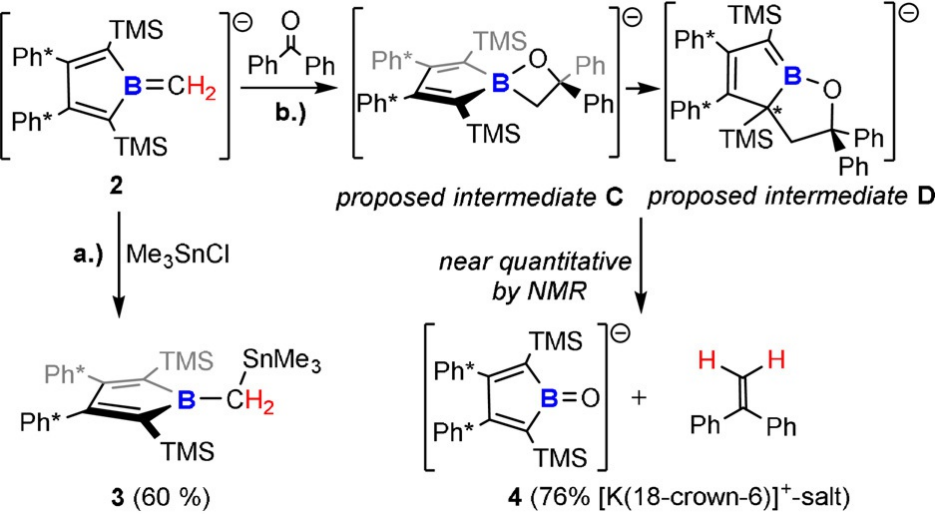
Reactivities of Boratafulvene **2**.

We treated **2** with Me_3_SnCl as a mild electrophile. As observed for borataalkenes before,[^11c^, ^11e^] **2** reacts as a carbon‐centered nucleophile to selectively give the stannaneopentyl borole **3** (Scheme 4 a). Remarkably, in the course of this reaction the unfavorable anti‐aromatic character within the borole ring is regained from a non‐aromatic precursor, as supported by a characteristic NICS_zz_ profile of **3** (see Supporting Information).^[27]^ However, on the basis of the NICS(1)_zz_ value, the anti‐aromatic character in **3** (13.0) is significantly reduced compared to **1** (24.8). Thus, boratafulvenes allow for the synthesis of functionalized alkyl‐substituted free boroles with the borole fragment being introduced as a nucleophilic reagent. It is important to stress that this new synthetic avenue can be very valuable as the known routes for the synthesis of highly reactive, anti‐aromatic free boroles, particularly the rare B‐alkyl‐substituted derivatives, are very limited. A distinct difference to formally related NHC‐supported borole anions (or rather B‐imidazolium substituted borole dianions), featuring a B−C_carbene_ single bond, must be noted as those are reported to react as one‐electron reductants or boron‐centered nucleophiles, yet for the latter not generating free boroles.[^10f^, ^28^] This differences of a π‐acidic formal methylene unit attached to the boron atom in borole anions compared to a dominantly σ‐donating N‐heterocyclic carbene is reflected in their electrostatic potential maps (Figure 4 d).

**Figure 5:**
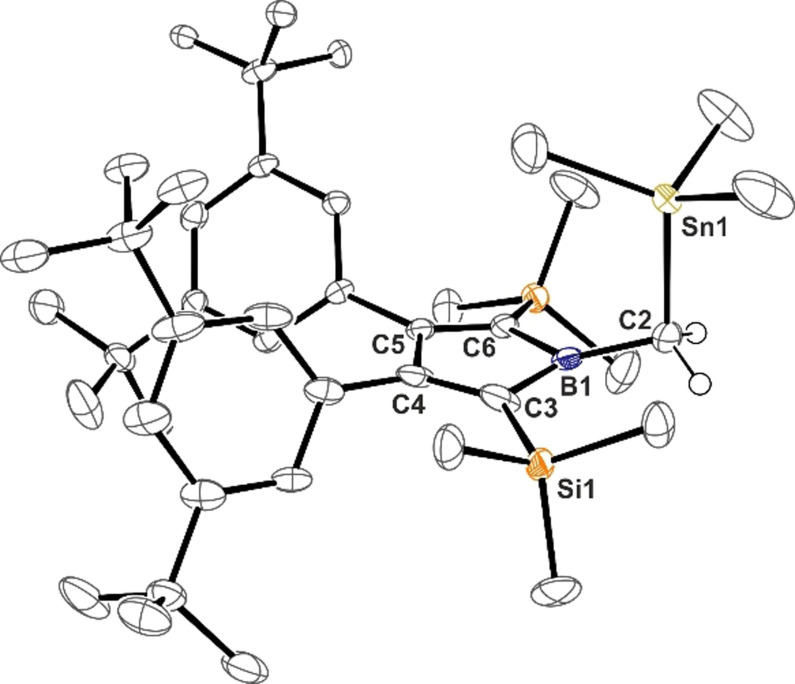
ORTEP of the molecular structure of stannaneopentyl borole **3**. ADP are drawn at 50 % probability. Non‐methylene hydrogen atoms and disorder are omitted for clarity. Selected bond lengths [Å]: B1–C2 1.496(7), B1–C3 1.597(6), C3–C4 1.346(8), C4–C5 1.525(6), C5–C6 1.354(5), C6–B1 1.617(9), C2–Sn1 2.215(3); Sn1–CH_3_ 2.073(5), 2.148(6), 2.180(9).

The molecular structure of stannaneopentyl borole **3** is depicted in Figure 5. The ‐CH_2_SnMe_3_ group is notably bent out of the borole plane by ca. 16° (C2‐B1‐(C4/C5)_centroid_ ca. 164°). The single and double bond lengths within the ring are as to be expected but the exocyclic B1–C2 distance is relatively short (1.496(7) Å) ranging between the single bond in **1** (ca. 1.56 Å) and the double bond in **2** (ca. 1.46 Å). The methylene−Sn bond is notably elongated compared to the other Sn−CH_3_ contacts and the B‐C‐Sn angle is found at 103(1)°.

The ^11^B NMR resonance of **3** is found at 68.7 ppm, which is considerably highfield‐shifted compared to other free 2,5‐disilylboroles.^[8d]^ The B‐bound methylene group resonates at 2.27 ppm (^1^H) and 24.9 ppm (^13^C). ^1^ *J*_Sn−C_ coupling amounts to 335 Hz for the CH_3_ groups (almost identical to the coupling in SnMe_4_) but only 46 Hz for the CH_2_ group. This points at reduced s‐orbital contributions involved in the C2−Sn1 bond.^[29]^ Indeed, NBO calculations suggest sp^3^ hybridization for the Sn‐atom and the methyl C‐atoms attached but only a fairly small s‐orbital contribution (10 %) of methylene C‐atom to the C2−Sn bond, thus reducing the Fermi contact (see SI for further details).

**Scheme 5:**
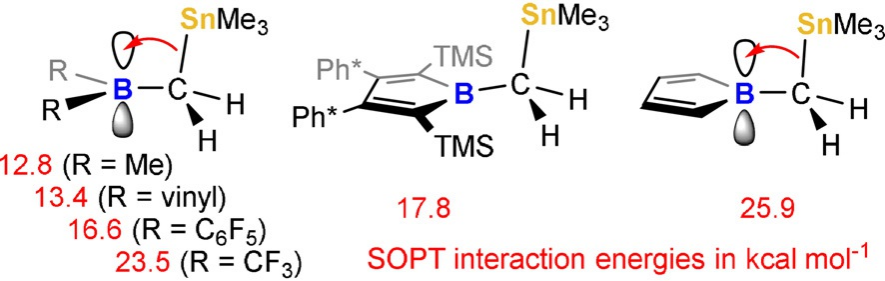
Sn−C hyperconjugation interactions with boron p‐orbital from SOPT.

A further spectroscopic feature of stannaneopentyl borole **3** is its bright yellow color. The characteristic π→π* transition in boroles is observed at *λ*_max_≈424 nm, notably blue‐shifted compared to its related methyl derivative **1** (458 nm). This correlates with an increased HOMO/LUMO gap (2.04 eV in **3**; 1.81 eV in **1**) that mainly results from an energetically elevated LUMO level.^[25]^ NBO analysis of **3** suggests a classic Lewis structure as depicted in Scheme 4, however second‐order perturbation theory (SOPT) calculations suggest significant hyperconjugation of the C−Sn σ‐bond into the empty p‐orbital of the boron atom (17.8 kcal mol^−1^).^[22]^ When this hyperconjugation is probed computationally for a series of boranes, the exceptional Lewis‐acidic character of anti‐aromatic boroles becomes apparent (Scheme 5). Acyclic boranes R_2_B(CH_2_SnMe_3_) including those with electron‐withdrawing substituents such as C_6_F_5_ groups reveal smaller respective hyperconjugation interaction energies from SOPT. Only CF_3_ groups render boranes comparably Lewis‐acidic to rival the hyperconjugation predicted in the parent borole (HC)_4_B(CH_2_SnMe_3_) in accordance with the p*K*_a_ approximations. Reduced interaction in **3** compared to the parent borole may stem from steric hindrance preventing from smaller B‐C‐Sn angles.

**Figure 6:**
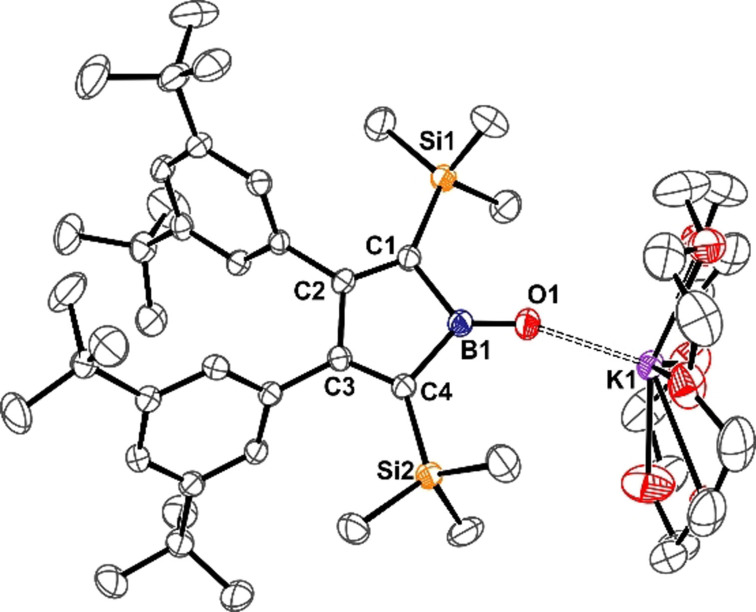
ORTEP depiction of the molecular structure of borole oxide [K(18‐crown‐6)]**4**. ADP are drawn at 50 % probability. Hydrogen atoms, lattice hexane molecule, and disorder in *t*Bu groups and the crown‐ether fragment are omitted for clarity. Selected bond lengths [Å] and angles [°]: B1–O1 1.281(3), B1–C1 1.645(4), C1–C2 1.349(2), C2–C3 1.525(3), C3–C4 1.355(3), C4–B1 1.648(3), O1–K1 2.522(2), K1–O(2‐7) 2.778(3)–2.892(2); B1‐O1‐K1 159.7, C4‐B1‐O1‐K1 46.6.

We further probed the reactivity of the boratafulvene anion **2** towards benzophenone as a model carbonyl compound and monitored the reaction by NMR spectroscopy (Scheme 4 b). After several days at room temperature, clean conversion to 1,1‐diphenylethylene and a new borole species, borole oxide **4** (the molecular structure of its [K(18‐crown‐6)]^+^ salt is shown in Figure 6) was observed, indicating that **2** serves as a methylene transfer reagent in a borata‐Wittig‐type reaction to form alkenes.

Such reactions were reported previously for borataalkenes and carbonyls.[^11c^, ^12a^, ^15^, ^30^] Preliminary analysis of the reaction mixtures by NMR spectroscopy indicates the formation of an oxaboretane intermediate **C** as the dominant species of a mixture after a few hours.^[15]^ The methylene CH_2_ signals are observed highfield‐shifted at 2.82 ppm (^1^H) and 24.3 ppm (^13^C via HSQC), indicating a saturated species with the ^11^B resonance at 11.2 ppm strongly advocating for a tetracoordinate boron atom and thus the four‐membered cycle. Along with slowly increasing amounts of **4** and 1,1‐diphenylethylene, intermediate presence of species lacking mirror‐plane symmetry and revealing two diastereotopic protons of the methylene group is observed, plausibly yet putatively assigned to a ring‐expanded oxaborolane **D**. The observation of individual intermediates seems to be dependent on solvent (benzene vs. THF) and presence of 18‐crown‐6, yet in each case clean conversion to **4** and 1,1‐diphenylethylene is reached eventually after two weeks. According to NBO analysis, borole oxide **4** is an oxoborane best represented by the Lewis structure depicted in Scheme 4 with a B=O double bond and the short B–O distance of 1.281(3) Å lies well in between those recently reported for neutral (1.2867(16) Å) or anionic (1.273(8) Å) acid‐free azaborole‐derived oxoboranes that were discussed as “bora carbonyls”, but longer than in a most recent entry (1.256(3) Å) by Xie and co‐workers.^[31]^ The K1–O1 distance is found at 2.522(2) Å, in the range of distances observed in a related, yet dimeric potassium salt of a diazaborole oxide (2.47–2.59 Å).^[32]^

Computationally (BP86/def2TZVPP and benzene solvation model, see SI), the overall reaction of **2** and benzophenone to form **4** and diphenylethylene is predicted to be exergonic (−27.3 kcal mol^−1^). In line with the proposed reaction progress, formation of oxaboretan **C** (−10.6 kcal mol^−1^) and its putative subsequent rearrangement to oxaborolane **D** (−14.4 kcal mol^−1^), as well as their respective reactions to the final products **4** and diphenylethylene are exergonic (**C**: −16.7 kcal mol^−1^; **D**: −2.2 kcal mol^−1^).

## Conclusion

In summary, we presented the first synthesis of a boratafulvene by deprotonation of methyl borole. According to computational p*K*_a_ approximations, anti‐aromaticity of boroles increases α‐CH acidity to a similar extent as strongly electron‐withdrawing fluorinated substituents. A first example for the suitability of boratafulvenes as nucleophilic reagents to generate free boroles is demonstrated. Borata‐Wittig reactivity as methylene transfer reagent was observed that also leads to a yet unprecedented borole oxide.

## Experimental Section

Experimental details and analytical data as well as computational details are documented in the Supporting Information. Crystallographic information files (CIF) for compounds **1**, **B**, **2 a**, **2 d**, **3**, and **4** have been deposited at the Cambridge Structural Database (Deposition numbers 2081057, 2081058, 2081059, 2081060, 2081061 and 2081062) where they can be obtained free of charge.

## Conflict of interest

The authors declare no conflict of interest.

## Supporting information

As a service to our authors and readers, this journal provides supporting information supplied by the authors. Such materials are peer reviewed and may be re‐organized for online delivery, but are not copy‐edited or typeset. Technical support issues arising from supporting information (other than missing files) should be addressed to the authors.

Supporting Information

Supporting Information
