# Supplementary material for: A Boratafulvene
Source: Angew Chem Int Ed Engl. 2021 Aug 6;60(36):20055–60. doi: 10.1002/anie.202107968 (PMC8456924; doi:10.1002/anie.202107968)
Supplement: Supplementary file 2 — Supporting Information [file ANIE-60-20055-s002.pdf]

Angewandte Chemie
Eine Zeitschrift der Gesellschaft Deutscher Chemiker

Supporting Information

A Boratafulvene

*Tobias Heitkemper, Leonard Naß, and Christian P. Sindlinger**

anie_202107968_sm_miscellaneous_information.pdf

Supporting Information

Table of Contents

Experimental Details 3

General Information 3

Mass spectrometry 3

NMR spectroscopy 3

Starting materials and reagents 3

Synthesis and Analytical Data 4

Compound 1 4

Analytical Data for Compound 1 4

Crystal structure of Compound 1 5

Spectra Plots for Compound 1 6

Potassium Salts of Compound 2 (2a-c) 9

(2a): [K(18-crown-6)]2 9

(2b): [K(THF)$_2$]2 9

(2c): [K(THF)$_2$(18-crown-6)]2 10

Analytical Data for Compound [K(18-crown-6)]2 (2a) 10

Crystal structure of Compound [K(18-crown-6)]2 (2a) 10

Spectra Plots for Compound [K(18-crown-6)]2 (2a) 11

^{1}H-NMR Data for Compound [K(THF)$_2$]2 (2b) 14

Preliminary crystal structure of Compound [K(THF)$_2$]2 (2b) 14

^{1}H- NMR Data for Compound [K(18-crown-6)(THF)$_2$]2 (2c) 15

Preliminary crystal structure of Compound [K(18-crown-6)(THF)$_2$]2 (2c) 15

Sodium Salts of Compound 2 (2d,e) 16

(2d): [Na(18-crown-6)]2 16

(2e): [Na(THF)$_2$]2 16

Analytical Data for Compound [Na(18-crown-6)]2 (2d) 17

Crystal structure of Compound [Na(18-crown-6)]2 (2d) 17

Spectra Plots for Compound [Na(18-crown-6)]2 (2d) 18

NMR Data for Compound [Na(THF)$_2$]2 (2e) 20

Preliminary Crystal structure of Compound [Na(THF)$_2$]2 (2e) 20

Spectra Plots for Compound [Na(THF)$_2$]2 (2e) 21

Side Product B [1(HMDS)]K(18-crown-6)] 23

Analytical Data for Compound [K(18-crown-6)]B 23

Crystal structure of Compound [K(18-crown-6)]B 24

Spectra Plots for Side Product B [1(HMDS)]K(18-crown-6)] 24

Compound 3 27

Analytical Data for Compound 3 27
Crystal structure of Compound 3 28
Spectra Plots for Compound 3 28
NMR Reaction of 2 with Benzophenone 32
NMR spectra monitoring the Borata-Wittig Reaction with Benzophenone 33
Compound [K(18-crown-6)]4 37
Analytical Data for Compound [K(18-crown-6)]4 37
Crystal structure of Compound [K(18-crown-6)]4 38
Spectra Plots for Compound [K(18-crown-6)]4 38
Crystallographic Details 41
General Data Acquisition and Processing 41
Crystallographic and Refinement Details 1 41
Crystallographic and Refinement Details 2a 41
Crystallographic and Refinement Details 2d 42
Crystallographic and Refinement Details 3 42
Crystallographic and Refinement Details B 42
Crystallographic and Refinement Details [K(18-crown-6)]4 43
Tabulated Crystallographic Details 1,2(a,d), 3, B and [K(18-crown-6)]4 44
Computational Details 45
Structure Optimisation, Frequency Calculation and Thermochemical Approximations 45
Frontier Orbital Details and Bonding Indices for 1,2,3 and 4 46
NBO and NRT Analyses 47
TD-DFT 48
Computational Approximations of pK_a values for α-CH bonds in boranes and boroles. 50
NICS$_{zz}$-Profile 52
XYZ-coordinates of optimised structures 53
Literature 68

Experimental Details

General Information

All manipulations requiring handling under inert conditions were carried out under argon atmosphere using standard Schlenk techniques or an MBraun Glovebox with an Ar atmosphere. Benzene was obtained from an MBraun SPS and stored over molecular sieves, toluene and ether were distilled from sodium. Hexane and pentane were distilled from Na/K alloy. THF was distilled from potassium. THF-d_8 was dried over $LiAlD_4$ and vacuum transferred, benzene-d_6 was distilled from potassium, and solvents were degassed and stored in a glove box. All solvents were routinely degassed three times using freeze-pump-thaw cycles. UV/Vis spectra were recorded in cuvettes equipped with a Young-type teflon valve on a Perkin Elmer Lambda 25 Instrument.

Elemental analyses were performed by the Analytisches Labor, Institut für Anorganische Chemie, Universität Göttingen

Mass spectrometry

Mass spectra were recorded by the Zentrale Analytik within the Faculty of Chemistry, Göttingen applying a Liquid Injection Field Desorption Ionisation-technique on a JEOL accuTOF instrument with an inert-sample application setup under argon atmosphere. The injection capillary was washed several times with dry, distilled and inertly injected toluene or THF before the samples were injected. Samples usually had a concentration of 1 – 2 mmol L^{-1}. This set-up only allows detection of positively charged ions. Anionic species were submitted in sealed vials as solutions in dry, degassed THF and probed on a ESI-MS setup on a Thermo Scientific LTQ-Orbitrap instrument without further inert manipulation/injection set-up.

NMR spectroscopy

NMR spectra were recorded with either a Bruker Avance III 400 NMR spectrometer equipped with a 5 mm BBFO ATM probe head and operating at 400.13 (^{1}H), 100.61 (^{13}C), 128.38 (^{11}B) and 376.45 MHz (^{19}F) along with a variable temperature set-up or a Bruker Avance Neo 400 NMR spectrometer with a CryoProbeProdigy BB ATM probe head operating at 400.25 MHz (^{1}H) and 100.65 MHz (^{13}C) or a Bruker AVIII HD 500 NMR spectrometer with a CryoProbeProdigy ATM probe head and operating at 500.25 (^{1}H), 125.80 (^{13}C), 160.50 MHz (^{11}B) and 99.37 MHz (^{29}Si) or a Bruker Avance III HD spectrometer operating at 116.64 MHz (^{7}Li). Chemical shifts are reported in δ values in ppm relative to external Me_4Si and, if not otherwise stated, referenced using the chemical shift of the solvent ^{2}H lock resonance frequency and Ξ = 19.867187% for ^{29}Si, Ξ = 38.863797 % for ^{7}Li, Ξ = 32.083974% for ^{11}B, and Ξ = 37.290632% for ^{119}Sn.[1] ^{1}H- and ^{13}C spectra have been referenced on specific values for the respective solvent signal. The proton and carbon signals were assigned where possible via a detailed analysis of ^{1}H, ^{13}C, ^{1}H-^{1}H COSY, ^{1}H-^{1}H NOESY, ^{1}H-^{13}C HSQC, ^{1}H-^{13}C HMBC NMR spectra.

Young-type teflon-valve borosilicate NMR tubes have been used throughout the study.

Starting materials and reagents

1-Chloro-3,4-(2',5'-*t*$Bu_2(C_6H_3)$)-2,5-($SiMe_3$)-Borole **A** was prepared as recently reported.[2]

MeMgBr or MeMgI (3.0 M in Et_2O) were obtained from Sigma Aldrich and used as received. Concentration was checked regularly by hydrolysis and titration. $Na[N(SiMe_3)_2]$, $K[N(SiMe_3)_2]$ and [18-crown-6] were purchased from Sigma Aldrich, stored in a glovebox and used as received. Benzophenone was sublimed prior to use. Li(2,2,6,6-tetramethylpiperidide) (LiTMP) was prepared according to the literature.[3]

Synthesis and Analytical Data

Compound 1

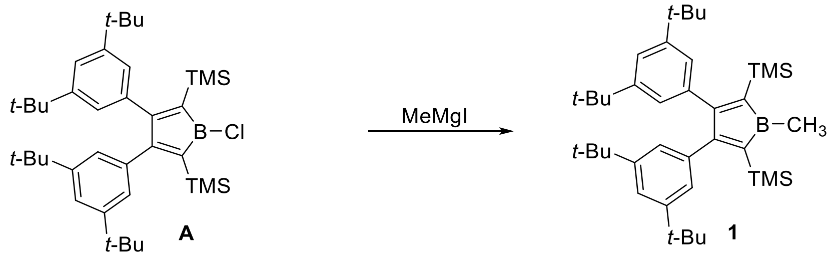

Molecular Weight: 619,33 Molecular Weight: 598,91

In a glovebox, chloro borole **A** (454.8 mg, 0.734 mmol, 1 eq) was dissolved in dry and degassed diethyl ether (20 mL). MeMgI (0.237 mL, 3 M in Et_2O, 0.712 mmol, 0.97 eq.) was added to this solution and the reaction mixture was stirred for two hours at ambient temperature. The solvent was removed under reduced pressure and the resulting red powder was placed into a syringe equipped with a pad of glass fiber (Whatman GF/B) and extracted with hexane (in toto ca. 42 mL), until the remaining solid was of a white colour. The solvent was removed under reduced pressure to yield a red powder (464 mg). ^{1}H-NMR spectroscopy of this crude solid revealed to be compound **1** contaminated with only minor impurities (approximately 3 %). Recrystallization from hot toluene (ca. 100 °C, 4.6 mL, ca. 1 mL/100 mg) yielded red crystals of compound **1** (285.4 mg, 0.476 mmol, 65%). The mother liquor was decanted off and stored at –40 °C for several days, to afford a second crop of red crystals of compound **1** (71.5 mg, 0.119 mmol, 16%). Isolated crystal crops were dried under reduced pressure.

Analytical Data for Compound 1

NMR:

^{1}H (300.13 MHz, 298 K, C_6D_6, CD_5H at 7.15 ppm): 7.24 (t, $^4J_{HH}$ = 1.9 Hz, 2H, *p-H$_{ar}$*), 6.81 (d, $^4J_{HH}$ = 1.9 Hz, 4H, *o-H$_{ar}$*), 1.32 (s, 3H, B-C*H*$_3$), 1.16 (s, 36H, Ar-C(*Me*)$_3$), 0.11 (s, 18H, Si(*Me*)$_3$).

^{13}C{^{1}H} (100.65 MHz, 298 K, C_6D_6, solvent signal at 128.0 ppm): 179.7 (borole-*C$_{3,4}$*), 149.5 (*m-C$_{ar}$*), 139.6 (*ipso-C$_{ar}$*), 139.0 (borole-*C$_{2,5}$*), 122.9 (*o-C$_{ar}$*), 120.8 (*p-C$_{ar}$*), 34.7 (Ar-*C*(CH$_3$)$_3$), 31.5 (Ar-C(*C*H$_3$)$_3$), 11.9 (B-*C*H$_3$), 1.3 (Si(*C*H$_3$)$_3$).

^{11}B (128.38 MHz, 298 K, C_6D_6): 80.2.

^{29}Si-INEPT (79.49 MHz, 298 K, C_6D_6): –9.7.

UV VIS: in pentane, λ_{max} at 458 nm

Elemental Analysis: $C_{39}H_{63}BSi_2$ calcd C 78.21, H 10.60; observed C 78.02, H 11.20.

LIFDI-MS: calcd exact mass: 598.5 m/z; observed m/z: 616.6 $[M+H_2O]^+$.

Crystal structure of Compound 1

For further details on the diffraction measurement please see the respective section. Crystals investigated using XRD were obtained from solutions in hexane in a freezer (-40°C).

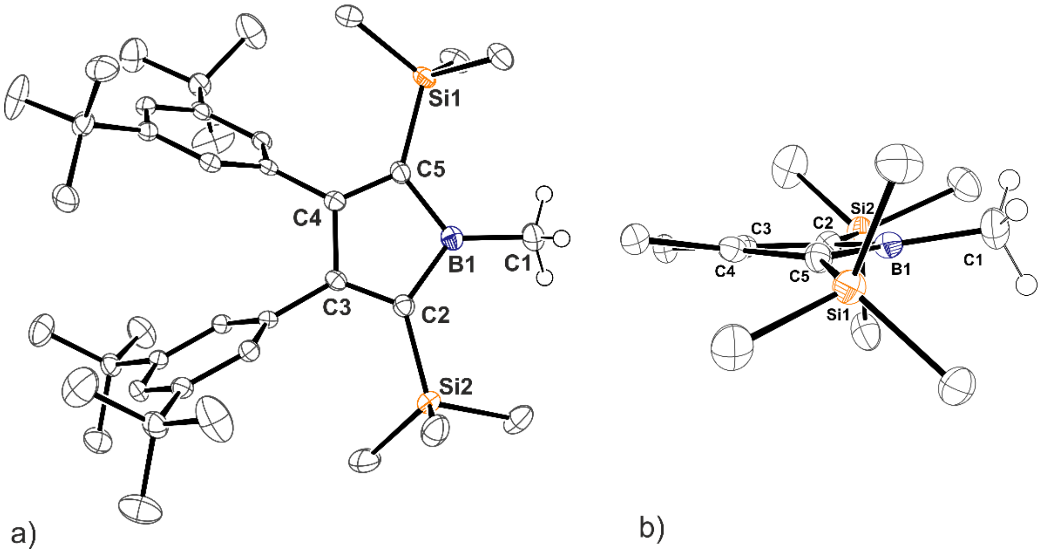

SI-Figure 1-SI ORTEP plot of the molecular structure of **1** [a) Full representation, b) excerpt]. Atomic displacement parameters are drawn at 50% probability level. Hydrogen atoms except for C1-bound H are omitted for the sake of clarity. Selected bond length in Å: B1-C1 1.559(2), B1-C2 1.595(2), C2-C3 1.357(2), C3-C4 1.539(2), C4-C5 1.354(2), C5-B1 1.587(2); C2-Si2 1.871(2); C5-Si1 1.866(2). The structure was deposited with the CCSD.

Spectra Plots for Compound 1

1H-NMR-spectrum of compound 1 in C6D6

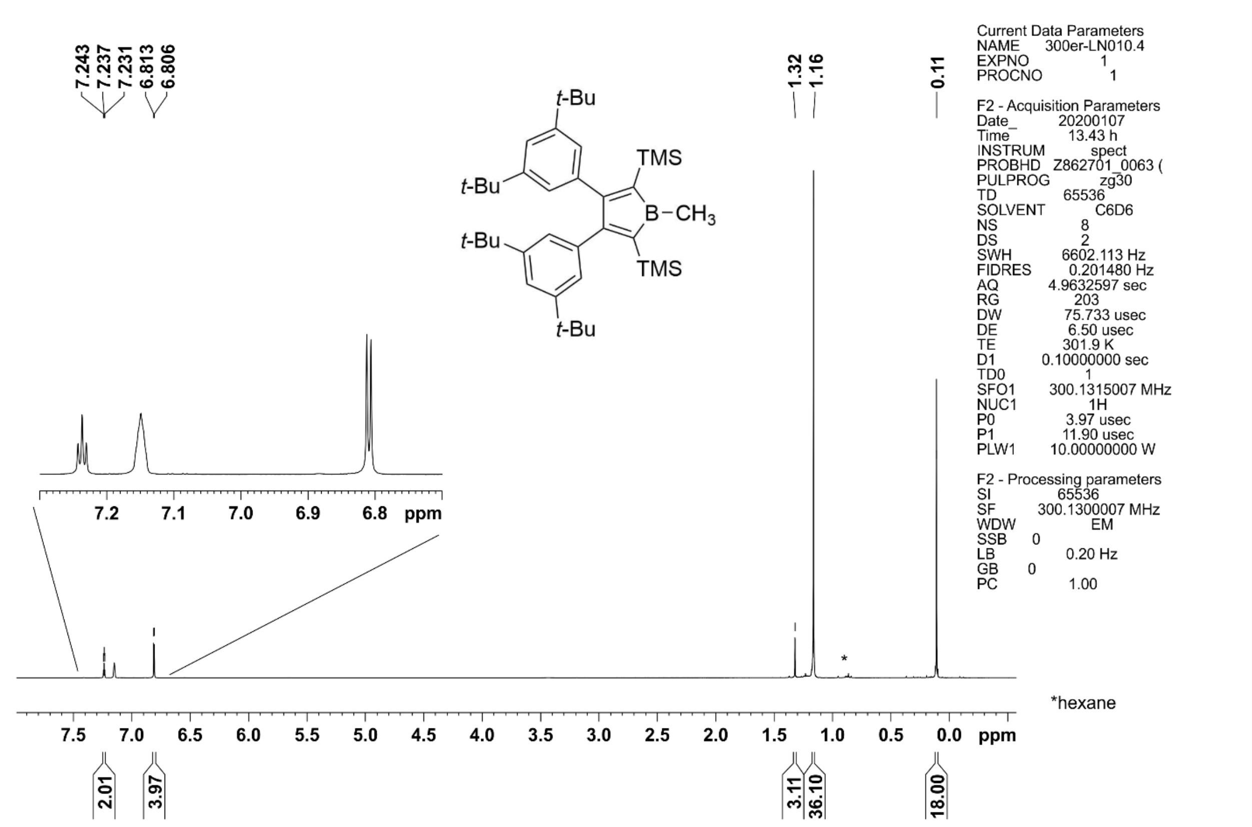

13C{1H}-NMR-spectrum of compound 1 in C6D6

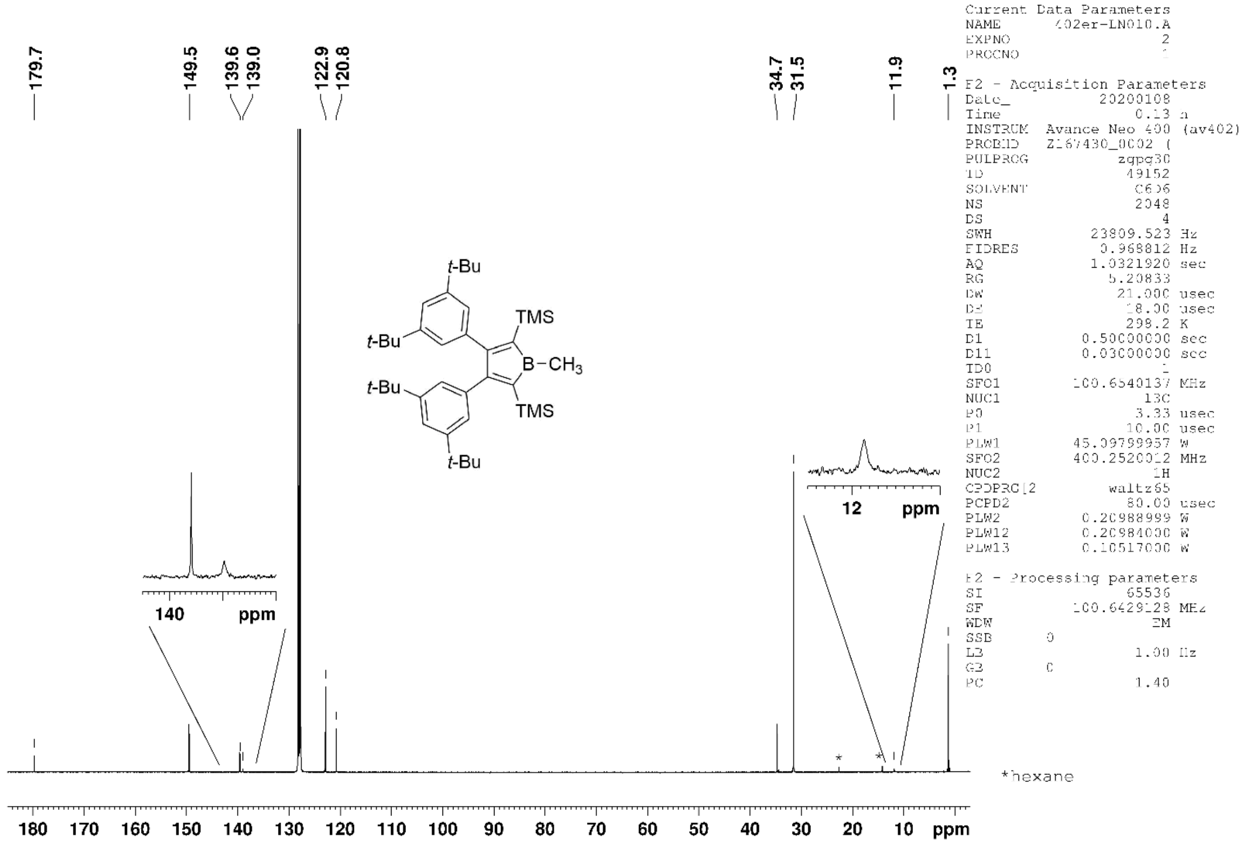

11B-NMR spectrum (background suppressed) of compound 1 in C6D6

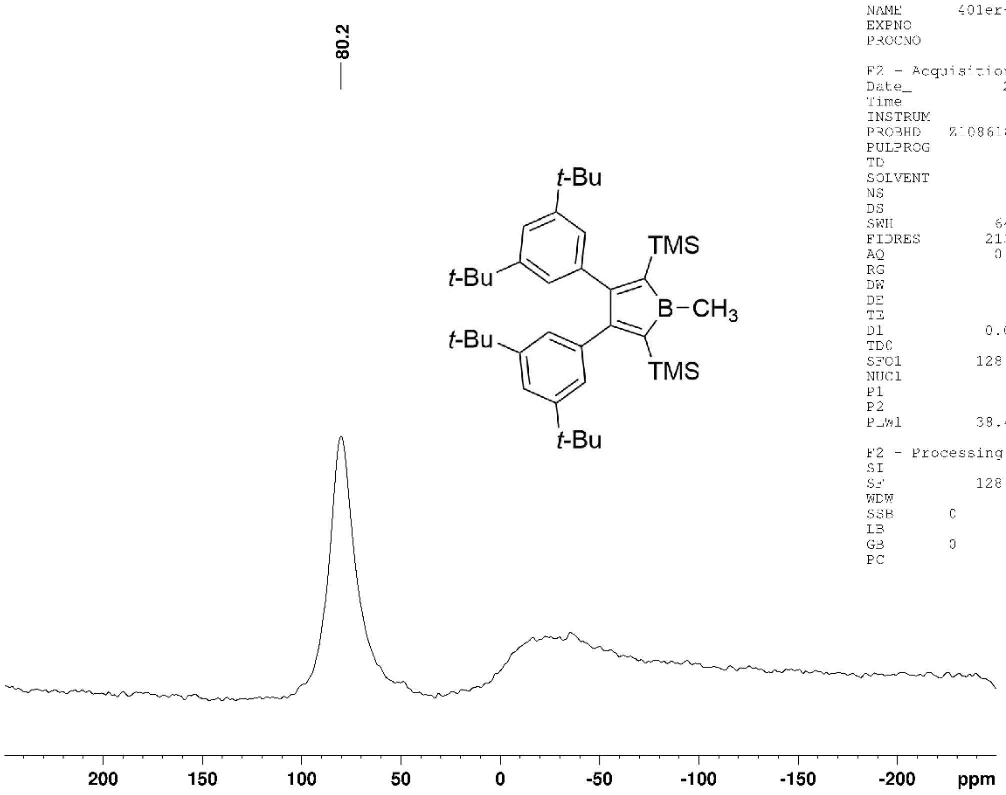


```
Current Data Parameters
NAME        401er-LN010.A
EXPNO                   2
PROCNO                  1

F2 - Acquisition Parameters
Date_            20200109
Time                17.03 h
INSTRUM             spect
PROBHD   Z108618_0095 (
PULPROG              zgbs
TD                    600
SOLVENT              C6D6
NS                   2261
DS                     32
SWH             64102.563 Hz
FIDRES         213.675217 Hz
AQ              0.0046800 sec
RG                    256
DW                  7.800 usec
DE                   6.50 usec
TE                  298.8 K
D1             0.00600000 sec
TD0                   256
SFO1          128.3776050 MHz
NUC1                  11B
P1                   9.50 usec
P2                  19.00 usec
PLW1          38.45899963 W

F2 - Processing parameters
SI                   1024
SF            128.3776050 MHz
WDW                    EM
SSB      0
LB                 200.00 Hz
GB       0
PC                   2.00
```

29Si-INEPT-NMR spectrum of compound 1 in C6D6

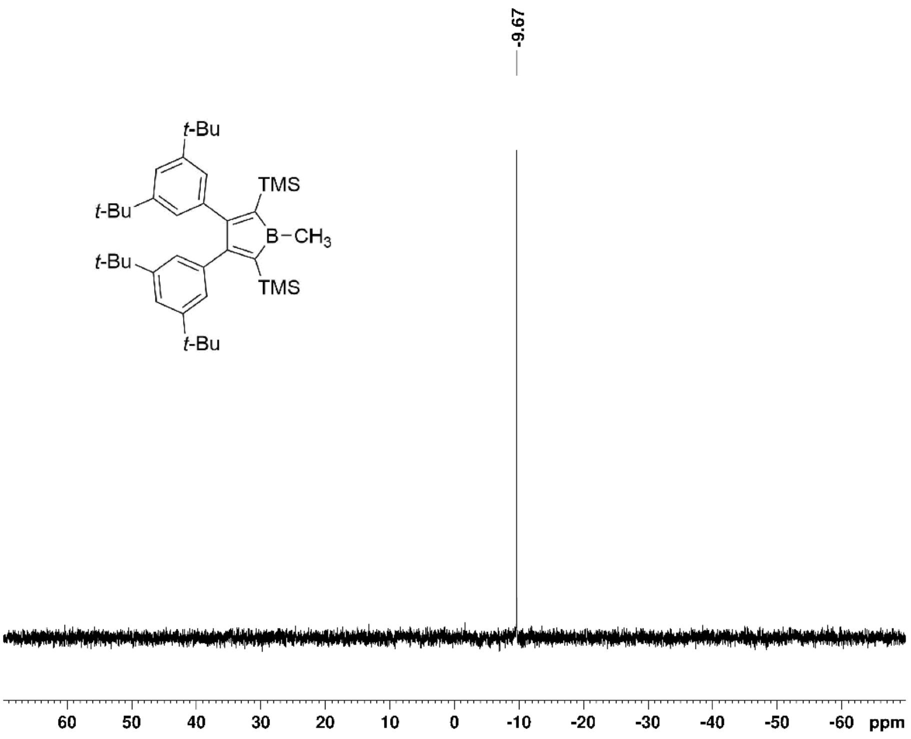


```
Current Data Parameters
NAME        401er-LN010.A
EXPNO                   3
PROCNO                  1

F2 - Acquisition Parameters
Date_            20200109
Time                17.09 h
INSTRUM             spect
PROBHD   Z108618_0095 (
PULPROG           ineptrd
TD                  32768
SOLVENT              C6D6
NS                     28
DS                      8
SWH             11111.111 Hz
FIDRES           0.678168 Hz
AQ              1.4745600 sec
RG                   2050
DW                 45.000 usec
DE                   6.50 usec
TE                  299.1 K
CNST2           6.0000000
CNST11         18.0000000
D1             3.00000000 sec
D3             0.00925926 sec
D4             0.04166667 sec
D12            0.00002000 sec
TD0                    64
SFO1           79.4945750 MHz
NUC1                 29Si
P1                  12.00 usec
P2                  24.00 usec
PLW1          63.09600067 W
SFO2          400.1320007 MHz
NUC2                   1H
CPDPRG[2          waltz16
P3                  10.10 usec
P4                  20.20 usec
PCPD2               90.00 usec
PLW2          27.73299980 W
PLW12          0.34926000 W

F2 - Processing parameters
SI                 131072
SF             79.4945750 MHz
WDW                    EM
SSB      0
LB                   1.00 Hz
GB       0
PC                   3.00
```

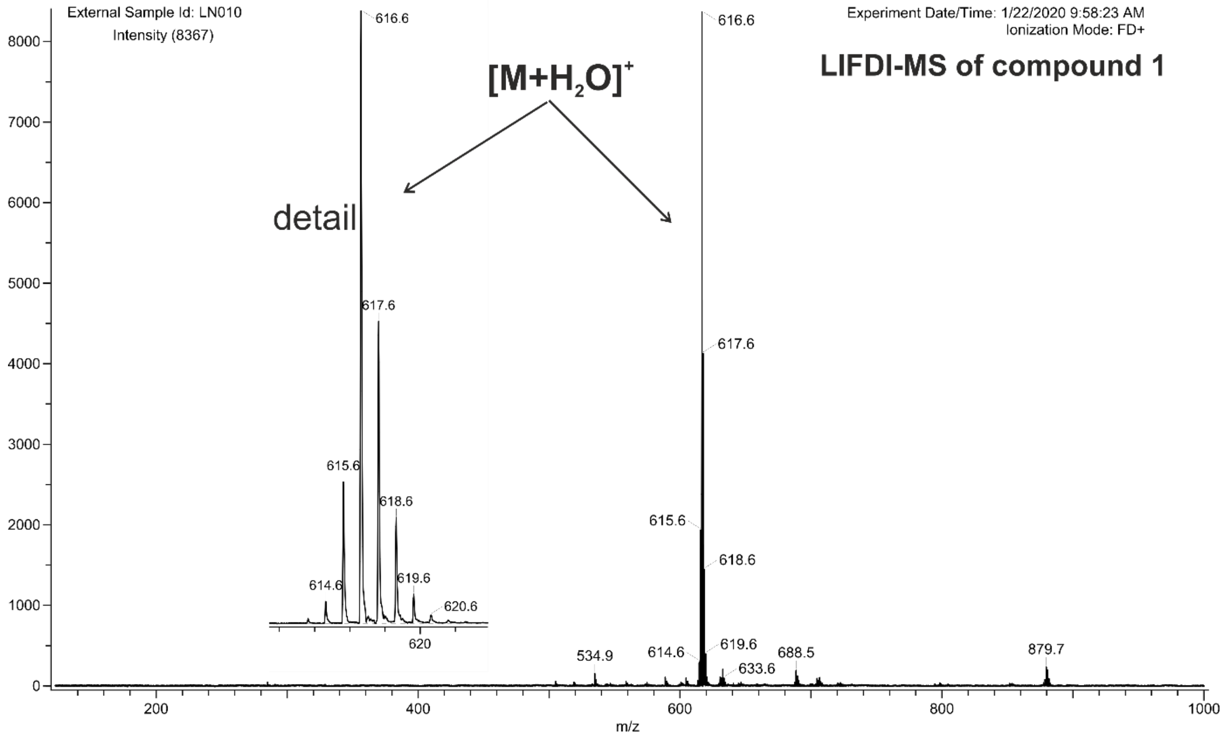
External Sample Id: LN010
Intensity (8367)
Experiment Date/Time: 1/22/2020 9:58:23 AM
Ionization Mode: FD+
LIFDI-MS of compound 1
[M+H2O]+
detail
616.6
617.6
615.6
618.6
614.6
619.6
620.6
534.9
633.6
688.5
879.7
m/z

Potassium Salts of Compound 2 (2a-c)

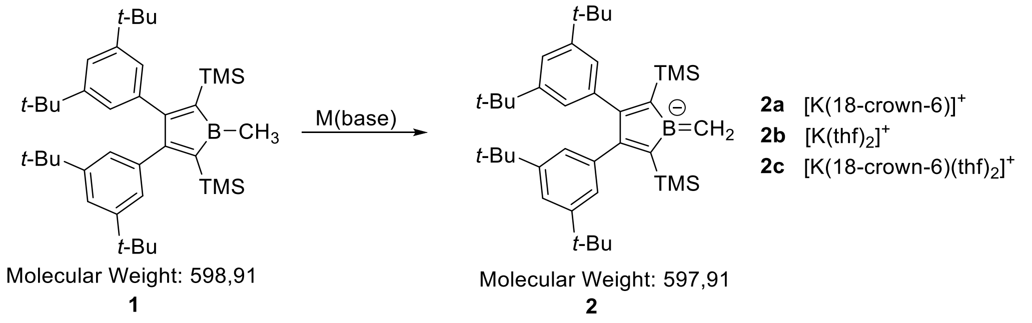

In a typical procedure: In a glovebox, compound methyl borole **1** (99.7 mg, 0.166 mmol, 1 eq) and KHMDS (33.2 mg, 0.166 mmol, 1 eq.) (HMDS = $N(SiMe_3)_2$) were dissolved in dry and degassed toluene (10 mL) and the initially red solution was stirred at ambient temperature for 16 h. Over the course of the reaction, the colour of the solution turned dark green and a yellow solid precipitated. The reaction mixture was filtered through a syringe equipped with a pad of glass fiber (Whatman GF/B) and the solid was washed with hexane (1 × 0.5 mL). The remaining yellow solid was rinsed from the filter with THF (4 mL) and the solvent was subsequently removed under reduced pressure to yield a yellow solid (ca. 70 mg). NMR examination (THF-*d*$_8$) of this solid revealed to be $[K(THF)_x]^+$ salts of boratafulvene anion **2** as the main borole derived component, but in varying amounts (0-30%) the mixture also contained amide adduct [**1·HMDS**] (**B**) and a further C_{2v}-symmetric borole derived side-product **E** of yet unknown constitution. Isolation of **2** from these mixtures is possible by fractional crystallization under various conditions:

Note: i) ^{1}H NMR spectroscopic finger prints of frequently observed side-products/impurities of **2** are: **E** in THF-d_8 [–0.10 ppm (Si*Me*$_3$), 1.12 ppm (C*Me*$_3$ perfectly superimposed with the respective signal in **2**), 6.99 ppm (*p*-C*H*$_{aromatic}$)]; **E** in C_6D_6 [0.45 ppm (Si*Me*$_3$, careful as can appear as a shoulder of the signal of **2**), 1.32 ppm (C*Me*$_3$, careful as can appear as a shoulder of the signal of **2**)]; for spectra of **B** see the respective analytical section below. ii.) When the reaction is carried out in THF rather than toluene or benzene the amide adduct **B** is the by far dominating product in the resulting mixtures (ca. 3 **B** : 1 **2**) iii) when re-crystallised samples of starting material **1** were used for this deprotonation reaction, the amount of amide adduct **B** as a side-product seemed to be drastically smaller than when crude crops of **1** (see above) were subjected. iv) when 18-crown-6 was already present at the deprotonation step, the reaction fails and only intractable mixtures are obtained.

(2a): [K(18-crown-6)]2

(**2a**) [K(18-crown-6)]**2**: The most reliable approach was to isolate the crown ether-adduct **2a** [K(18-crown-6)]**2**. To the crude solid (ca 70 mg) described above, 18-crown-6 (24 mg, 0.091 mmol) was added and the solids were dissolved in toluene (1 mL). The solvent is again removed under reduced pressure to also ensure co-evaporation of remaining THF. The solid is then re-dissolved in toluene (ca. 7 mL) and solutions are stored at –40°C for a day. **2a** [K(18-crown-6)]**2** can then be obtained as pale-yellow needle shaped crystals (66.2 mg, 0.074 mmol, 44% with regards to starting material borole **1). Some batches may still** contain ca. 5% (by NMR) of the yet unidentified side-product **E** and need to be further recrystallized.

(2b): [K(THF)$_2$]2

(**2b**) $[K(THF)_2]$**2**: From concentrated THF solutions of the crude yellow solid at –40°C, small amounts of $[K(THF)_2]$**2** can be obtained as pale yellow plate-shaped crystals. X-ray diffraction studies on these crystals repeatedly gave very poor data sets and crystals were notoriously twinned. Yet, the connectivity pattern could unambiguously be identified from the structure (see below).

(2c): [K(THF)$_2$(18-crown-6)]2

(**2c**) [K(THF)$_2$(18-crown-6)]**2**: 18-crown-6 (10 mg) is added to concentrate THF (ca. 0.5 mL) solutions of the crude yellow solid (ca. 25 mg), the mixture is layered with pentane (3 mL) and the mixture is stored at –40°C. Over the course of several days, small amounts of yellow crystalline material can be isolated. The material was of poor crystallinity and X-ray diffraction studies on these crystals repeatedly gave very poor data sets. Yet again, the connectivity pattern could unambiguously be identified to comprise a potassium cation that is coordinated by a crown-ether molecule and two axial THF molecules without direct contact to the boratafulvene anion. (see below).

Analytical Data for Compound [K(18-crown-6)]2 (2a)

NMR:

^{1}H (400.13 MHz, 298 K, C_6D_6, CD_5H at 7.15 ppm): 7.23 (t, $^4J_{HH}$ = 1.9 Hz, 2H, *p-H$_{ar}$*), 7.08 (d, $^4J_{HH}$ = 1.9 Hz, 4H, *o-H$_{ar}$*), 4.48 (s, 2H, B-C*H*$_2$), 3.19 (s, 24H, OC*H*$_2$CH$_2$), 1.33 (s, 36H, Ar-C(*Me*)$_3$), 0.45 (s, 18H, Si(*Me*)$_3$).

^{13}C{^{1}H} (100.65 MHz, 298 K, C_6D_6, solvent signal at 128.0 ppm): 164.3 (borole-*C$_{3,4}$*), 148.1 (*m-C$_{ar}$*), 145.6 (borole-*C$_{2,5}$*, superimposed by ipso-C$_{ar}$-Signal, assigned via HMBC), 145.5 (*ipso-C$_{ar}$*), 125.0 (*o-C$_{ar}$*), 117.8 (*p-C$_{ar}$*), 96.1 (B-*C*H$_2$), 69.8 (O*C*H$_2$CH$_2$), 34.8 (Ar-*C*(CH$_3$)$_3$), 32.0 (Ar-C(*C*H$_3$)$_3$), 4.2 (Si(*C*H$_3$)$_3$).

^{11}B (128.38 MHz, 298 K, C_6D_6): 40.3.

^{29}Si-INEPT (79.49 MHz, 298 K, C_6D_6): –11.8.

Elemental Analysis: 2a: $C_{51}H_{86}BSi_2KO_6$ calcd C 67.96, H 9.62; (**2a×1 toluene):** $C_{58}H_{94}BSi_2KO_6$ calcd C 70.12, H 9.54; observed C 67.87, H 9.77. *Note*: sample submitted to EA still contained <1 equiv. of toluene according to NMR.

ESI-HRMS: calcd HR-mass [**2**+MeOH]$^-$ ($C_{39}H_{62}BSi_2$ + H_3COH) 629.4758 m/z; observed 629.4762 m/z [M+MeOH]$^-$.

Crystal structure of Compound [K(18-crown-6)]2 (2a)

For further details on the diffraction measurement please see the respective section. Crystals investigated using XRD were obtained from solutions in toluene in a freezer (-40°C).

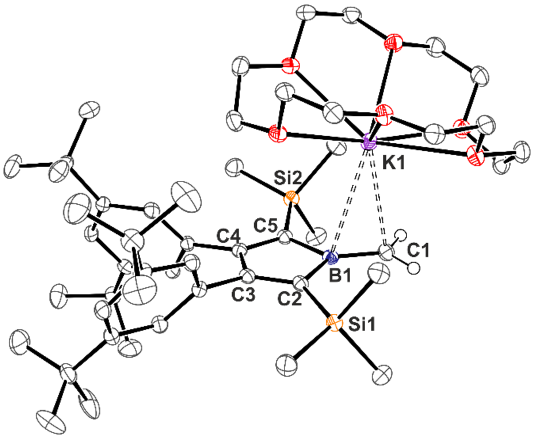

SI-Figure 2-SI ORTEP of the molecular structure of boratafulvene **2**[K(18-crown-6)] (**2a**). ADP are drawn at 50% probability. Non-methylene hydrogen atoms, disorder in *t*Bu groups and lattice toluene are omitted for clarity. Selected bond lengths [Å]: B1–C1 1.457(2), B1–C2 1.602(2), C2–C3 1.367(2)m, C3–C4 1.501(2), C4–C5 1.370(2), C5–B1 1.601(2), C1–K1 3.210(2), K1–B1 3.322(2).

Spectra Plots for Compound [K(18-crown-6)]2 (2a)

1H-NMR spectrum of compound 2a in C6D6

referenced to C6D5H at 7.15 ppm

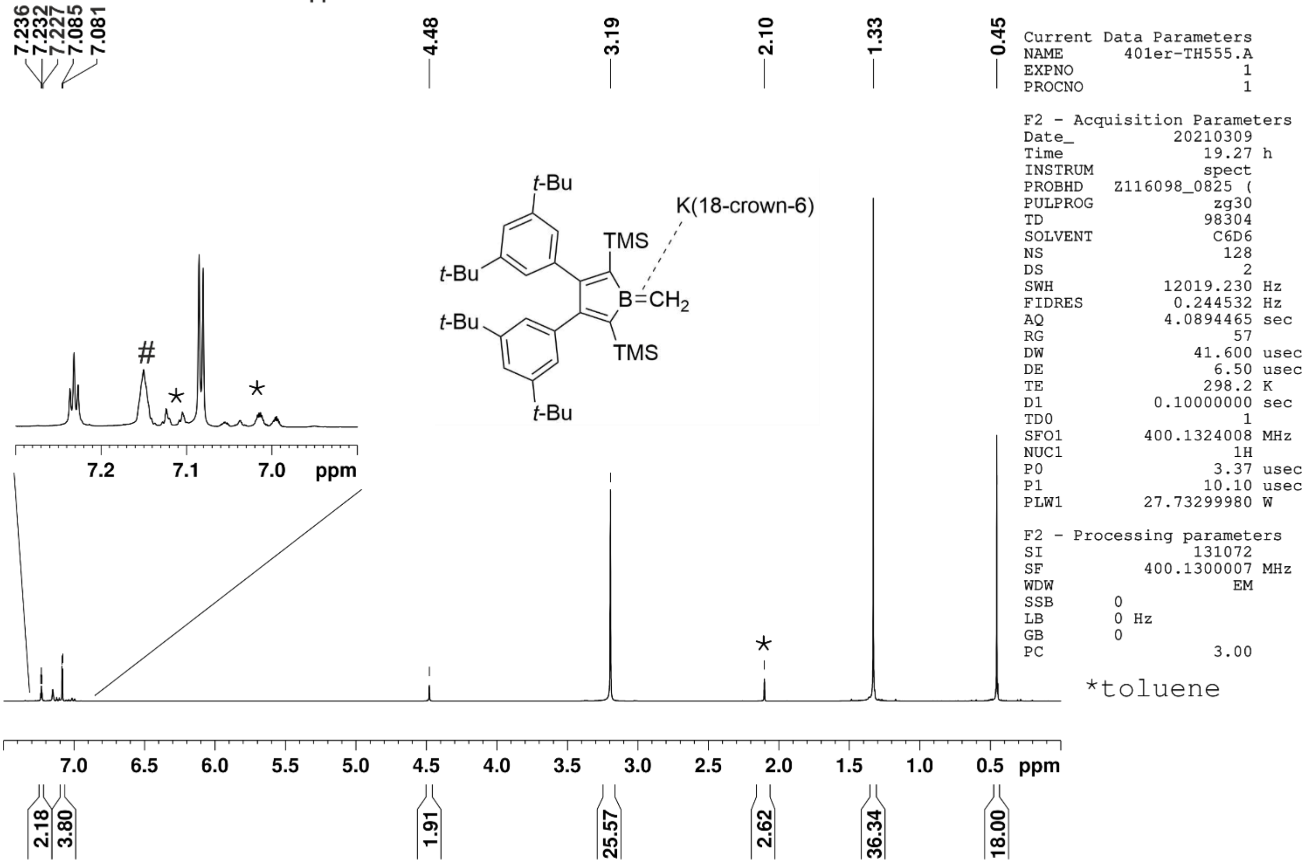

13C{1H}-NMR spectrum of compound 2a in C6D6

referenced to C6D6 at 128.0 ppm

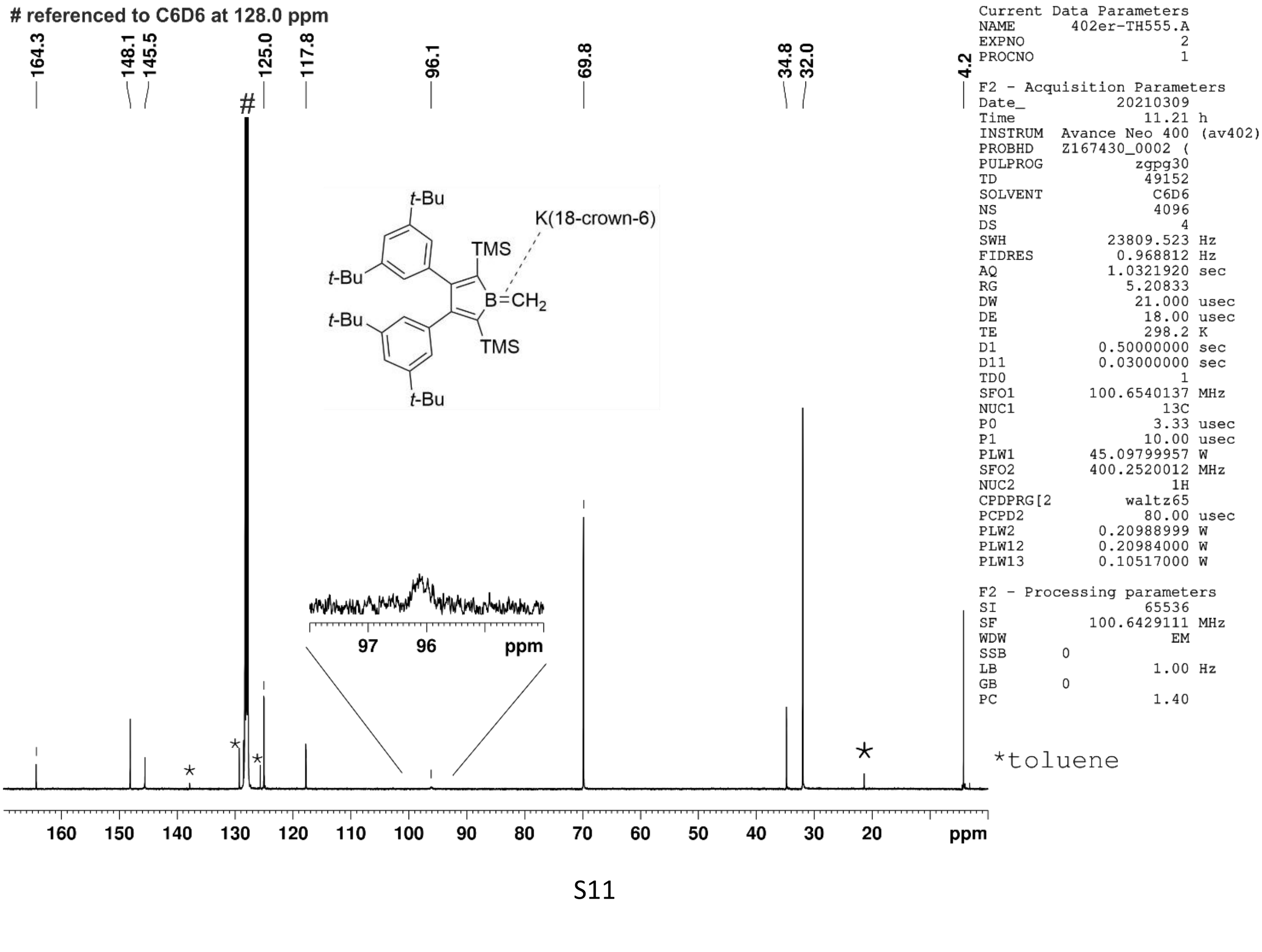

11B-NMR-(background-suppressed) spectrum of compound 2a in C6D6

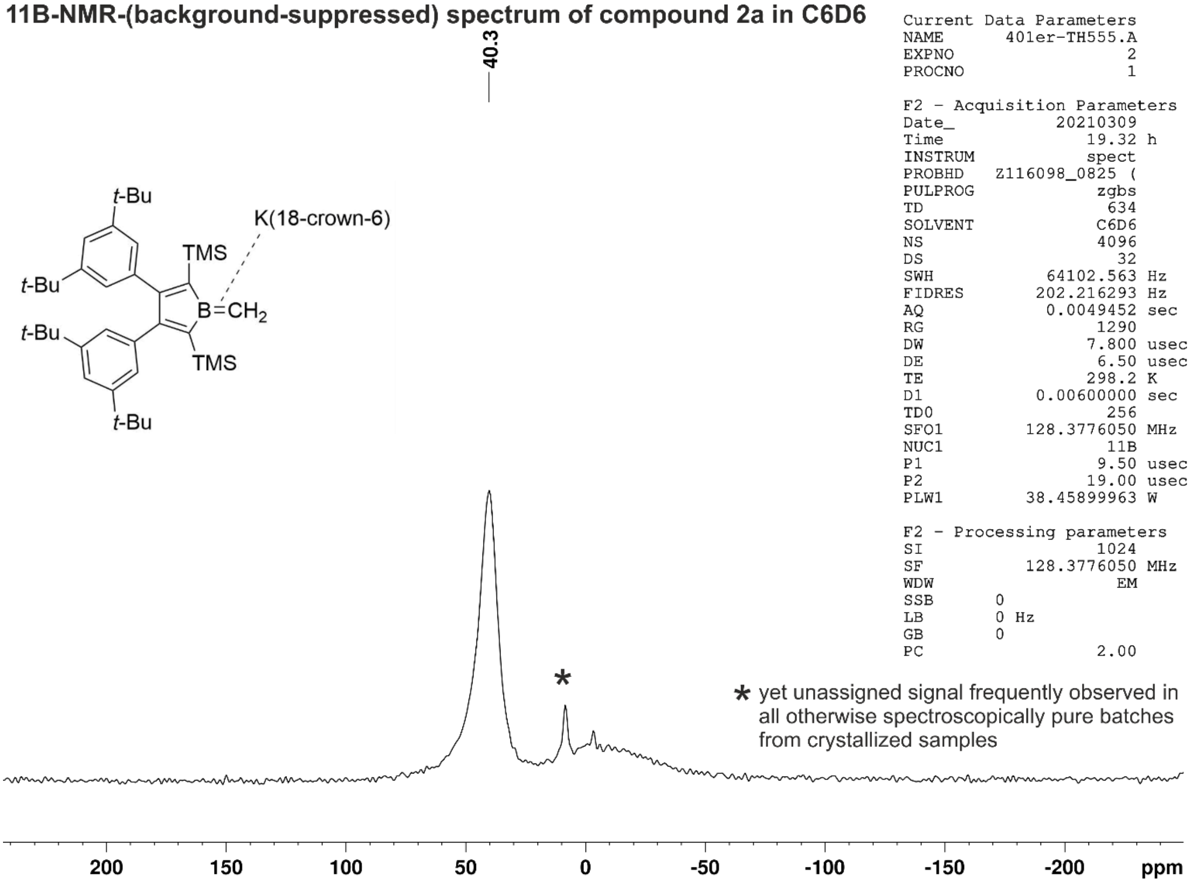

29Si-INEPT-NMR spectrum of compound 2a in C6D6

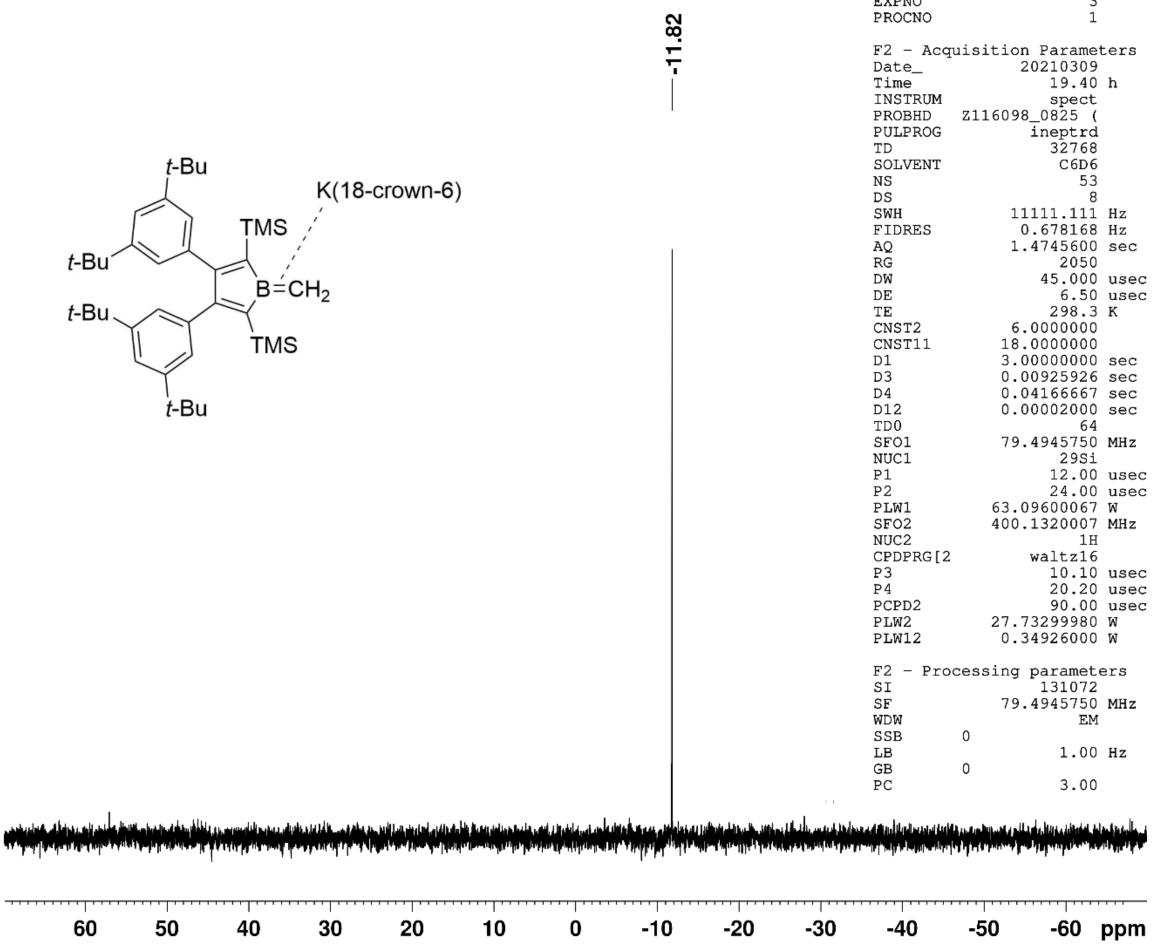

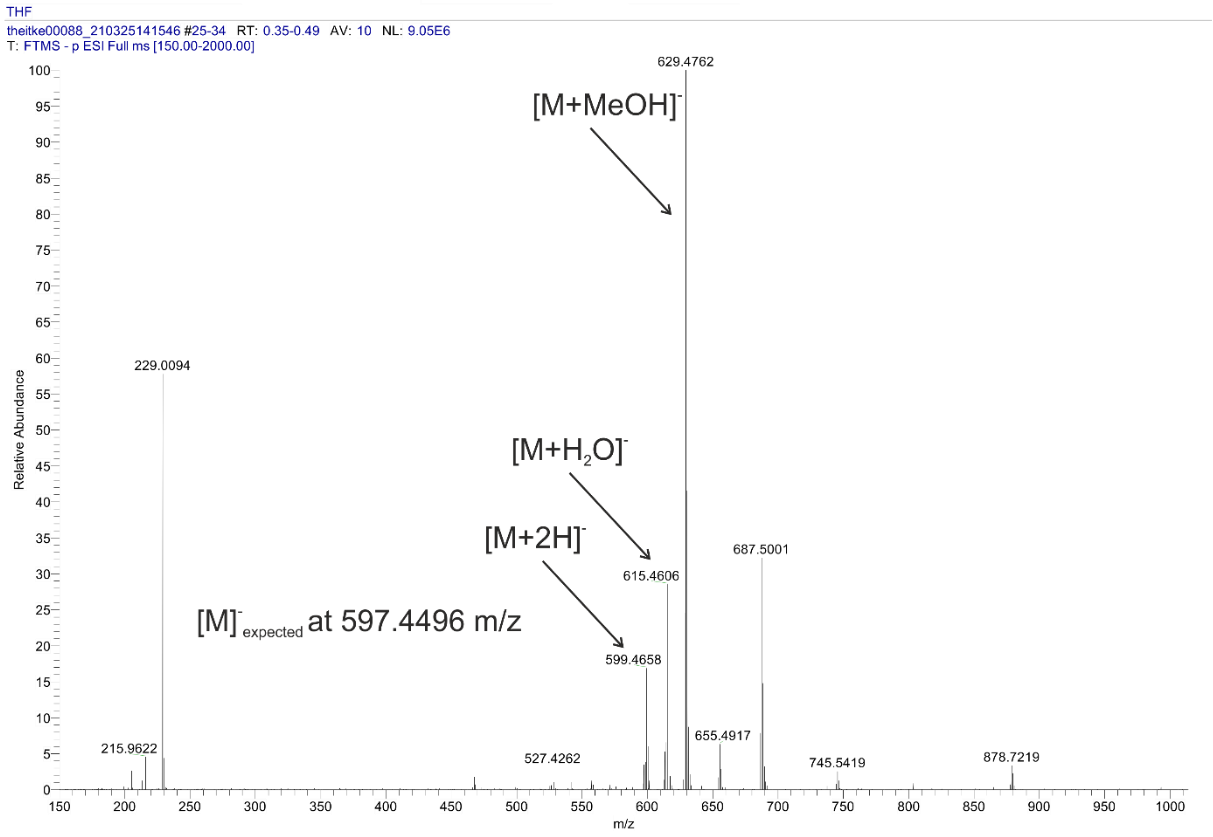
THF
theitke00088_210325141546 #25-34 RT: 0.35-0.49 AV: 10 NL: 9.05E6
T: FTMS - p ESI Full ms [150.00-2000.00]
[M+MeOH]⁻
[M+H₂O]⁻
[M+2H]⁻
[M]⁻expected at 597.4496 m/z
629.4762
229.0094
687.5001
615.4606
599.4658
655.4917
215.9622
527.4262
745.5419
878.7219
Relative Abundance
m/z

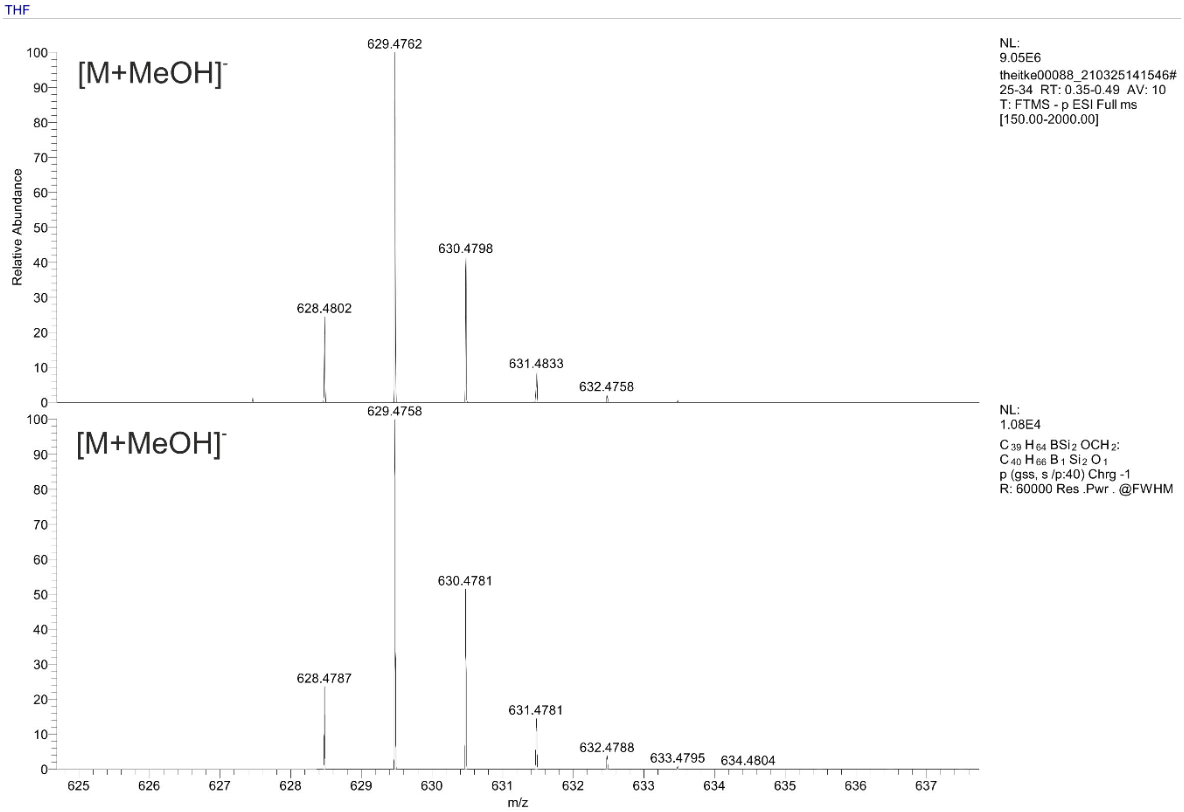
THF
[M+MeOH]⁻
629.4762
630.4798
628.4802
631.4833
632.4758
NL: 9.05E6
theitke00088_210325141546# 25-34 RT: 0.35-0.49 AV: 10
T: FTMS - p ESI Full ms [150.00-2000.00]
Relative Abundance
[M+MeOH]⁻
629.4758
630.4781
628.4787
631.4781
632.4788
633.4795
634.4804
NL: 1.08E4
C39 H64 BSi2 OCH2:
C40 H66 B1 Si2 O1
p (gss, s /p:40) Chrg -1
R: 60000 Res .Pwr . @FWHM
m/z

^{1}H-NMR Data for Compound [K(THF)$_2$]2 (2b)

^{1}H (300.13 MHz, 298 K, THF-d$_8$, solvent signal at 1.72 ppm): 6.96 (t, $^{4}J_{HH}$ = 1.9 Hz, 2H, *p-H$_{ar}$*), 6.66 (d, $^{4}J_{HH}$ = 1.9 Hz, 4H, *o-H$_{ar}$*), 3.95 (s, 2H, B-C*H$_2$*), 3.63–3.58 (m, 6H, OC*H$_2$*CH$_2$), 1.80–1.74 (m, 6H, OCH$_2$C*H$_2$*),1.12 (s, 36H, Ar-C(*Me*)$_3$), –0.07 (s, 18H, Si(*Me*)$_3$).

Preliminary crystal structure of Compound [K(THF)$_2$]2 (2b)

Crystals investigated using XRD were obtained from solutions in THF in a freezer (-40°C). Microscope examination of the crystal samples under polarized light indicated twinning. The quality of the crystals examined with XRD was very poor as the crystals diffracted weakly and scattering pattern indicated presence of at least two domains that could not be adequately separated upon data processing. Only a preliminary rather structure solution in monoclinic space group C*2* could be obtained, that however reliably reveals the connectivity pattern of the structure, yet prevents from any detailed discussion of e. g. bond lengths.

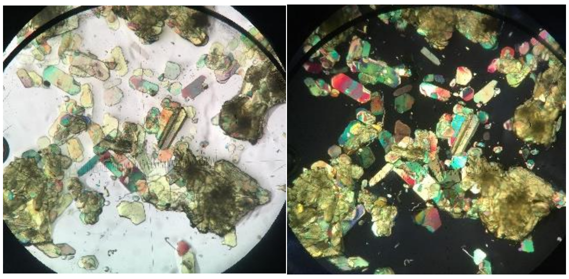

Preliminary cell and structure solution: monoclinic C*2*, a = 20.44(4) Å, b = 10.68(2) Å, c = 43.12(9) Å, α = 90°, β = 93.60(2)°, γ = 90°, V = 9393(33)Å^3; R_{Int} 8.1%, R^1 = 21.8%, wR^2 = 43.1 %

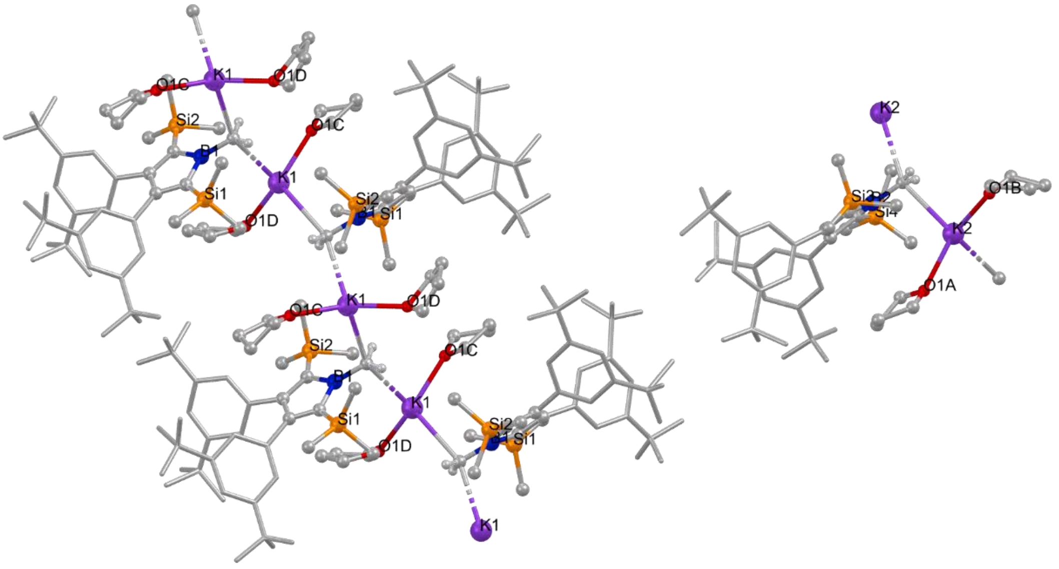

SI-Figure 3-SI Depiction of the connectivity pattern found as preliminary structure solution of [K(THF)$_2$]**2** (**2b**) with {[K(THF)$_2$]-**2**-[K(THF)$_2$] -**2**-}$_\infty$ coordination strings in the solid state.

^{1}H- NMR Data for Compound [K(18-crown-6)(THF)$_2$]2 (2c)

^{1}H (400.13 MHz, 298 K, C_6D_6, CD_5H at 7.15 ppm): 7.23 (t, $^4J_{HH}$ = 1.9 Hz, 2H, *p-H$_{ar}$*), 7.09 (d, $^4J_{HH}$ = 1.9 Hz, 4H, *o-H$_{ar}$*), 4.48 (s, 2H, B-C*H*$_2$), 3.19 (s, 24H, OC*H*$_2$CH$_2$), 1.33 (s, 36H, Ar-C(*Me*)$_3$), 0.45 (s, 18H, Si(*Me*)$_3$).

Preliminary crystal structure of Compound [K(18-crown-6)(THF)$_2$]2 (2c)

Crystals investigated using XRD were obtained from solutions in THF in a freezer (-40°C). However, only brittle aggregates of needle-type crystals were obtained. The yellow needles dispersed in per-fluorinated oil decolorized very rapidly when exposed to air and diffracted only very weakly at resolutions <0.95Å and the diffraction pattern indicates contamination with a further crystal domain which we were not able to appropriately account for during data processing. A poor data set was collected which allowed identification of the connectivity pattern.

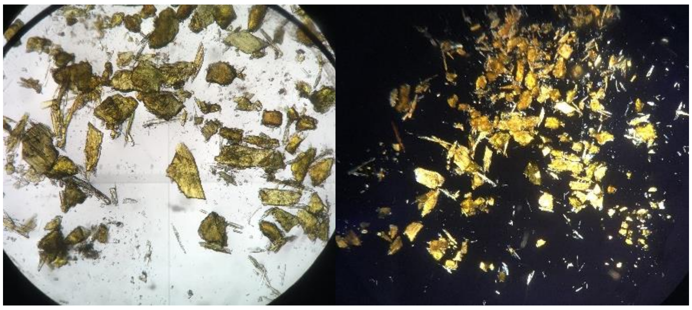

Preliminary call and structure solution: monoclinic *P2$_1$/c*, a = 11.903(3) Å, b = 41.487(8) Å, c = 39.772(8) Å, α = 90°, β = 94.405(4)°, γ = 90°, V = 19582(7)Å^3; R_{Int} 17.0%, R^1 = 14.6%, wR^2 = 30.5%

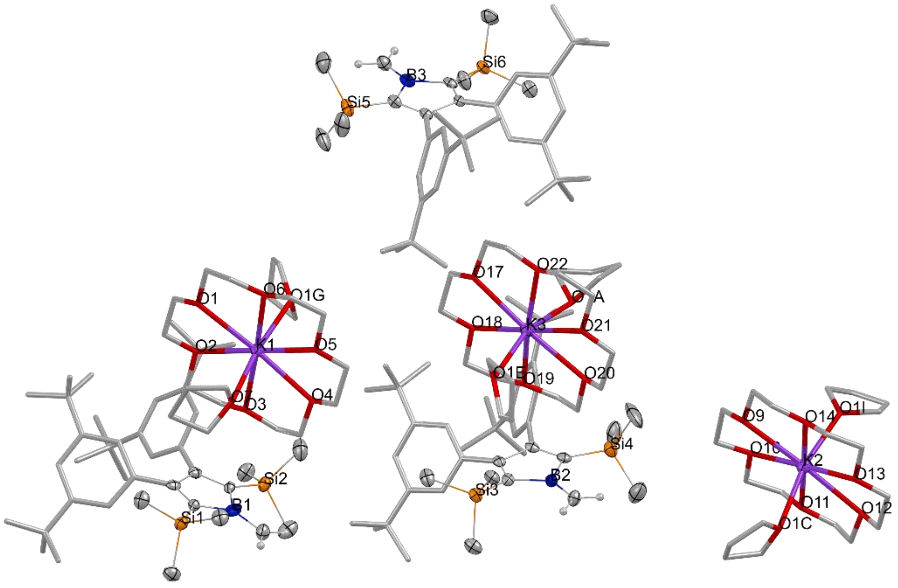

SI-Figure 4-SI Depiction of the asymmetric unit of the solid state structure including three molecules of **2c**. Lattice THF molecules and disordered parts are omitted. Despite being a preliminary structure solution, it becomes apparent that the potassium cation is coordinatively saturated by crown-ether and THF thus proving anion **2** without further contacts.

Sodium Salts of Compound 2 (2d,e)

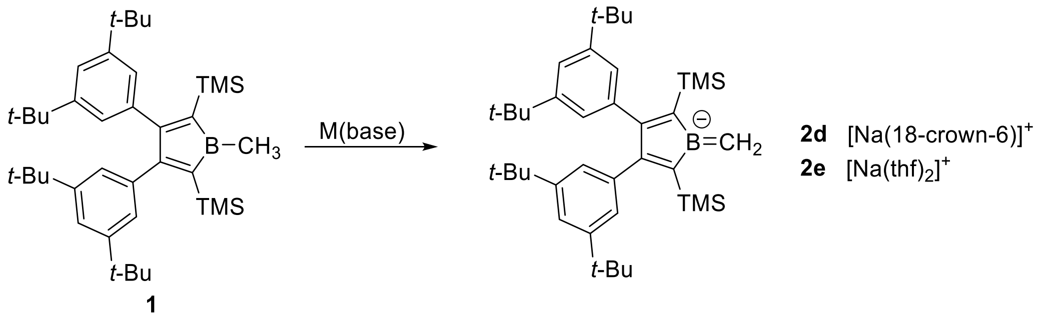

In a typical procedure: In a glovebox, a Schlenk flask was charged with methyl borole **1** (200.5 mg, 0.345 mmol, 1 eq) and NaHMDS (50.6 mg, 0.345 mmol, 1 eq) and the solids were dissolved in dry and degassed toluene (15 mL). The red solution was heated to 60 °C for three days. Over the course of the reaction the colour of the reaction mixture turned brown and a yellow solid precipitated. The solvent of the reaction mixture was removed under reduced pressure to yield a brown solid. In a glovebox, the solid was transferred from the Schlenk flask into a syringe equipped with a thin pad of glass fiber (Whatman GF/B) and washed with hexane (ca. 2 × 1 mL and 1 × 0. 5mL), until the remaining solid was of a yellow colour. The remaining yellow solid was rinsed from the filter with THF (3 mL) and the solvent was subsequently removed under reduced pressure to yield a yellow solid (103.0 mg). Analogous observations as for the potassium compounds detailed above have been made. Work-up of this crude yellow solid was then followed by either of the following options to obtain the respective solvate.

Note: When the reaction mixture was kept at room temperature, no reaction is observed after two days.

(2d): [Na(18-crown-6)]2

(**2d**) [Na(18-crown-6)]**2**: The most reliable approach was to isolate the crown ether-adduct **2d** [Na(18-crown-6)]**2**. 18-crown-6 (35.6 mg) was added and the solids were dissolved in toluene (1 mL). The solvent was removed under reduced pressure to also co-evaporate residual THF and the remaining solid was once again dissolved in a minimal amount of toluene (ca. 6 mL; note: the solubility drastically decreases after the first dissolution in toluene and subsequent toluene removal) and stored at –40 °C overnight, yielding compound **2d** as yellow needle-shaped crystals. The mother liquor was carefully decanted off and the crystals were washed with cold hexane (–40 °C, 2 × 0.3 mL). After drying the crystals under reduced pressure, compound **2d** (88.5 mg, 0.091 mmol, 27 %) was obtained as a yellow solid.

(2e): [Na(THF)$_2$]2

(**2e**) [Na(THF)$_2$]**2**: The crude solid as described above is dissolved in THF (ca. 1 mL/15 mg solid) and the yellow solutions are stored at –40°C over the course of several days. [Na(THF)$_2$]**2** can be obtained as pale yellow flat plate-shaped crystals (ca. 40% in mass with regards to the dissolved crude solid). X-ray diffraction studies on these crystals repeatedly gave only mediocre data sets. Yet, the connectivity pattern could unambiguously be identified from the structure (see below).

Analytical Data for Compound [Na(18-crown-6)]2 (2d)

NMR:

^{1}H (400.13 MHz, 298 K, C_6D_6, CD_5H at 7.15 ppm): 7.24 (t, $^{4}J_{HH}$ = 1.9 Hz, 2H, *p-H$_{ar}$*), 7.07 (d, $^{4}J_{HH}$ = 1.9 Hz, 4H, *o-H$_{ar}$*), 4.18 (s, 2H, B-C*H$_2$*), 3.22 (s, 24H, OC*H$_2$*CH$_2$), 1.33 (s, 36H, Ar-C(*Me*)$_3$), 0.46 (s, 18H, Si(*Me*)$_3$).

^{13}C{^{1}H} (100.65 MHz, 298 K, C_6D_6, solvent signal at 128.0 ppm): 165.0 (borole-*C$_{3,4}$*), 148.2 (*m-C$_{ar}$*), 145.9 (borole-*C$_{2,5}$*), 145.4 (*ipso-C$_{ar}$*), 124.9 (*o-C$_{ar}$*), 117.9 (*p-C$_{ar}$*), 89.2 (B-*C*H$_2$, only observed in the HMBC-spectrum), 68.8 (O*C*H$_2$CH$_2$), 34.8 (Ar-*C*(CH$_3$)$_3$), 31.9 (Ar-C(*C*H$_3$)$_3$), 4.0 (Si(*C*H$_3$)$_3$).

^{11}B (128.38 MHz, 298 K, C_6D_6): 42.2.

^{29}Si-INEPT (79.49 MHz, 298 K, C_6D_6): –11.6.

Elemental Analysis: (**2d**): $C_{51}H_{86}BNaO_6Si_2$ calcd C 69.20, H 9.79; (**2d × 1 toluene**): $C_{58}H_{94}BNaO_6Si_2$ calcd C 71.28, H 9.69 observed C 69.25, H 9.72. *Note*: sample submitted to EA still contained residual toluene according to NMR.

Crystal structure of Compound [Na(18-crown-6)]2 (2d)

For further details on the diffraction measurement please see the respective section. Crystals investigated using XRD were obtained from solutions in toluene in a freezer (-40°C).

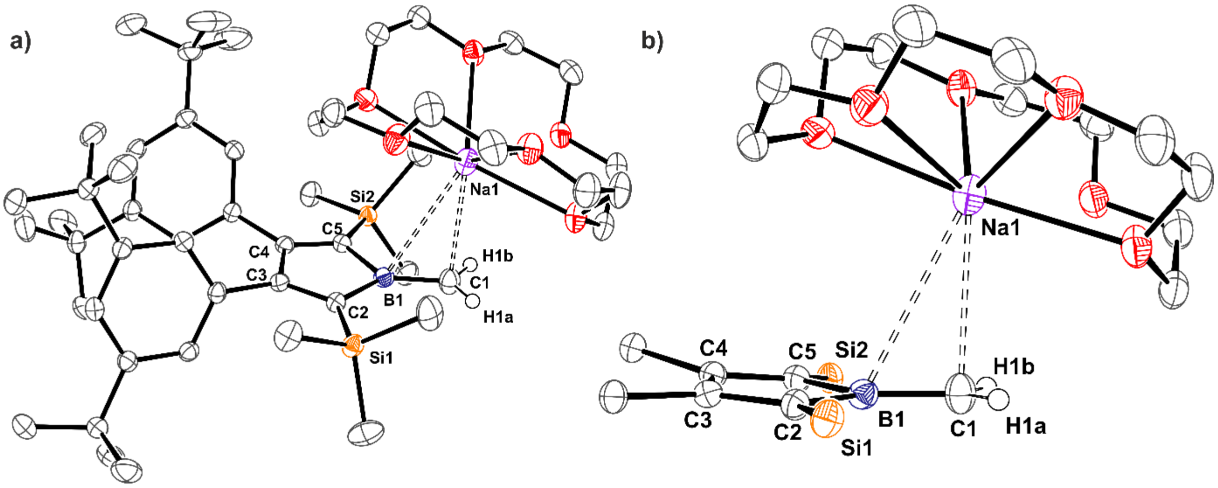

SI-Figure 5-SI ORTEP of the molecular structure of boratafulvene **2**[Na(18-crown-6)] (**2d**). ADP are drawn at 50% probability. a) Full depiction of the molecular structure. Non-methylene hydrogen atoms, disorder in *t*Bu groups and lattice toluene are omitted for clarity. Selected bond lengths [Å]: B1–C1 1.447(3), B1–C2 1.605(2), C2–C3 1.367(2)m, C3–C4 1.502(2), C4–C5 1.364(2), C5–B1 1.608(2), C1–Na1 2.953(2), Na1–B1 3.343(2). b) Detail of the immediate coordination environment of boratafulvene and sodium atom.

Spectra Plots for Compound [Na(18-crown-6)]2 (2d)

1H-NMR spectrum of compound 2d in C6D6

referenced to C6D5H at 7.15 ppm

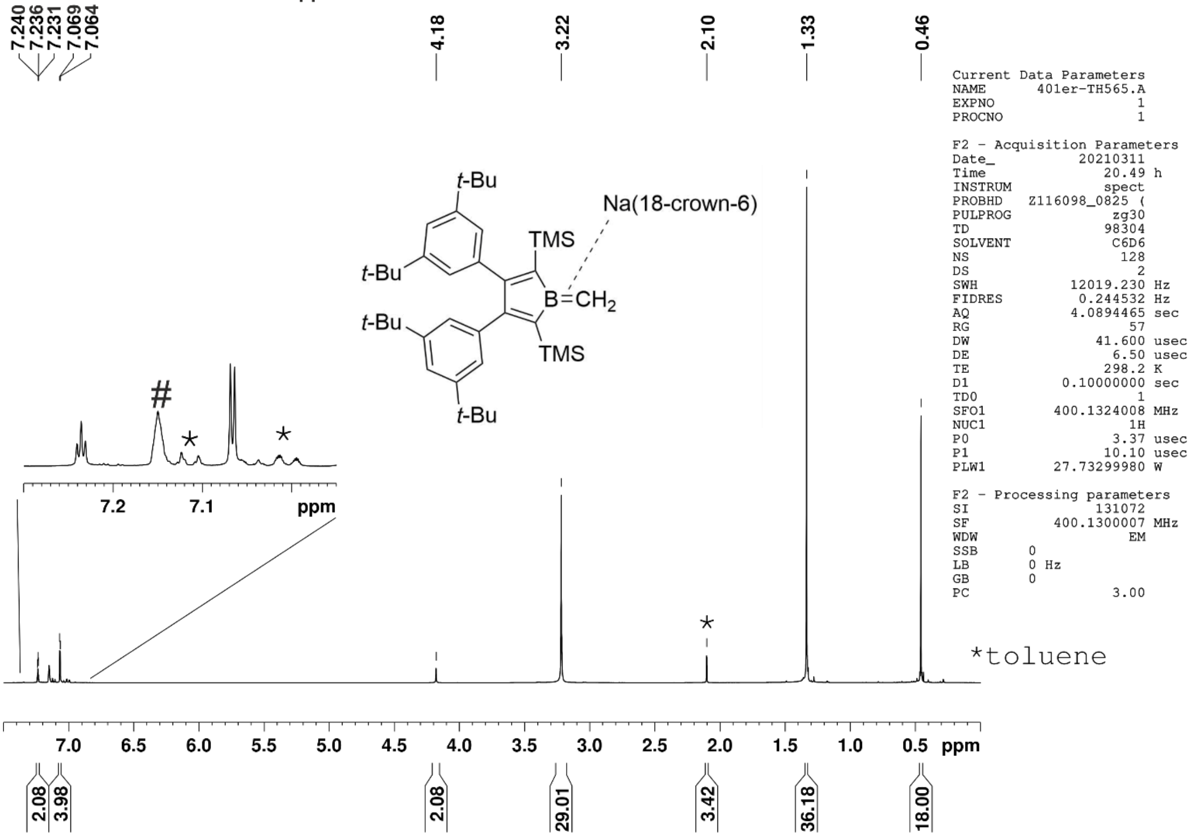

13C{1H}-NMR spectrum of compound 2d in C6D6

referenced to C6D6 at 128.0 ppm

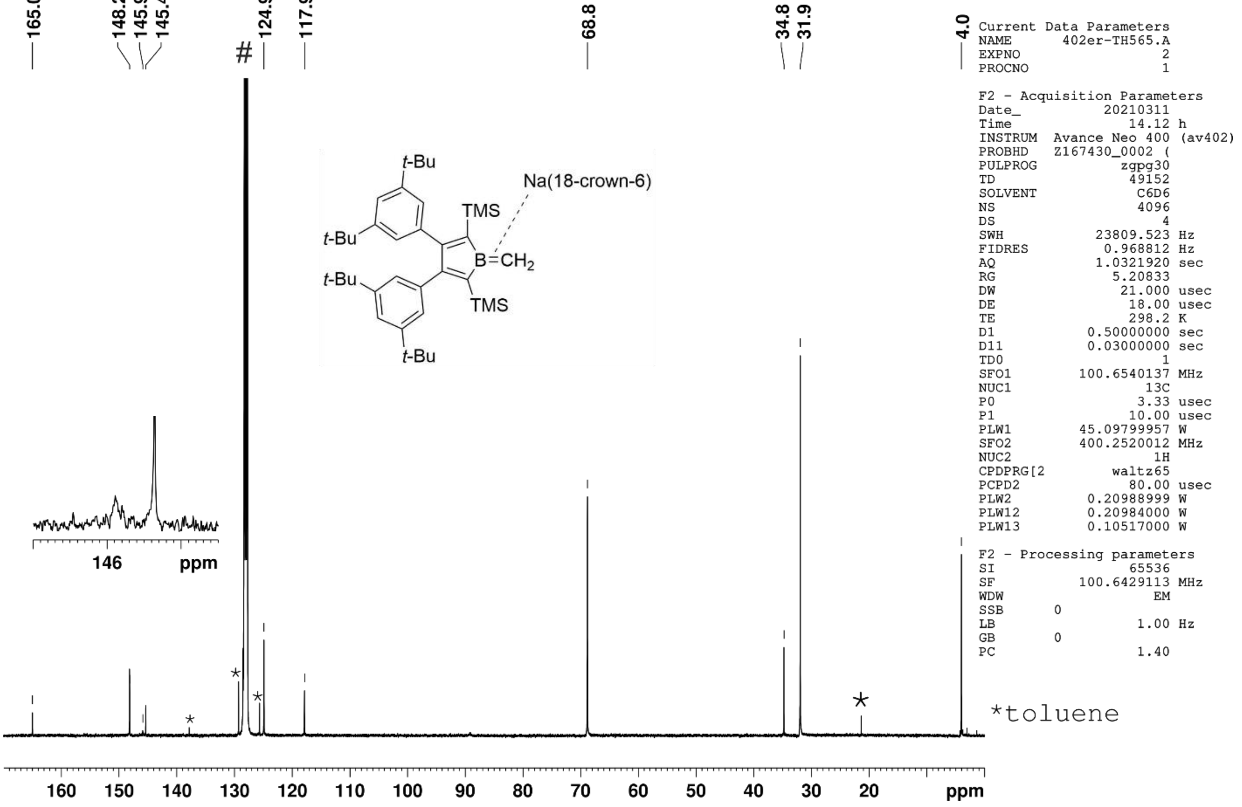

11B-NMR-(background-suppressed) spectrum of compound 2d in C6D6

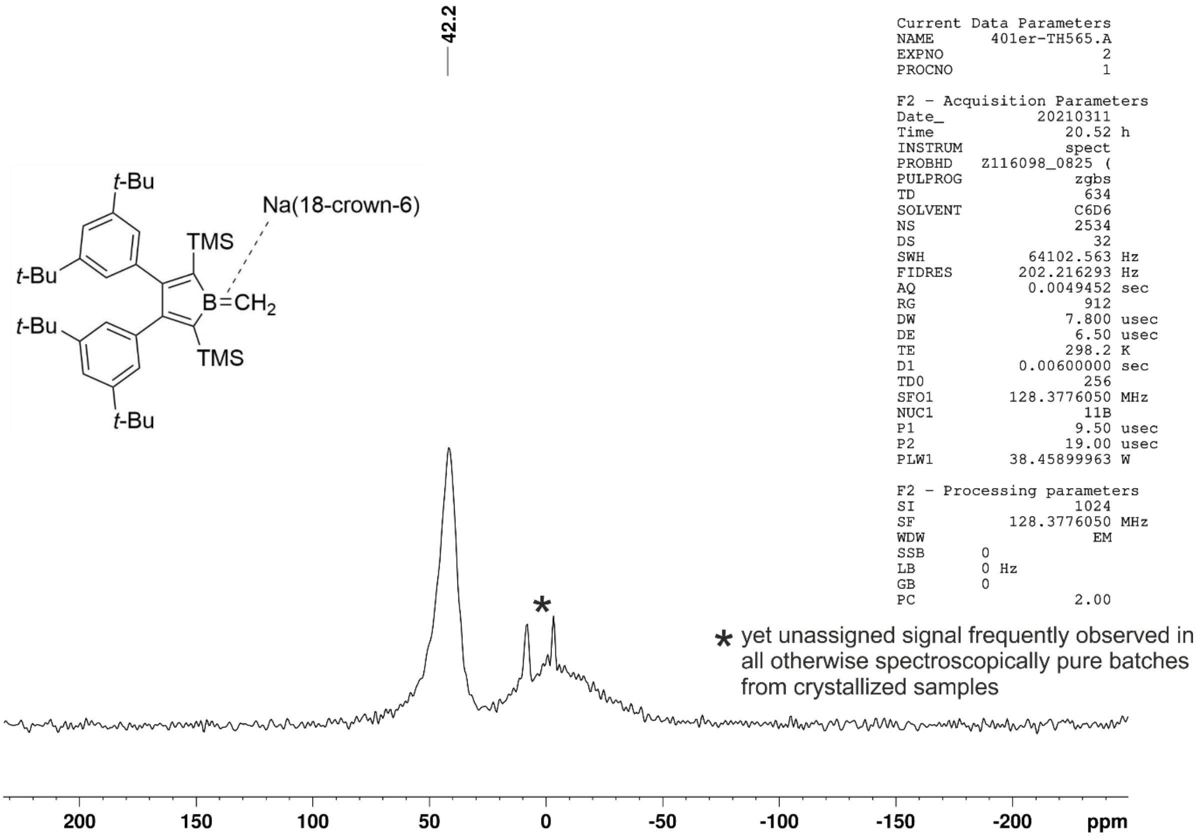

29Si-INEPT-NMR spectrum of compound 2d in C6D6

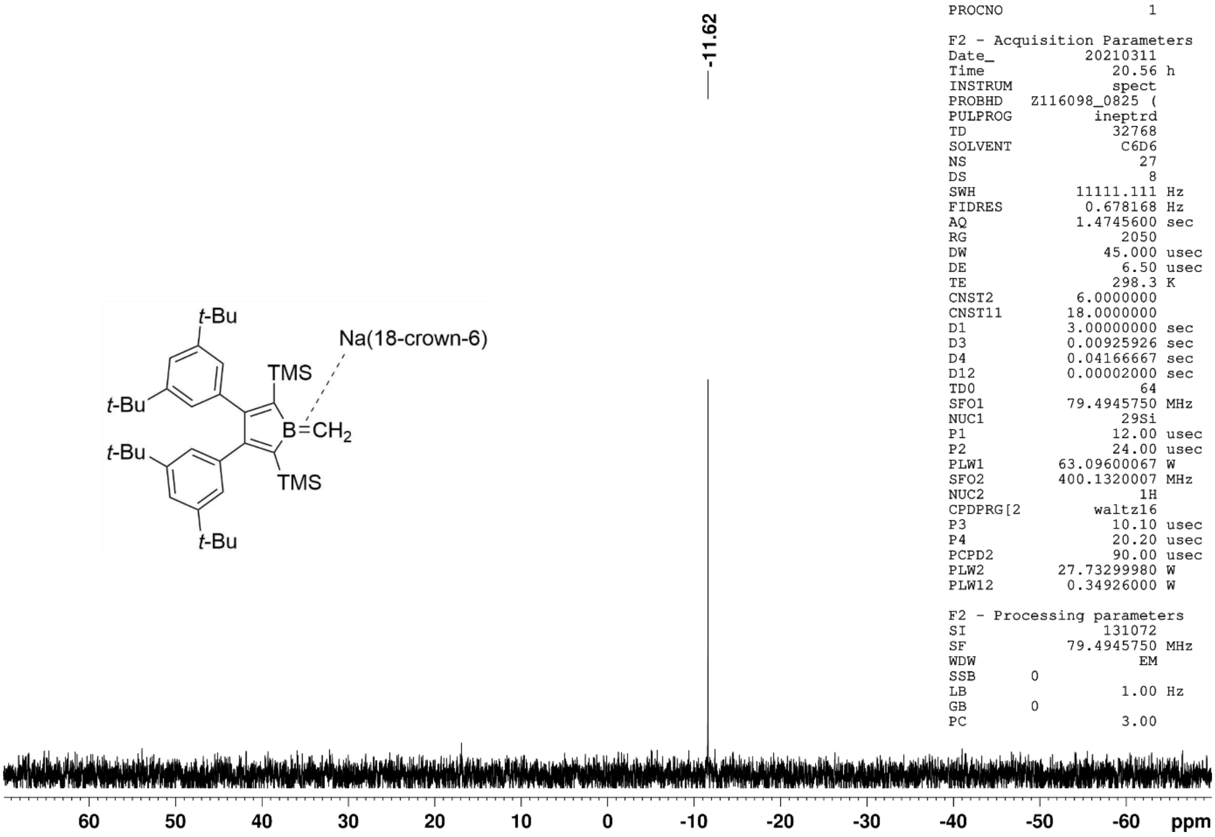

NMR Data for Compound [Na(THF)$_2$]2 (2e)

NMR:

^{1}H (500.25 MHz, 298 K, THF-d$_8$, solvent signal at 1.72 ppm): 6.97 (t, $^4J_{HH}$ = 1.9 Hz, 2H, *p-H$_{ar}$*), 6.65 (d, $^4J_{HH}$ = 1.9 Hz, 4H, *o-H$_{ar}$*), 3.71 (s, 2H, B-C*H*$_2$), 3.62–3.59 (m, 8H, OC*H*$_2$CH$_2$), 1.78–1.75 (m, 8H, OCH$_2$C*H*$_2$), 1.12 (s, 36H, Ar-C(*Me*)$_3$), –0.09 (s, 18H, Si(*Me*)$_3$).

^{13}C{^{1}H} (100.65 MHz, 298 K, THF-d$_8$, solvent signal at 25.31 ppm): 165.5 (borole-*C$_{3,4}$*), 148.5 (*m-C$_{ar}$*), 145.2 (*ipso-C$_{ar}$*), 144.4 (borole-*C$_{2,5}$*), 125.0 (*o-C$_{ar}$*), 118.4 (*p-C$_{ar}$*), 88.1 (B-*C*H$_2$), 68.2 (O*C*H$_2$CH$_2$), 35.1 (Ar-*C*(CH$_3$)$_3$), 32.0 (Ar-C(*C*H$_3$)$_3$), 26.4 (OCH$_2$*C*H$_2$), 3.6 (Si(*C*H$_3$)$_3$).

^{11}B (160.50 MHz, 298 K, THF-d$_8$): 40.2.

^{29}Si-INEPT (79.49 MHz, 298 K, THF-d$_8$): –11.9.

Preliminary Crystal structure of Compound [Na(THF)$_2$]2 (2e)

Crystals investigated using XRD were obtained from solutions in THF in a freezer (-40°C). The quality of the fine colourless needle crystals examined with XRD was very poor as the crystals diffracted very weakly at resolutions <0.96Å even with very long exposure times. Only a rather preliminary structure solution in monoclinic space group C*2/c* could be obtained, that however reliably reveals the connectivity pattern of the structure, yet prevents from any detailed discussion of e. g. bond lengths. Sodium atom occupies a special position. The structure was refined as a non-merohedral twin using the twin law -1 0 0 0 -1 0 0.2 0 1 with the fraction of the minor component refining to 0.44.

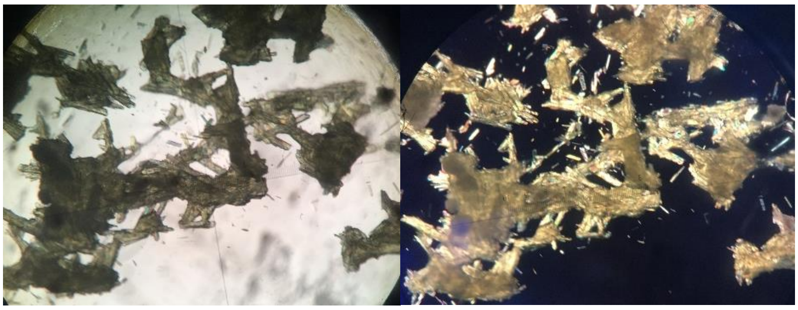

Preliminary cell and structure solution: monoclinic C*2/c*, a = 10.640(7)Å, b = 20.991(13) Å, c = 43.12(3) Å, α = 90°, β = 91.390(10)°, γ = 90°, V = 9627(10)Å^3; R_{Int} 11.1%, R^1 = 14.8%, wR^2 = 38.1 %

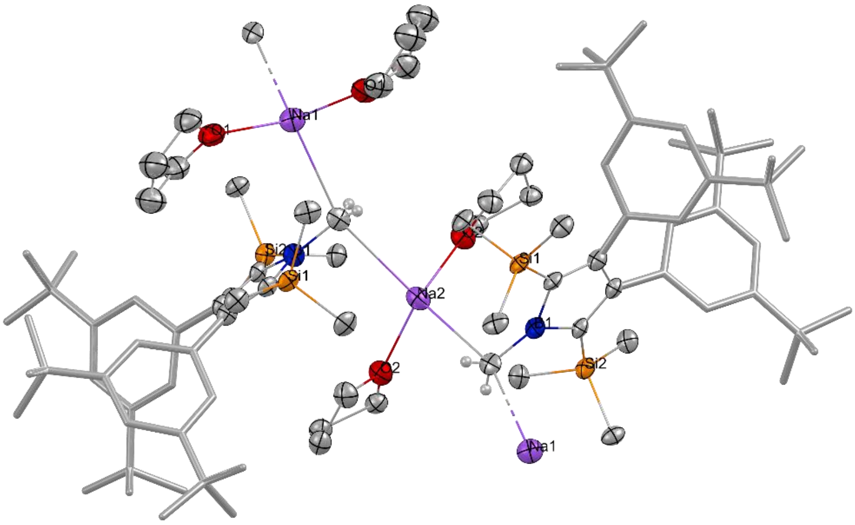

SI-Figure 6-SI Depiction of the connectivity pattern found as preliminary structure solution of [Na(THF)$_2$]**2** (**2e**) with {[Na(THF)$_2$]-**2**-[Na(THF)$_2$] -**2**-}$_\infty$ coordination strings in the solid state.

Spectra Plots for Compound [Na(THF)$_2$]2 (2e)

1H-NMR spectrum of compound 2e in THF-d8
\# referenced to THF-d7 at 1.72 ppm

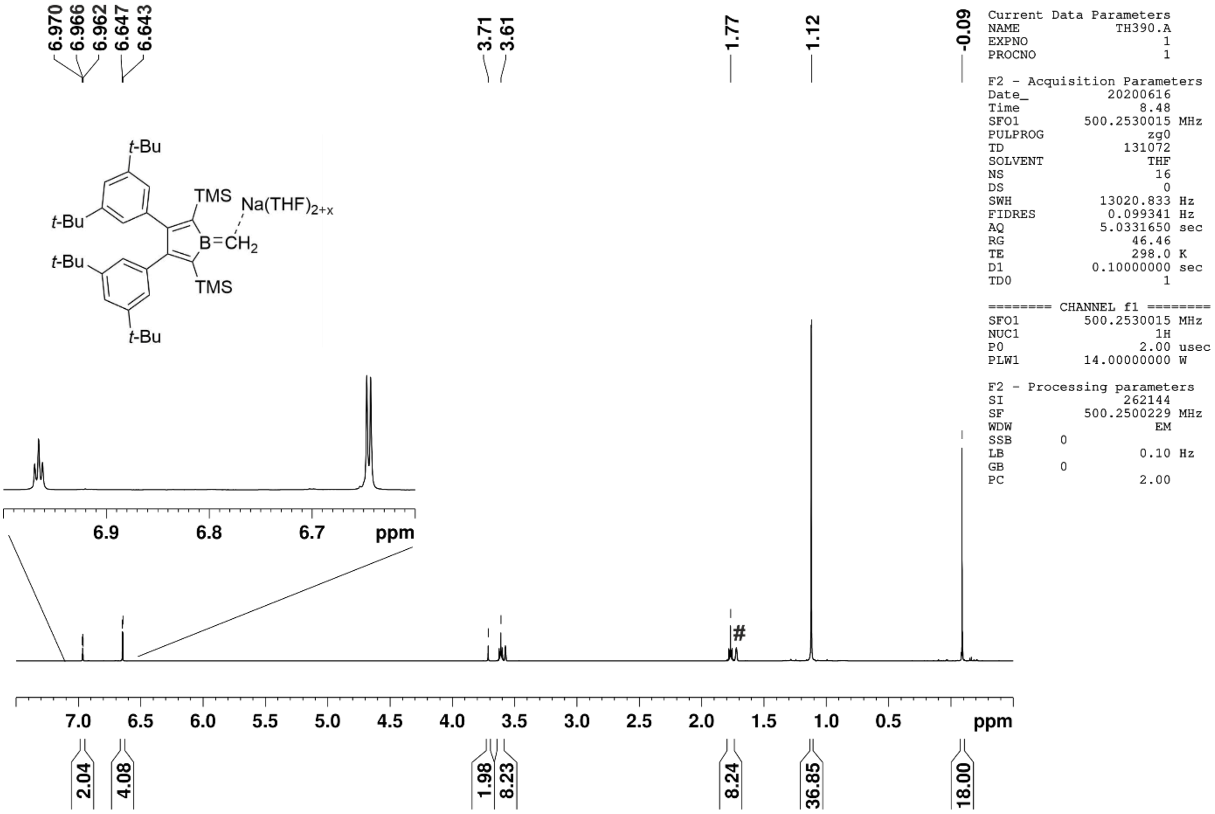

13C{1H}-NMR spectrum of compound 2e in THF-d8
\# referenced to THF-d8 at 25.3 ppm

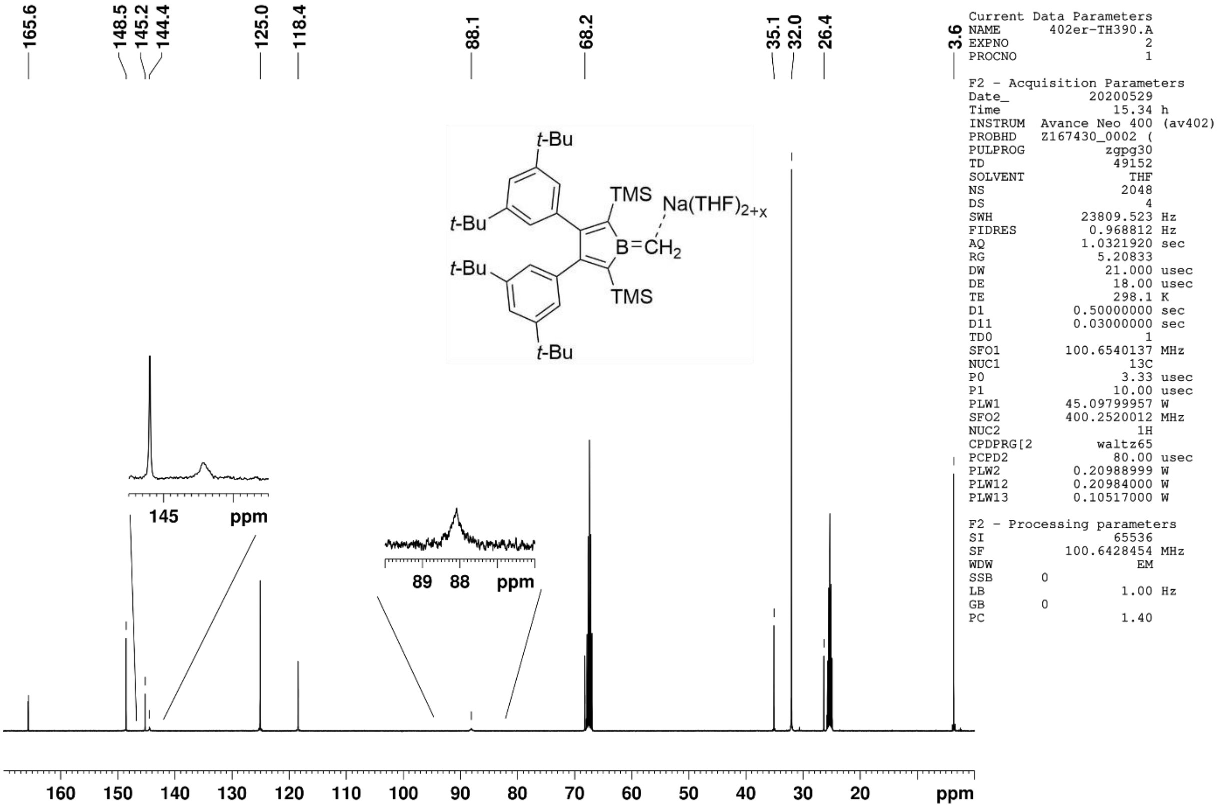

11B-NMR-(background-suppressed) spectrum of compound 2e in THF-d8

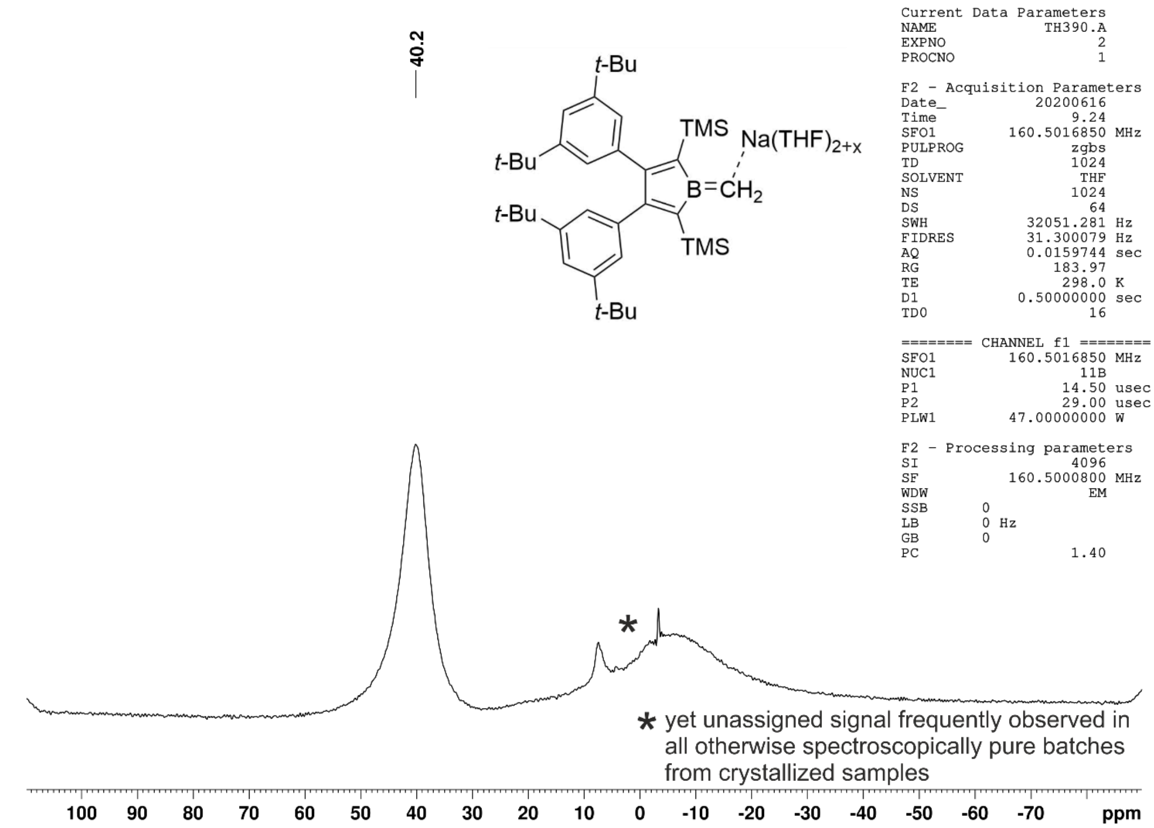

29Si-INEPT-NMR spectrum of compound 2e in THF-d8

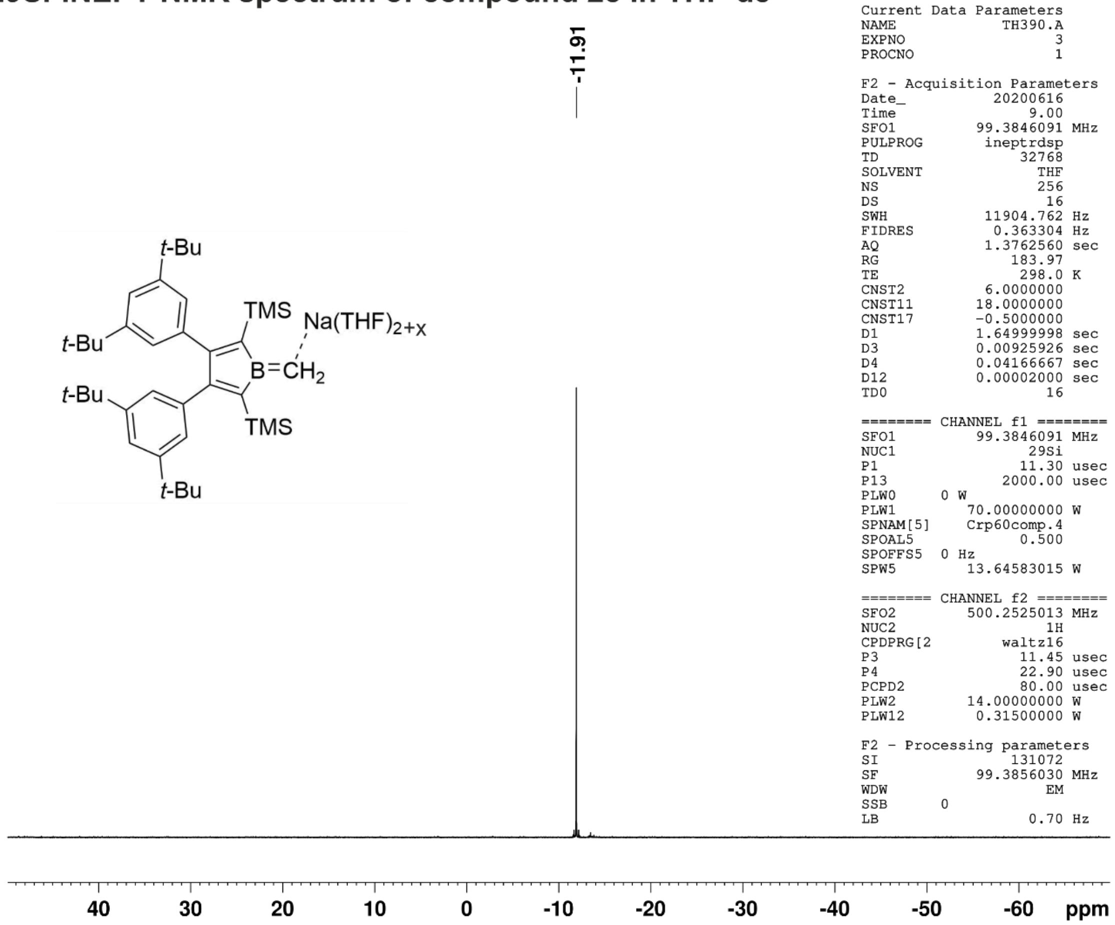

Side Product B [1(HMDS)]K(18-crown-6)]

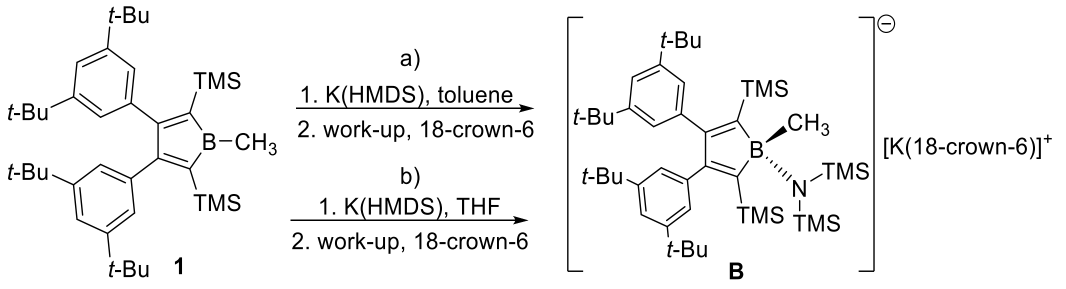

Molecular Weight: 598,91

Molecular Weight: 759,30

a) As described for the procedures of the deprotonation reactions of **1** with M(HMDS) (M = Na, K) in toluene detailed above, the crude reaction mixtures, after initial, work-up contained further side products in varying amounts. Key feature of **B** is its insolubility in hexane which allows removal of **2** and other side-products, that reveal poor but sufficient solubility in hexane, by extraction with ample amounts of hexane until the residual solids remain colourless. Addition of crown-ether (18-crown-6) to THF solutions of this colourless solid allowed isolation of small crops of colourless crystals which were identified as [K(18-crown-6)]**B**, the amide adduct to **1**, as one of the undesired signature side-products of the deprotonation reaction.

b) In THF, formation of adduct **B** is preferred over the deprotonation. Therefore, **B** can be accessed more conveniently from THF reactions of **1** and K(HMDS) after removal of all volatiles and extensive washing of the resulting crude solid with hexane until a colourless solid is obtained. NMR examination of this solid in THF-d_8 reveals to be spectroscopically pure $[K(THF)_x]^+$ salt of amide adduct anion **B.**

Note: Key feature for the removal of **B** from other compounds involved in the reaction mixtures is its insolubility in hexane.

Analytical Data for Compound [K(18-crown-6)]B

NMR:

^{1}H (400.13 MHz, 298 K, THF-d_8, solvent signal at 1.72 ppm): 6.83 (t, $^{4}J_{HH}$ = 1.9 Hz, 2H, *p-H$_{ar}$*), 6.81–6.75 (br, 2H, *o-H$_{ar}$*), 6.66–6.59 (br, 2H, *o-H$_{ar}$*), 1.11 (s, 36H, Ar-C(*Me*)$_3$), 0.21 (s, 9H, NSi(*Me*)$_3$), 0.14 (s, 9H, NSi(*Me*)$_3$), 0.05 (s, 3H, BC*H*$_3$), –0.16 (s, 18H, Si(*Me*)$_3$).

^{13}C{^{1}H} (100.65 MHz, 298 K, THF-d_8, solvent signal at 25.3 ppm): 169.4 (br, borole-*C$_{2,5}$*), 156.9 (borole-*C$_{3,4}$*), 146.6 (*m-C$_{ar}$*), 146.5 (*ipso-C$_{ar}$*), 124.8 (br, *o-C$_{ar}$*), 124.2 (br, *o-C$_{ar}$*), 116.2 (*p-C$_{ar}$*), 34.0 (Ar-*C*(CH$_3$)$_3$), 31.2 (Ar-C(*C*H$_3$)$_3$), 14.8 (B*C*H$_3$), 8.3 (N(Si(*C*H$_3$)$_3$)$_2$), 6.6 (N(Si(*C*H$_3$)$_3$)$_2$), 2.9 (Si(*C*H$_3$)$_3$).

^{11}B (128.38 MHz, 298 K, THF-d_8): 4.1.

^{29}Si-INEPT (79.49 MHz, 298 K, THF-d_8): –3.8 (s), –6.6 (s), –13.9 (s).

Crystal structure of Compound [K(18-crown-6)]B

For further details on the diffraction measurement please see the respective section. Crystals investigated using XRD were obtained from solutions in toluene in a freezer (-40°C).

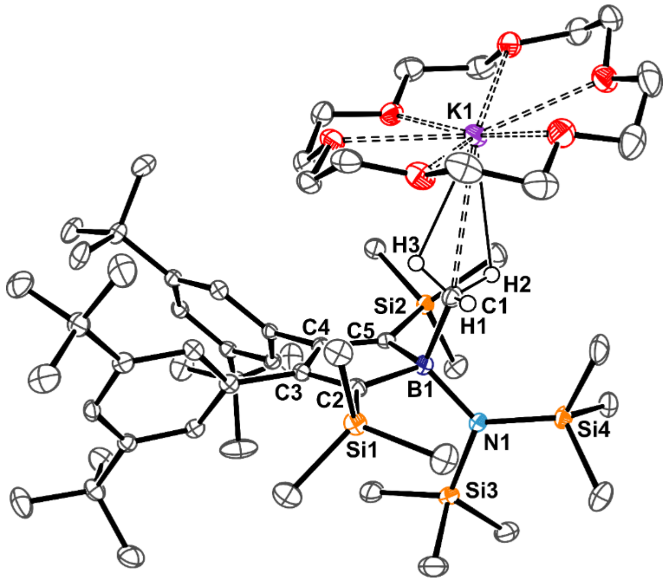

SI-Figure 7-SI ORTEP of the molecular structure of HMDS amide adduct to methylborole **1** [K(18-crown-6)]**B**. ADP are drawn at 50% probability. Full depiction of the molecular structure. Non-B-methyl hydrogen atoms and disorder in a *t*Bu group omitted for clarity. Selected bond lengths [Å]: B1–N1 1.611(2), B1–C1 1.647(3), B1–C2 1.659(3), C2–C3 1.360(2), C3–C4 1.504(3), C4–C5 1.365(3), C5–B1 1.661(2), C1–K1 3.218(2), H3–K1 2.78(2), H2–K1 2.85(2), H1–K1 3.39(2).

Spectra Plots for Side Product B [1(HMDS)]K(18-crown-6)]

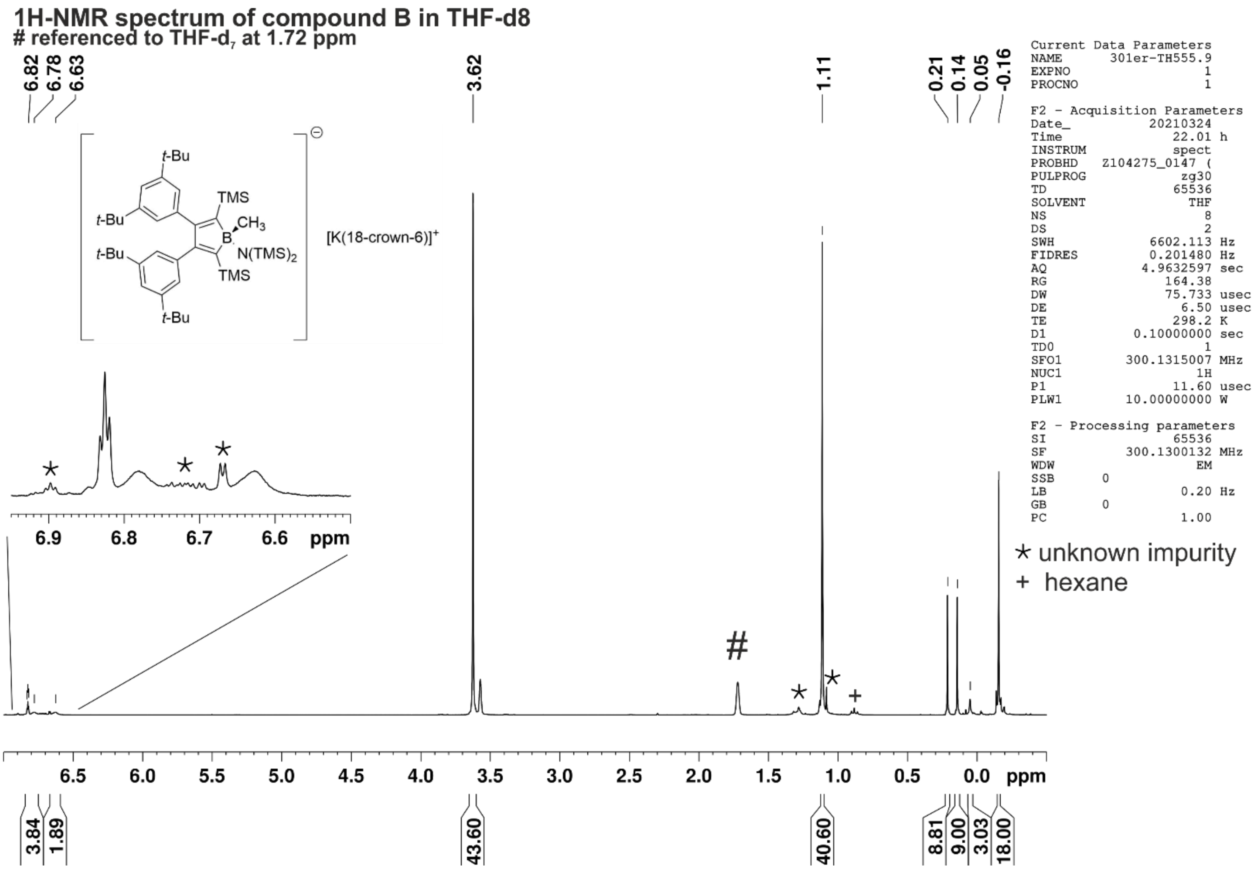

13C-NMR spectrum of compound B in THF-d8

# referenced to THF-d8 at 25.3 ppm

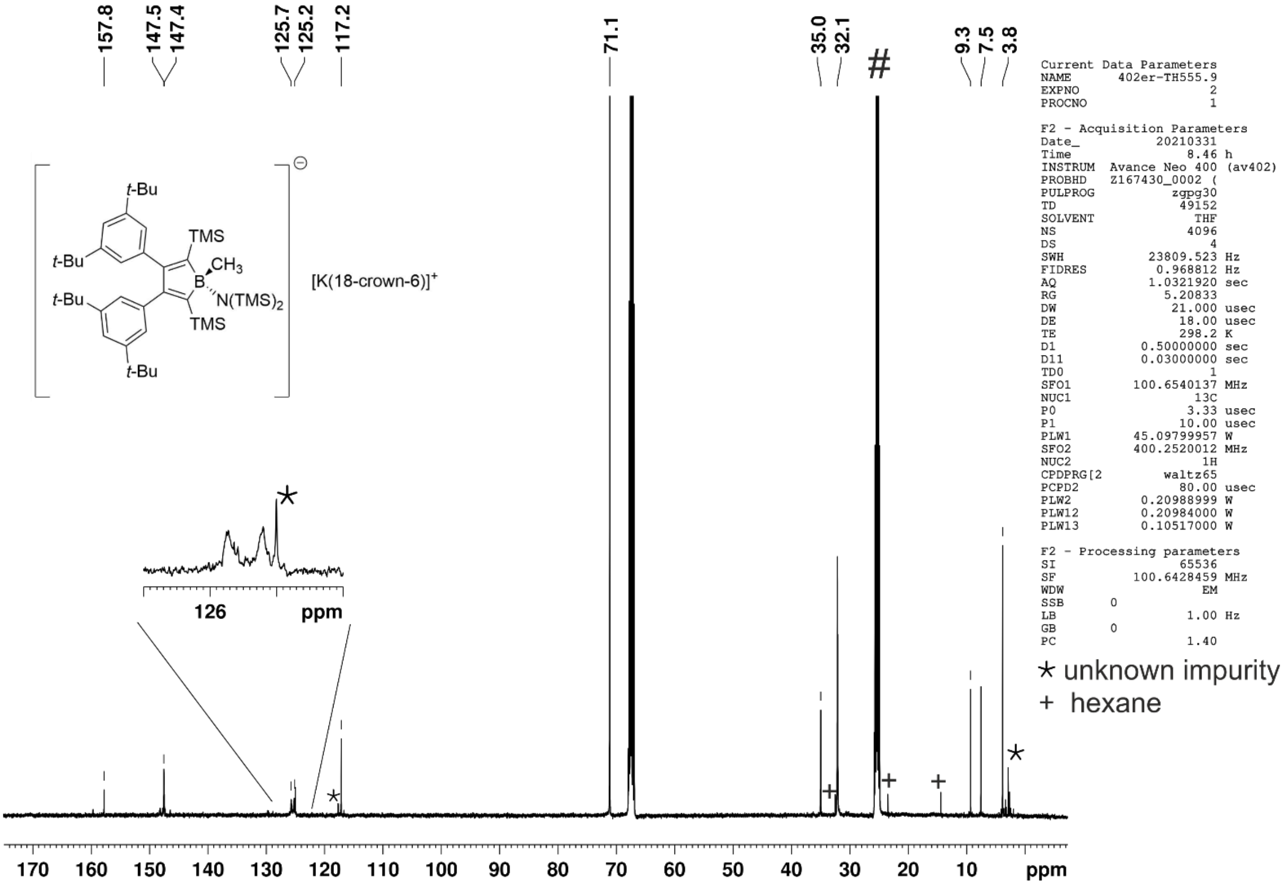

11B-NMR spectrum (background suppressed) of compound B in THF-d8

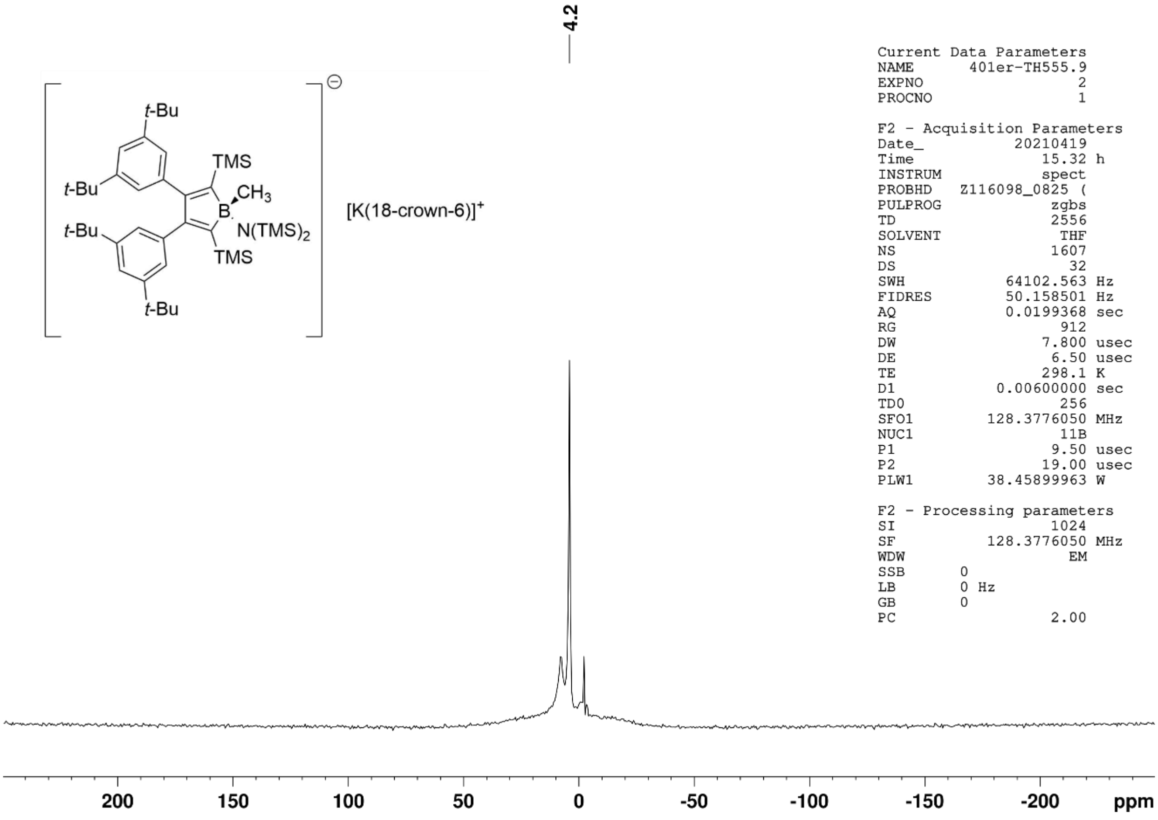

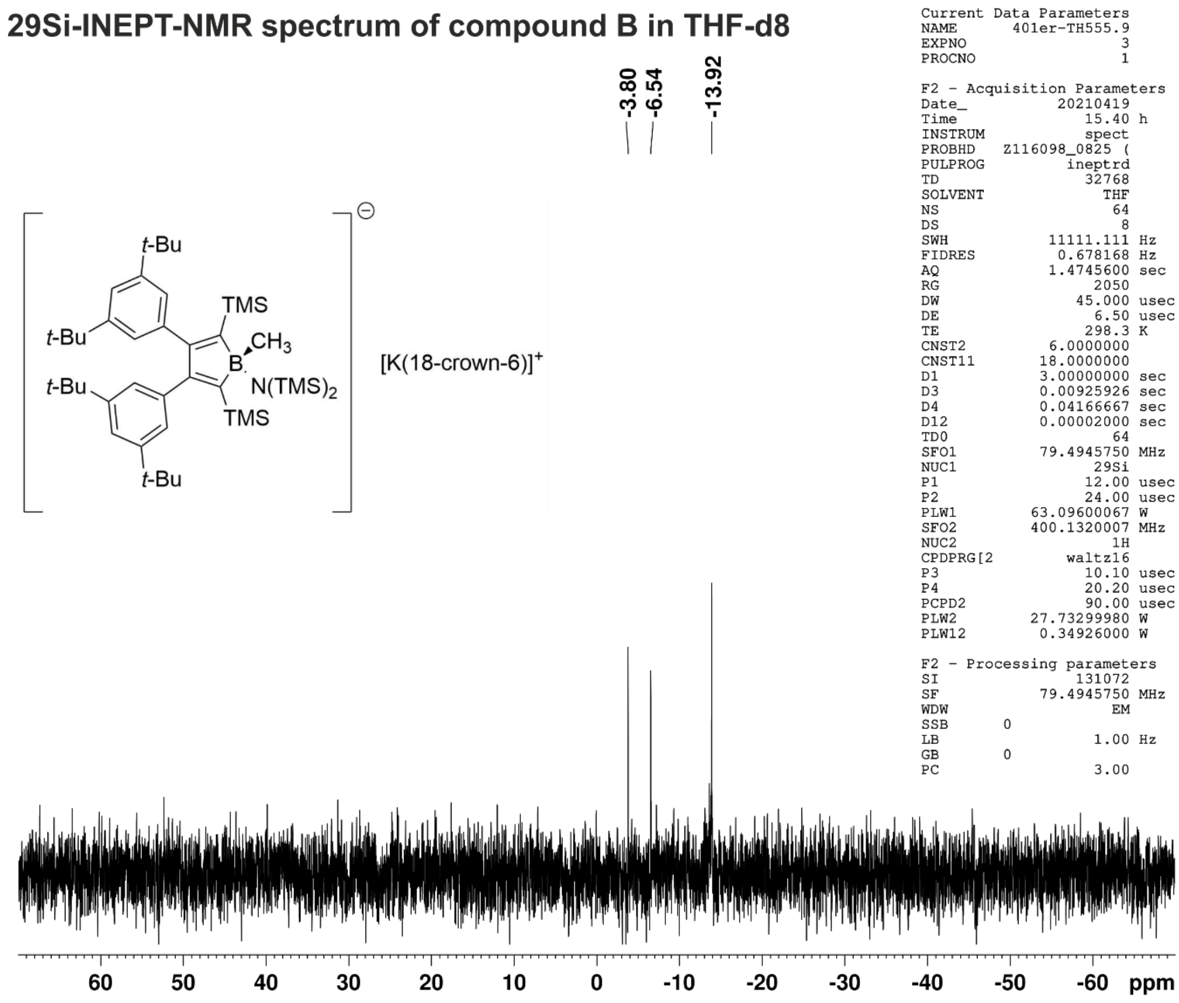

29Si-INEPT-NMR spectrum of compound B in THF-d8
-3.80
-6.54
-13.92
t-Bu
TMS
CH3
B
N(TMS)2
[K(18-crown-6)]+
Current Data Parameters
NAME 401er-TH555.9
EXPNO 3
PROCNO 1
F2 - Acquisition Parameters
Date_ 20210419
Time 15.40 h
INSTRUM spect
PROBHD Z116098_0825 (
PULPROG ineptrd
TD 32768
SOLVENT THF
NS 64
DS 8
SWH 11111.111 Hz
FIDRES 0.678168 Hz
AQ 1.4745600 sec
RG 2050
DW 45.000 usec
DE 6.50 usec
TE 298.3 K
CNST2 6.0000000
CNST11 18.0000000
D1 3.00000000 sec
D3 0.00925926 sec
D4 0.04166667 sec
D12 0.00002000 sec
TD0 64
SFO1 79.4945750 MHz
NUC1 29Si
P1 12.00 usec
P2 24.00 usec
PLW1 63.09600067 W
SFO2 400.1320007 MHz
NUC2 1H
CPDPRG[2 waltz16
P3 10.10 usec
P4 20.20 usec
PCPD2 90.00 usec
PLW2 27.73299980 W
PLW12 0.34926000 W
F2 - Processing parameters
SI 131072
SF 79.4945750 MHz
WDW EM
SSB 0
LB 1.00 Hz
GB 0
PC 3.00
60 50 40 30 20 10 0 -10 -20 -30 -40 -50 -60 ppm

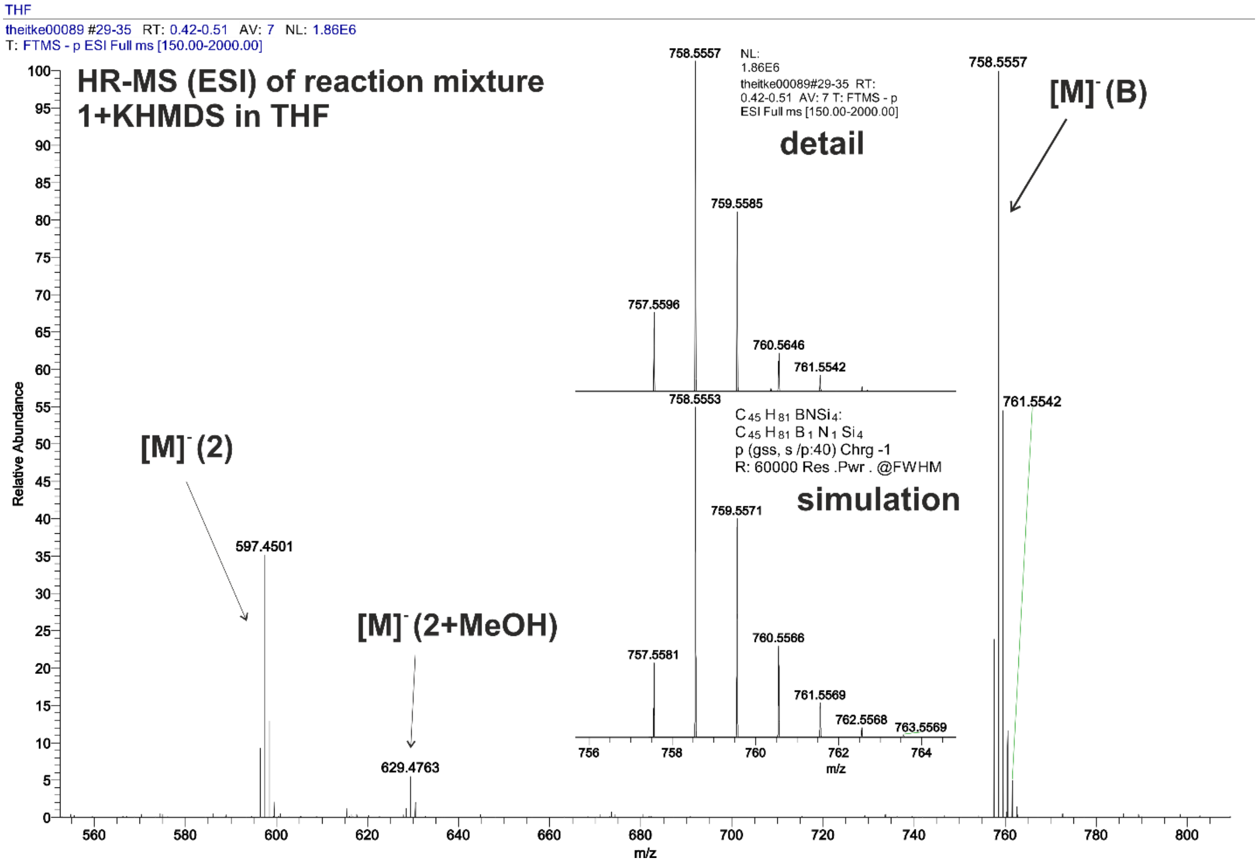

THF
theitke00089 #29-35 RT: 0.42-0.51 AV: 7 NL: 1.86E6
T: FTMS - p ESI Full ms [150.00-2000.00]
HR-MS (ESI) of reaction mixture
1+KHMDS in THF
[M]- (B)
[M]- (2)
[M]- (2+MeOH)
597.4501
629.4763
758.5557
761.5542
detail
NL: 1.86E6
theitke00089#29-35 RT: 0.42-0.51 AV: 7 T: FTMS - p ESI Full ms [150.00-2000.00]
757.5596
759.5585
760.5646
simulation
C45 H81 BNSi4:
C45 H81 B1 N1 Si4
p (gss, s /p:40) Chrg -1
R: 60000 Res .Pwr . @FWHM
758.5553
757.5581
759.5571
760.5566
761.5569
762.5568
763.5569
Relative Abundance
m/z

Compound 3

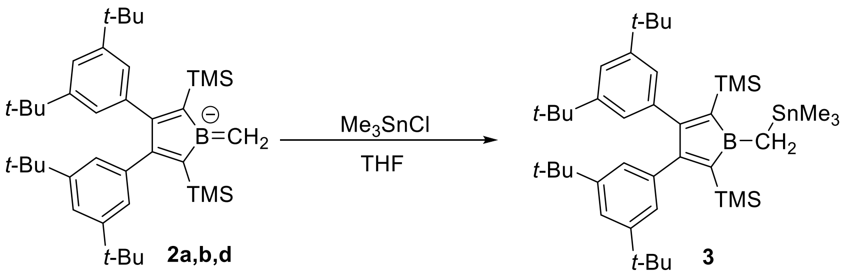

Molecular Weight: 761,72

In a glovebox, compound **2b** (50.3 mg, 0.065 mmol, 1 eq) was dissolved in dry and degassed THF (4 mL). A solution of Me_3SnCl (13.5 mg, 0.068 mmol, 1.05 eq) in dry and degassed THF (3 mL) was added and the yellow reaction mixture was stirred at ambient temperature for one hour. During this time a fine white solid precipitated but no visible colour change occurred. Volatiles were removed under reduced pressure to yield a yellow solid. This solid was extracted with hexane (3 × 1 mL) and the extracts filtered through a pad of glass fibre (Whatman GF/B). The solvent of the orange-red extract was once again removed under reduced pressure to yield an orange solid (47.5 mg), which was dissolved in a minimal amount of hexane (ca. 3 mL) and stored at –40 °C. The resulting yellow-orange crystals were isolated by decanting off the mother liquor and the crystals were washed with cold hexane (–40 °C, 1 × 0.1 mL). After drying in vacuo, compound **5** (29.3 mg, 0.038 mmol, 60 %) was isolated as an orange powder.

Note: In this metathetic reaction, the application of the THF-adducts of **2** is preferred over the crown-ether solvates. NMR monitoring of the reaction confirms equally clean initial conversions but the crown-ether (derivatives) have been found to be less reliably removed from samples that contain the (Lewis-acidic) free borole **3**. In some cases, in reaction mixtures containing crown-ethers decomposition occurred over the course of 2-3 days.

Analytical Data for Compound 3

NMR:

^{1}H (500.25 MHz, 298 K, C_6D_6, CD_5H at 7.15 ppm): 7.24 (t, $^{4}J_{HH}$ = 1.9 Hz, 2H, *p-H$_{ar}$*), 6.89 (d, $^{4}J_{HH}$ = 1.9 Hz, 4H, *o-H$_{ar}$*), 2.27 (s,^{2}J(^{1}H-$^{117/119}$Sn = 78.4/82.3 Hz, 2H, BC*H$_2$*SnMe$_3$), 1.19 (s, 36H, Ar-C(*Me*)$_3$), 0.41 (s, ^{2}J(^{1}H-$^{117/119}$Sn = 51.9/54.1 Hz, 9H, Sn*Me$_3$*), 0.14 (s, 18H, Si*Me*$_3$).

^{13}C{^{1}H} (100.65 MHz, 298 K, C_6D_6, solvent signal at 128.0 ppm): 178.5 (borole-*C$_{3,4}$*), 149.5 (*m-C$_{ar}$*), 140.6 (*ipso-C$_{ar}$*), 139.4 (borole-*C$_{2,5}$*), 123.2 (*o-C$_{ar}$*), 120.4 (*p-C$_{ar}$*), 34.7 (Ar-*C*(CH$_3$)$_3$), 31.6 (Ar-C(*C*H$_3$)$_3$), 24.9 (^{1}J(^{13}C-^{119}Sn = 45.9 Hz, B-*C*H$_2$SnMe$_3$), 2.0 (Si(*C*H$_3$)$_3$), –5.9 (^{1}J(^{13}C-$^{117/119}$Sn = 321.2/335.7 Hz, Sn(*C*H$_3$)$_3$).

^{11}B (160.50 MHz, 298 K, C_6D_6): 68.7.

^{29}Si-INEPT (79.49 MHz, 298 K, C_6D_6): –10.3.

^{119}Sn (186.55 MHz, 298 K, C_6D_6): –14.4.

UV VIS: in pentane, λ_{max} at 424 nm

Elemental Analysis: $C_{42}H_{71}BSi_2Sn$ calcd C 66.23, H 9.40; observed C 66.89, H 9.52.

LIFDI-MS: calcd exact mass: 762.4 m/z; observed m/z: 762.3 $[M]^+$, 780.3 $[M+H_2O]^+$.

Crystal structure of Compound 3

For further details on the diffraction measurement please see the respective section. Crystals investigated using XRD were obtained from solutions in hexane in a freezer (-40°C).

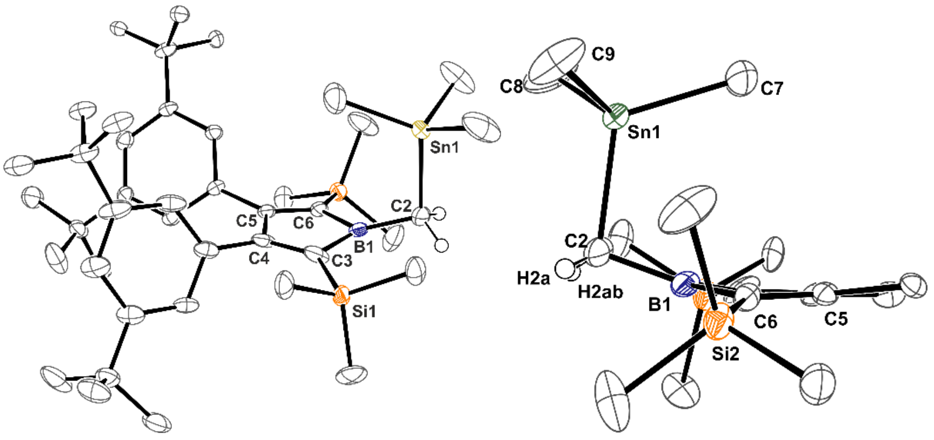

SI-Figure 8-SI (left): ORTEP of the molecular structure of stannaneopentyl borole **3**. ADP are drawn at 50% probability. Non-methylene hydrogen atoms and disorder are omitted for clarity. (right): Detailed excerpt of the central borole ring moiety to illustrate the distorted environment around the boron atom. Selected bond lengths [Å]: B1–C2 1.496(7), B1–C3 1.597(6), C3–C4 1.346(8), C4–C5 1.525(6), C5–C6 1.354(5), C6–B1 1.617(9), C2–Sn1 2.215(3); Sn–CH_3 2.073(5), 2.148(6), 2.180(9).

Spectra Plots for Compound 3

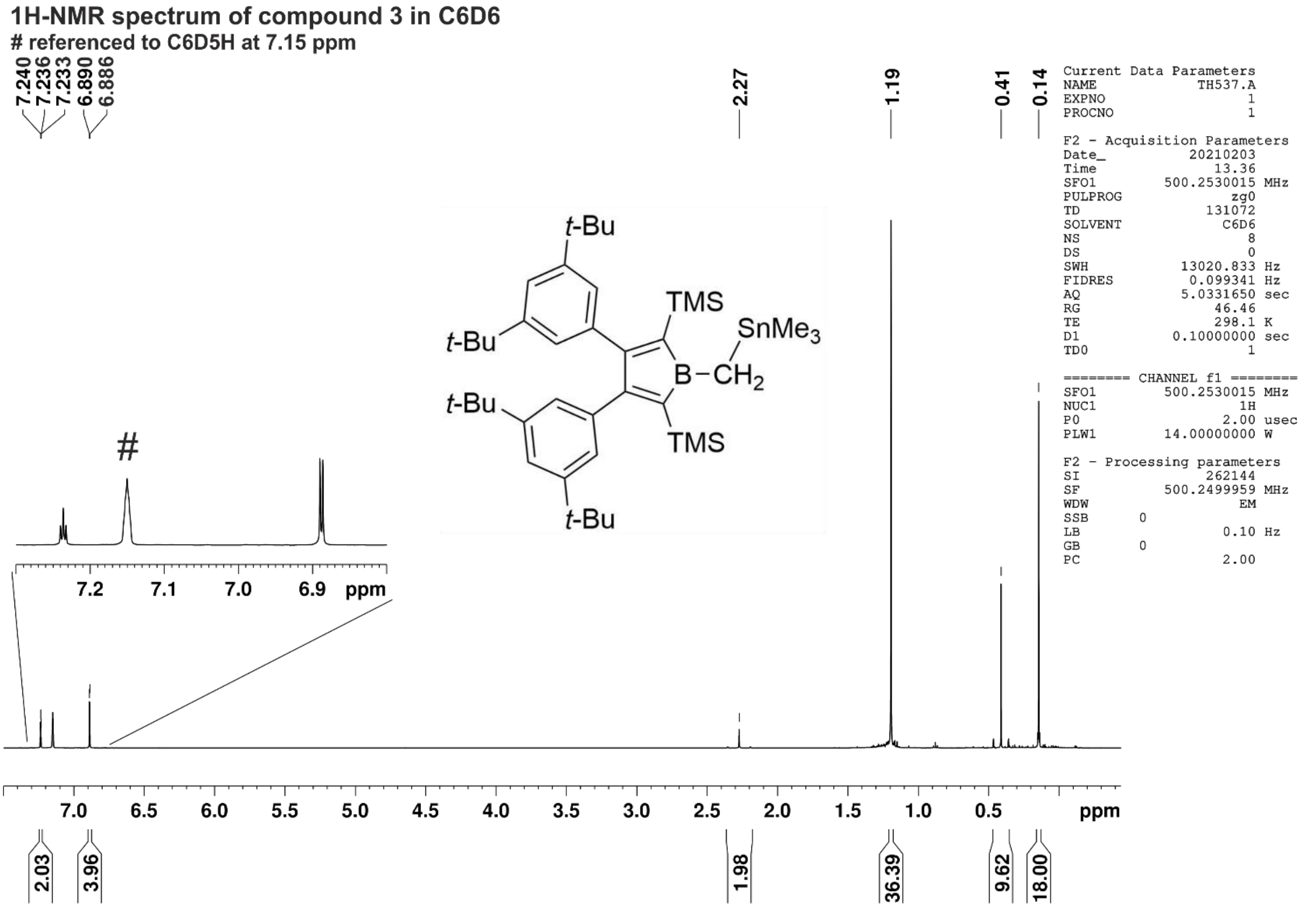

13C{1H}-NMR spectrum of compound 3 in C6D6

# referenced to C6D6 at 128.0 ppm

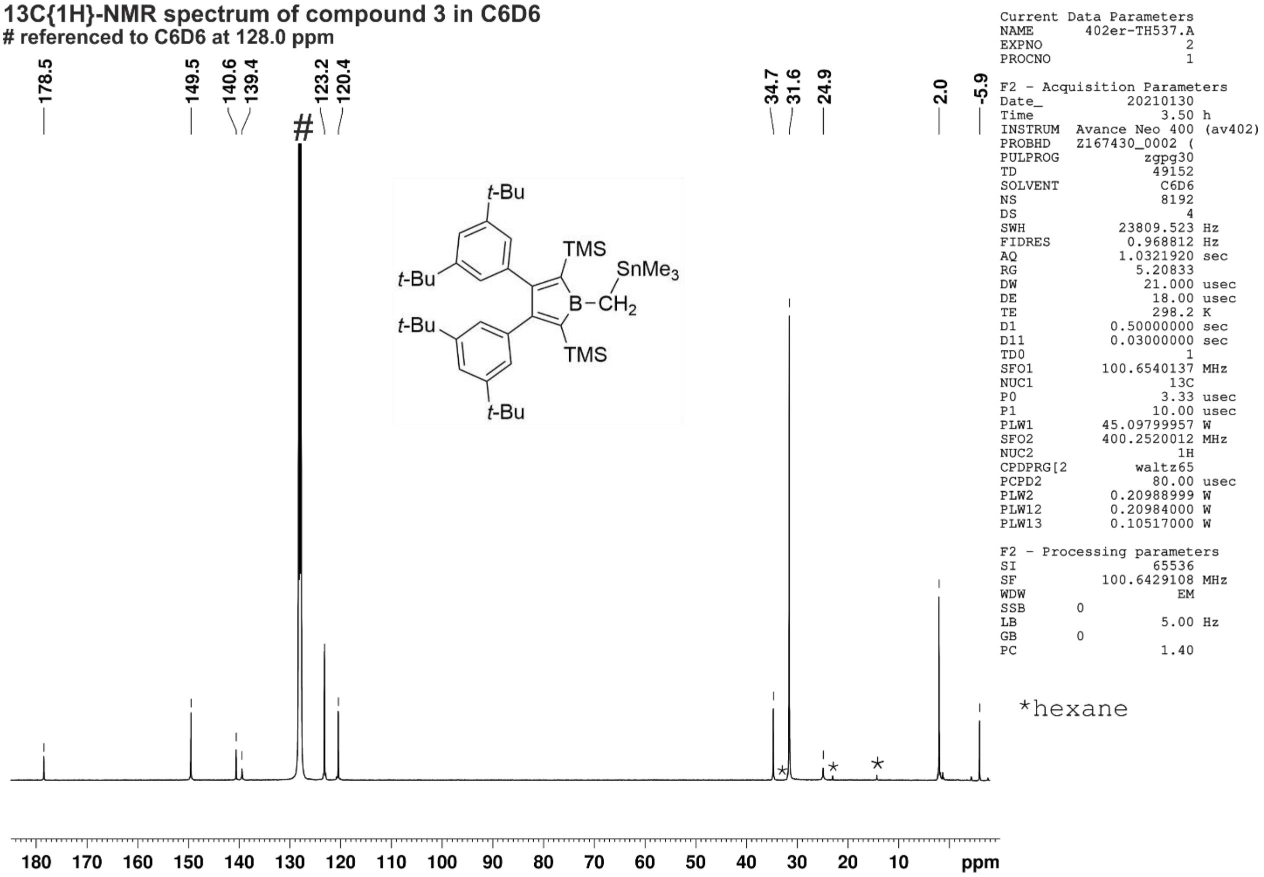

11B-NMR-(background-suppressed) spectrum of compound 3 in C6D6

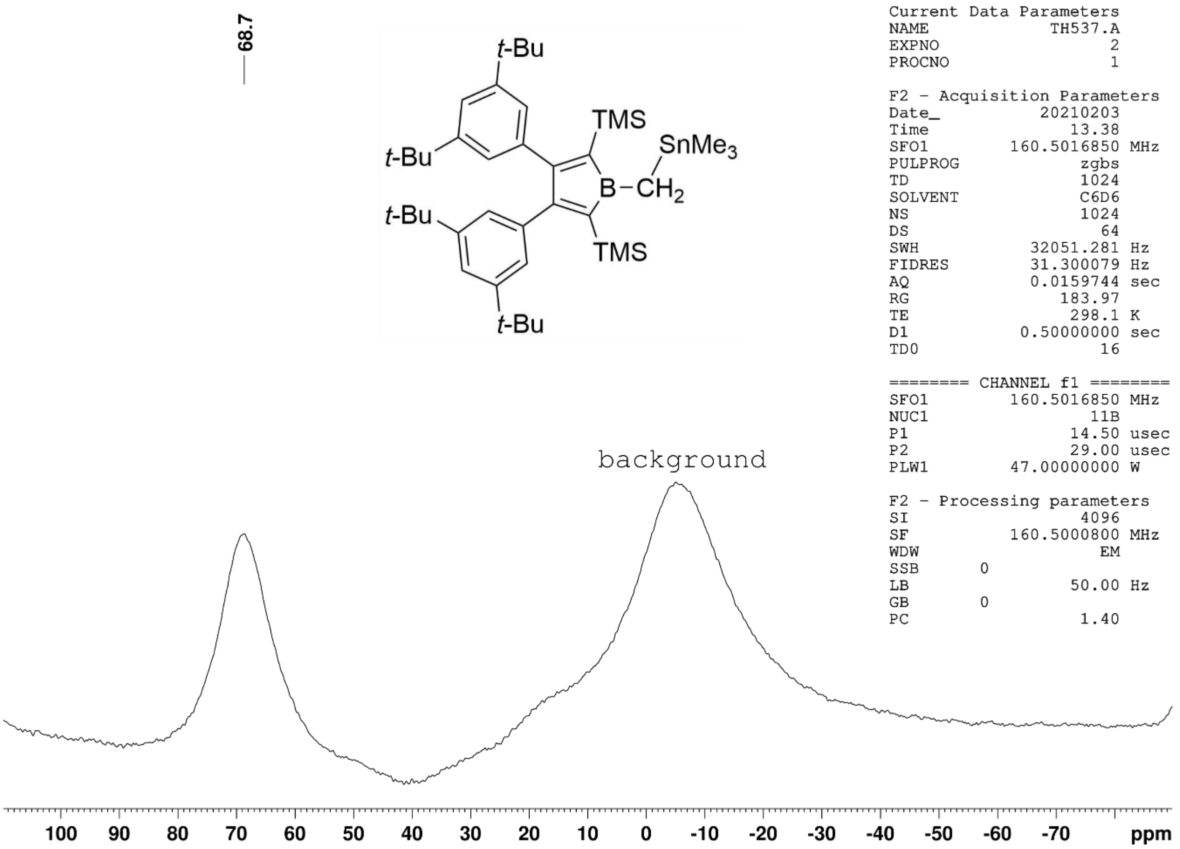

29Si-INEPT-NMR spectrum of compound 3 in C6D6

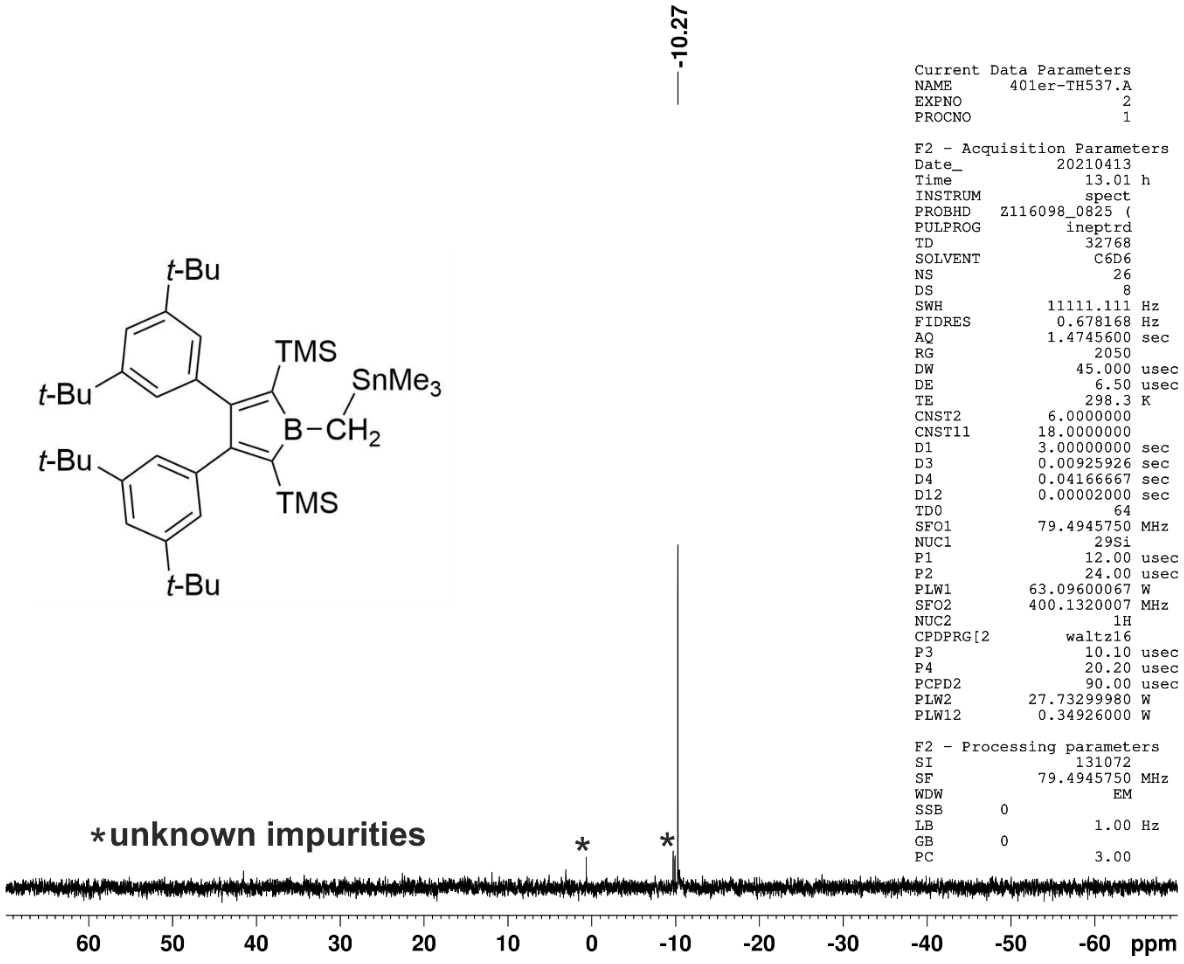

119Sn{1H}-NMR spectrum of compound 3 in C6D6

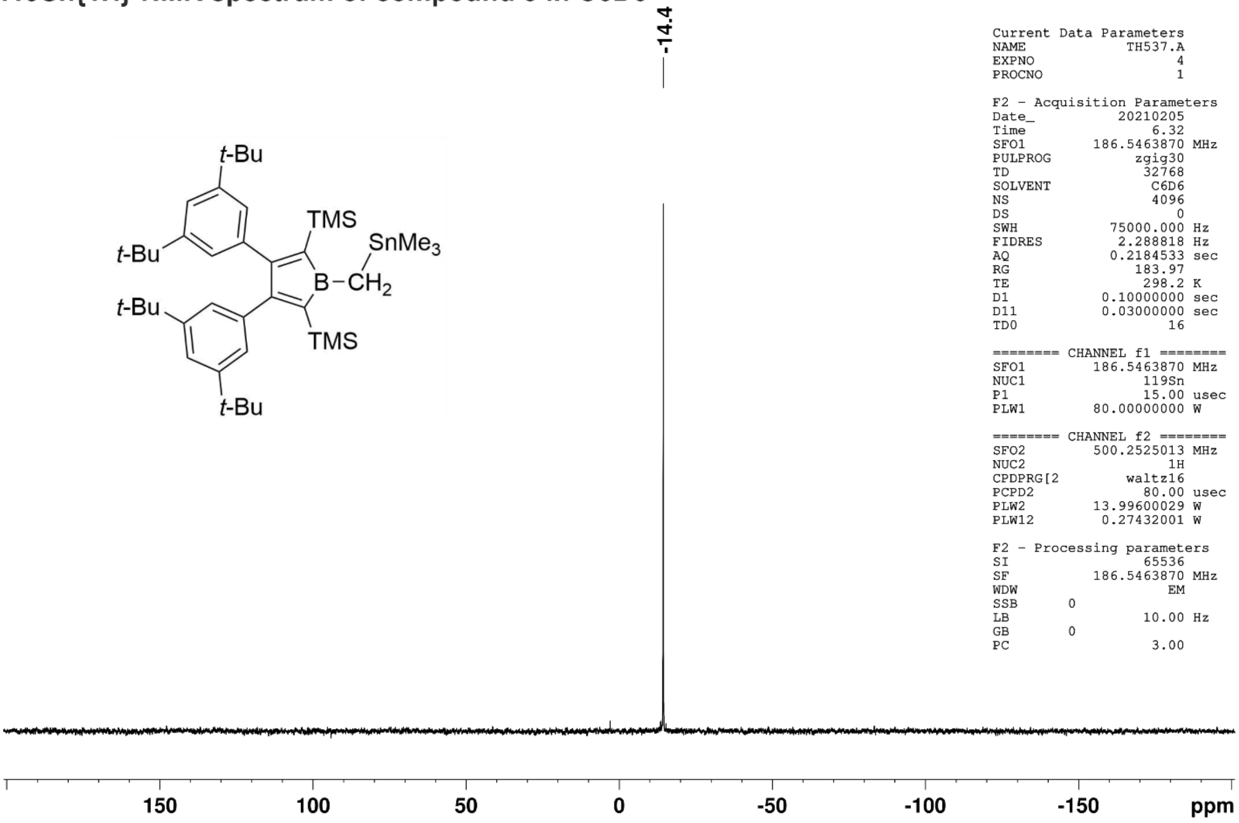

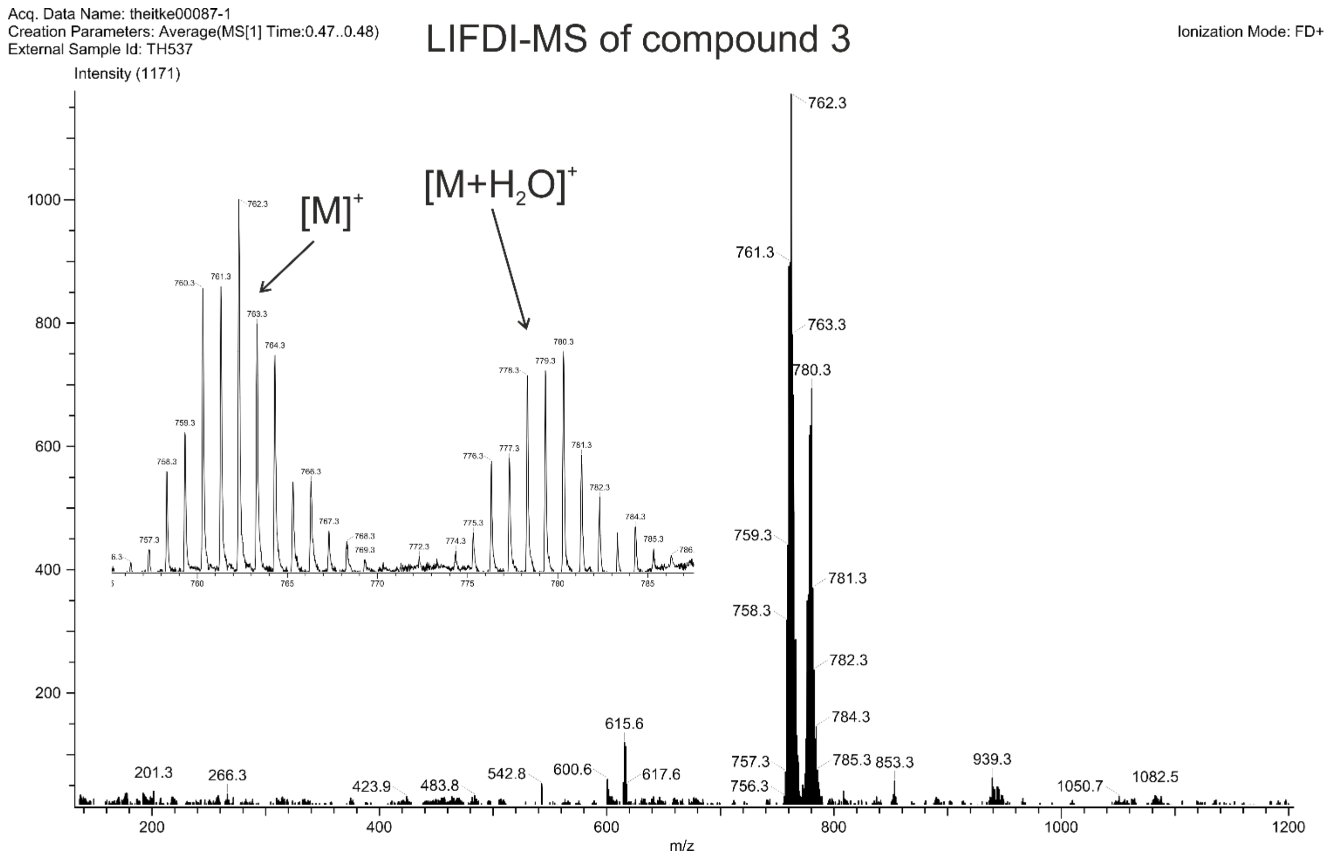

Acq. Data Name: theitke00087-1
Creation Parameters: Average(MS[1] Time:0.47..0.48)
External Sample Id: TH537
LIFDI-MS of compound 3
Ionization Mode: FD+
Intensity (1171)
[M]+
[M+H2O]+
762.3
761.3
763.3
780.3
759.3
781.3
758.3
782.3
784.3
615.6
757.3
785.3
853.3
939.3
756.3
201.3
266.3
423.9
483.8
542.8
600.6
617.6
1050.7
1082.5
m/z

NMR Reaction of 2 with Benzophenone

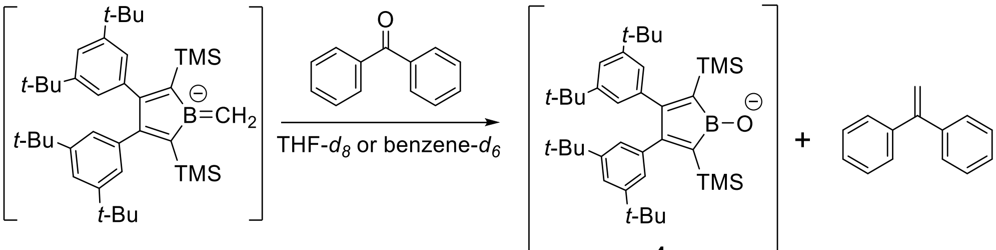

2a Molecular Weight: 597,91

4 Molecular Weight: 599,88 isolated as [K(18-crown-6] salt

In a glovebox, compound **2a** (6.3 mg, 6.3 µmol, 1 eq) was dissolved in C_6D_6 (0.6 mL). Benzophenone (ca. 1.8 mg, 9.5 µmol, ca. 1.5 eq) was added and the reaction solution was transferred into a *Young*-type NMR tube fitted with an air-tight teflon-valve. The reaction mixture is then kept at ambient temperature. The progress of the reaction was monitored by ^{1}H-NMR spectroscopy over the course of a week to give insight into the species formed in the course of the reaction.

1. Initially **2** and benzophenone vanish over the course of the first 12h to produce a σ_v -symmetric product **C** that we assign to anionic oxaboretan on account of its spectroscopic features (^{1}H: CH_2 group at 2.82 ppm, ^{13}C by HSQC- & HMBC-NMR: CH_2 group at 24.3 ppm, CPh_2 group at 85.2 ppm, ^{11}B: at 11.2 ppm). Only minor amounts of 1,1-diphenylethylene are formed in this first period.
2. After ca. 12h, **2** is almost completely consumed and **C** is the dominating species. However, signals of further intermediates of lower symmetry (lacking σ_v mirror symmetry) are observed to increasingly form of which one reveals very broad signals. The unidentified intermediate with broad signals is only observed after 12-32 h and then vanishes until only intermediate **D** is observed in the mixtures. For **D**, that features sharp resonances, we putatively propose a bicyclic oxaborole structure on account of the following observations: i) the methylene group protons are diastereotopic and are observed as two doublets at 3.65 ppm and 3.25 ppm (the latter partially superimposed by the strong crown-ether resonance). ii) two signal sets for $SiMe_3$ groups and thus loss of a σ_v mirror symmetry through the borole moiety.
3. Over the course of the following 36h **C** is increasingly consumed and **D** and 1,1-diphenylethylene and signals assigned to **4** increase.
4. After approx. 7d only minor amounts of **D** are still present and the mixture reveals dominant signals of **4** and 1,1-diphenylethylene.
5. After 7 days, apart from minor impurities and unreacted benzophenone, the mixture exclusively contains signal sets assigned to **4** and 1,1-diphenylethylene. Since both **C** and **D** are eventually transformed into the final products, we conclude that cyclo-reversion can either occur from both intermediates **C** and **D** or the actual species from which cyclo-reversion (to form the olefin and the borole oxide) occurs is (reversibly) accessible from **C** or **D**.

NMR spectra monitoring the Borata-Wittig Reaction with Benzophenone

Reaction mixture **2a** [K(18-crown-6)]**2** + (exc) benzophenone in C6D6 // after ca. **20** min

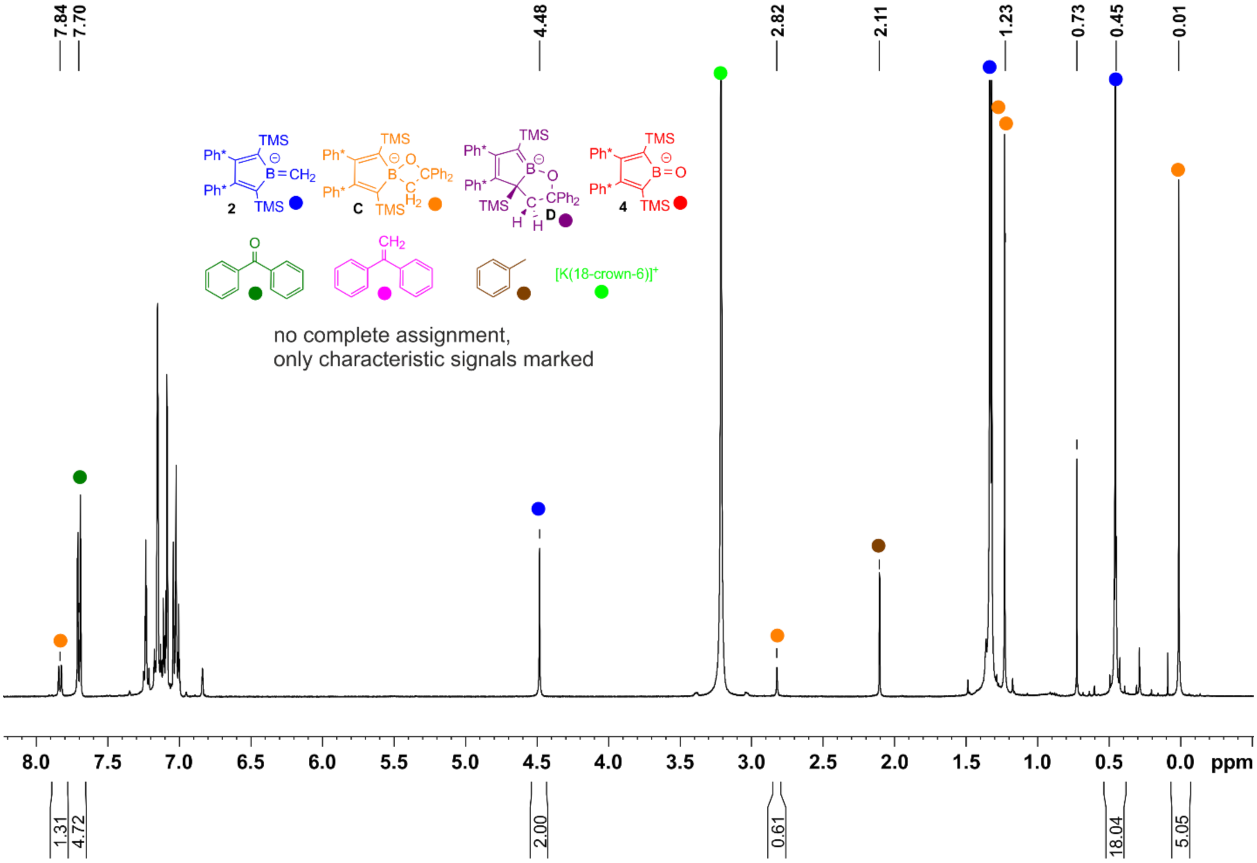

Reaction mixture **2a** [K(18-crown-6)]**2** + (exc) benzophenone in C6D6 // after ca. 3 h

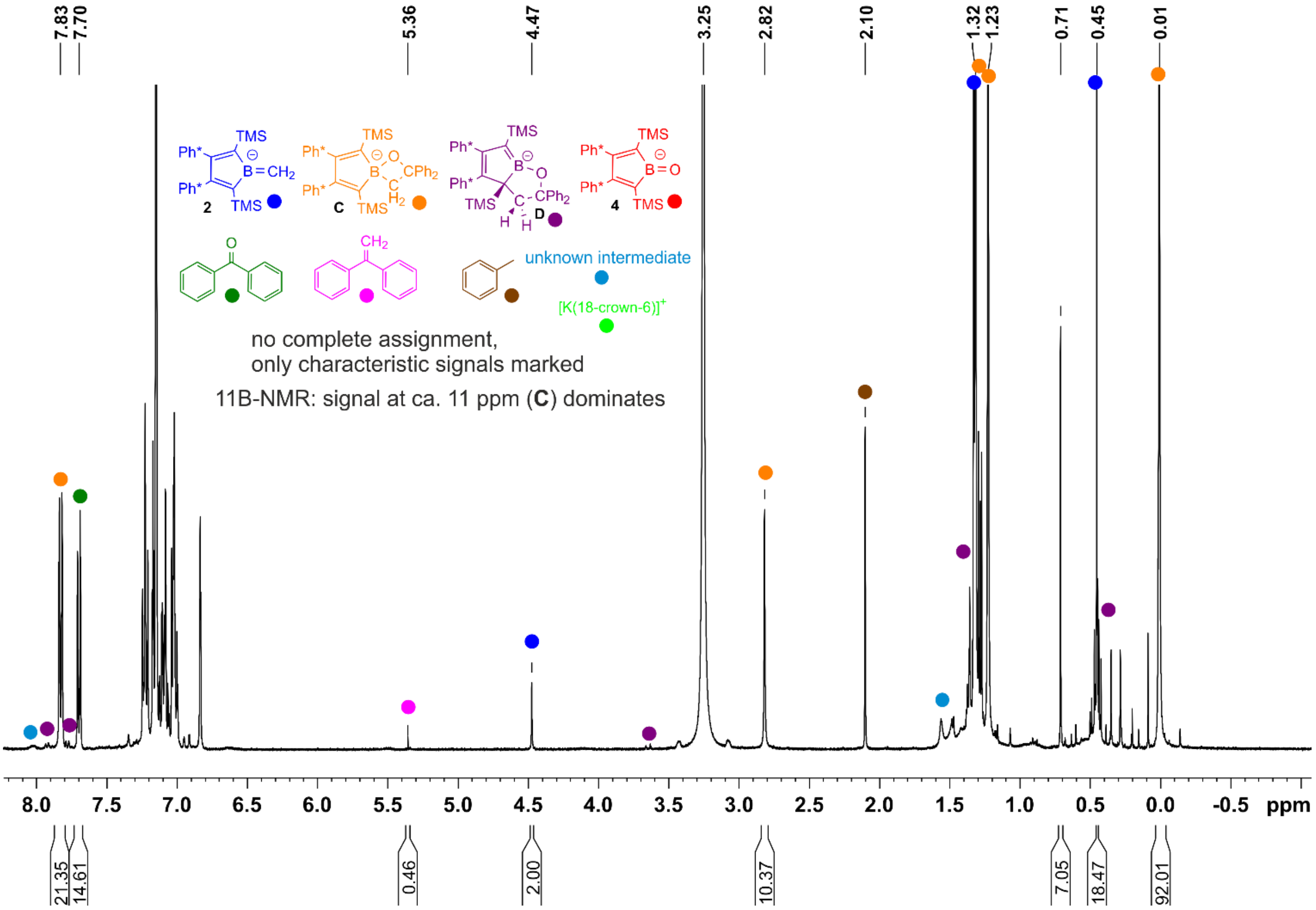

Reaction mixture **2a** [K(18-crown-6)]**2** + (exc) benzophenone in C6D6 // after ca. 9 h

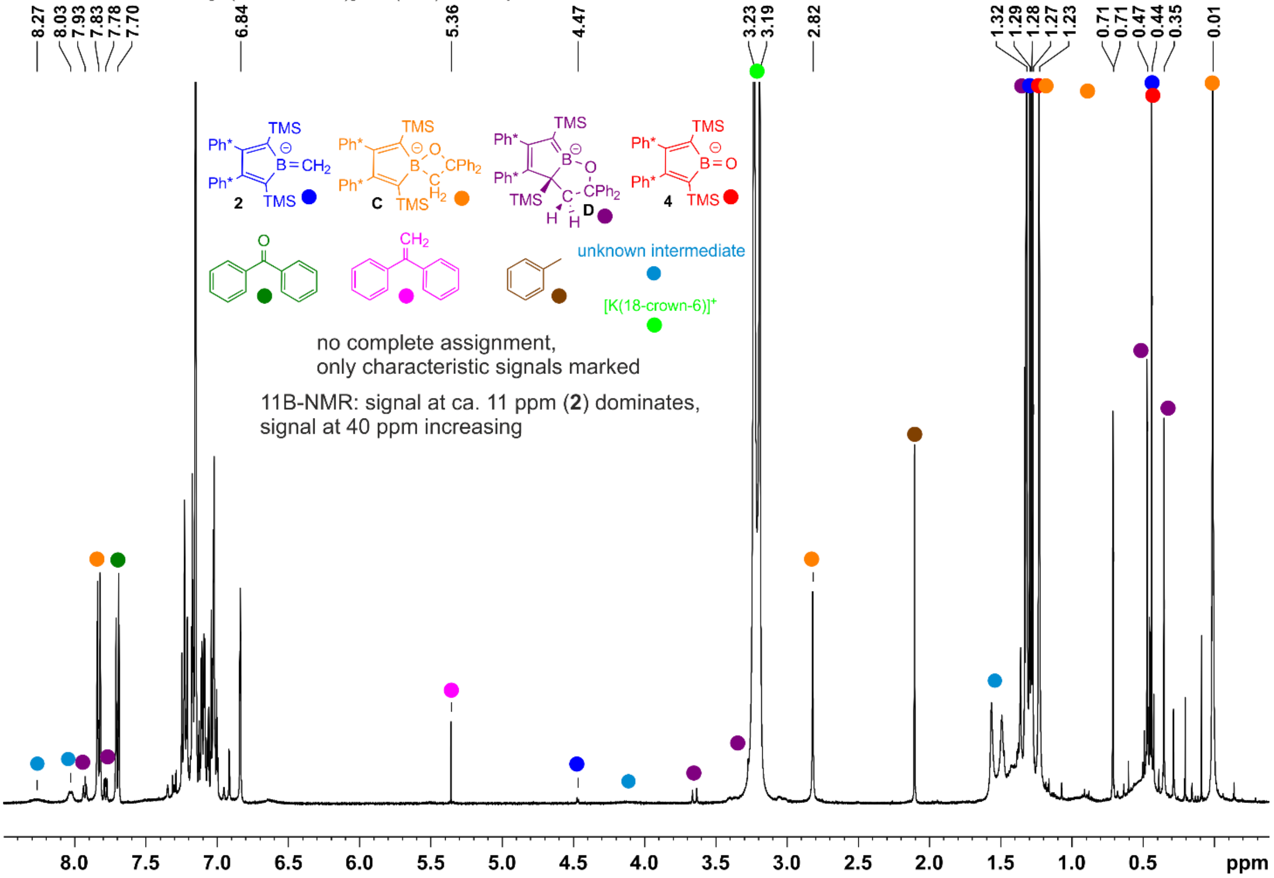

Reaction mixture **2a** [K(18-crown-6)]**2** + (exc) benzophenone in C6D6 // after ca. 21 h

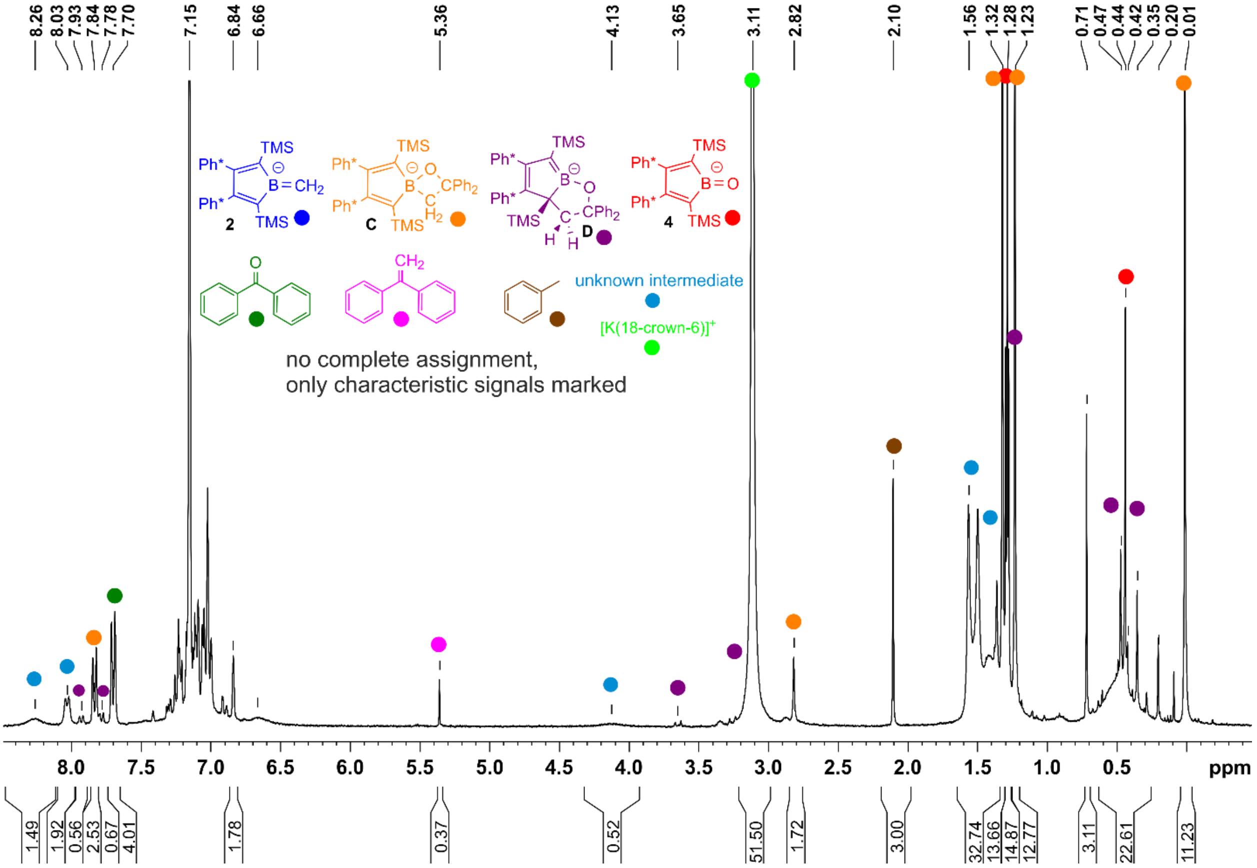

Reaction mixture **2a** [K(18-crown-6)]**2** + (exc) benzophenone in C6D6 // after ca. 32 h

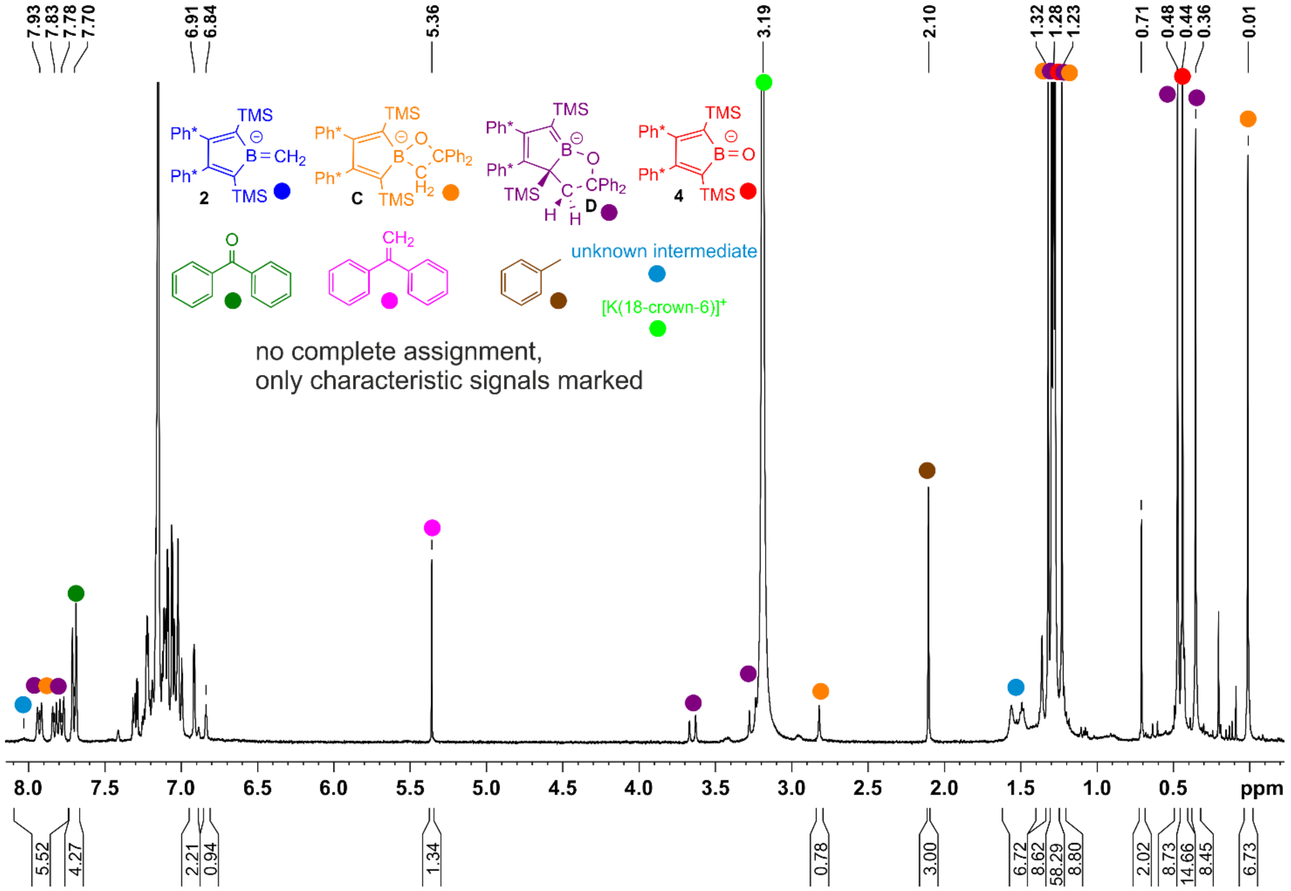

Reaction mixture **2a** [K(18-crown-6)]**2** + (exc) benzophenone in C6D6 // after ca. 2d

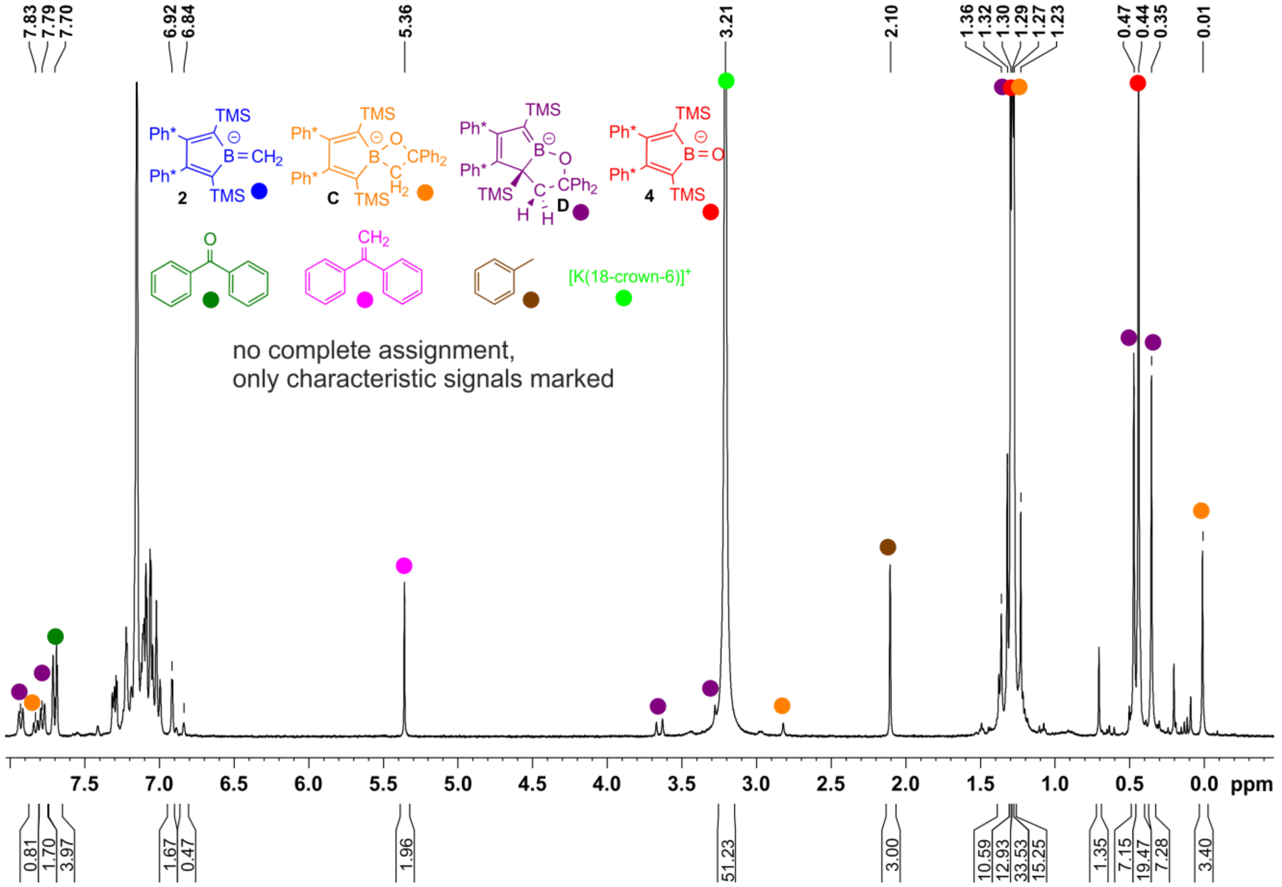

Reaction mixture **2a** [K(18-crown-6)]**2** + (exc) benzophenone in C6D6 // after ca. 78 h

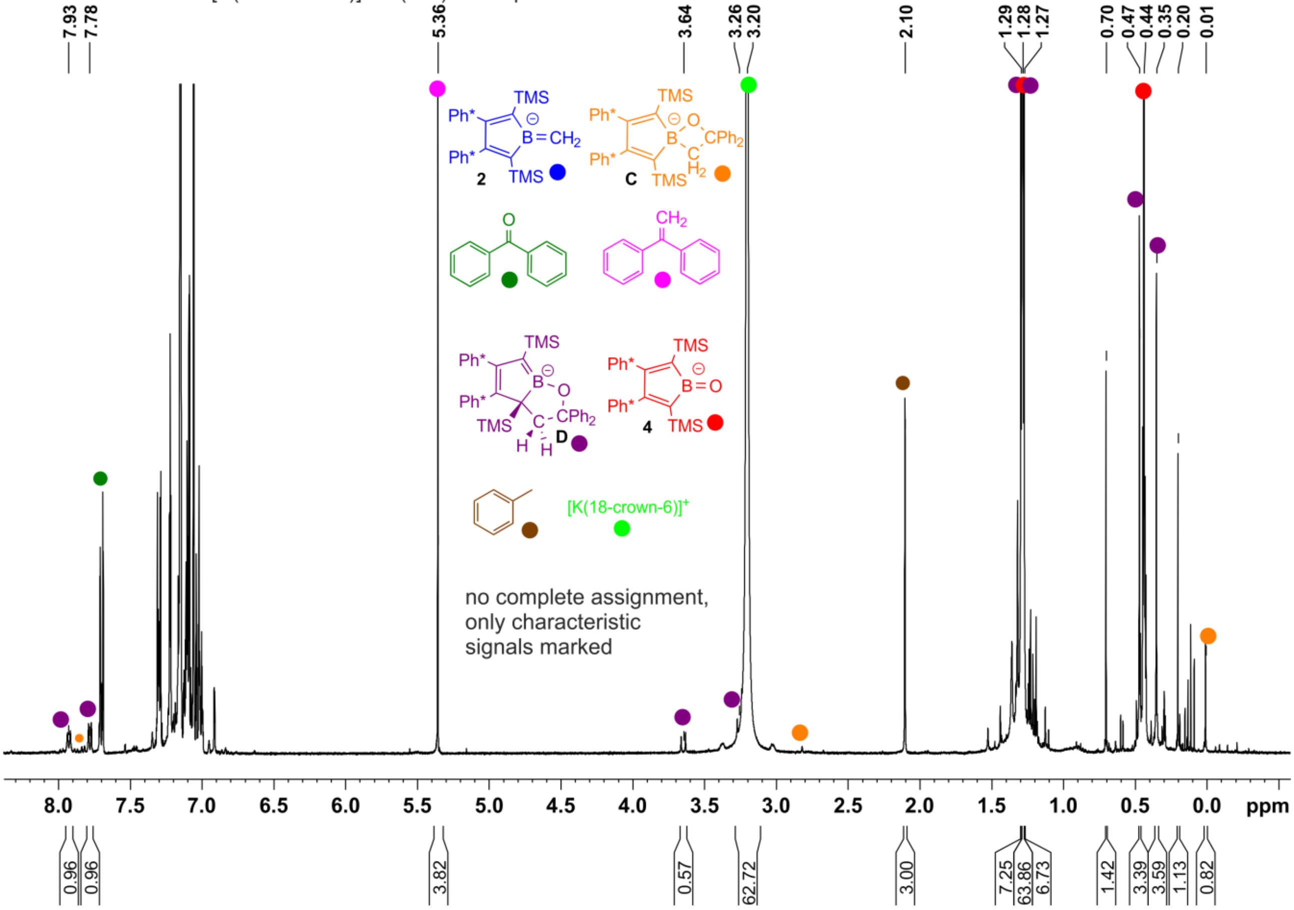

Reaction mixture **2a** [K(18-crown-6)]**2** + (exc) benzophenone in C6D6 // after ca. 7d

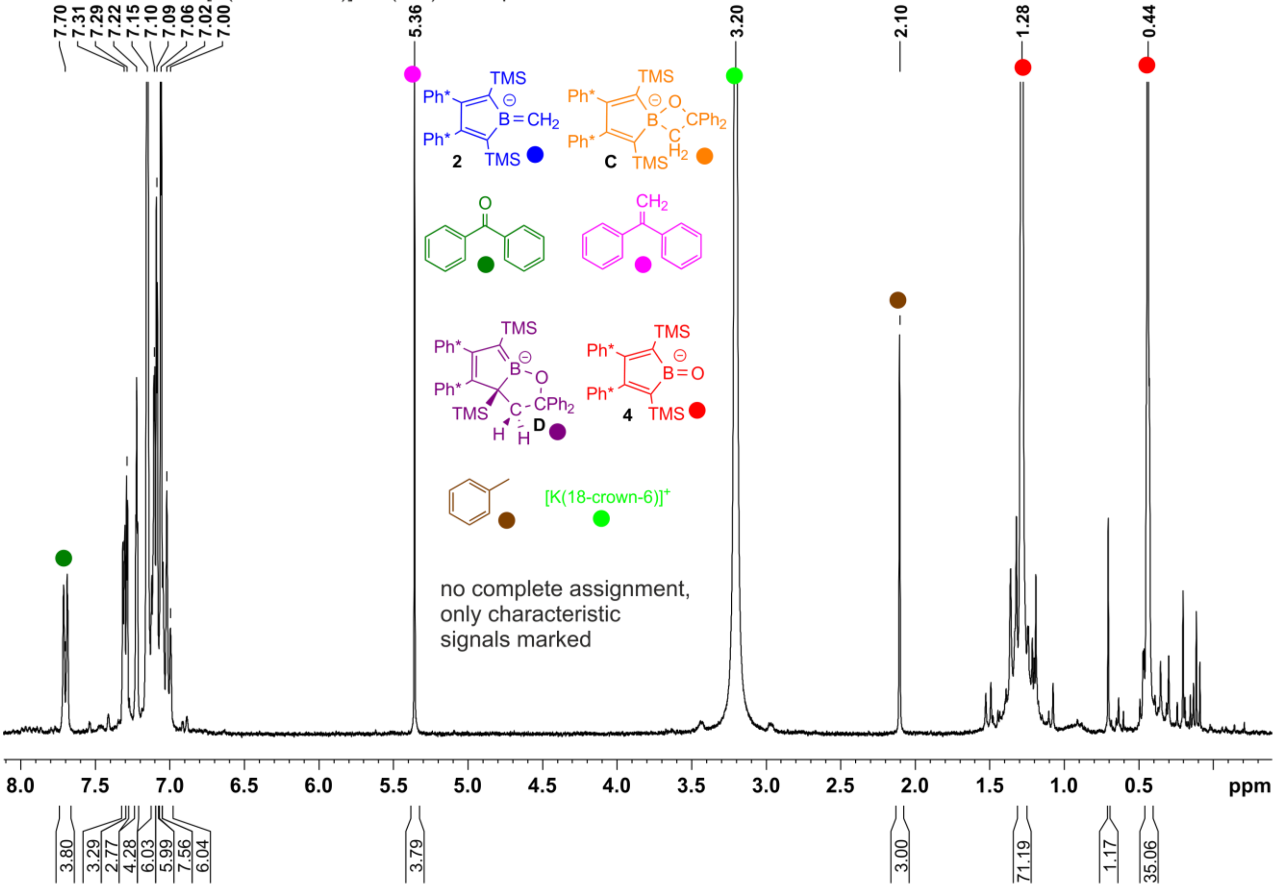

Compound [K(18-crown-6)]4

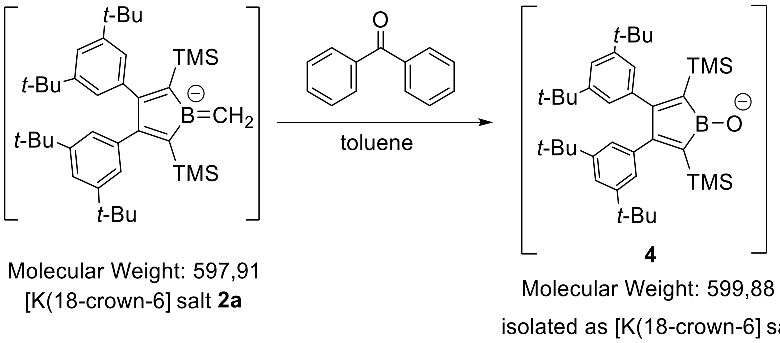

Molecular Weight: 597,91
[K(18-crown-6] salt **2a**

4
Molecular Weight: 599,88
isolated as [K(18-crown-6] salt

Preparative scale reaction of the NMR monitored reaction described above: In a glovebox, compound **2a** (as the toluene solvate, 55.4 mg, 0.056 mmol, 1 eq) was dissolved in toluene (7 mL). Benzophenone (10.2 mg, 0.056 mmol, 1eq) was added and the initially intensely yellow reaction mixture was left standing at ambient temperature for a total of 14 days. The reaction mixture turns increasingly orange-red over the course of 6-8 days after which the colour again fades to finally give a yellow to pale orange solution. The solvent of the reaction mixture was then removed under reduced pressure to yield a red, sticky solid (56.0 mg). The solid was thoroughly washed with hexane (3 × 2 mL, then 3 × 1 mL) and afterwards dried under reduced pressure. Compound **[K(18-crown-6)]4** (38.1 mmol, 0.042 mmol, 76 %) was obtained as an orange solid.

The crystals used for X-ray diffraction were obtained from solutions of the crude reaction mixture in hexane/toluene.

Analytical Data for Compound [K(18-crown-6)]4

NMR:

^{1}**H** (400.13 MHz, 298 K, C_6D_6, C_6D_5H at 7.15 ppm): 7.22 (t, $^{4}J_{HH}$ = 1.9 Hz, 2H, *p-H$_{ar}$*), 7.06 (d, $^{4}J_{HH}$ = 1.9 Hz, 4H, *o-H$_{ar}$*), 3.21 (s, 24H, OC_2CH_2), 1.28 (s, 36H, Ar-C(*Me*)$_3$), 0.43 (s, 18H, Si(*Me*)$_3$),

^{13}**C{^{1}H}** (100.65 MHz, 298 K, C_6D_6, solvent signal at 128.0 ppm): 170.5 (borole-*C$_{3,4}$*), 148.4 (*m-C$_{ar}$*), 144.3 (*ipso-C$_{ar}$*), 141.9 (borole-*C$_{2,5}$*), 124.0 (*o-C$_{ar}$*), 118.3 (*p-C$_{ar}$*), 69.9 (O*C*H_2CH_2), 34.7 (Ar-*C*(CH_3)$_3$), 31.8 (Ar-C(*C*H_3)$_3$), 2.4 (Si(*C*H_3)$_3$).

^{11}**B** (128.38 MHz, 298 K, C_6D_6): 40.2.

^{29}**Si-INEPT** (79.49 MHz, 298 K, C_6D_6): –11.2.

UV VIS: in toluene, λ_{max} at 471 nm and 343 nm.

Crystal structure of Compound [K(18-crown-6)]4

For further details on the diffraction measurement please see the respective section. Crystals investigated using XRD were obtained from solutions in hexane/toluene in a freezer (–40°C).

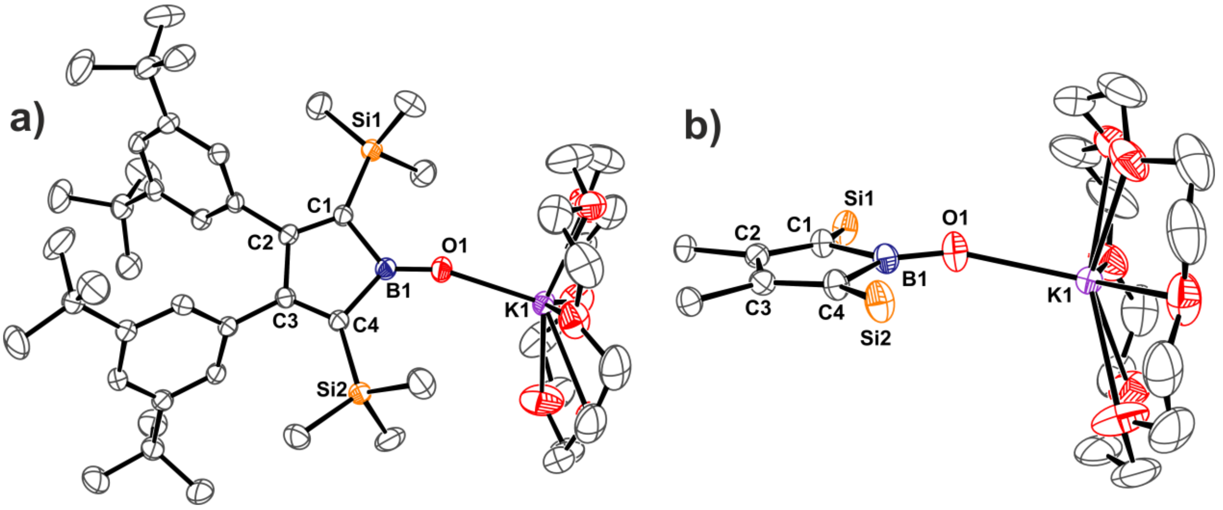

SI-Figure 9-SI (a): ORTEP depiction of the molecular structure of borole oxide [K(18-crown-6)]**4**. ADP are drawn at 50% probability. Hydrogen atoms, severely disordered lattice hexane molecule and minor disorder in *t*Bu groups and the crown-ether fragment are omitted for clarity. (b): Detailed excerpt of the central borole ring moiety. Selected bond lengths and angles [Å]: B1–O1 1.281(3), B1–C1 1.645(4), C1–C2 1.349(2), C2–C3 1.525(3), C3–C4 1.355(3), C4–B1 1.648(3), O1–K1 2.522(2), K1–O(2-7) 2.778(3)-2.892(2); B1-O1-K1 159.7, C4-B1-O1-K1 46.6.

Spectra Plots for Compound [K(18-crown-6)]4

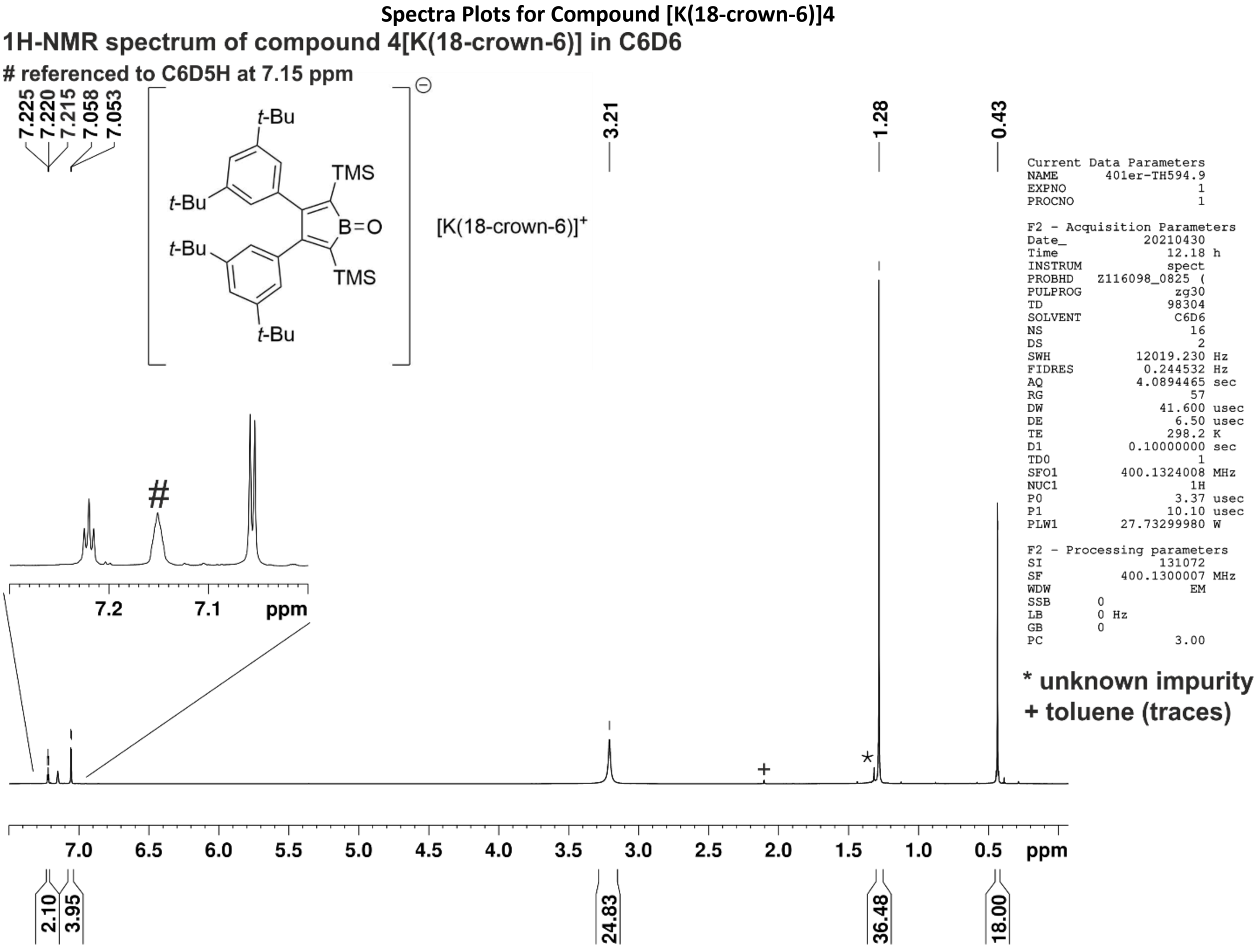

13C{1H}-NMR spectrum of compound 4[K(18-crown-6)] in C6D6

# referenced to C6D6 at 128.0 ppm

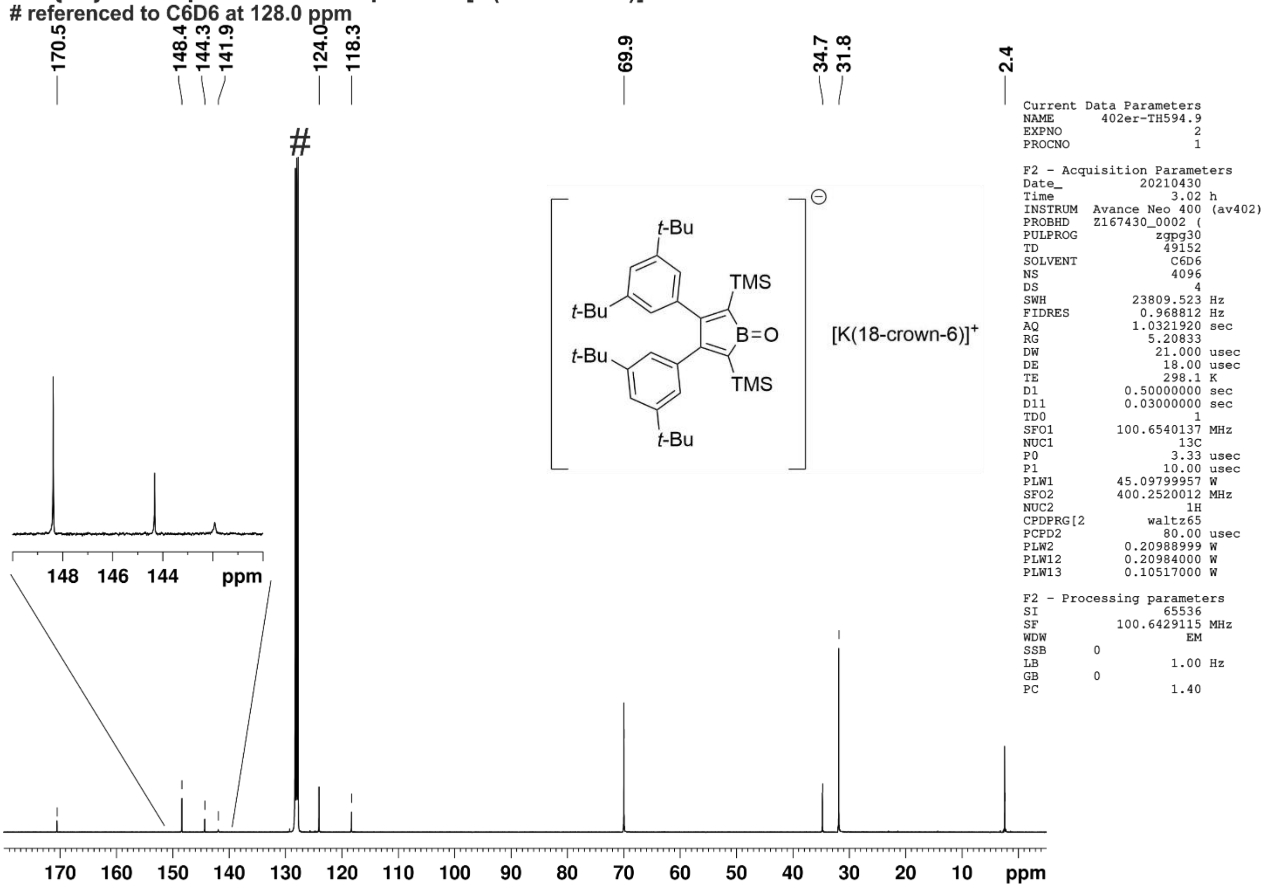

11B(background suppressed)-NMR spectrum of compound 4[K(18-crown-6)] in C6D6

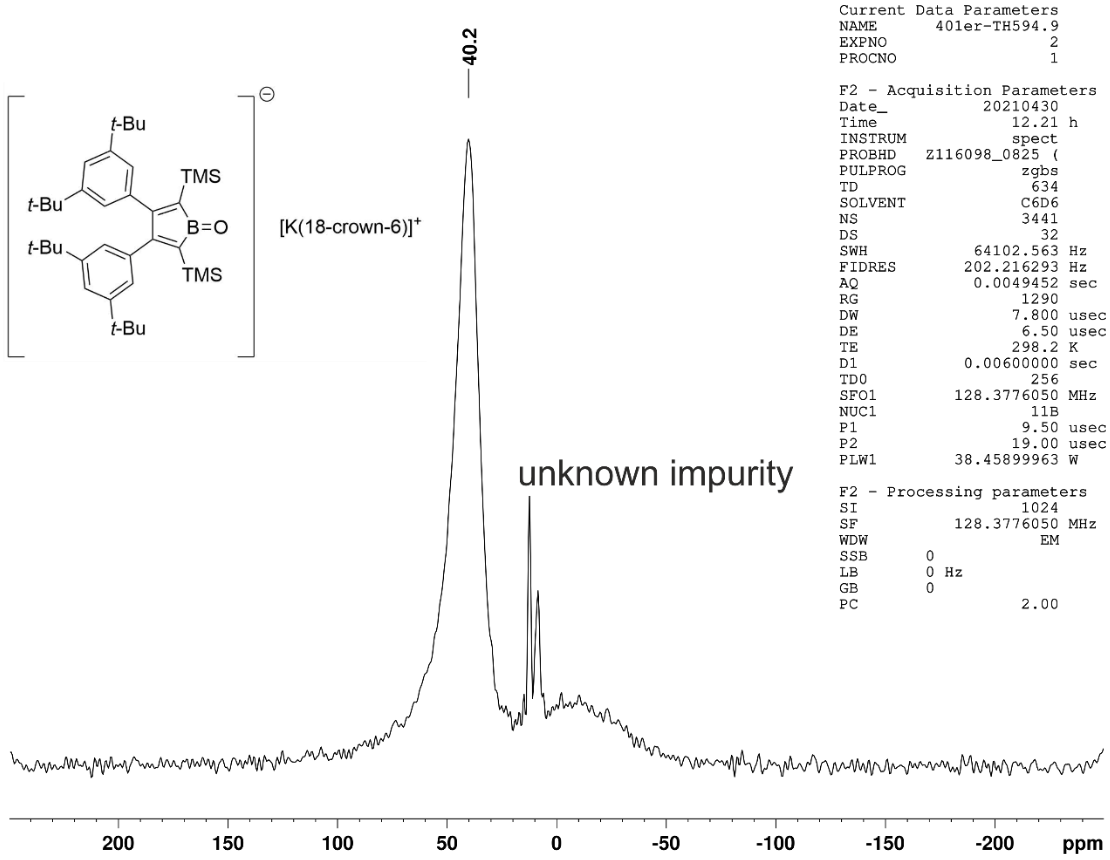

29Si-INEPT-NMR spectrum of compound 4[K(18-crown-6)] in C6D6

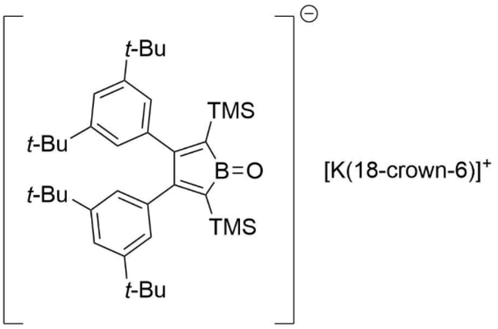

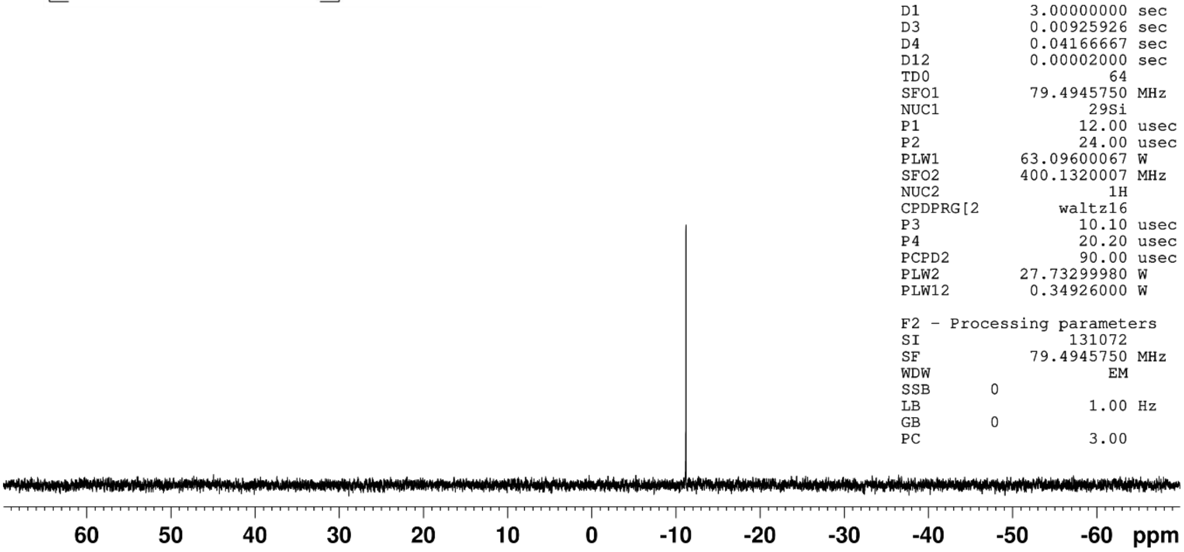

Crystallographic Details

General Data Acquisition and Processing

X-ray data for **1, 2a** ([K(18-crown-6)]**2**), **2d** ([Na(18-crown-6)]**2**), **3**, **B**, **[K(18-crown-6)]4** were collected on Bruker APEX II CCD diffractometers with Mo Kα radiation. If not otherwise stated the data were obtained from crystals cooled to -173°C via a cryo-stream. The data were integrated using SAINT implemented in Bruker's APEX3 programme suite.[4] The qualities of the data quality obtained for $[Na(thf)_2]$**2**, $[K(thf)_2]$**2** and $[K(18\text{-crown-}6)(thf)_2]$**2** were too poor to extract more data than the connectivity pattern. SADABS was used for multi-scan absorption correction.[5] Two domains in twinned crystal of **3** was identified in the reciprocal lattice, sorted, integrated and absorption corrected as a two domain twin using TWINABS.[6] Structure solution was performed with SHELXT[7] and refined using SHELXL[8] along the graphical user interphase of ShelXle.[9] In some cases DSR has been applied to treat disordered solvent molecules.[10] Hydrogen atoms were usually placed with a riding model except for methylene CH_2 in [M]**2** and methyl CH_3 in **B** where they were found and freely refined. Further details on the individual data sets are tabulated in the analytical section of each compound.

Crystallographic and Refinement Details 1

Bright orange crystals of **1** were found to be suitable for X-ray diffraction. The crystals revealed only slow (obvious after ca. 1 h) decolourisation/decomposition when dispersed in fluorinated oil.

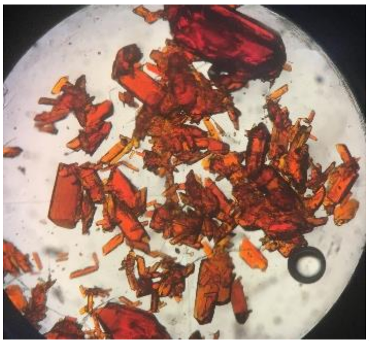

.

Crystallographic and Refinement Details 2a

Pale yellow needle-shaped crystal blocks reliably grow from solutions of **2a** in toluene over the course of a few days. The asymmetric unit contained one molecule of [K(18-crown-6]**2** and a lattice toluene molecule. The potassium ion features a contact to the B=C-moiety. The H-atoms attached to the terminal carbon atom in the exocyclic methylene unit were found in the difference Fourier map and refined without restraints. As would be expected for a planar ethylene moiety, the H-atom positions do not indicate any kind of pyramidalization at the exocyclic C-atom that could indicate the localisation of a lone-pair of electrons. Two *t*-Bu groups were found disordered over two positions and the disorder was modelled accordingly using SAME, SIMU and RIGU commands.

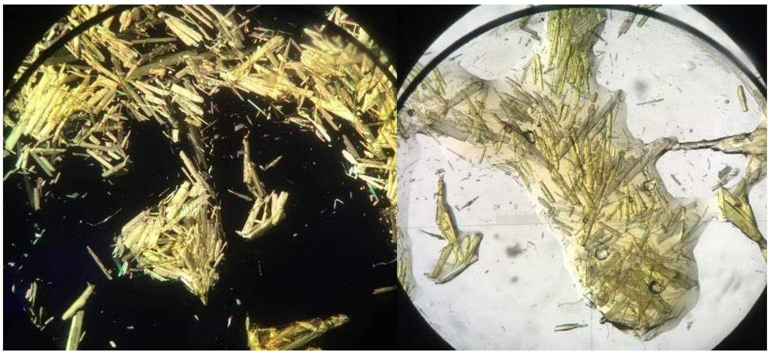

Crystallographic and Refinement Details 2d

Essentially as in **2a**, pale yellow crystal blocks reliably grow from solutions of **2d** in toluene over the course of a few days. The asymmetric unit contained one molecule of [Na(18-crown-6]**2** and a lattice toluene molecule. The sodium ion features a contact to the B=C-moiety. One *t*-Bu group was found disordered over two positions and the disorder was modelled accordingly using SAME, SIMU and RIGU commands.

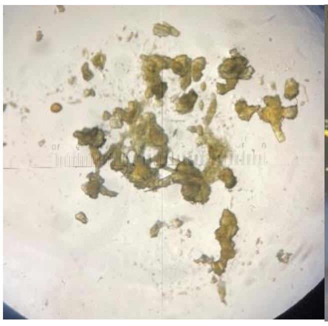 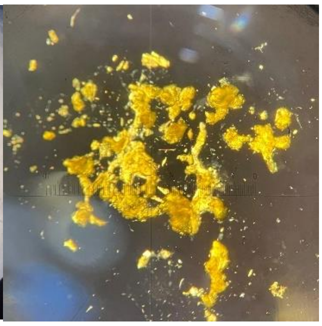

Crystallographic and Refinement Details 3

Yellow crystal plates of compound **3** were grown from solutions in hexane at –40°C. At room temperature the crystals were yellow but when cooled to -173°C the crystals were colourless. The crystal revealed to be a 2-domain twin and the data were integrated and processed using TWINABS. The crystal was refined as a two domain twin with the fraction of the minor component refining to 0.44. A $SiMe_3$ group, and three *t*Bu groups were found to be disordered over two positions each and modelled accordingly using SADI, SIMU and RIGU commands. The modelling of the disorder is depicted below. Highest residual electron density peak and hole are both located close to the heavy Sn atom.

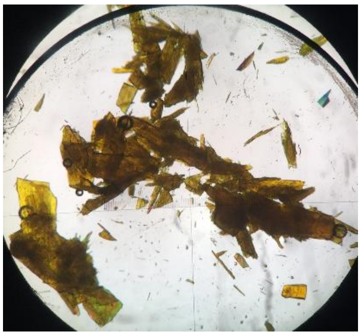 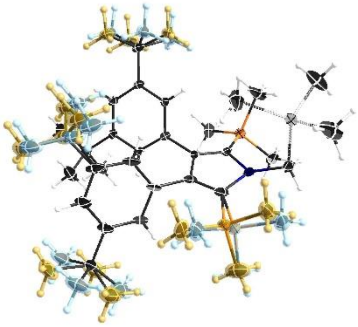

Crystallographic and Refinement Details B

Colourless crystals of [K(18-crowon-6)]**B** were grown from cold (–40°C) solutions in toluene. A *t*-Bu group was found disordered over two positions and modelled accordingly using SADI, SIMU and RIGU commands. The hydrogen atoms at methyl group C1 were found in the difference Fourier map and refined freely.

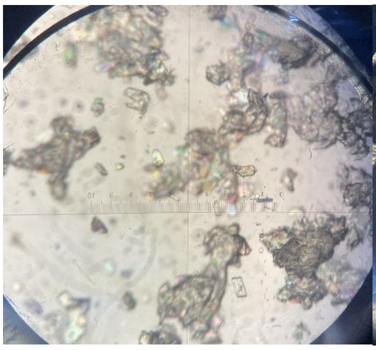 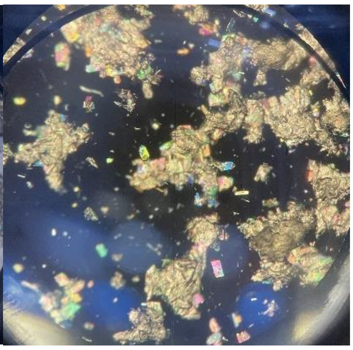

Crystallographic and Refinement Details [K(18-crown-6)]4

Pale orange crystals of [K(18-crown-6)]**4** appeared near colourless from different perspectives. Plate-shaped blocks were grown from cold (–40°C) solutions in hexane/toluene. The structure contained a heavily disordered solvent site which was modelled as one molecule of hexane disordered over two positions. The DSR tool was used and SADI, SIMU and RIGU commands were applied. Further residual electron density hints at yet unmodelled disorder, however further modelling did not provide significant improvements or led to unstable refinements. A *t*Bu group and a subunit within the crown-ether molecule were found disordered over two positions and modelled accordingly using SADI, SIMU and RIGU commands. A depiction of the asymmetric unit including the disorder modelling is given below. Reflection 123 was omitted in the refinement.

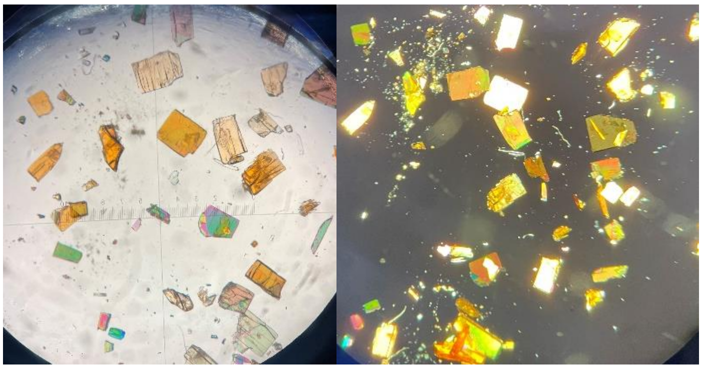

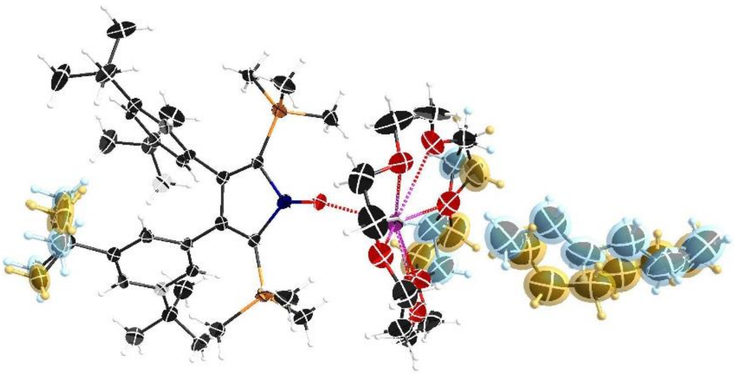

Tabulated Crystallographic Details 1,2(a,d), 3, B and [K(18-crown-6)]4

SI-Table 1-SI: Summarized crystallographic data for data sets of acceptable quality.

	**1**	**2a**×1(C_7H_8)	**2d**×1(C_7H_8)	**3**	**B**	**[K(18-crown-6)]4**
CCDC number	***2081057***	***2081060***	***2081061***	***2081058***	***2081059***	***2081062***
empirical formula	$C_{39}H_{63}Si_2B$	$C_{51}H_{86}BSi_2O_6K×(C_7H_8)$	$C_{51}H_{86}BSi_2O_6Na×(C_7H_8)$	$C_{42}H_{71}BSi_2Sn$	$C_{57}H_{105}BSi_4O_6KN$	$C_{50}H_{84}BKO_7Si_2×(C_6H_{14})$
formula weight	598.88	993.42	977.31	761.66	1062.68	989.43
T / K	100(2)	100(2)	100(2)	100(2)	100(2)	100(2)
λ / Å	0.71073, Mo K_α	0.71073, Mo K_α	0.71073, Mo K_α	0.71073, Mo K_α	0.71073, Mo K_α	0.71073, Mo K_α
crystal system	monoclinic	monoclinic	monoclinic	triclinic	triclinic	monoclinic
space group	$P2_1/c$	$P2_1/c$	$P2_1/c$	$P–1$	$P–1$	$P2_1/c$
a / Å	11.7596 (3)	22.528(4)	22.921(8)	11.5790(16)	11.484(2)	22.151(4)
b / Å	12.5475 (4)	14.996(3)	14.930(5)	12.0097(16)	12.945(2)	14.173(2)
c / Å	26.5574 (8)	18.013(3)	17.673(6)	17.835(2)	22.483(4)	21.363(3)
α	90	90	90	70.540(2)	89.139(4)	90
β / °	99.088 (2)	99.507(3)	99.251(5)	82.054(2)	77.745(3)	113.574(2)
γ	90	90	90	71.695(2)	77.169(3)	90
V / Å³	3869.4 (2)	6001.7(18)	5969(3)	2218.6(5)	3182.9(10)	6147.3(17)
Z	4	4	4	2	2	4
ρ / Mg m^{-3}	1.028	1.099	1.088	1.140	1.109	1.069
μ / mm^{-1}	0.115	0.173	0.111	0.655	0.203	0.170
F(000)	1320	2168	2136	812	1164	2168
crystal size / mm^3	0.43/0.16/0.14	0.40/0.17/0.11	0.36/0.23/0.12	0.16/0.14/0.12	0.18/0.07/0.07	0.29/0.17/0.16
θ range / °	1.6 to 26.5	0.9 to 27.9	0.9 to 26.8	1.2 to 28.7	1.6 to 26.8	1.0 to 27.5
index ranges	$-14\leq h \leq 14$	$-29\leq h \leq 29$	$-28\leq h \leq 29$	$-14\leq h \leq 15$	$-14\leq h \leq 14$	$-28\leq h \leq 28$
	$-15\leq k \leq 15$	$-19\leq k \leq 19$	$-18\leq k \leq 18$	$-14\leq k \leq 15$	$-16\leq k \leq 16$	$-18\leq k \leq 18$
	$-33\leq l \leq 33$	$-23\leq l \leq 23$	$-22\leq l \leq 22$	$0\leq l \leq 23$	$-28\leq l \leq 28$	$-27\leq l \leq 27$
refl. Collected	43548	90420	122493	10340	67288	111777
indep. reflections/ R_{int}	8012/ 0.067	14320 / 0.048	12729 / 0.055	10340 / 0.043	13587 / 0.064	14057 / 0.038
completeness to θ_{max}	99.7 %	100 %	99.8 %	100 % (θ_{full})	99.5%	99.9 %
data/restraints/ parameters	8012 / 0 / 398	14320 / 318 / 702	12729 /138 / 663	10340 / 751 / 579	13587 / 138 / 698	14057 / 530 / 740
GooF	1.04	1.02	1.05	1.19	1.01	1.04
final R indices [I>2sigma(I)] R_1 / wR_2	0.047 / 0.131	0.038 / 0.098	0.043 / 0.112	0.04 / 0.096	0.041 / 0.098	0.063 / 0.181
R indices (all data) R_1 / wR_2	0.059 / 0.122	0.053 / 0.090	0.063 / 0.100	0.042/ 0.095	0.066 / 0.0879	0.078 / 0.169
largest diff. peak and hole / e$Å^{-3}$	0.61/ –0.26	0.41 / −0.26	0.37 / −0.36	1.00 / –1.80	0.38 / −0.29	0.76 / −0.41
absorption correction	multi-scan	multi-scan	multi-scan	multi-scan	multi-scan	multi-scan
miscellaneous		mixed H treatment		2-component twin	mixed H-treatment	

Computational Details

Structure Optimisation, Frequency Calculation and Thermochemical Approximations

Computational examination was performed using ORCA (version 4.2.1.).[11] All structures were optimised starting from (where available) experimental X-Ray structures on RI-BP86-D3BJ[12] def2SVP/J model chemistry[13] in the gas phase followed by a frequency calculation on the same level of theory. Thermochemical corrections were taken from these frequency calculations. For numerical accuracy, grid6 and finalgrid7 were applied. No imaginary frequencies were observed confirming true minima. All structures were then reoptimized using RI-BP86-D3BJ-def2TZVP/J model chemistry. Single-point property calculations (TD-DFT for UV/Vis, solvation effects by CPCM single-points) as well as NBO analyses[14] are based on these gas phase structures. For an energy profile of the reaction of **2** with benzophenone single-point calculations were performed using RI-BP86-D3BJ-def2TZVPP/J and (CPCM=benzene) model chemistry. Graphical depictions were created using ChemCraft or IBOview and electrostatic potential maps were plotted using the AIMAll programme suite.[15] For tin an ECP-28 was applied.[16]

SI-Table 2-SI: Thermochemical data for compounds and reactions considered:

		RI-BP86-D3BJ-**def2TZVP/J** (gasphase structures); thermal corrections from def2-SVP frequency calculations in Hartree							RI-BP86-D3BJ-**def2TZVPP/J** (CPCM = C_6H_6)
**ENTRY**	**MOLECULE**	**E(SCF)**	**$E(ZPV)_{corr}$**	**H_{corr}**	**G_{corr}**	**E(ZPV)**	**H**	**G**	**$E(SCF)_{solv}$**
1	Fulvene ($C_5H_4CH_2$)	-232,2890694	0,095040875	0,101071249	0,06699064	-232,1940286	-232,1879982	-232,2220788	
2	benzene	-232,3443076	0,097833847	0,103280834	0,070951754	-232,2464738	-232,2410268	-232,2733559	
3	**Boratafulvene 2^H^**	-219,063847	0,08927461	0,09585055	0,06071972	-218,974572	-218,967996	-219,003127	
4	boratabenzene	-219,113704	0,09096992	0,09677129	0,06323391	-219,022734	-219,016933	-219,05047	
5	**1**	-2128,83521	0,89083931	0,94685521	0,81347775	-2127,94437	-2127,88836	-2128,02173	-2128,86476
6	**2 (anion)**	-2128,28496	0,87802195	0,93320161	0,80194648	-2127,40694	-2127,35176	-2127,48301	-2128,345
7	**B (anion)**	-3002,53072	1,11405918	1,18574028	1,02469494	-3001,41667	-3001,34498	-3001,50603	-3002,59078
8	$[N(SiMe_3)_2]^-$ (anion)	-873,59371	0,21667147	0,23481688	0,17413534	-873,377038	-873,358893	-873,419574	-873,643061
9	$HN(SiMe_3)_2$	-874,180142	0,23066109	0,24882768	0,18899805	-873,949481	-873,931314	-873,991144	-874,191104
10	**3**	-2462,49162	0,9874997	1,05201421	0,90233898	-2461,50412	-2461,43961	-2461,58928	
11	benzophenone	-576,885934	0,18633223	0,19830698	0,1497646	-576,699601	-576,687627	-576,736169	-576,896178
12	**C (anion)**	-2705,22564	1,06845959	1,13465992	0,98236443	-2704,15718	-2704,09098	-2704,24328	-2705,28872
13	**D (anion)**	-2705,25252	1,07016062	1,13609395	0,98404061	-2704,18236	-2704,11643	-2704,26848	-2705,31339
14	**D* (anion)**	-2705,20638	1,06870222	1,13496908	0,98170181	-2704,13767	-2704,07141	-2704,22467	-2705,26549
15	**4** (**anion**)	-2164,27751	0,85644918	0,91117244	0,78096393	-2163,42107	-2163,36634	-2163,49655	-2164,34149
16	1,1-diphenylethylene	-540,935256	0,20879933	0,22109946	0,17215215	-540,726456	-540,714156	-540,763104	-540,944318
17	[K(18-crown-6)]+	-1523,32653	0,35931653	0,38186934	0,31118656	-1522,96722	-1522,94467	-1523,01535	-1523,37426
18	**2a** ([K(18-crown-6)]**2**)	-3651,74409	1,24075773	1,31847044	1,14453029	-3650,50333	-3650,42562	-3650,59956	-3651,793247
19	**C-**[K(18-crown-6)]	-4228,6931	1,43101009	1,52002847	1,32447869	-4227,26209	-4227,17307	-4227,36862	-4228,746622
20	[K(18-crown-6)]**4**	-3687,7405	1,21823357	1,29605444	1,12038416	-3686,52226	-3686,44444	-3686,62011	-3687,78766

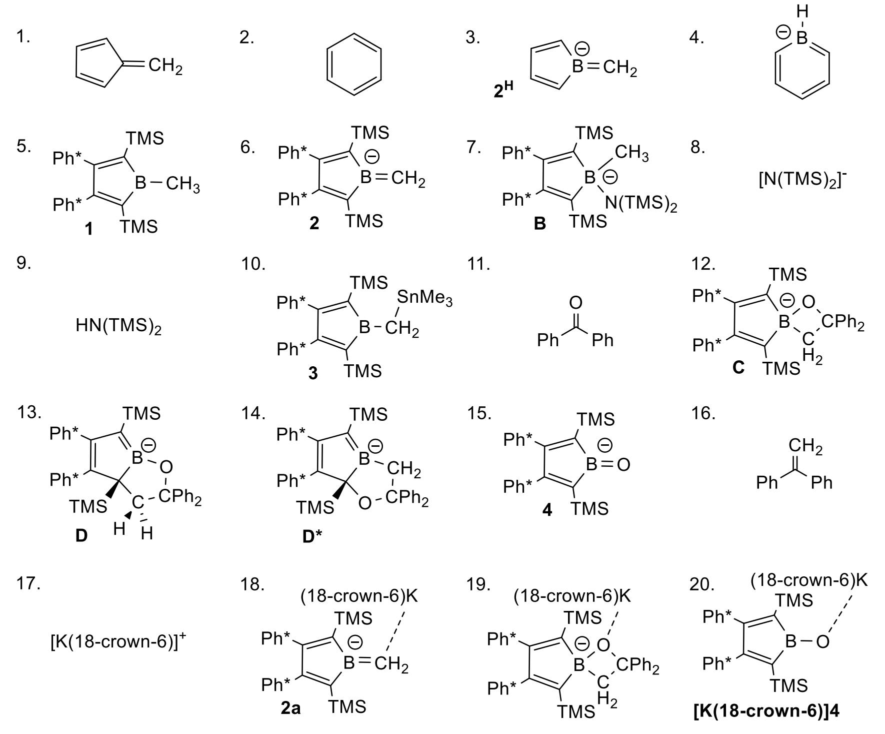

SI-FIGURE 10-SI: Structure depictions of the computationally considered compounds as listed in **SI-Table 2-SI**

SI-Table 3-SI: Thermochemical data summary for some considered reactions

			RI-BP86-D3BJ-**def2TZVP/J** (gasphase) in kcal mol^{-1}				RI-BP86-D3BJ-**def2TZVPP/J** (CPCM = benzene) in kcal mol^{-1}	
**ENTRY**	**Reactant Entries**	**Reaction Description**	**dE(SCF)**	**dE(ZPV)**	**dH**	**dG**	**dE(SCF)$_{solv}$**	**dG$_{solv}$**
RXN1	1 ---> 2	Isomerisation Fulvene -> Benzene	-34,66	-32,91	-33,28	-32,18		
RXN2	3 ---> 4	Isomerisation Boratafulvene -> Boratabenzene	-31,29	-30,22	-30,71	-29,71		
RXN 3	5 + 8 ---> 6 + 9	Deprotonation **1** + $[N(TMS)_2]^-$ -> **2** + $HN(TMS)_2$	-22,70	-21,97	-22,48	-20,61	-17,75	-15,66
RXN 4	5 + 8 ---> 7	Adduct formation **1** + $[N(TMS)_2]^-$ -> **B**	-63,88	-59,77	-61,33	-40,61	-52,06	-28,79
RXN 5	18 --> 6 + 17	Dissociation **2a** ---> **2**$^-$ + $[K(18\text{-crown-}6)]^+$	83,20	81,06	81,07	63,50	46,43	26,72
RXN 6	6 + 11 ---> 12	**2** + benzophenone --> **C**	-34,36	-31,78	-32,38	-15,12	-29,83	-10,60
RXN7	12 --> 13	**C** --> **D**	-16,87	-15,80	-15,97	-15,82	-15,48	-14,43
RXN8	12 --> 14	**C** --> **D***	12,09	12,24	12,28	11,67	14,57	14,16
RXN 9	12 --> 15 + 16	**C** --> **4** + 1,1-diphenylethylene	8,08	6,01	6,56	-10,42	1,82	-16,67
RXN 10	13 --> 15 + 16	**D** --> **4** + 1,1-diphenylethylene	24,95	21,82	22,53	5,40	17,30	-2,24
RXN 11	20 --> 15 + 17	Dissociation [K(crown)]**4** --> **4** + $[K(18\text{-crown-}6)]^+$	85,62	84,03	83,71	67,76	45,12	27,26

Frontier Orbital Details and Bonding Indices for 1,2,3 and 4

Frontier Orbitals, Löwdin and Mayer Bond orders are reported for the RI-BP86-D3BJ-def2TZVP/J obtained gas-phase structures of the isolated molecules or anions respectively. Only one of two typically nearly identical values is given for the mirror-symmetry related bonds. Structure depictions below also represent the Natural Lewis Structure (NLS) as suggested by NBO analyses in each case.

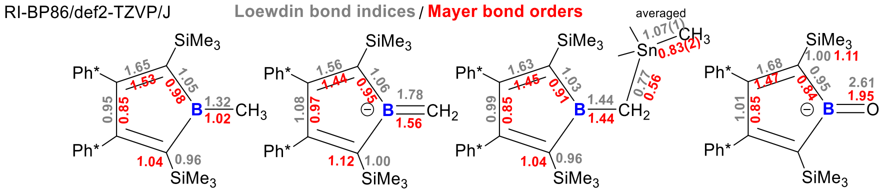

	1	2 (anion)	3	4 (anion)
LUMO	-3.33 eV	0.84 eV	-3.01 eV	0.55 eV
HOMO	-5.14 eV	-1.19 eV	-5.05 eV	-0.77 eV
HOMO-1		-1.58 eV		-1.91 eV
Δ(H-L)	1.81 eV	2.03 eV	2.04 eV	1.32 eV

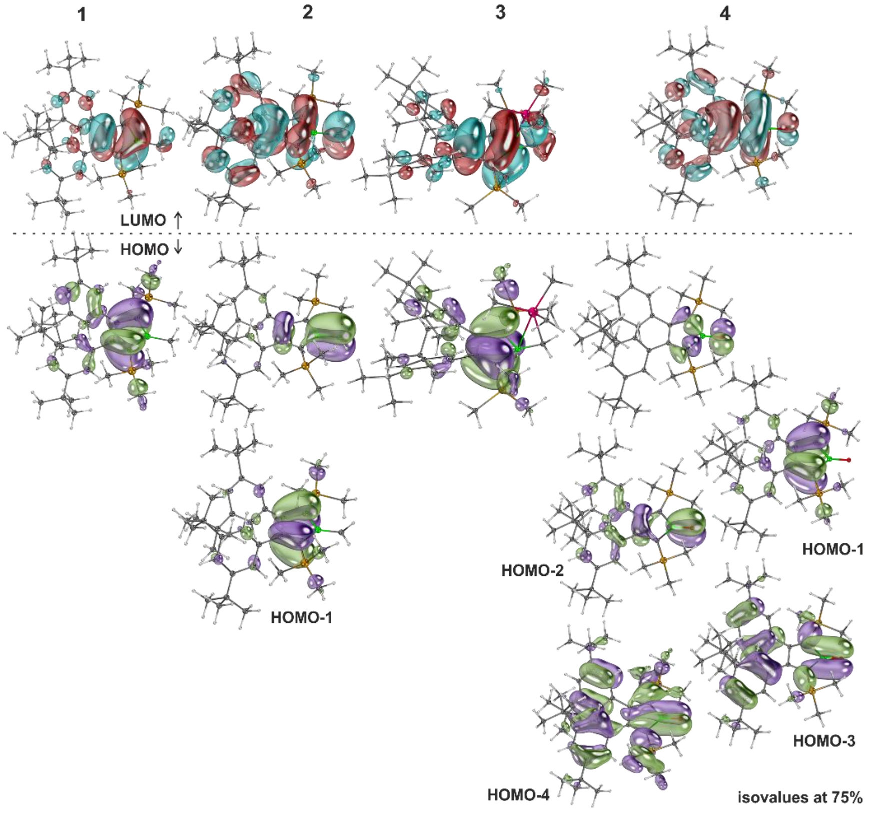

NBO and NRT Analyses

NBO and NRT analyses were conducted using NBO7.0.[14] Wavefunctions to be analysed by NBO were obtained on the structures optimised on RI-BP86-D3BJ-def2TZVP/J level of theory. BP86 single point calculation with def2-SVP basis sets on the organic substituents and def2-TZVP basis set for the elements of the central heterocyclic fulvene or [C_4BX]-fragment were chosen. NRT calculations were restricted to consider resonance structures of the central fulvene or [C_4B-X] unit. Selected depictions of NBOs are given below.

For the analysis of various hyperconjugation effects of α-C-Sn bonds into empty p-orbitals of a variety of boranes the structures were optimised in RI-BP86-D3BJ-def2-SVP/J model chemistry including a frequency analysis that revealed no imaginary frequencies followed by a re-optimisation using RI-BP86-D3BJ-def2-TZVP/J. NBO analyses were then performed on the basis of these gas-phase structures with wavefunctions obtained from single point calculations using def2-SVP basis set on all atoms except for the carbon atoms of residues attached to the boron atom, the CH_2 group and the Sn atom for which def2-TZVP basis (with ECP-28 on the Sn-atom) was used. The energies discussed in the manuscript are those resulting from 2nd-order perturbation theory (SOPT) interactions of the σ-C_{H2}-Sn_{Me3} bond NBO into the empty (LV-) p-orbital at the boron atom.

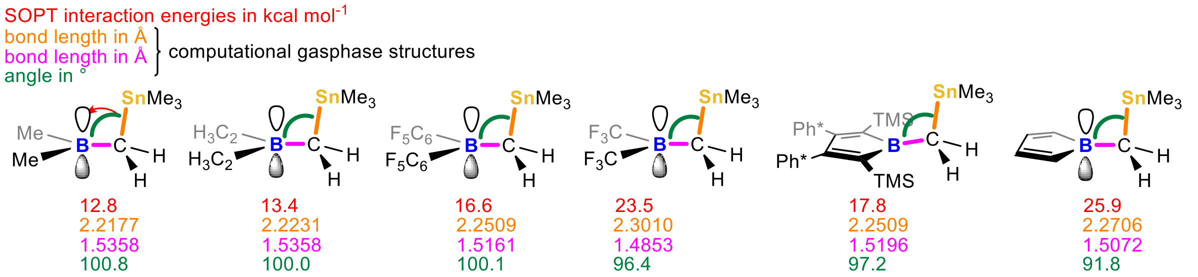

SI-FIGURE 11-SI: Summary of hyperconjugation interactions from SOPT and structural features in stannaneopentyl boranes.

Depictions of selected NBOs (at iso-surface values of 0.05 a.u.):

Compound 2 (anion)

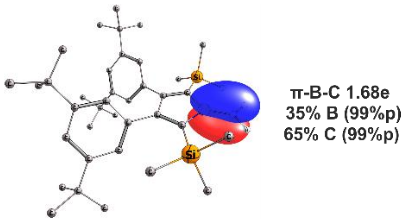

Compound 3

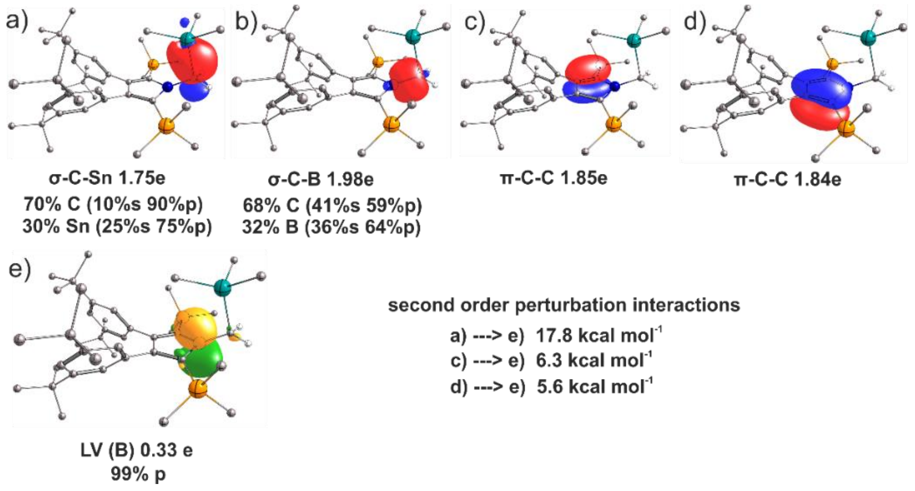

Compound 4 (anion)

The characteristics of the NBO bonding description do not differ when considering contact to a cation (see below).

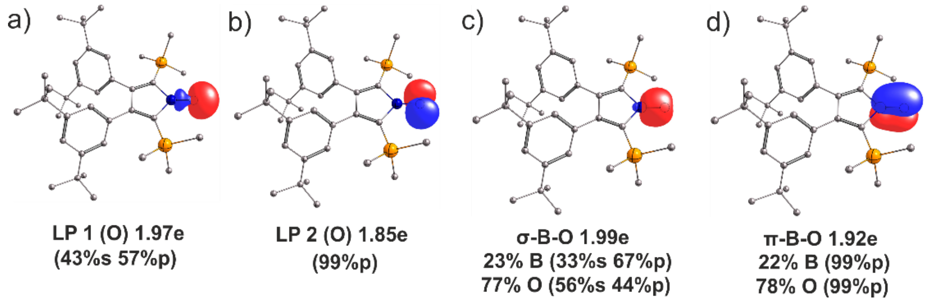

Compound 4-K(18-crown-6)

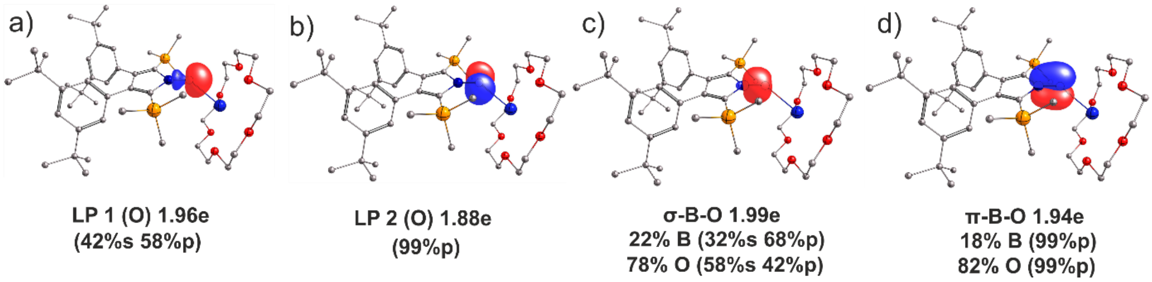

TD-DFT

TD-DFT calculations were performed in ORCA 4.2.1 using RIJCOSX-approximation and CAM-B3LYP functional[17] with def2-SVP/J basis set on all atoms. Single-point calculations were performed on the structures obtained from BP86 optimisation with def2-TZVP basis set as detailed above. For Sn an ECP-28 was applied.[16] Listed are the four absorptions of lowest wavenumbers and a depiction of the difference density plots (green: positive; magenta: negative) for the transition of lowest wavenumber.

Compound 1

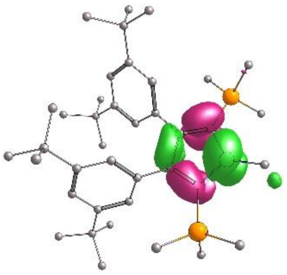

transition	energy (cm^{-1})	wavelength (nm)	f_{osc}
1	22397.3	446.5	0.008257153
2	30288.5	330.2	0.048262142
3	33860.7	295.3	0.104989926
4	34315.0	291.4	0.035692033

Transition 1 (isovalue 0.003 a.u.)

Compound 2

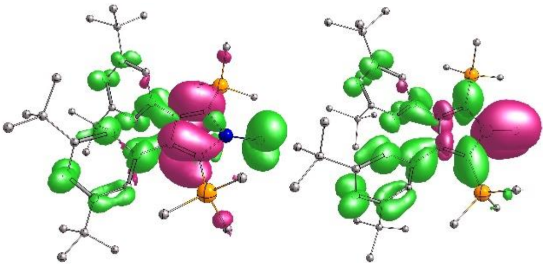

transition	energy (cm^{-1})	wavelength (nm)	f_{osc}
1	30506.0	327.8	0.034194984
2	30718.6	325.5	0.530285605
3	33548.8	298.1	0.115679146
4	34622.0	288.8	0.001839034

Transition 1 (left), **Transition 2** (right), (isovalues 0.001 a.u.)

Compound 3

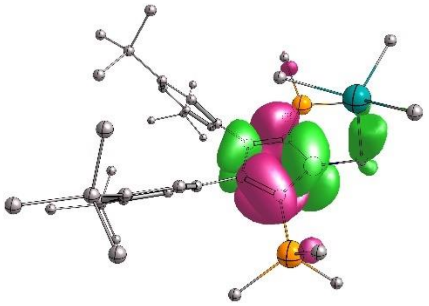

transition	energy (cm^{-1})	wavelength (nm)	f_{osc}
1	24705.8	404.8	0.015467454
2	32704.3	305.8	0.075517648
3	35352.6	282.9	0.129980102
4	36638.2	272.9	0.065544861

Transition 1 (isovalues 0.002 a.u.)

Compound 4

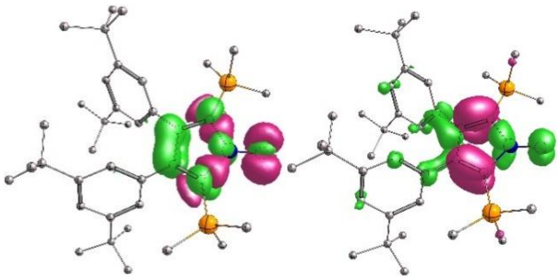

transition	energy (cm^{-1})	wavelength (nm)	f_{osc}
1	19142.0	***522.4***	0.002446902
2	31102.1	321.5	0.067381803
3	34684.2	288.3	0.008253314
4	36606.1	273.2	0.002219954

Transition 1 (left, isovalues 0.003 a.u.); **Transition 2** (right, isovalues 0.002 a.u.)

Compound [K(18-crown-6)]4

Consideration of counter cation contacts has significant influence on the wavelength of the computationally predicted absorption in the case of **4**.

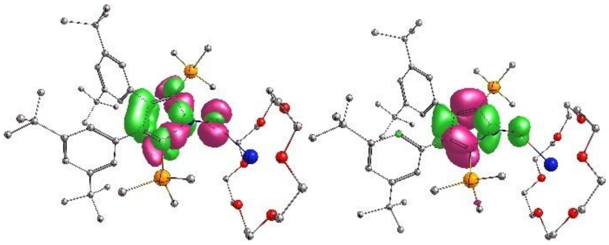

	energy (cm^{-1})	wavelength (nm)	f_{osc}
1	23330.0	***428.6***	0.002954382
2	30093.5	332.3	0.059551151
3	40020.9 .2	249.9	0.060579627
4	40081.6	249.5	0.139656394

Transition 1 (left, isovalues 0.003 a.u.); **Transition 2** (right, isovalues 0.002 a.u.)

Computational Approximations of pK_a values for α-CH bonds in boranes and boroles.

The computational approximation of pK_a values in DMSO of series of substituted methylboranes and methylboroles (and some reference compounds of experimentally known C-H acidities) was performed along the lines of a previous study on similar compounds by Erker and coworkers.[18] The approach of this previous study was to calculate the pK_a through computational approximation of the thermodynamics in deprotonation reactions of organoboranes (**BH**) with cyclopentadienyl anion (Cp^-) as a base and use the experimental pK_a of CpH (in DMSO = 18.0) as a reference.[18-19] Instead of applying TPSS-functional (as in Erker's study) we used BP86 and found only minor deviations from the reported computational predictions of directly comparable compounds. Our computational examination was performed using ORCA (version 4.2.1.).[11] All structures were optimised starting from (where available) experimental X-Ray structures on RI-BP86-D3BJ[12] def2SVP/J model chemistry[13] in the gas phase followed by a frequency calculation on the same level of theory. Thermochemical corrections were taken from these frequency calculations. For numerical accuracy, grid6 and finalgrid7 were applied. No imaginary frequencies were observed. All structures were then reoptimized using BP86-D3BJ-def2TZVP/J model chemistry. For the calculation of pK_a values, single-point calculations were then performed on these optimised structures using RI-BP86-D3BJ-def2TZVPP/J and (CPCM=DMSO) and thermochemical G_{corr} from gas-phase frequency calculations was then applied on this $E(SCF)_{DMSO}$ to access G_{DMSO} which was used for the pK_a calculations.

SI-Table 4-SI: Thermochemical data for compounds and reactions for pK_a approximations:

			RI-BP86-D3BJ-**def2TZVPP/J** (CPCM = DMSO) in Hartree	Thermochemical correction from RI-BP86-D3BJ-**def2SVP/J** (gasphase) at 298.15 K in Hartree	in Hartree	in kJ mol^{-1}		
**entry**	**acid pair**	**MOLECULE**	**$E(SCF)_{DMSO}$**	**G_{corr}**	**G_{DMSO}**	**ΔG_{DMSO}**	**pK_a(calc)**	**pK_a(ref)**
1	**A**	CpH	-194,1893869	0,062838452	-194,1265485	0.00	18.0	18.0 (exp)[19]
2		Cp^- (anion)	-193,7048908	0,049822797	-193,655068			
3	**B**	BMe_3	-144,6634144	0,080542937	-144,5828714	81.19	32.2	
4		Me_2BCH_2 (anion)	-144,1490255	0,068586285	-144,0804392			
5	**C**	Divinyl-B-Me	-220,8568929	0,089994983	-220,7668979	61.77	28.8	
6		Divinyl-B-CH_2^- (anion)	-220,3488761	0,077007413	-220,2718687			
7	**D**	Mes_2B-Me	-764,3147483	0,326632609	-763,9881157	62.50	29.0	29.2[18]
8		Mes_2B-CH_2^- (anion)	-763,8063483	0,313542645	-763,4928056			
9	**E**	„9-BBN-B-Me"	-378,2219762	0,204819566	-378,0171566	80.09	32.0	33.1[18]
10		„9-BBN-B-CH_2^- (anion) "	-377,7074452	0,192302874	-377,5151423			
11	**F**	Bis(1-TMS-2-Ph-vinyl)-B-Me	-1500,753011	0,41846393	-1500,334547	80.96	32.2	
12		Bis(1-TMSl-2-Ph-vinyl)-B-CH_2^- (anion)	-1500,238106	0,405906748	-1499,8322			
13	**G**	$(C_6F_5)_2B$-Bz	-1752,268908	0,164022061	-1752,104886	-37.18	11.5	11.6[18]
14		$(C_6F_5)_2B$-$CHPh^-$ (anion)	-1751,79762	0,150040188	-1751,64758			
15	**H**	$(C_6F_5)_2B$-Me	-1521,113428	0,08826407	-1521,025164	4.16	18.7	18.3[18]
16		$(C_6F_5)_2B$-CH_2^- (anion)	-1520,627846	0,07574688	-1520,5521			
17	**I**	Borole **1**	-2128,868948	0,813477752	-2128,05547	26.30	22.6	
18		Boratafulvene **2** (anion)	-2128,375908	0,801946479	-2127,573962			
19	**J**	Borol $(HC)_4B$-Me	-219,6381845	0,071835071	-219,5663494	3.18	18.6	
20		Borol $(HC)_4B$-CH_2^- (anion)	-219,1543757	0,060718565	-219,0936571			
21	**K**	Borol $(MeC)_4B$-Me	-376,9875782	0,170978431	-376,8165998	38.99	24.8	
22		Borol $(MeC)_4B$-CH_2^- (anion)	-376,4890389	0,158785561	-376,3302534			
23	**L**	Borol $(PhC)_4B$-Me	-1144,306433	0,366474302	-1143,939959	4.49	18.8	
24		Borol $(PhC)_4B$-CH_2^- (anion)	-1143,821741	0,354975306	-1143,466765			
25	**M**	Borol $([C_6F_5]C)_4B$-Me	-3129,886584	0,187010945	-3129,699573	-39.42	11.1	
26		Borol $([C_6F_5]C)_4B$-CH_2^- (anion)	-3129,418583	0,175462046	-3129,243121			
27	**N**	Borol $(F_3CC)_4B$-Me	-1568,45563	0,072811739	-1568,382818	-64.48	6.7	
28		Borol $(F_3CC)_4B$-CH_2^- (anion)	-1567,999278	0,063359626	-1567,935919			
29	**O**	„Benzoborole" Boraindene-B-Me	-373,3806972	0,115426394	-373,2652708	21.58	21.8	
30		„Benzoborole" Boraindene-B-CH_2^- (anion)	-372,8893084	0,103744939	-372,7855634			
31	**P**	„Dibenzoborole" Borafluorene-B-Me	-527,1164021	0,158819712	-526,9575824	32.29	23.7	
32		„Dibenzoborole" Borafluorene-B-CH_2^- (anion)	-526,6205147	0,146725079	-526,4737897			
33	**Q**	Toluene	-271,6860505	0,095080858	-271,5909697	128.25	40.5	41.7[18]; 41 (exp)[20]
34		Benzyl-Anion	-271,1502301	0,079636704	-271,0705934			
35	**R**	Pentane	-197,8519699	0,125288529	-197,7266814	226.43	57.7	
36		1-Pentyl-Anion	-197,2763785	0,107501381	-197,1688772			
37	**S**	Cyclopentane	-196,6393734	0,107453079	-196,5319204	233.53	58.9	60.4[18]; 58 (exp)[18-19]
38		Cyclopentyl-Anion	-196,0619662	0,090558043	-195,9714082			
39	**T**	*N,N*-Dimethylthioacetamide	-610,9557914	0,09215058	-610,8636408		28.4	25.7[21]
0		*N,N*-Dimethylthioacetamide Anion	-610,4481573	0,078640006	-610,3695173	59.39		
41	**U**	Dimedon	-462,7661636	0,146588048	-462,6195756		9.2	11.2[22]
42		Dimedon-Anion	-462,3016888	0,134413494	-462,1672754	-50.31		
43	**V**	Meldrum's acid	-534,6553455	0,100750502	-534,554595		8.5	7.3[23]
44		Meldrum's acid Anion	-534,192364	0,088618211	-534,1037458	-54.12		
45	**W**	Phenylmalononitrile	-456,2407385	0,086549034	-456,1541894		5.4	4.2[24]
46		Phenylmalononitrile-Anion	-455,7857113	0,075645759	-455,7100656	-71.76		
47	**X**	B-Methyl-3-Borolen	-220,8815348	0,092453392	-220,7890814		31.7	
48		B-Methyl-3-Borolen-Anion	-220,3682208	0,080383298	-220,2878375	78.07		
49	**Y**	B-Methyl-2-Borolen	-220,8895429	0,09368186	-220,7958611		32.6	
50		B-Methyl-3-Borolen-Anion	-220,374071	0,081421113	-220,2926499	83.23		

ΔG_{DMSO} for the deprotonation reactions was calculated for the following reaction

$$(Acid - CH) + Cp^{-} \rightarrow (Acid - C)^{-} + Cp - H$$

pK_a(calc) was then calculated using the experimental pK_a of CpH in DMSO (18.0) as a reference according to

$$pK_a(calc) = 18.0 + \frac{\Delta G_{DMSO}}{\ln(10)RT} \qquad \text{with R = 8.3145 J K}^{-1} \text{ and T = 298.15 K}$$

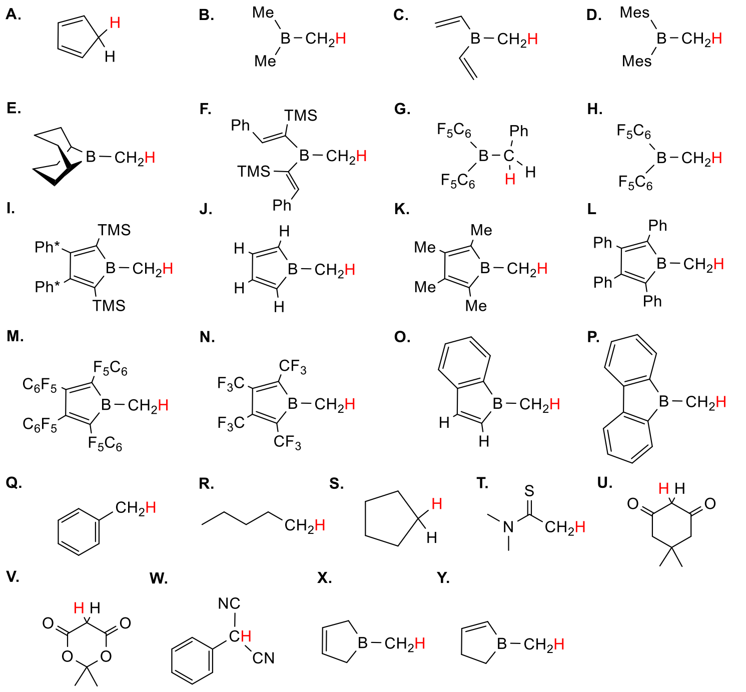

SI-Figure 12-SI: Structure depictions of the computationally considered compounds as listed in **SI-Table 4-SI**

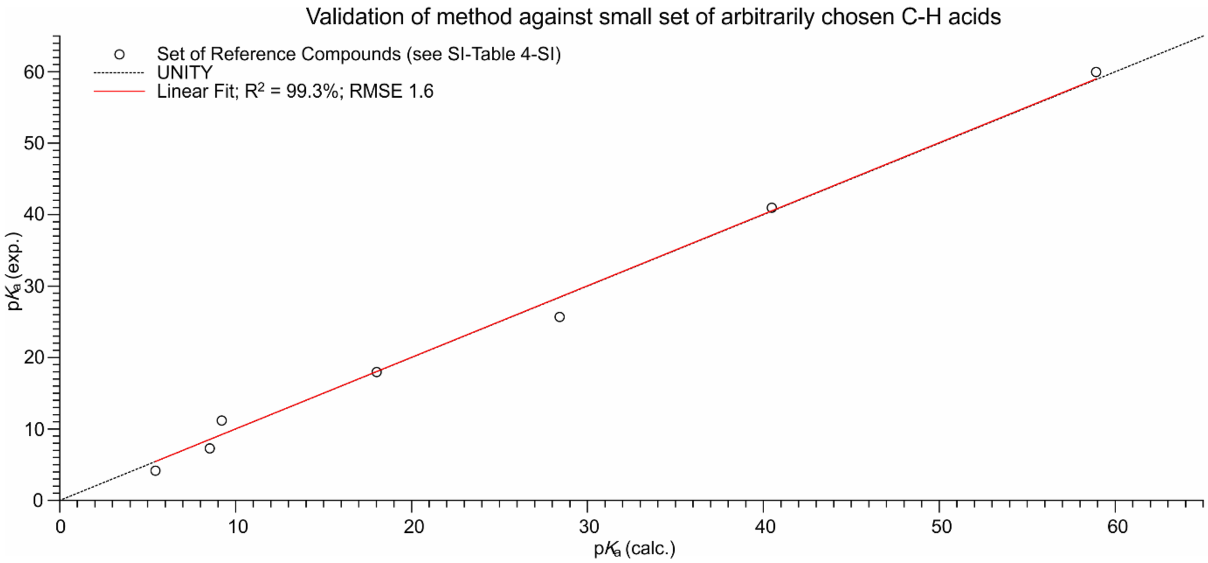

SI-Figure 13-SI: Plot of computational pK_a values for a set of arbitrarily chosen CH-acids vs. experimental pK_a.

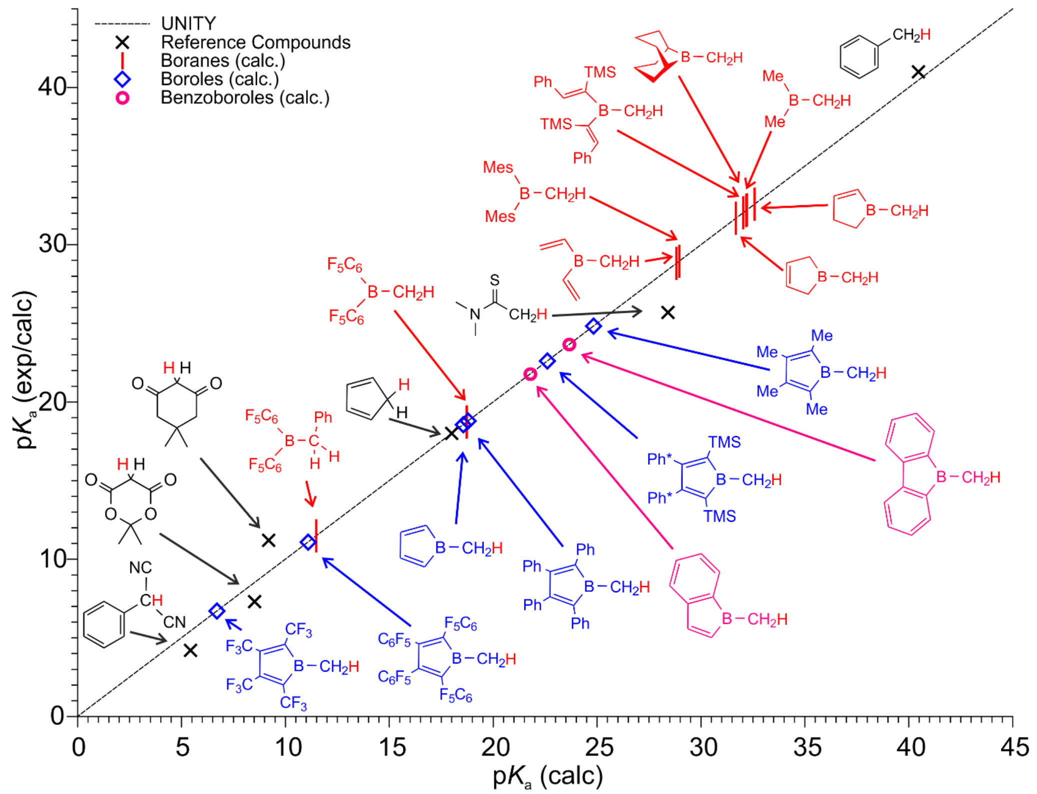

SI-Figure 13-SI: Plot of computational pK_a values of assigned structures under investigation in this study..

NICS$_{zz}$-Profile

NICS$_{zz}$ profiles[25] were calculated with the GIAO method and RIJK-PBE0[26] functional and def2-TZVP basis set on gas phase structures previously optimised using the RI-BP86-D3BJ-def2TZVP/J model chemistry. NICS values were calculated for a series of points along a line orthogonal to the borole plane on both sides dissecting it at its centroid. For the structure of compound **3** due to the trimethylstannyl group, no mirror symmetry through the borole plane can be assumed. The trimethylstannyl group coincides with the NICS scan axis on one side seriously affecting the resulting values. As to be expected, mirror symmetry is reflected in the NICS profiles above and below the ring plane. Therefore, for comparisons only the profile through the half-sphere over the borole plane in **3** not affected by the trimethylstannyl group is presented. Both, profiles of **1** and **3** are in line with behaviour expected for anti-aromatic compounds.[27]

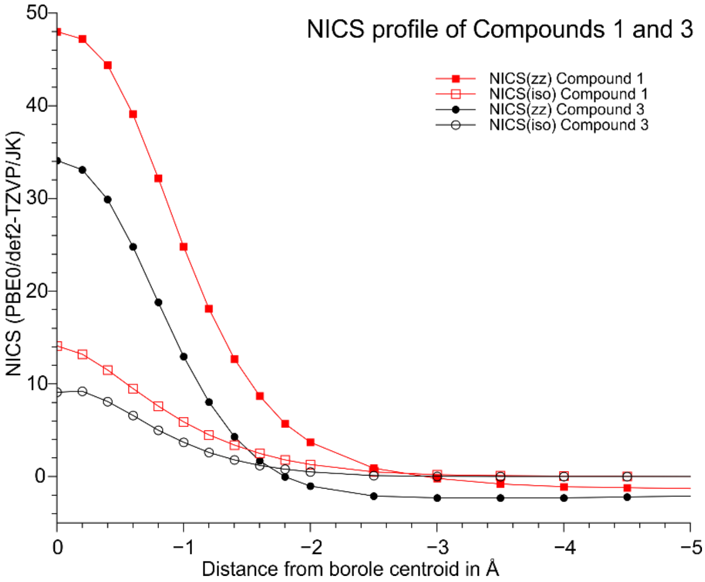

SI-Figure 14-SI: NICS profiles of the half-spheres not affected by organic substituents through compounds **1** and **3**.

XYZ-coordinates of optimised structures

All structures optimised at RI-BP86-D3BJ-def2TZVP/J level of theory in the gas phase (see above).

Molecular structure of Fulvene

C	3.193835405	10.128400651	15.031318563
C	0.836089235	10.212242849	15.101909784
C	1.259545458	9.100933958	15.759788777
C	2.732648991	9.048555261	15.715691841
C	2.027903482	10.922306504	14.605712711
C	2.047687608	12.071610640	13.901425236
H	2.986694451	12.522884796	13.576771218
H	1.125357471	12.589073475	13.632497105
H	-0.187669132	10.545514902	14.950994657
H	0.629669949	8.358538799	16.246519601
H	3.336206282	8.262304302	16.165501426
H	4.227813244	10.388504014	14.818802413

Molecular structure of Benzene

C	-0.577489795	-0.000000002	-5.454049167
C	-1.734697638	-0.355041424	-4.755196521
C	0.579718046	0.355041426	-4.755196521
H	-2.638041919	-0.632191631	-5.300736717
H	1.483062328	0.632191635	-5.300736716
C	-1.734697639	-0.355041425	-3.357491454
C	0.579718046	0.355041426	-3.357491455
H	-2.638041921	-0.632191632	-2.811951260
H	1.483062328	0.632191637	-2.811951261
C	-0.577489795	-0.000000004	-2.658638809
H	-0.577489795	-0.000000003	-1.567558037
H	-0.577489795	-0.000000004	-6.545129939

Molecular structure of Boratafulvene (anion)

C	3.249249394	10.084053188	15.055475337
C	0.779063346	10.171779661	15.129478863
C	1.257918551	9.067530816	15.780384501
C	2.733138270	9.015119728	15.736167969
B	2.028789107	10.981332297	14.569583093
C	2.050437187	12.230301880	13.804289776
H	2.974177958	12.716853535	13.458815215
H	1.144898265	12.782326658	13.513363379
H	-0.293571316	10.383912746	15.055113619
H	0.659595674	8.295872495	16.283449438
H	3.304250706	8.201914867	16.204194232
H	4.327835304	10.219872283	14.916617911

Molecular structure of Boratabenzene (anion)

C	-1.148148981	-0.000039386	-3.380358631
C	-2.335795841	-0.364418402	-2.730571700
C	0.013233793	0.356283152	-2.670328606
H	-3.185719478	-0.625181263	-3.379108260
H	0.920222520	0.634552951	-3.218070366
B	-2.396862808	-0.383154991	-1.214840772
C	0.020400792	0.358481295	-1.263252961
H	-3.410780389	-0.694228224	-0.602523861
H	0.955835014	0.645477888	-0.759114837
C	-1.111403158	0.011234025	-0.512293687
H	-0.999419219	0.045592068	0.581893839
H	-1.097824843	0.015400889	-4.479807835

Molecular structure of 1

Si	-8.746436665	-2.217242299	-11.258355042
C	-5.699595185	-0.167626894	-11.647375456
H	-5.017097515	0.447452497	-11.041538940
H	-5.296719605	-0.104007670	-12.676104067
H	-6.690076093	0.304724622	-11.649975639
B	-5.685658198	-1.663868827	-11.194921036
Si	-2.580703836	-2.101317481	-11.000525197
C	-4.394842912	-2.548275001	-10.868553795
C	-4.901647621	-3.681508406	-10.300457629
C	-6.440510530	-3.674421568	-10.270192073
C	-6.955322429	-2.559528687	-10.856511387
C	-1.905380755	-2.657432952	-12.678064067
H	-1.946389623	-3.749775686	-12.792636463
H	-2.487982598	-2.215656386	-13.500048186
H	-0.857183269	-2.344610996	-12.801356920
C	-2.356038728	-0.226313588	-10.905862213
H	-1.281410278	0.012317465	-10.913171651
H	-2.817649784	0.293781225	-11.756089399
H	-2.781236889	0.188759455	-9.980251834
C	-1.529185975	-2.832019331	-9.611653972
H	-1.970290461	-2.602994805	-8.630361409
H	-1.425160145	-3.921790152	-9.679355476
H	-0.522449996	-2.387229328	-9.641012467
C	-8.820654032	-1.435808210	-12.979057983
H	-8.276445839	-0.483143076	-13.031588612
H	-8.385225211	-2.111447239	-13.730424046
H	-9.865022170	-1.243165806	-13.268001780
C	-9.808535598	-3.775808489	-11.321613136
H	-9.977710936	-4.215410868	-10.330974068
H	-10.788185854	-3.535361665	-11.762776063
H	-9.335755307	-4.546708678	-11.947437820
C	-9.473092039	-1.004821737	-10.002810154
H	-9.488452743	-1.445621672	-8.994985717
H	-8.879259093	-0.080444435	-9.952031117
H	-10.505820729	-0.730692749	-10.266754697
C	-4.180192243	-4.829372345	-9.731680702
C	-3.260398649	-5.555015910	-10.502422960
H	-3.083244012	-5.233258580	-11.526789624
C	-2.603286996	-6.673429431	-9.978974023
C	-1.578760620	-7.479389150	-10.787290105
C	-1.381594795	-6.913434864	-12.201367011
H	-1.008866600	-5.879328721	-12.176227028
H	-0.642164376	-7.520878865	-12.742533730
H	-2.315717396	-6.929692051	-12.781370813
C	-0.220040814	-7.438623859	-10.055344585
H	-0.290539869	-7.870819438	-9.047830346
H	0.534035676	-8.009863764	-10.617557776
H	0.136315365	-6.403515969	-9.953350020
C	-2.054568796	-8.942130061	-10.908623873
H	-2.188746489	-9.407754345	-9.922696011
H	-3.015775208	-8.996781715	-11.438632728
H	-1.317569503	-9.538404956	-11.467418173
C	-2.900634075	-7.053021500	-8.662374411
H	-2.404065691	-7.931091673	-8.245716228
C	-3.821867518	-6.358463018	-7.865074294
C	-4.131425550	-6.845896506	-6.444375260
C	-2.833994053	-6.854621774	-5.610574457
H	-3.039096098	-7.203394224	-4.587204297
H	-2.076021106	-7.519076442	-6.048073539
H	-2.402751104	-5.845000215	-5.551775196
C	-4.713187337	-8.273274388	-6.516904437
H	-4.913679971	-8.655954410	-5.504744975
H	-5.659372598	-8.270418249	-7.076633997
H	-4.024516618	-8.971275842	-7.012853192
C	-5.166422431	-5.953174210	-5.744015249
H	-5.369524853	-6.344887883	-4.736789098
H	-4.809626646	-4.918769287	-5.635529050
H	-6.115984408	-5.934656503	-6.297812795
C	-4.455137718	-5.246183384	-8.419695902
H	-5.184041247	-4.684515640	-7.840650296
C	-7.163174164	-4.794348517	-9.648689308
C	-8.029509899	-4.567040506	-8.572686754
H	-8.180163668	-3.537972889	-8.243585164
C	-8.662995756	-5.627826924	-7.914243857
C	-9.621309268	-5.329835783	-6.753207991
C	-10.813887608	-4.514965180	-7.299377727
H	-11.521395321	-4.280052203	-6.489793061
H	-10.481779702	-3.567184755	-7.745292549
H	-11.351233617	-5.080677908	-8.074079389
C	-8.894257167	-4.504465959	-5.671105225
H	-8.047168251	-5.065792876	-5.252764156
H	-8.506430585	-3.558510532	-6.072292595
H	-9.585636942	-4.263334232	-4.849776711
C	-10.162068747	-6.610195793	-6.099550762
H	-10.829214164	-6.345078216	-5.266879619
H	-10.741989705	-7.217860197	-6.809049966
H	-9.350667459	-7.232229212	-5.694224948
C	-8.411052512	-6.930103406	-8.370018406
H	-8.888195066	-7.764980184	-7.863282104
C	-7.564236837	-7.192908201	-9.459177697
C	-7.340030971	-8.608278835	-10.006805716
C	-8.102184283	-8.729201002	-11.345020554
H	-9.178889260	-8.561372907	-11.198306058
H	-7.741406656	-7.987533863	-12.071396293
H	-7.962846552	-9.731505467	-11.778288057
C	-7.854286691	-9.692397037	-9.046752709
H	-7.369542999	-9.624413883	-8.061941819
H	-8.942095979	-9.630447104	-8.900572428
H	-7.634156647	-10.686315376	-9.461858929
C	-5.837910176	-8.854606732	-10.256716671
H	-5.253987858	-8.729861544	-9.335830510
H	-5.682043724	-9.877177163	-10.631426977
H	-5.425084653	-8.161093227	-11.000670034
C	-6.939242216	-6.109208847	-10.079115124
H	-6.259254909	-6.272138949	-10.914363440

Molecular structure of 2 (anion)

Si	5.024920072	10.630509096	14.981131943
C	3.261888811	10.089795609	15.108698096
C	5.177251595	12.416690909	15.602006216
H	4.457910811	13.068789165	15.088580580
H	6.195243385	12.812093654	15.452919835
H	4.947109066	12.460280315	16.677722857
C	0.745034127	10.149878857	15.150206128
C	1.249256080	9.039832421	15.799847559
Si	-1.052812275	10.498302950	14.873585954
C	2.735552440	8.991843944	15.757842873
C	6.301998139	9.654392302	15.999665624
H	6.523355306	8.667270470	15.572554478
H	5.951192468	9.489789589	17.029036607
H	7.243643915	10.225880768	16.045119831
C	5.600492355	10.585858594	13.172849957
H	4.909569681	11.168414123	12.546246506
H	5.598325153	9.552431364	12.792933873
H	6.617077244	10.993514135	13.051777369

C	-1.344081387	11.525226752	13.306951139
H	-0.834939952	11.075342700	12.442779477
H	-0.960154469	12.548506562	13.419354457
H	-2.422001882	11.586070943	13.085800599
C	-1.799704205	11.507846356	16.303511098
H	-1.218698713	12.430202917	16.453137386
H	-1.777811299	10.945678699	17.248787317
H	-2.846065422	11.787339000	16.099774404
C	-2.116081350	8.937290940	14.652747440
H	-3.146791525	9.213955308	14.377118603
H	-2.155291860	8.321055903	15.560351401
H	-1.705638937	8.309780516	13.847049522
B	2.014364530	10.952019911	14.615032316
C	4.376414720	7.098844522	15.653306853
H	4.554060087	7.382669734	14.616999137
C	3.459816520	7.870102020	16.384043611
C	5.027292613	5.999166047	16.222123728
C	6.066221472	5.172721735	15.450947536
C	6.229818234	5.652890857	14.000750319
H	6.980278065	5.032317870	13.487807801
H	6.567972469	6.697722480	13.954450468
H	5.286569455	5.576182952	13.441135685
C	7.430933197	5.303541816	16.161309631
H	8.202790119	4.725627607	15.627761697
H	7.378532281	4.934085865	17.195048561
H	7.746474477	6.356149922	16.197678235
C	5.643134210	3.689441028	15.422870266
H	4.676387373	3.571789539	14.913334502
H	5.536961784	3.279939641	16.436856130
H	6.393649103	3.085219012	14.888498919
C	4.717524377	5.657666433	17.546522949
H	5.201569558	4.788195763	17.996606987
C	3.793155709	6.391403622	18.305748624
C	3.445854514	5.944328397	19.732768407
C	4.726118383	5.893721344	20.590826863
H	5.463304673	5.192641023	20.174838979
H	4.490360148	5.570255059	21.617328435
H	5.197562197	6.886057194	20.639332244
C	2.802946371	4.542409156	19.677527659
H	1.869097704	4.579294751	19.098366716
H	2.566366963	4.185492159	20.692843852
H	3.470800864	3.809201020	19.203572764
C	2.439529948	6.891164211	20.403787940
H	2.835892717	7.913090732	20.489919626
H	2.210269592	6.528919171	21.417746478
H	1.499189049	6.935934888	19.836701736
C	3.188001953	7.499259189	17.712341095
H	2.464475353	8.094374242	18.264552065
C	0.498315714	7.940445695	16.437831738
C	0.691465834	6.617700886	16.011560851
H	1.360698990	6.452411521	15.167255152
C	0.077633606	5.537596014	16.647134433
C	0.316445903	4.118698726	16.111757438
C	-0.394009599	3.989021201	14.746743761
H	-0.010019214	4.731957654	14.034331273
H	-1.475848001	4.156214701	14.854201450
H	-0.235779373	2.985132091	14.320363000
C	1.827127016	3.871228306	15.920876671
H	1.999661034	2.852864053	15.537990583
H	2.371975483	3.985587754	16.866709809
H	2.270838722	4.580444699	15.210777202
C	-0.232920642	3.036652153	17.054510145
H	-1.324324146	3.109397538	17.169672206
H	0.223346439	3.103396260	18.053079184
H	-0.006864524	2.040353212	16.645412291
C	-0.768499072	5.802135749	17.736592215
H	-1.249220678	4.971888242	18.248220763
C	-1.010752497	7.112001745	18.178286521
C	-1.987086124	7.423971482	19.322179201
C	-1.308338512	8.322816941	20.376141890
H	-0.442647504	7.814668375	20.824019282
H	-2.019097760	8.569396477	21.180729601
H	-0.954480252	9.265196391	19.938427895
C	-3.204765258	8.166966140	18.730661724
H	-2.899238517	9.099263617	18.236799720
H	-3.929950649	8.416363664	19.522075762
H	-3.710240448	7.543737016	17.978676962
C	-2.485183315	6.154455856	20.030117863
H	-3.046732656	5.500625312	19.347149682
H	-3.158030370	6.431129319	20.855899692
H	-1.650569327	5.575128293	20.452009542
C	-0.362214895	8.164760510	17.521070870
H	-0.498694453	9.194978055	17.851968123
C	2.052626848	12.172418848	13.803656204
H	2.982948040	12.648466483	13.469816198
H	1.165224353	12.700282534	13.440148608

Molecular structure of B (anion)

C	12.403805026	5.647556134	14.478947175
H	12.438503611	5.941044893	15.540740667
H	11.533393671	6.150057180	14.037398044
H	13.301766348	6.067753848	14.000126090
B	12.336665612	4.000099358	14.403511778
N	12.259942365	3.342264379	12.950705966
Si	15.401701883	3.923162911	14.926978747
Si	9.297524851	3.778135679	15.039885932
C	13.630770124	3.526893703	15.320858286
Si	12.442261386	1.608583793	12.882762797
C	13.142893566	3.046743343	16.503696570
Si	12.065383064	4.156684126	11.434488108
C	11.647681004	3.018660319	16.530556664
C	11.102024238	3.522102582	15.384461630
C	15.927942550	5.573868302	15.715515354
H	16.930142295	5.866205278	15.361968300
H	15.956453675	5.533271271	16.812107144
H	15.220869237	6.366304355	15.430223584
C	8.584200390	2.375008617	13.979991373
H	7.538175670	2.582636344	13.704054670
H	8.609329685	1.407369209	14.502712135
H	9.168827428	2.270837329	13.055243206
C	15.655402858	4.221617227	13.077261232
H	15.448035986	3.331011279	12.474902492
H	16.693296742	4.537641946	12.885411009
H	14.985456730	5.019005992	12.727144521
C	16.639309620	2.594915683	15.481370510
H	16.419258873	1.626720697	15.008121227
H	16.604715654	2.445924387	16.569484285
H	17.665973409	2.881334434	15.201895398
C	9.013126601	5.374174783	14.058807094
H	9.617399540	5.408689995	13.143902336
H	9.271773023	6.256591531	14.661986045
H	7.952219663	5.455420171	13.772694265
C	11.867083597	0.622292243	14.385878633
H	10.799923384	0.764820062	14.589136558
H	12.430561589	0.880928148	15.288207319
H	12.035465483	-0.442638095	14.155695842
C	8.204261853	3.975759695	16.580887777
H	7.198774376	4.316139529	16.284373719
H	8.634150011	4.731897912	17.255069726
H	8.098839461	3.048369477	17.156905251
C	13.303640777	3.537659118	10.126071904
H	13.085023569	4.013532307	9.156251172
H	13.289047565	2.450706811	9.973019461
H	14.326576035	3.819456388	10.416507506
C	11.436782070	0.805429770	11.475405194
H	11.674209324	1.168082630	10.467172278
H	10.356995012	0.938396814	11.637053741
H	11.641159346	-0.277445038	11.494732078
C	14.225173837	1.020253787	12.608433461
H	14.237731807	-0.078024530	12.517714593
H	14.862887707	1.290957066	13.460404628
H	14.674693501	1.439328181	11.697614447
C	10.335119716	3.939890002	10.673050580
H	10.251994936	4.530823620	9.746145987
H	9.551750873	4.286772575	11.361682371
H	10.115937356	2.893243449	10.424968524
C	12.340611678	6.029501736	11.405396964
H	12.284776927	6.337620970	10.348174088
H	13.325504987	6.323574394	11.790486097
H	11.580441714	6.591195210	11.961994332
C	13.910136064	2.512966262	17.655074759
C	13.711341015	1.184608726	18.046009040
H	12.988005013	0.589602121	17.487374130
C	14.393463999	0.619915045	19.129628319
C	14.092486720	-0.832654391	19.519264578
C	14.401782744	-1.755747606	18.321799907
H	13.800832089	-1.485584974	17.443500824
H	14.180769276	-2.804167859	18.577666341
H	15.461921352	-1.682511878	18.038269531
C	12.596302015	-0.949403246	19.879042375
H	11.955232134	-0.618162206	19.052517515
H	12.350890048	-0.319113582	20.745421791
H	12.338455113	-1.992237685	20.124108297
C	14.922193296	-1.301368474	20.723386953
H	14.667722813	-2.344370013	20.964786334
H	14.719034752	-0.691650370	21.616227204
H	16.001605177	-1.257688528	20.515043333
C	15.306231222	1.421009028	19.822053141
H	15.853205343	1.000947299	20.665557776
C	15.528946716	2.764249435	19.470665823
C	16.556040202	3.584073381	20.264426367
C	16.632249391	5.041686741	19.787530627
H	16.951052163	5.108714674	18.738174520
H	17.364296504	5.592984079	20.396879497
H	15.662063760	5.550350018	19.882439979
C	16.184746944	3.585964655	21.762026818
H	15.205235686	4.059283510	21.918126436
H	16.936066396	4.144890112	22.342199178
H	16.130812791	2.566671241	22.168239741
C	17.949178594	2.943004743	20.083263339
H	18.230880229	2.928577453	19.020616404
H	17.962499322	1.906520156	20.448267452
H	18.711872571	3.512568602	20.638144311
C	14.812480534	3.295747782	18.395183352
H	14.933081455	4.336912234	18.105094927
C	10.935979090	2.456932841	17.698225352
C	10.056628963	1.378018188	17.537330515
H	9.895567386	1.004360702	16.526126666
C	9.420325729	0.772478666	18.625923462
C	8.469364179	-0.405611923	18.373036046
C	7.285621379	0.082821084	17.511566239
H	7.630855081	0.491106208	16.552232745

H 6.729396017 0.877354312 18.030027009
H 6.592111138 -0.746930415 17.301065372
C 9.221996403 -1.521153844 17.616626495
H 8.544823878 -2.363100817 17.402062254
H 10.062479768 -1.898126407 18.216928105
H 9.628074029 -1.159229606 16.662809680
C 7.913055251 -0.999125831 19.675835079
H 7.248041934 -1.844473842 19.443165849
H 7.329258360 -0.258986369 20.242578312
H 8.718796398 -1.372078724 20.325214707
C 9.687895182 1.268087548 19.909975330
H 9.216892674 0.797724985 20.769599771
C 10.569610222 2.340829959 20.113810151
C 10.907260818 2.880031104 21.510205895
C 12.431965620 2.791873695 21.729445181
H 12.777471749 1.749788550 21.681043614
H 12.704298201 3.202588802 22.714727931
H 12.985482228 3.343883377 20.959335271
C 10.463847388 4.355502670 21.602589539
H 10.963747511 4.968094002 20.840294099
H 10.709226164 4.771733548 22.592793823
H 9.378880543 4.445340888 21.445990754
C 10.211071360 2.091674255 22.629487208
H 9.115528047 2.146182429 22.546076080
H 10.495095349 2.508742345 23.607451958
H 10.503997389 1.031454169 22.618210994
C 11.177784654 2.921995157 18.998297383
H 11.878807603 3.747897502 19.123942345

Molecular structure of HMDS (anion)

N -0.093014203 -1.940848849 -0.333794886
Si 1.460602660 -2.335751134 0.081950470
Si -1.170088354 -0.687043510 -0.232028105
C 1.841494609 -2.294022136 1.962447035
H 2.870503423 -2.615748242 2.199647542
H 1.704605033 -1.273901247 2.356169007
H 1.142691191 -2.950210661 2.505404869
C 1.904149687 -4.113949484 -0.464472012
H 1.793083082 -4.213620961 -1.556027449
H 2.936249283 -4.396260128 -0.195854363
H 1.216766776 -4.836539268 0.003644769
C 2.823711702 -1.231909994 -0.696921061
H 3.844490489 -1.563349638 -0.438465993
H 2.730227718 -1.231837071 -1.794632178
H 2.708815022 -0.189273086 -0.359102088
C -0.631621951 0.929933957 -1.114410303
H 0.289235165 1.325593291 -0.656288507
H -0.408284324 0.727188042 -2.173997531
H -1.401135607 1.720192755 -1.069950828
C -1.608216913 -0.124890574 1.549891300
H -2.370002217 0.673721141 1.573234778
H -1.987345379 -0.978415871 2.134238621
H -0.707826533 0.248761142 2.063794896
C -2.850375631 -1.137581391 -1.026370592
H -3.282625065 -2.018003573 -0.524593418
H -3.582295062 -0.314139233 -0.968743233
H -2.709694735 -1.399395909 -2.087231016

Molecular structure of H-HMDS

N -0.174186000 -2.087186185 -0.423868176
Si 1.506486464 -2.321575297 -0.019167675
Si -1.229471526 -0.725925358 -0.146859804
C 1.842353807 -1.630273979 1.700155011
H 2.899680790 -1.764393722 1.974245507
H 1.624956895 -0.553287184 1.755897500
H 1.226635185 -2.137342654 2.456879423
C 1.846481974 -4.172798159 -0.057034041
H 1.635391418 -4.596574013 -1.050871866
H 2.901081027 -4.385433648 0.173047919
H 1.226282660 -4.706146331 0.678233561
C 2.642704195 -1.454028851 -1.246891075
H 3.703147617 -1.616208680 -0.999725240
H 2.471955076 -1.825475860 -2.268280823
H 2.461495976 -0.369232418 -1.253109174
C -0.297045544 0.874636154 -0.487317274
H 0.578696778 0.983667649 0.169564710
H 0.056287686 0.912175256 -1.527810716
H -0.944940081 1.747319426 -0.314895178
C -1.858676397 -0.686836373 1.628932162
H -2.537311233 0.163369577 1.797880989
H -2.405302977 -1.610370491 1.871066266
H -1.025092753 -0.597566136 2.340831542
C -2.690897255 -0.893083128 -1.321743922
H -3.235713235 -1.833871719 -1.148746793
H -3.406265403 -0.069093883 -1.181678511
H -2.361471514 -0.879375903 -2.371040800
H -0.610458319 -2.873366563 -0.901742560

Molecular structure of 3

Sn 10.492596776 5.191810236 1.391422093
C 10.669156303 4.251751303 3.428947130
H 9.808920165 3.575149555 3.437174708
H 11.616934336 3.705889018 3.354719173
B 10.636039728 5.538787337 4.236195437
C 9.351295200 6.434770582 4.559749606
C 9.866020107 7.692660661 4.715990305
C 11.388180597 7.712956532 4.656720047
C 11.908648590 6.463451160 4.488481737
Si 13.695223786 5.949799770 4.619444601
C 10.488349253 7.351332457 1.291419238
H 10.573583749 7.649916695 0.238597135
H 11.331452037 7.757161931 1.860022003
H 9.553044409 7.737677807 1.709789686
C 8.652194919 4.471869904 0.496293807
H 7.783221029 4.984816402 0.923505596
H 8.546570393 3.390995577 0.650818925
H 8.691478230 4.675647707 -0.582162765
C 12.143160653 4.427954847 0.207354736
H 11.837986144 4.409791174 -0.847261724
H 12.399383977 3.408900530 0.522001297
H 13.023131201 5.071756779 0.316001238
C 14.753059456 7.183953928 5.577038367
H 15.724316702 6.726481201 5.820922616
H 14.264290492 7.465605744 6.521050454
H 14.940159894 8.107495301 5.015410240
C 13.745589189 4.312195546 5.562587379
H 13.155615770 3.532251293 5.061042068
H 13.336116205 4.435967551 6.576159629
H 14.779195351 3.946811826 5.659300683
Si 7.526808126 6.045092641 4.393048574
C 7.271917740 4.224075106 3.958802191
H 7.718618865 3.569735976 4.721968966
H 7.693436232 3.947926570 2.984394131
H 6.193654631 4.005664628 3.923844789
C 6.558797466 6.318533337 5.996309677
H 5.608471086 5.764335319 5.957431208
H 6.322662082 7.380336876 6.141916137
H 7.118315000 5.967397685 6.875485604
C 6.753425509 7.118937883 3.043934606
H 5.683576868 6.890868716 2.921955388
H 7.243531617 6.960911686 2.072194980
H 6.850302158 8.184839927 3.294926966
C 14.450766391 5.662620235 2.909367364
H 13.949297511 4.833293262 2.391332255
H 15.518526402 5.408368074 2.990551634
H 14.365445825 6.555547348 2.272777489
C 12.113866927 8.992607000 4.700602291
C 14.612371857 10.856050141 2.464383039
C 13.840093962 10.741630178 1.132498079
H 14.510826464 10.942013328 0.283354564
H 13.014101320 11.466305507 1.097693326
H 13.413202285 9.738591391 0.996601211
C 15.211941305 12.266910310 2.555739891
H 15.881320976 12.439330485 1.700725509
H 15.802487998 12.399843007 3.473738754
H 14.431693704 13.041489132 2.531576226
C 15.765746260 9.830482617 2.477005005
H 15.392301717 8.801923617 2.379007432
H 16.333359656 9.892961692 3.416609286
H 16.457786162 10.020414991 1.642650543
C 13.660285655 10.550699475 3.627517886
C 13.008434319 9.314210702 3.671693942
H 13.169947564 8.576215939 2.884825831
C 13.401586903 11.466356510 4.656959496
H 13.890243740 12.436860240 4.632745122
C 12.516493537 11.178275291 5.706986462
C 12.220269601 12.173525759 6.835456105
C 12.929017620 13.520326924 6.629730517
H 14.023056976 13.409775345 6.617570659
H 12.672489812 14.201152106 7.454049664
H 12.620239081 14.001639858 5.690159986
C 12.695401472 11.565972584 8.172741419
H 13.777146663 11.370208490 8.150034896
H 12.185517561 10.615688362 8.382597959
H 12.485533819 12.256465286 9.003774088
C 10.700151347 12.428247430 6.903362322
H 10.329350349 12.863914380 5.965285472
H 10.467179517 13.124518961 7.723168686
H 10.137144725 11.501122506 7.070989082
C 11.874513390 9.937253890 5.707449040
H 11.166455873 9.688143731 6.497388164
C 9.124297347 8.964119305 4.813024663
C 9.308470343 9.933894041 3.822786535
H 10.001635516 9.721243674 3.009588755
C 8.630198190 11.156549438 3.862784819
C 8.891270057 12.182936790 2.754851294
C 10.396934708 12.524909864 2.739030674
H 11.015250299 11.631054870 2.584949775
H 10.708684731 12.973214730 3.692780882
H 10.617575036 13.240320517 1.932154337
C 8.493177829 11.570020350 1.395284897
H 8.680543924 12.287842250 0.582331766
H 7.426008850 11.305292730 1.384084934
H 9.068715757 10.658798269 1.182309817
C 8.096837665 13.480269551 2.961423658
H 8.322796115 14.185640994 2.148876421
H 8.359434188 13.969646472 3.910793877
H 7.011970453 13.299589180 2.954556490
C 7.762287651 11.389397492 4.934039495
H 7.224294930 12.334671086 4.982391238
C 7.579684895 10.455579905 5.969376040
C 6.615413214 10.777387045 7.118990115

C	6.711383755	9.754088253	8.261405579
H	6.424149555	8.744225203	7.937582999
H	6.031103082	10.045513514	9.074506125
H	7.729495028	9.706328610	8.674778824
C	5.175383238	10.763507741	6.560992658
H	5.053046129	11.505674779	5.759678171
H	4.451220338	10.994953953	7.356990322
H	4.928352111	9.775741228	6.145886694
C	6.923609998	12.171487803	7.703442041
H	6.825068196	12.965820674	6.951745897
H	7.946524749	12.210774448	8.102887443
H	6.225077628	12.398783882	8.522514658
C	8.272091588	9.243756515	5.893537641
H	8.185156640	8.501776055	6.683738108

Molecular structure of Benzophenone

C	0.287120900	-1.951712414	-0.058194075
C	0.945053445	-1.411084923	-1.169107173
C	1.460193281	-0.118765401	-1.112853418
C	1.349451352	0.639077526	0.064367316
C	0.686992521	0.090860875	1.174708650
C	0.149638782	-1.195616175	1.108514029
H	-0.123684923	-2.961315968	-0.105241312
H	1.049635419	-2.000177025	-2.081253656
H	1.955785435	0.331143593	-1.973627615
H	0.569146606	0.684525094	2.081493656
H	-0.379149175	-1.608192259	1.968766024
C	1.877479700	2.040001525	0.059724257
C	2.364706259	2.642637081	1.340686221
C	2.284801394	4.036358204	1.494309249
C	2.961492330	1.872930088	2.352438989
C	2.765117368	4.645756970	2.650402324
H	1.840813458	4.621539029	0.688454646
C	3.464595524	2.488040441	3.499903903
H	3.055836785	0.794106524	2.226682262
C	3.357587974	3.872296203	3.655397361
H	2.684423803	5.727006098	2.770265675
H	3.942849835	1.885887603	4.273566246
H	3.741314130	4.350927711	4.557583529
O	1.908985305	2.689340250	-0.984640849

Molecular structure of C (anion)

Si	-8.000708985	-3.363677697	-14.974128788
C	-5.542169406	-2.191421058	-12.240923944
H	-4.811174188	-2.335438085	-11.437503558
H	-6.552388773	-2.388098246	-11.862524831
B	-5.159369571	-2.831748365	-13.744393350
Si	-2.107983658	-3.364365619	-13.337222785
C	-3.857436353	-3.834900625	-13.747794066
C	-4.348609277	-5.090740263	-13.972263218
C	-5.822338472	-5.086047086	-14.243594120
C	-6.340716175	-3.824271124	-14.286553092
C	-1.474900804	-2.220586973	-14.707262681
H	-1.328803284	-2.786599985	-15.640282425
H	-2.220367601	-1.438715934	-14.903312852
H	-0.520224554	-1.741162157	-14.440141599
C	-1.993092520	-2.459608504	-11.678314203
H	-0.939721824	-2.224760448	-11.457148806
H	-2.555486094	-1.519473688	-11.667124457
H	-2.375523935	-3.095263600	-10.865034828
C	-0.837672751	-4.771271995	-13.162888992
H	-1.193294608	-5.569200103	-12.495361436
H	-0.584817501	-5.238250539	-14.123616093
H	0.088580812	-4.353020233	-12.735468396
C	-7.684775986	-2.109228161	-16.356946585
H	-6.885434261	-1.423107275	-16.050767102
H	-7.349609163	-2.634937402	-17.264609804
H	-8.585091047	-1.526872945	-16.606839800
C	-8.960927918	-4.785214710	-15.792874055
H	-9.373994814	-5.498269126	-15.067713198
H	-9.797418345	-4.361244712	-16.372792523
H	-8.321798833	-5.355010374	-16.482941970
C	-9.163693352	-2.613969485	-13.682482846
H	-9.332223344	-3.324198405	-12.858088889
H	-8.759227031	-1.688502878	-13.253704430
H	-10.141077797	-2.379633468	-14.133453958
C	-3.634211461	-6.386664256	-13.946593736
C	-2.649028549	-6.693658987	-14.894631918
H	-2.393898247	-5.924246996	-15.621686349
C	-2.012618586	-7.940013976	-14.910636867
C	-0.865326252	-8.261726679	-15.879105742
C	-0.632403214	-7.135043030	-16.897480186
H	-0.353075564	-6.192845939	-16.405394194
H	0.186826689	-7.414407410	-17.577124728
H	-1.529447575	-6.948900512	-17.505601441
C	0.427970434	-8.448089339	-15.055462802
H	0.325183861	-9.275723533	-14.339529506
H	1.280947549	-8.668209394	-15.717517702
H	0.655202961	-7.537017961	-14.483737150
C	-1.165186245	-9.558064581	-16.658717270
H	-1.317437704	-10.412479292	-15.985182614
H	-2.073670151	-9.445880846	-17.266464808
H	-0.326804252	-9.802222247	-17.330180757
C	-2.424494428	-8.903558471	-13.979688790
H	-1.954648588	-9.889048297	-13.995737118
C	-3.432053816	-8.646538873	-13.037602379
C	-3.907599582	-9.763198169	-12.098349915
C	-2.711605974	-10.349960480	-11.322357406
H	-3.047649193	-11.154612048	-10.649273873
H	-1.955159338	-10.770639216	-11.999458928
H	-2.224501625	-9.572231723	-10.716360708
C	-4.571275866	-10.869601691	-12.946214524
H	-4.920968172	-11.693154264	-12.303011586
H	-5.436188903	-10.461301774	-13.489121757
H	-3.869757428	-11.285134359	-13.683656477
C	-4.947986057	-9.259341082	-11.087133530
H	-5.262367582	-10.088301422	-10.434884758
H	-4.542642799	-8.459898213	-10.450250570
H	-5.840828775	-8.871257420	-11.596508497
C	-4.010996790	-7.376888598	-13.026231759
H	-4.798012676	-7.133593874	-12.315574909
C	-6.547010010	-6.367238074	-14.387945129
C	-7.555461899	-6.713617874	-13.480037872
H	-7.809263812	-5.982817811	-12.709829765
C	-8.213924807	-7.947442359	-13.545357245
C	-9.342037282	-8.252848213	-12.549061503
C	-10.482438574	-7.235491755	-12.768560925
H	-11.308428671	-7.422212951	-12.063761163
H	-10.132075450	-6.205124525	-12.620356008
H	-10.876088593	-7.308963151	-13.792677254
C	-8.818005589	-8.122300240	-11.104009350
H	-8.024458473	-8.857621161	-10.909324186
H	-8.403762390	-7.123427504	-10.913974954
H	-9.633202517	-8.295912852	-10.383770551
C	-9.912636754	-9.668339770	-12.724571911
H	-10.705821219	-9.842033822	-11.981814075
H	-10.352054908	-9.808434731	-13.722962580
H	-9.139030317	-10.436616288	-12.578323562
C	-7.830896612	-8.849755139	-14.548271397
H	-8.324072300	-9.816926418	-14.607504909
C	-6.830203131	-8.534893334	-15.481958878
C	-6.441412400	-9.485213355	-16.622705798
C	-6.946599608	-8.880540837	-17.950848907
H	-8.039859814	-8.761711426	-17.932987827
H	-6.505354503	-7.889016193	-18.122052620
H	-6.679004904	-9.530039546	-18.799922934
C	-7.055037286	-10.884116247	-16.455742388
H	-6.746127149	-11.348031562	-15.507514958
H	-8.154116910	-10.855761364	-16.483327431
H	-6.718347761	-11.535394731	-17.276362728
C	-4.908515535	-9.636227653	-16.685830812
H	-4.510717341	-10.040209271	-15.745964765
H	-4.626615902	-10.314569331	-17.506606027
H	-4.410405315	-8.673190595	-16.854419189
C	-6.201805898	-7.291490241	-15.383029671
H	-5.413352386	-7.015103064	-16.083239449
C	-5.397437515	-0.849564652	-12.996779366
O	-5.040877319	-1.400688283	-14.272673032
C	-6.671205600	-0.007981519	-13.081873849
C	-7.002316192	0.626801165	-14.283679367
C	-7.509264108	0.172607787	-11.974095637
C	-8.157293658	1.403536161	-14.385905488
C	-8.661105404	0.956309014	-12.068451241
C	-8.994331716	1.571056597	-13.278737607
H	-6.348588967	0.475729164	-15.141845098
H	-7.271002933	-0.331811182	-11.036269934
H	-8.410841486	1.873528217	-15.338457856
H	-9.312300438	1.070484043	-11.199269580
H	-9.903893525	2.169168041	-13.360185747
C	-4.307532790	0.085492700	-12.472989373
C	-4.220607099	0.423834013	-11.116059307
C	-3.375714263	0.628389465	-13.362997765
C	-3.211799697	1.270449739	-10.655334716
C	-2.362779615	1.474174181	-12.905942666
C	-2.273684844	1.797343467	-11.549383380
H	-4.934279190	-0.005831387	-10.411026331
H	-3.448940423	0.343260692	-14.411933429
H	-3.146698067	1.508637386	-9.591510196
H	-1.631289864	1.873250973	-13.611866277
H	-1.474752952	2.447747826	-11.188210098

Molecular structure of D (anion)

Si	-7.131867405	-3.487790839	-15.613443741
C	-7.478904980	-3.557369094	-12.667281802
H	-7.300409286	-4.238297069	-11.821336585
H	-8.565518349	-3.551075539	-12.841851633
B	-5.299321929	-3.165350281	-13.486205455
Si	-2.325073388	-3.567563965	-13.358439964
C	-4.066891996	-3.992005384	-13.729932309
C	-4.626591123	-5.305417150	-13.965735558
C	-6.031391752	-5.395385084	-13.974344890
C	-6.600066491	-3.989562665	-13.849235265
C	-1.181017023	-3.488348435	-14.880139446
H	-1.015971446	-4.494331039	-15.292495511
H	-1.625483061	-2.867395856	-15.672558137
H	-0.196676150	-3.064925224	-14.621642210
C	-2.285335279	-1.836869730	-12.582941457
H	-1.254560088	-1.520866425	-12.357423169
H	-2.729667387	-1.097229968	-13.265741627
H	-2.870066110	-1.804272284	-11.652342060
C	-1.478184460	-4.769018780	-12.151162654
H	-2.065729383	-4.867862535	-11.225895817

H	-1.388278229	-5.771864405	-12.593665566
H	-0.467637494	-4.420726052	-11.881883365
C	-6.942085155	-1.645065495	-16.022015628
H	-7.711919958	-1.019712768	-15.552701473
H	-5.960241437	-1.265209264	-15.704942181
H	-7.020744148	-1.514436182	-17.113661685
C	-6.057871238	-4.416066704	-16.859379319
H	-6.276104390	-5.492880493	-16.855749872
H	-6.220628503	-4.026006572	-17.876467149
H	-4.997813565	-4.282111515	-16.598596404
C	-8.947089196	-3.926802944	-15.933072799
H	-9.104111803	-5.011373261	-15.859153166
H	-9.613291048	-3.434465960	-15.208158469
H	-9.251870277	-3.596467504	-16.938858798
C	-3.727622312	-6.476138240	-14.099166029
C	-2.869462539	-6.592726744	-15.200396713
H	-2.923156469	-5.821111014	-15.965991010
C	-1.987862210	-7.671950062	-15.323053579
C	-1.041863777	-7.827493846	-16.521322108
C	-1.283710063	-6.754358099	-17.594073864
H	-1.099355925	-5.742036461	-17.208195095
H	-0.601630044	-6.919931997	-18.441817632
H	-2.314544992	-6.791226076	-17.974490809
C	0.415296333	-7.705966625	-16.026544392
H	0.646691589	-8.476201047	-15.277236921
H	1.120332914	-7.819388759	-16.865678284
H	0.583805633	-6.723888965	-15.561869553
C	-1.251975666	-9.210947091	-17.171739209
H	-1.049816559	-10.027474996	-16.465030169
H	-2.288555371	-9.320694174	-17.520639369
H	-0.578868898	-9.334545459	-18.034948597
C	-1.974283421	-8.630741723	-14.300465034
H	-1.280575054	-9.470416846	-14.374528267
C	-2.826807450	-8.551813403	-13.189024854
C	-2.757797852	-9.632936267	-12.102207747
C	-1.379136576	-9.557203991	-11.412001111
H	-1.298261516	-10.320966087	-10.621826533
H	-0.564671391	-9.720735368	-12.131956168
H	-1.230147632	-8.567391227	-10.957187050
C	-2.937244043	-11.026629645	-12.738698938
H	-2.886236144	-11.809965756	-11.966039154
H	-3.911835596	-11.101163281	-13.241360445
H	-2.159488477	-11.237052620	-13.485590052
C	-3.850669982	-9.450283931	-11.037923752
H	-3.785202592	-10.261692041	-10.297247589
H	-3.740250110	-8.496629544	-10.502942215
H	-4.854648920	-9.475463194	-11.485146991
C	-3.700011662	-7.464182712	-13.105742906
H	-4.388474460	-7.353046110	-12.270129503
C	-6.839550930	-6.564545598	-14.248354734
C	-8.193769774	-6.616923591	-13.842182269
H	-8.603825751	-5.751005776	-13.323325654
C	-9.003518422	-7.738507049	-14.048831469
C	-10.448875516	-7.719312338	-13.524510888
C	-11.214196730	-6.536417800	-14.151937367
H	-12.249010462	-6.501235105	-13.774089845
H	-10.732048257	-5.578102540	-13.919232910
H	-11.247436016	-6.631846999	-15.246932400
C	-10.426530605	-7.551312562	-11.989974720
H	-9.883947602	-8.384133013	-11.519011815
H	-9.923438724	-6.619170821	-11.699888878
H	-11.451928033	-7.530223219	-11.585840170
C	-11.211172993	-9.011849081	-13.854787607
H	-12.238557467	-8.946733173	-13.464682553
H	-11.271049478	-9.178221406	-14.940411367
H	-10.733063949	-9.891331581	-13.399110265
C	-8.458304210	-8.854559278	-14.696454325
H	-9.069513655	-9.740768783	-14.860926388
C	-7.124183741	-8.839248954	-15.154638857
C	-6.562372427	-10.069097197	-15.883408746
C	-7.396189498	-10.344129115	-17.152255913
H	-8.453846794	-10.515555020	-16.907612215
H	-7.345716803	-9.485246054	-17.837280508
H	-7.016932584	-11.233540802	-17.681434798
C	-6.644234089	-11.291366953	-14.944461918
H	-6.066859108	-11.107721105	-14.026849414
H	-7.681349848	-11.502919638	-14.649050206
H	-6.236195646	-12.188709507	-15.437204232
C	-5.094324818	-9.882501282	-16.298264725
H	-4.444405128	-9.687294689	-15.434068454
H	-4.731375757	-10.794110268	-16.797840036
H	-4.976593625	-9.043259744	-16.998747577
C	-6.349924087	-7.706258264	-14.941472970
H	-5.336793560	-7.673129558	-15.326509455
C	-6.970718009	-2.121161805	-12.250079601
O	-5.600081893	-2.031509574	-12.706735824
C	-7.761603406	-0.968331507	-12.859457377
C	-7.093254843	0.236387847	-13.121966089
C	-9.130730465	-1.044430327	-13.142562213
C	-7.768247086	1.323762639	-13.673488496
C	-9.810370628	0.042405217	-13.701034636
C	-9.131539871	1.231073252	-13.972061983
H	-6.025082134	0.289167817	-12.915432096
H	-9.677709139	-1.966324922	-12.946209453
H	-7.224460057	2.246418082	-13.885172069
H	-10.872797871	-0.048686473	-13.934382969
H	-9.658030947	2.076878443	-14.417755439
C	-6.961994325	-2.018875111	-10.723539756
C	-8.076509347	-1.574476607	-10.001295938
C	-5.831693020	-2.457001016	-10.017621979
C	-8.066934946	-1.572775670	-8.603728095
C	-5.820207077	-2.450792939	-8.622443722
C	-6.938208209	-2.010118004	-7.907175574
H	-8.956009674	-1.213386213	-10.535878477
H	-4.964019898	-2.798522820	-10.581624030
H	-8.944707606	-1.220014583	-8.058340554
H	-4.930403399	-2.793031798	-8.090285689
H	-6.927581011	-2.004260590	-6.815645036

Molecular structure of D* (anion)

Si	-6.889458311	-3.428860286	-16.249264842
B	-5.126047437	-2.846125689	-14.058354076
Si	-2.260177573	-3.405133989	-13.404480995
C	-3.946099398	-3.761811599	-14.037928325
C	-4.520391827	-5.089964374	-14.205391010
C	-5.916158674	-5.132683995	-14.294265366
C	-6.403947225	-3.705536876	-14.423470916
C	-0.878763462	-3.505831701	-14.712692294
H	-0.736283478	-4.545192167	-15.042946559
H	-1.131483896	-2.902311625	-15.597726315
H	0.082826237	-3.144828403	-14.312933571
C	-2.215998978	-1.637496056	-12.718406543
H	-1.199789557	-1.361766029	-12.395472679
H	-2.543890900	-0.909732034	-13.475556473
H	-2.887221181	-1.543815396	-11.851997115
C	-1.734008378	-4.565079705	-11.993891557
H	-2.500775230	-4.569600558	-11.204402074
H	-1.615256287	-5.598398681	-12.350161388
H	-0.779730484	-4.244165759	-11.545576169
C	-7.028442596	-1.577193648	-16.621752561
H	-7.699108252	-1.087960385	-15.899251938
H	-6.048183805	-1.081673851	-16.565239152
H	-7.439471914	-1.410422674	-17.629971678
C	-5.589736737	-4.212802448	-17.372777995
H	-5.658596162	-5.309215530	-17.327556838
H	-5.716013250	-3.899020922	-18.420608323
H	-4.583738708	-3.923440894	-17.035452198
C	-8.583648778	-4.181342274	-16.621799912
H	-8.571491152	-5.273140376	-16.505153777
H	-9.337762038	-3.777024183	-15.931092004
H	-8.897634898	-3.939525070	-17.649900371
C	-3.681094972	-6.308420922	-14.163583777
C	-2.703814108	-6.544404640	-15.138858166
H	-2.606783679	-5.817337177	-15.943062564
C	-1.893830018	-7.684109733	-15.091520792
C	-0.824725470	-7.977398576	-16.152547741
C	-0.824343754	-6.934080893	-17.280371730
H	-0.601336243	-5.926161870	-16.903168301
H	-0.054287824	-7.193587898	-18.022605058
H	-1.794408740	-6.897611841	-17.796354161
C	0.565538665	-7.970671211	-15.481975314
H	0.628637302	-8.725194461	-14.685290291
H	1.353035802	-8.186108416	-16.221784764
H	0.771313238	-6.988827429	-15.032060984
C	-1.089422780	-9.363086273	-16.778410719
H	-1.057627751	-10.161096919	-16.023930946
H	-2.081731009	-9.390332105	-17.250947980
H	-0.331980663	-9.589207726	-17.545658371
C	-2.076249864	-8.580232759	-14.028297012
H	-1.439772882	-9.464945827	-13.971360984
C	-3.051783019	-8.380728239	-13.039906118
C	-3.194570564	-9.382034987	-11.886111051
C	-1.979311224	-9.222721457	-10.947345863
H	-2.048093463	-9.922774798	-10.099087007
H	-1.039505442	-9.420390359	-11.482852540
H	-1.930163746	-8.198217422	-10.551609667
C	-3.234478122	-10.824958309	-12.428881644
H	-3.357226982	-11.538910435	-11.599610550
H	-4.074710286	-10.957846216	-13.124642549
H	-2.311680915	-11.089095306	-12.963197000
C	-4.480758763	-9.142512690	-11.078623784
H	-4.579298564	-9.914649083	-10.300786574
H	-4.471001782	-8.164748885	-10.577418731
H	-5.372165119	-9.184256914	-11.721012631
C	-3.845734239	-7.234899481	-13.125114912
H	-4.622861805	-7.031331067	-12.390945028
C	-6.766948819	-6.301843033	-14.454030069
C	-8.093912772	-6.257789015	-13.982610593
H	-8.428709109	-5.326581958	-13.524366507
C	-8.952938541	-7.357741201	-14.072634244
C	-10.378194101	-7.228161075	-13.515114200
C	-11.097535328	-6.058459513	-14.218612039
H	-12.118736095	-5.938924797	-13.822070941
H	-10.559038930	-5.113275850	-14.070854650
H	-11.162463379	-6.236317438	-15.301807403
C	-10.300387496	-6.938317597	-12.000754310
H	-9.793184630	-7.761148725	-11.475682623
H	-9.736566874	-6.017510411	-11.800958796
H	-11.310693599	-6.824236180	-11.575301950
C	-11.209847199	-8.502695777	-13.723466821
H	-12.221812484	-8.356395390	-13.315864564
H	-11.307818584	-8.750762392	-14.790761052

H	-10.762773787	-9.366746351	-13.209958185
C	-8.469491010	-8.542162380	-14.643464325
H	-9.119865877	-9.413614097	-14.712149567
C	-7.153153883	-8.626674945	-15.140772686
C	-6.659620563	-9.940779555	-15.763349343
C	-7.514019162	-10.276928451	-17.003352690
H	-8.577585358	-10.370031559	-16.742068004
H	-7.422778740	-9.482922479	-17.758758601
H	-7.186481301	-11.226879949	-17.456218594
C	-6.798849702	-11.074738854	-14.725714669
H	-6.215910750	-10.842221775	-13.823002063
H	-7.844709922	-11.215207375	-14.419477431
H	-6.431484810	-12.027412791	-15.139995513
C	-5.185041893	-9.863609202	-16.191681295
H	-4.525392140	-9.616396785	-15.347913935
H	-4.866527471	-10.834731346	-16.600910659
H	-5.029513883	-9.102956052	-16.970148176
C	-6.332435247	-7.506770571	-15.057761194
H	-5.328114027	-7.540265458	-15.468403158
C	-7.005016876	-2.082901841	-12.750337233
C	-8.154778566	-1.091602090	-12.675621465
C	-7.933261644	0.286962567	-12.567372115
C	-9.478027996	-1.556859771	-12.694979381
C	-9.004419770	1.181908693	-12.482578017
C	-10.549075720	-0.666375156	-12.616982027
C	-10.317783573	0.708955070	-12.508459848
H	-6.909597999	0.662670208	-12.555894614
H	-9.652191510	-2.627785654	-12.788982393
H	-8.809248029	2.253367213	-12.403389975
H	-11.571881267	-1.047851642	-12.643039237
H	-11.155857713	1.406023727	-12.449117147
C	-6.643244801	-2.620116953	-11.358099643
C	-6.068785300	-3.895588239	-11.243365570
C	-6.790963563	-1.843292511	-10.201748071
C	-5.649109501	-4.372747880	-10.000589615
C	-6.373938806	-2.323742668	-8.956643133
C	-5.796515673	-3.591267685	-8.851141276
H	-5.959956629	-4.526359752	-12.128045699
H	-7.242494110	-0.852515508	-10.272203750
H	-5.196865960	-5.364461783	-9.934852376
H	-6.504664942	-1.704172547	-8.066753689
H	-5.467161912	-3.968346904	-7.880845797
C	-5.701995899	-1.518686401	-13.405387217
O	-7.488153637	-3.193838738	-13.575342387
H	-5.067989077	-1.033961768	-12.652538084
H	-5.975708804	-0.762692678	-14.159198922

Molecular structure of 4 (anion)

O	4.440456038	5.424497947	15.037690417
C	1.812976366	5.641177302	15.409872930
B	3.237292185	5.562186403	14.579513866
Si	1.732410068	5.618938079	17.257558888
C	0.831913090	5.781536201	14.480183554
Si	3.860173773	5.584360336	11.558172068
C	1.377811888	5.845290373	13.063328827
C	2.728623752	5.706577364	13.016829747
C	3.272056717	6.495178510	17.904716046
H	4.130082353	6.125541073	17.320155539
H	3.434882918	6.316508068	18.979310625
H	3.200769925	7.581809989	17.742683443
C	1.775241142	3.822844676	17.871903421
H	0.891102558	3.261494503	17.533014795
H	1.811918202	3.771152224	18.971881081
H	2.667426678	3.319647454	17.471207136
C	0.209798722	6.464295218	18.020445463
H	0.079402600	7.473329910	17.600995094
H	0.340507462	6.563725949	19.110412263
H	-0.720861154	5.911730293	17.833030081
C	5.289694619	4.450592114	12.034568845
H	4.961384777	3.400038446	12.060474038
H	5.603207111	4.724193088	13.055170762
H	6.138576192	4.529491902	11.337262293
C	4.580280094	7.294653576	11.150297514
H	5.319045619	7.238737514	10.334837323
H	5.077983039	7.707782689	12.039994081
H	3.792620167	8.001543079	10.847742201
C	-1.342148314	6.984317637	14.201541698
H	-0.801529512	7.733828987	13.623014574
C	-0.617607756	5.906219663	14.722881045
C	3.052559543	4.891167769	9.984060773
H	2.281671011	5.557354159	9.574619905
H	2.570914074	3.925400935	10.200051240
H	3.815021392	4.723356069	9.205976993
C	-1.312950955	4.924557359	15.451628046
H	-0.736010313	4.084207310	15.834617219
C	-2.686939356	5.017272020	15.673962369
C	-4.100317529	4.664622881	17.713437864
H	-4.797554094	5.455927450	17.404825853
H	-3.327757125	5.125660706	18.345495738
H	-4.657793827	3.934527272	18.321740946
C	-2.544780772	2.847722468	17.003493236
H	-2.070398906	2.302803064	16.174769967
H	-3.136879277	2.126561007	17.587009244
H	-1.748742351	3.236208164	17.654427900
C	-3.457738537	3.972375276	16.492101701
C	-2.724119394	7.105769778	14.390266044
C	-3.378258668	6.116214593	15.130802652
H	-4.454305455	6.188518989	15.285939388
C	-4.563287692	3.342334408	15.619326835
H	-4.125642302	2.829619289	14.750924236
H	-5.260416973	4.102671307	15.240861155
H	-5.141691337	2.607037648	16.201167399
C	-3.455742696	8.305011177	13.772532948
C	-2.904258383	9.605049128	14.395489593
H	-1.825200755	9.703675062	14.216614784
H	-3.066201834	9.613416338	15.483333196
H	-3.405924713	10.484446780	13.960897788
C	-3.205090791	8.325314668	12.249934744
H	-3.608105164	7.421184242	11.772989890
H	-2.134259925	8.358879392	12.013142600
H	-3.689034555	9.203263210	11.792798501
C	-4.971732164	8.254126290	14.013107026
H	-5.451149306	9.124667618	13.540567342
H	-5.214947884	8.277677802	15.085710379
H	-5.418315649	7.347310973	13.578936939
C	0.453745715	5.996418405	11.923270464
C	-0.619849055	5.109646142	11.753691697
H	-0.733500895	4.301602481	12.476898283
C	-1.536636148	5.252900622	10.710537984
C	-2.697807059	4.255382135	10.598912198
C	-2.123817024	2.847283300	10.333429436
H	-2.935781693	2.106103599	10.260019517
H	-1.448146758	2.536933850	11.141822262
H	-1.551601581	2.831904327	9.394255712
C	-3.488677237	4.239687139	11.924123157
H	-3.926170609	5.225354170	12.134548943
H	-2.848573613	3.988590875	12.779315121
H	-4.305077601	3.501727496	11.875311042
C	-3.667788636	4.609157420	9.461720426
H	-3.170231743	4.591845887	8.480799514
H	-4.109868040	5.606260817	9.604937649
H	-4.489133625	3.877270568	9.434596801
C	-1.360784361	6.316245795	9.811682888
H	-2.072898609	6.448287140	9.000974362
C	-0.301115559	7.225485973	9.946909649
C	-0.094835744	8.400117753	8.980114538
C	1.275296746	8.246573488	8.286552945
H	1.315949380	7.310383032	7.711124213
H	2.094783491	8.221671966	9.017187298
H	1.455929208	9.086789252	7.597167507
C	-0.120769090	9.725363772	9.771085710
H	0.042396266	10.580079424	9.095471752
H	0.660743559	9.747424381	10.542113736
H	-1.090951620	9.859498511	10.271186561
C	-1.182739522	8.466826229	7.897643023
H	-0.992866462	9.324670247	7.235002240
H	-2.183057307	8.594891957	8.336899641
H	-1.193239102	7.558900334	7.276991323
C	0.591956793	7.050632132	11.010328804
H	1.420320873	7.744494214	11.162336512

Molecular structure of 1,1-Diphenylethylene

C	0.263431473	-2.025164498	-1.113518005
C	1.069897811	-1.356001469	-2.038042279
C	1.602315936	-0.104470069	-1.728182677
C	1.336494550	0.505661176	-0.490256355
C	0.535533796	-0.185495332	0.436513154
C	-0.000948635	-1.434449095	0.125391654
H	-0.149545563	-3.005840538	-1.353604084
H	1.295736609	-1.816806204	-3.000952768
H	2.254114591	0.403767348	-2.440302050
H	0.325969427	0.272286198	1.404118480
H	-0.629500347	-1.949410062	0.853541747
C	1.867457964	1.854462052	-0.171028327
C	2.343270756	2.104827849	1.212453029
C	2.108386121	3.335727957	1.848921727
C	3.060181295	1.117490730	1.911979871
C	2.590202649	3.579657911	3.135066522
H	1.520081492	4.095563508	1.332387937
C	3.546255225	1.362551246	3.195792784
H	3.244557133	0.154694182	1.433681181
C	3.313863421	2.594950015	3.812985129
H	2.389181214	4.538508142	3.615417731
H	4.110239740	0.587710264	3.717336955
H	3.687081808	2.783588809	4.820476914
C	1.916064127	2.822555532	-1.108959951
H	2.352524328	3.797973193	-0.894170286
H	1.520436406	2.660287796	-2.111567084

Molecular structure of K[18-crown-6] (cation)

K	7.621437190	5.646105694	15.508798887
O	6.971144135	3.532371951	17.250066210
O	7.446229652	3.023505908	14.504112403
O	8.624758270	5.072383108	12.942443119
O	8.274095345	7.759563383	13.768469380
O	7.798742294	8.268800343	16.514415801
O	6.620248159	6.219488569	18.075358025
C	6.531791170	2.333066385	16.604727316
H	5.512721372	2.471764392	16.197886578
H	6.503612358	1.497649166	17.327992975
C	7.495561299	1.992488961	15.495311777
H	8.522455082	1.898493922	15.895192426

H	7.207822438	1.021612223	15.052115968
C	8.302573973	2.744361440	13.392060569
H	8.023202445	1.781640295	12.926290930
H	9.353194423	2.672183596	13.730036014
C	8.156150286	3.846323995	12.372315148
H	8.749476535	3.589943099	11.475619035
H	7.097746229	3.948659408	12.068058097
C	8.560727791	6.158799321	12.013208351
H	7.514156166	6.328473816	11.698306109
H	9.158655443	5.927769872	11.112690161
C	9.116485150	7.398159830	12.669529385
H	9.149052634	8.216485642	11.927165667
H	10.147351919	7.213814846	13.025321826
C	8.713826664	8.958680515	14.413930125
H	8.742586530	9.794063928	13.690653301
H	9.732710583	8.819513581	14.821065935
C	7.749888099	9.299683619	15.523066598
H	8.037707923	10.270568531	15.966195139
H	6.723120124	9.393852804	15.122889752
C	6.941285922	8.547658109	17.625661000
H	5.890904862	8.619154470	17.286770368
H	7.219646655	9.510640875	18.091498270
C	7.087471638	7.445974615	18.645743358
H	8.145638702	7.344388715	18.951041140
H	6.493064219	7.702087335	19.541796711
C	6.683833356	5.133379047	19.004993926
H	6.085356011	5.364663608	19.905079551
H	7.730238146	4.963908083	19.320534556
C	6.128490937	3.893766990	18.348789352
H	6.095965701	3.075577873	19.091308104
H	5.097637168	4.077797135	17.992779655

Molecular structure of 2a

K	18.358439793	2.953347929	3.695142999
Si	18.787088413	6.225728211	6.614789517
O	20.248132351	4.242492476	1.753329401
C	17.918381575	2.691963246	6.909020034
H	18.915474653	2.941008627	7.289007511
H	17.683183434	1.628061108	7.012553511
B	16.975721187	3.703826544	6.419422302
O	21.211713697	2.579410106	3.815884991
C	17.249488493	5.263192336	6.217650797
Si	14.529085377	1.908962154	5.763282054
O	19.371338519	0.467087131	4.441984636
C	16.064338939	5.793950783	5.750823366
C	14.993056761	4.760565105	5.626298150
O	17.680430195	0.439646566	2.191437953
C	15.462860631	3.500400876	5.949473522
O	16.151585435	2.759803832	1.987574991
C	18.488070621	8.030574127	7.100946991
H	18.110580456	8.643891808	6.274116941
H	19.425323897	8.478884277	7.466508213
H	17.747954297	8.085478431	7.913134988
O	17.643442487	5.193021553	2.252208223
C	19.740194328	5.460640759	8.060087226
H	20.189139317	4.494413685	7.792602603
H	19.078262756	5.289709480	8.920765816
H	20.551322380	6.134319995	8.377903344
C	19.967311666	6.177815518	5.121260895
H	19.458220216	6.513397358	4.206102221
H	20.310167660	5.143802805	4.960654198
H	20.857156807	6.807311048	5.277832134
C	13.543596202	1.436040844	7.311240598
H	13.081417396	0.442466968	7.201108176
H	12.744766124	2.154568868	7.538195145
H	14.217643840	1.404784170	8.180220786
C	13.382822482	1.925160251	4.251521145
H	13.966327279	2.201843693	3.360265793
H	12.568437624	2.654066051	4.353848681
H	12.938354637	0.931895102	4.081357668
C	15.725801609	0.458513921	5.498003163
H	15.182661219	-0.417892897	5.109292386
H	16.205505707	0.162195178	6.440863460
H	16.533023968	0.713596314	4.799663058
C	15.787186384	7.203992697	5.412650878
C	16.508537407	7.856738104	4.405432670
H	17.299376275	7.298417488	3.901951596
C	16.227741040	9.179316516	4.042858889
C	17.066025706	9.844963640	2.943259309
C	18.529753718	9.942425311	3.423542776
H	19.162568710	10.401373039	2.647734400
H	18.938633179	8.950044823	3.659250422
H	18.600180104	10.554868997	4.333648989
C	17.012768677	8.981006353	1.665787890
H	15.978717201	8.880890462	1.304272343
H	17.406782777	7.974111438	1.858755881
H	17.616027238	9.437133510	0.865457926
C	16.566406098	11.254246653	2.594568733
H	17.187322452	11.680924025	1.793117710
H	16.626492126	11.931128995	3.458674899
H	15.524627490	11.237896402	2.242576548
C	15.190004852	9.844897987	4.710852930
H	14.948915415	10.866925645	4.429717554
C	14.439481923	9.219332927	5.718926362
C	13.292169658	9.919824900	6.458727410
C	12.993084487	11.314069354	5.888303946
H	12.714128831	11.265239784	4.825275222
H	13.855232009	11.989678880	5.987019403
H	12.152302385	11.765034776	6.435261685
C	13.680264316	10.068734856	7.945590997
H	13.867548985	9.089594762	8.406958290
H	12.872442785	10.563348203	8.506880015
H	14.594813255	10.670232580	8.050461589
C	12.010247977	9.065633309	6.351235265
H	11.693952466	8.958126189	5.304656388
H	11.190000779	9.539239865	6.912205324
H	12.155875802	8.052487713	6.747729659
C	13.621235741	5.140482901	5.217977882
C	14.756964620	7.901976851	6.056083861
H	14.192574105	7.381902141	6.830326229
C	12.517666283	4.787831026	6.008569491
H	12.709958138	4.222150487	6.919835115
C	11.213269339	5.172516282	5.673461970
C	10.043287808	4.755468857	6.574829586
C	10.337012789	5.166535827	8.032799830
H	11.246994821	4.687341308	8.417078992
H	9.501322519	4.874121606	8.686601063
H	10.470672025	6.255363182	8.109884811
C	9.878835584	3.222785187	6.494880414
H	9.657312737	2.910009824	5.464078160
H	9.054330308	2.887910642	7.143206298
H	10.793957682	2.704963722	6.811057306
C	8.718219711	5.409659904	6.153425354
H	8.783785474	6.507300233	6.176712499
H	7.920319747	5.104307778	6.845908557
H	8.413836609	5.103469052	5.142127995
C	11.022116671	5.927288042	4.507303549
H	10.016825677	6.241030002	4.239322940
C	12.097686888	6.309960329	3.693688434
C	11.912166154	7.134558979	2.411272280
C	12.305725259	6.262429861	1.200019514
H	12.161395237	6.816497634	0.259426571
H	11.698371116	5.346455507	1.162071566
H	13.362049625	5.966012769	1.259933569
C	10.461546877	7.602889597	2.222421930
H	10.384477086	8.210757497	1.309064757
H	10.125461027	8.221318011	3.067286986
H	9.767808899	6.755979297	2.119199084
C	12.822643003	8.378883287	2.447878737
H	13.878807769	8.114776103	2.588158931
H	12.549455585	9.043676656	3.277843611
H	12.731565071	8.945701038	1.508315679
C	13.382475175	5.914453817	4.070908189
H	14.232572870	6.242369308	3.478287547
C	21.568779666	4.383022480	2.272056924
H	21.571104296	5.119362817	3.095898071
H	22.260628141	4.739484431	1.483718077
C	22.056529439	3.041593058	2.764524743
H	22.056982613	2.305456514	1.937298106
H	23.097273410	3.156600429	3.123637831
C	21.672130929	1.352780795	4.380053924
H	22.544879405	1.530294940	5.037600680
H	22.000915714	0.671639624	3.571940607
C	20.553429586	0.728075511	5.196398474
H	20.930519038	-0.201698389	5.664128013
H	20.226783289	1.414717000	5.991366974
C	19.501874459	-0.506045831	3.411811008
H	19.906176736	-1.453057480	3.818780593
H	20.178858133	-0.156097380	2.610354094
C	18.125595979	-0.753581972	2.838267047
H	18.168635454	-1.590293853	2.114116552
H	17.430264442	-1.026000015	3.651799861
C	16.295707499	0.376004870	1.847378037
H	15.687603876	0.248306466	2.759431974
H	16.101682873	-0.482417303	1.175085362
C	15.899826955	1.647102920	1.135521031
H	16.469721028	1.762151664	0.193021422
H	14.824014720	1.584481155	0.884642142
C	15.631343865	3.980215929	1.463034194
H	14.530901406	4.007799039	1.568828001
H	15.868552091	4.056272084	0.384623816
C	16.214461156	5.139287931	2.246197563
H	15.796744940	6.084605880	1.853550169
H	15.939313316	5.048230366	3.305908092
C	18.265550866	5.319692599	0.978513681
H	17.883163037	6.208265389	0.441744263
H	18.080964671	4.427847798	0.352036970
C	19.748750838	5.470431754	1.220364958
H	20.264278537	5.715427615	0.271871297
H	19.923439633	6.293103102	1.937340675

Molecular structure of [K(18-crown-6)]4

K	6.839179675	5.660361206	15.059483024
O	4.472877615	4.814650225	15.265382701
C	1.866150824	5.201627503	15.593345449
B	3.280007808	5.047155924	14.779232095
Si	1.766403323	5.201558064	17.439520124
O	7.037084702	3.832394956	17.334214983
C	0.922506162	5.490952368	14.660697974
Si	3.863416118	5.077109117	11.697588850
O	7.850646246	3.081155312	14.666277049
C	1.504222327	5.579186392	13.258227794

C	2.836905781	5.306921284	13.222076551
O	8.849748610	5.066130081	12.936508312
C	2.986324377	6.527832735	18.027335308
H	3.939644747	6.401501236	17.497255061
H	3.159793412	6.479149232	19.113855032
H	2.590627120	7.527938783	17.792094748
O	7.554950781	7.585402034	13.129735102
C	2.316704893	3.507059344	18.078564521
H	1.540616884	2.753660070	17.875325316
H	2.507479332	3.511168765	19.162868214
H	3.231236780	3.188984462	17.559161303
O	6.810096466	8.596384657	15.675689836
C	0.100793333	5.623107154	18.232970663
H	-0.339382301	6.526877192	17.787904783
H	0.243126214	5.805517478	19.310318540
H	-0.633505961	4.814495072	18.121455248
O	6.421340054	6.614593890	17.598733138
C	5.135680473	3.719688228	12.047172870
H	4.627635445	2.748930618	12.149695021
H	5.663625003	3.896020633	12.993875959
H	5.863424654	3.628878741	11.225293214
C	4.799026015	6.665638863	11.243578279
H	5.410527940	6.524821406	10.337521473
H	5.463655914	6.991456395	12.055878067
H	4.090657448	7.482224518	11.039988457
C	-1.121107789	6.909874237	14.437981041
H	-0.496409751	7.661141733	13.954120065
C	-0.519013996	5.725518967	14.873245938
C	2.895130520	4.520745923	10.170571908
H	2.247389528	5.312105894	9.772354978
H	2.252362775	3.662058020	10.414055798
H	3.590327217	4.209949977	9.374433430
C	-1.321606083	4.737288683	15.467700829
H	-0.837677324	3.811221756	15.774137860
C	-2.691301442	4.929497555	15.652607989
C	-4.167237550	4.499680204	17.626351074
H	-4.771409564	5.390860550	17.407268583
H	-3.361312005	4.800205570	18.311197207
H	-4.809532603	3.770301811	18.143683941
C	-2.805596874	2.615213807	16.713520731
H	-2.370328845	2.128208095	15.828907661
H	-3.484060043	1.895656005	17.194605976
H	-1.991900298	2.831277837	17.420735513
C	-3.583377736	3.884924773	16.335803765
C	-2.494716169	7.134898242	14.593369541
C	-3.257960425	6.137131623	15.206794309
H	-4.328627543	6.290858253	15.334862200
C	-4.735612334	3.482752269	15.391860245
H	-4.344005542	3.026843685	14.471607839
H	-5.344706925	4.349827757	15.102452006
H	-5.396706827	2.753356381	15.884555143
C	-3.098528785	8.444859587	14.071561701
C	-2.447654035	9.631608212	14.813395084
H	-1.359914263	9.649334588	14.660975911
H	-2.635621943	9.564839270	15.894878884
H	-2.858838597	10.585285659	14.447883354
C	-2.806866746	8.567044022	12.560808276
H	-3.276039313	7.745057573	12.002690898
H	-1.730823952	8.524998041	12.348936134
H	-3.199983847	9.518596972	12.170339067
C	-4.618231701	8.513532577	14.280676451
H	-5.003899960	9.463405110	13.882470349
H	-4.885877182	8.464212535	15.346332740
H	-5.136234482	7.696695017	13.757099917
C	0.625378906	5.909893626	12.119819156
C	-0.515634521	5.141842349	11.853943731
H	-0.724847949	4.288759497	12.499495054
C	-1.380077854	5.455393694	10.802818432
C	-2.619096778	4.581574598	10.570667893
C	-2.161603526	3.152213981	10.209043128
H	-3.032886355	2.499128827	10.046445110
H	-1.552183834	2.714621559	11.011476013
H	-1.555863554	3.157753333	9.291240356
C	-3.457972065	4.535691092	11.865085203
H	-3.811148275	5.538493709	12.142466165
H	-2.877762594	4.155269787	12.715423239
H	-4.335936233	3.885761035	11.728989178
C	-3.506769753	5.112728439	9.435630276
H	-2.974593089	5.128056016	8.473241815
H	-3.867334743	6.130309879	9.646261009
H	-4.386526813	4.463017467	9.320140658
C	-1.080042174	6.572330178	10.008636194
H	-1.750870844	6.835843686	9.195156523
C	0.047158992	7.370833576	10.247875726
C	0.385306317	8.596921270	9.389138596
C	1.742695068	8.360245633	8.693478400
H	1.696607706	7.475932852	8.041663133
H	2.544566039	8.192706241	9.425454007
H	2.018011706	9.230463373	8.077610149
C	0.483209726	9.845722440	10.290843253
H	0.740769575	10.732191332	9.690959906
H	1.252808961	9.725285936	11.065319050
H	-0.474821769	10.037207691	10.795030619
C	-0.674107995	8.866329408	8.310111105
H	-0.392075687	9.756881452	7.729596257
H	-1.663553167	9.052761586	8.752315506
H	-0.763290191	8.024764272	7.608116468
C	0.887726131	7.023530502	11.312269003
H	1.767097563	7.625781179	11.545597577
C	6.609043981	2.645876549	16.658547422
H	5.701610689	2.864170471	16.063774243
H	6.373790356	1.851112155	17.393198540
C	7.733803818	2.178655457	15.767388249
H	8.680368035	2.153173344	16.340021288
H	7.520804997	1.158173105	15.396336778
C	9.015553067	2.866994413	13.881895270
H	9.112761829	1.798521979	13.607894616
H	9.920681880	3.159644367	14.449008980
C	8.891506351	3.674884613	12.612104960
H	9.755511543	3.462331507	11.952670538
H	7.965950246	3.385013919	12.083949283
C	8.536102449	5.857983277	11.792104211
H	7.536388157	5.587277331	11.406999755
H	9.275991132	5.682922906	10.986520485
C	8.568713662	7.320738619	12.164667456
H	8.391644179	7.919118929	11.250367595
H	9.560016031	7.594887907	12.573142746
C	7.406663582	8.977167342	13.399951390
H	7.105896233	9.512915159	12.479287404
H	8.365837269	9.400083395	13.753823697
C	6.342348588	9.161476838	14.455173284
H	6.140525055	10.243657240	14.577128283
H	5.400352078	8.673028487	14.138850565
C	5.869753326	8.752618880	16.740414880
H	4.884301646	8.353678623	16.438524705
H	5.752928560	9.823690657	16.996277309
C	6.367660056	7.990052554	17.947532992
H	7.363821311	8.356379388	18.261270031
H	5.655903399	8.149584738	18.778636154
C	6.411748392	5.703162965	18.697349887
H	5.672602970	6.030311100	19.450857709
H	7.409255135	5.661319746	19.174223114
C	5.997671784	4.349637906	18.172662391
H	5.814932386	3.662617881	19.021786910
H	5.073580116	4.454299051	17.577143596

Structures for the pK_a approximations

Molecular structure Acid A

C	-0.481632308	-0.727050035	-4.988442741
C	-0.481632433	-1.940212826	-4.165265188
C	-0.481632315	-1.592866892	-2.855752644
C	-0.481632371	-0.093613060	-2.749369297
C	-0.481632385	0.361467094	-4.181843118
H	-0.481632271	-0.719005370	-6.077639581
H	-0.481632477	-2.955311909	-4.560218698
H	-0.481632287	-2.265800554	-2.001000140
H	-1.361788795	0.281916232	-2.195929931
H	0.398524036	0.281916281	-2.195929938
H	-0.481632399	1.404369637	-4.491352518

Molecular structure Base A

C	-0.889486376	0.878243259	-6.148522956
C	0.258299330	0.053552632	-6.272229730
C	-0.180116710	-1.280820242	-6.472394115
C	-1.598856231	-1.280820180	-6.472394108
C	-2.037272155	0.053552731	-6.272229716
H	-0.889486330	1.958825489	-5.986423382
H	1.297484718	0.387496563	-6.222134480
H	0.462160190	-2.155015272	-6.603533535
H	-2.241133206	-2.155015155	-6.603533529
H	-3.076457513	0.387496749	-6.222134456

Molecular structure Acid B

B	-1.138461221	-0.004102759	1.167523435
C	-1.549270120	-1.474423192	0.777543767
H	-1.012659853	-1.801887118	-0.129933484
H	-1.402389685	-2.231181642	1.560951138
H	-2.615645801	-1.487530223	0.486936275
C	-0.701543088	0.338270111	2.640615360
H	-1.623446926	0.351760795	3.254855330
H	-0.069116524	-0.440931681	3.094543572
H	-0.220194406	1.318278697	2.770854066
C	-1.180177422	1.131255172	0.076657164
H	-0.133662618	1.316450490	-0.235352716
H	-1.745424487	0.887672758	-0.834504197
H	-1.532293060	2.096371414	0.474494362

Molecular structure Base B

B	-1.053339135	-0.101920276	1.157701539
C	-1.230383507	-1.498638387	0.768790582
H	-1.482529577	-1.819175918	-0.254551651
H	-1.126081549	-2.343895858	1.467511826
C	-0.681741333	0.345204664	2.673541902
H	-1.444946200	1.021183182	3.111801425
H	-0.581930411	-0.517078700	3.356916785
H	0.271043599	0.911962912	2.723347260
C	-1.212680057	1.126689398	0.108453153
H	-0.286966158	1.733280576	0.027225807
H	-1.465363226	0.783083119	-0.910655870
H	-2.002921681	1.842606127	0.415830557

Molecular structure Acid C

B	4.540375513	-0.370324735	0.424201211

C	4.021001956	-1.771267193	-0.026801317
C	4.575588015	-2.946991597	0.324368429
H	3.148001857	-1.819581477	-0.692106681
H	4.188529946	-3.909876237	-0.020847515
H	5.453162598	-2.991941314	0.975197091
C	3.839939804	0.879020028	-0.195605313
C	4.227281725	2.153874502	-0.000821850
H	2.974184607	0.724199387	-0.854082589
H	3.716477775	3.004024061	-0.461755912
H	5.085073099	2.399147221	0.631446541
C	5.685865886	-0.224156138	1.496839391
H	5.215699232	-0.198602620	2.497302725
H	6.392474913	-1.066363239	1.515603540
H	6.266032724	0.705775102	1.410373261

Molecular structure Base C

B	4.834973009	-0.360767326	0.251600361
C	4.211372154	-1.771349308	-0.115457070
C	4.259519363	-2.922604841	0.589017437
H	3.639517632	-1.834499554	-1.061290455
H	3.810288788	-3.859827469	0.236021617
H	4.768792557	-2.966106583	1.558363104
C	4.029114821	0.894349332	-0.285638981
C	3.914447479	2.121255990	0.267121585
H	3.462396174	0.759242528	-1.227012706
H	3.345441113	2.936657042	-0.197802333
H	4.404469766	2.355672868	1.218862508
C	6.110870215	-0.228182032	0.961140920
H	6.720189893	-1.088297097	1.271861671
H	6.595148113	0.739001682	1.155164650

Molecular structure Acid D

B	1.101824306	-4.357741078	-1.412633280
C	2.671733540	-4.328985253	-1.484460480
C	3.362567825	-3.468953969	-2.382587709
C	3.452186611	-5.194358231	-0.664976684
C	4.759564187	-3.497530909	-2.452883497
C	4.848190684	-5.161644748	-0.738888868
C	5.526787816	-4.325583863	-1.630197570
H	5.264695779	-2.849152433	-3.174180839
H	5.425039745	-5.822491388	-0.085845185
C	0.321726378	-5.725588887	-1.393991833
C	0.455188481	-6.628089671	-2.481649092
C	-0.536169027	-6.079382302	-0.329257452
C	-0.267544088	-7.823209699	-2.489039846
C	-1.223613946	-7.299655194	-0.356621826
C	-1.112469074	-8.183814407	-1.431164327
H	-0.160874914	-8.500332184	-3.341506580
H	-1.859504739	-7.569642795	0.491740370
C	0.233154805	-3.040014638	-1.428925996
H	0.748439152	-2.131457202	-1.087487729
H	-0.069255296	-2.861166894	-2.477826346
H	-0.710550568	-3.154201791	-0.878595154
C	2.833628861	-6.162145115	0.317787010
H	2.112716970	-5.673623550	0.986994502
H	2.290688175	-6.967384338	-0.195935628
H	3.612574936	-6.619372835	0.942430479
C	2.643552918	-2.539081714	-3.332916333
H	1.838579708	-3.046407874	-3.883153601
H	2.186736488	-1.690268987	-2.804911021
H	3.345308860	-2.130689757	-4.071936808
C	7.031101523	-4.304822268	-1.684117430
H	7.393197545	-3.906246172	-2.641394512
H	7.443456605	-3.668725366	-0.884258930
H	7.451555018	-5.311082510	-1.546212018
C	-0.701472370	-5.200554530	0.891640565
H	-0.714000917	-5.808478674	1.807908275
H	0.103717865	-4.462029935	0.994519044
H	-1.652596319	-4.645368247	0.861375674
C	1.361185854	-6.315213596	-3.649783624
H	1.107378398	-5.351142549	-4.116981177
H	2.412650267	-6.247099942	-3.336216964
H	1.282194226	-7.089324478	-4.424616792
C	-1.879529230	-9.479940774	-1.462997948
H	-2.695456535	-9.438846924	-2.202000920
H	-1.231671965	-10.322878194	-1.745465878
H	-2.326760089	-9.704363405	-0.485449420

Molecular structure Base D

B	1.043062940	-4.260620612	-1.365493536
C	2.641048634	-4.295056004	-1.519332947
C	3.342627940	-3.543613268	-2.498962594
C	3.432048065	-5.117371095	-0.665702493
C	4.738336971	-3.642614301	-2.623206732
C	4.822819509	-5.183231591	-0.802409576
C	5.503438843	-4.456581710	-1.787213495
H	5.242688511	-3.064772463	-3.406643895
H	5.393320220	-5.822519127	-0.118591500
C	0.273696058	-5.669958088	-1.377585805
C	0.488612440	-6.602104115	-2.433730684
C	-0.628887129	-6.069888990	-0.356821911
C	-0.150876183	-7.846357083	-2.448037197
C	-1.243692793	-7.332477801	-0.389476752
C	-1.021466162	-8.241198945	-1.424693844
H	0.036823707	-8.529615480	-3.284512453
H	-1.916807275	-7.615169065	0.428481954
C	0.320315198	-3.001491901	-1.220331979
H	0.804479947	-2.016710905	-1.246698239
H	-0.765591511	-2.938058134	-1.073036559
C	2.786684055	-5.927702663	0.433442644
H	2.089009238	-5.308371660	1.016357672
H	2.191429879	-6.760218137	0.029925735
H	3.546735593	-6.340945233	1.114367144
C	2.624288978	-2.622264311	-3.456913300
H	3.131450854	-2.597552935	-4.435363702
H	1.574105857	-2.921767678	-3.574997285
H	2.600806860	-1.589432774	-3.071487929
C	7.003339049	-4.545192462	-1.928027080
H	7.351974276	-3.971085824	-2.799253048
H	7.521825210	-4.148040605	-1.038873847
H	7.340403328	-5.587156685	-2.055388702
C	-0.954748788	-5.169440844	0.811786361
H	-1.136131346	-5.759089433	1.725356532
H	-0.152236089	-4.438205851	0.978460912
H	-1.864323441	-4.577962205	0.615104110
C	1.401644344	-6.258843387	-3.586900444
H	1.171112811	-5.258648630	-3.982583168
H	2.456484318	-6.228205252	-3.276085693
H	1.299517540	-6.993494906	-4.400514551
C	-1.698767803	-9.589508647	-1.450913541
H	-2.405190350	-9.676082250	-2.293994694
H	-0.970367094	-10.409922829	-1.559037460
H	-2.265753651	-9.763890309	-0.524456238

Molecular structure Acid E

C	-6.342371200	1.765363588	-0.377847698
C	-7.058529320	1.258743124	0.888127150
C	-6.187916315	0.349710293	1.775736697
C	-5.385310814	-0.747927566	1.007869305
C	-6.264728158	-1.836852612	0.344755691
C	-7.144183028	-1.351468291	-0.821938531
C	-6.418905257	-0.420150743	-1.810281919
C	-5.540634125	0.678157099	-1.161287984
B	-4.581922251	0.061632096	-0.076326460
H	-7.074113714	2.239894095	-1.054868112
H	-5.636634746	2.564348408	-0.089015133
H	-7.387482887	2.126837322	1.482341173
H	-7.978794336	0.730935139	0.607260616
H	-5.463491862	0.978673863	2.322985208
H	-6.815688162	-0.128306433	2.547997280
H	-4.730977065	-1.242362453	1.745955973
H	-6.902023676	-2.321841952	1.105212070
H	-5.589370046	-2.626872469	-0.028276379
H	-7.522633676	-2.228686234	-1.371533193
H	-8.037420773	-0.849123327	-0.427837965
H	-5.762842898	-1.032127791	-2.454075822
H	-7.159082749	0.040382335	-2.488291412
H	-4.996929942	1.199227383	-1.967674673
C	-3.037025691	0.307977470	-0.025207600
H	-2.598424706	0.246734879	0.982216479
H	-2.578777480	-0.519458611	-0.603860766
H	-2.706758750	1.235153853	-0.517477117

Molecular structure Base E

C	-6.457099695	1.810717023	-0.352297761
C	-7.176357910	1.261148426	0.899123039
C	-6.297524164	0.382934030	1.816641730
C	-5.395706257	-0.635342053	1.081363156
C	-6.178706316	-1.774139025	0.388021403
C	-7.033411437	-1.338046908	-0.822173298
C	-6.338334595	-0.346592201	-1.780921605
C	-5.555524941	0.793630042	-1.089604172
B	-4.519893985	0.148353273	-0.028923547
H	-7.223989209	2.245501548	-1.030303655
H	-5.807470754	2.647524673	-0.040023242
H	-7.575576641	2.109172698	1.486746611
H	-8.061349650	0.686414954	0.585847921
H	-5.628511066	1.045188131	2.394245129
H	-6.960046937	-0.116356915	2.557334048
H	-4.742644073	-1.103345720	1.842363017
H	-6.838958174	-2.312055195	1.103260179
H	-5.432742033	-2.510121269	0.039216792
H	-7.337247326	-2.236713942	-1.391305622
H	-7.973924072	-0.894578368	-0.461082020
H	-5.611761435	-0.908254237	-2.394450871
H	-7.102981832	0.049322979	-2.484773551
H	-5.016837433	1.347846155	-1.881622903
C	-3.070562504	0.253493365	-0.066418098
H	-2.407376499	-0.205892162	0.682710687
H	-2.520051381	0.800976351	-0.846962072

Molecular structure Acid F

B	4.522358515	-0.346080862	0.142520926
C	3.991213676	-1.743465023	-0.307599555
C	3.597571263	-2.657226007	0.613667866
H	3.767307268	-2.439507085	1.678953437
C	3.646390825	0.893576269	-0.228464352
C	4.240539500	1.848728542	-0.990704351
H	5.328626181	1.789447462	-1.148961309
C	5.875423922	-0.202017714	0.929164900
H	5.620450512	-0.359347763	1.995563077
H	6.620560611	-0.969765753	0.678761349
H	6.327330199	0.798586438	0.866458882
Si	1.913478416	0.949940541	0.529623255
Si	4.154037530	-2.012404467	-2.178894401

C	3.612416016	2.964656755	-1.706538132
C	4.297595256	4.186288969	-1.849578157
C	2.348547187	2.837015802	-2.309240432
C	3.713488582	5.259967143	-2.518906565
H	5.291941039	4.291420536	-1.410310461
C	1.768029049	3.905026931	-2.991239220
H	1.834628261	1.876710829	-2.258172571
C	2.443590795	5.124605237	-3.089784455
H	4.251641935	6.205323771	-2.602711906
H	0.789302738	3.782690464	-3.457676112
H	1.991000504	5.960819380	-3.624177620
C	2.914675243	-3.938285775	0.390150522
C	1.925071178	-4.084407353	-0.597470643
C	3.204447596	-5.046285621	1.208615744
C	1.279673894	-5.304646592	-0.792911287
H	1.648242209	-3.218081513	-1.198982247
C	2.569650361	-6.270723478	1.007704133
H	3.952263185	-4.941624597	1.997697277
C	1.606551619	-6.406368353	0.002336398
H	0.510147914	-5.393716204	-1.561337894
H	2.821851294	-7.123106767	1.640404461
H	1.101570008	-7.361346423	-0.148233283
C	4.430683488	-3.820098270	-2.639767801
H	5.135481904	-4.294305139	-1.941557725
H	3.511431723	-4.417154474	-2.638841644
H	4.871209773	-3.866043680	-3.647788683
C	5.724605632	-1.089344631	-2.690017216
H	5.912387351	-1.242056629	-3.763650645
H	5.645954364	-0.006618523	-2.520358468
H	6.603205874	-1.458168528	-2.141043062
C	2.722173776	-1.264773894	-3.151499593
H	2.627748753	-0.195222869	-2.917682102
H	2.912307793	-1.359044343	-4.231598346
H	1.759991535	-1.747401736	-2.933435568
C	0.659041551	-0.123125998	-0.381042889
H	1.013051992	-1.162062174	-0.425824789
H	-0.301716711	-0.115128091	0.156108120
H	0.474711507	0.219242478	-1.408482478
C	2.117855530	0.249582792	2.274500146
H	1.163762921	0.306398205	2.819895681
H	2.428805406	-0.803935116	2.261915278
H	2.864028964	0.822004143	2.845249239
C	1.268642801	2.710552760	0.741281949
H	2.068492495	3.379299380	1.091021083
H	0.858180013	3.139764015	-0.180279703
H	0.473704872	2.708972835	1.503072722

Molecular structure Base F

B	4.522359000	-0.346081000	0.142521000
C	3.991214000	-1.743465000	-0.307600000
C	3.597571000	-2.657226000	0.613668000
H	3.767307000	-2.439507000	1.678953000
C	3.646391000	0.893576000	-0.228464000
C	4.240539000	1.848729000	-0.990704000
H	5.328626000	1.789447000	-1.148961000
C	5.875424000	-0.202018000	0.929165000
H	5.620451000	-0.359348000	1.995563000
H	6.620561000	-0.969766000	0.678761000
H	6.327330000	0.798586000	0.866459000
Si	1.913478000	0.949941000	0.529623000
Si	4.154038000	-2.012404000	-2.178894000
C	3.612416000	2.964657000	-1.706538000
C	4.297595000	4.186289000	-1.849578000
C	2.348547000	2.837016000	-2.309240000
C	3.713489000	5.259967000	-2.518907000
H	5.291941000	4.291421000	-1.410310000
C	1.768029000	3.905027000	-2.991239000
H	1.834628000	1.876711000	-2.258173000
C	2.443591000	5.124605000	-3.089784000
H	4.251642000	6.205324000	-2.602712000
H	0.789303000	3.782690000	-3.457676000
H	1.991001000	5.960819000	-3.624178000
C	2.914675000	-3.938286000	0.390151000
C	1.925071000	-4.084407000	-0.597471000
C	3.204448000	-5.046286000	1.208616000
C	1.279674000	-5.304647000	-0.792911000
H	1.648242000	-3.218082000	-1.198982000
C	2.569650000	-6.270723000	1.007704000
H	3.952263000	-4.941625000	1.997697000
C	1.606552000	-6.406368000	0.002336000
H	0.510148000	-5.393716000	-1.561338000
H	2.821851000	-7.123107000	1.640404000
H	1.101570000	-7.361346000	-0.148233000
C	4.430683000	-3.820098000	-2.639768000
H	5.135482000	-4.294305000	-1.941558000
H	3.511432000	-4.417154000	-2.638842000
H	4.871210000	-3.866044000	-3.647789000
C	5.724606000	-1.089345000	-2.690017000
H	5.912387000	-1.242057000	-3.763651000
H	5.645954000	-0.006619000	-2.520358000
H	6.603206000	-1.458169000	-2.141043000
C	2.722174000	-1.264774000	-3.151500000
H	2.627749000	-0.195223000	-2.917682000
H	2.912308000	-1.359044000	-4.231598000
H	1.759992000	-1.747402000	-2.933436000
C	0.659042000	-0.123126000	-0.381043000
H	1.013052000	-1.162062000	-0.425825000
H	-0.301717000	-0.115128000	0.156108000
H	0.474712000	0.219242000	-1.408482000
C	2.117856000	0.249583000	2.274500000
H	1.163763000	0.306398000	2.819896000
H	2.428805000	-0.803935000	2.261915000
H	2.864029000	0.822004000	2.845249000
C	1.268643000	2.710553000	0.741282000
H	2.068492000	3.379299000	1.091021000
H	0.858180000	3.139764000	-0.180280000
H	0.473705000	2.708973000	1.503073000

Molecular structure Acid G

B	-0.957700099	-0.721316934	0.054816445
C	0.615037091	-0.753331836	0.188398355
C	-1.675986926	0.677279559	0.044475162
C	-2.750340933	0.939560656	0.904123652
C	-1.237181073	1.756026276	-0.732707027
C	-3.348432955	2.194338343	1.009568230
C	-1.821391285	3.018941795	-0.675043253
C	-2.880822675	3.239413554	0.209554038
C	-1.823483590	-2.023146222	-0.030685365
C	-2.958972808	-2.095182249	-0.858051599
C	-1.520751688	-3.194749437	0.683896644
C	-3.735219736	-3.245201705	-0.986511427
C	-2.287344111	-4.355642428	0.601681136
C	-3.398321382	-4.381076422	-0.245530250
F	-0.479693847	-3.222262705	1.543175121
F	-1.976681690	-5.441902425	1.324759720
F	-4.137401450	-5.489768932	-0.346540241
F	-4.795803431	-3.276374010	-1.806792018
F	-3.322972411	-1.034274754	-1.606272669
F	-3.224032355	-0.037155451	1.709744904
F	-4.360358059	2.409345279	1.864669505
F	-3.447318747	4.449355483	0.287785934
F	-1.382510166	4.021046792	-1.452919607
F	-0.230793877	1.585265167	-1.620306705
H	1.032022223	-0.395505094	-0.768778950
H	0.999823300	-1.769296884	0.342193007
C	1.048205680	0.173455038	1.304657892
C	1.665174435	1.404514708	1.042508676
C	0.759410738	-0.159574757	2.638474893
C	1.984401339	2.280228897	2.082417735
H	1.894618774	1.679979367	0.011858099
C	1.079434505	0.713605944	3.679407287
H	0.281778914	-1.115677809	2.860207125
C	1.690402089	1.939711505	3.405177161
H	2.468234184	3.231997332	1.857491664
H	0.847206772	0.435907663	4.708642404
H	1.939085039	2.623991675	4.217159202

Molecular structure Base G

B	-1.175106544	-0.728465347	0.610471103
C	0.210687825	-0.777814045	1.081457052
C	-2.085159119	0.587013807	0.614963705
C	-3.283444991	0.644276167	1.330756922
C	-1.760325739	1.746453326	-0.098235999
C	-4.107865158	1.769878450	1.367628995
C	-2.555846720	2.892452343	-0.093045418
C	-3.738018988	2.905835769	0.648852000
C	-1.873483265	-2.031813436	0.014900221
C	-2.716852264	-1.990652472	-1.106943674
C	-1.719437850	-3.312173489	0.573921424
C	-3.356242063	-3.107581921	-1.644101800
C	-2.342421362	-4.454202431	0.068837374
C	-3.167652150	-4.355415491	-1.051254058
F	-0.968101245	-3.496623350	1.685136587
F	-2.171626544	-5.661547855	0.660192358
F	-3.770068523	-5.455060832	-1.559476236
F	-4.141459152	-3.004075533	-2.743194702
F	-2.927694934	-0.817281603	-1.758575507
F	-3.687612355	-0.430239293	2.057242164
F	-5.253656019	1.780272923	2.090237854
F	-4.520978173	4.008629978	0.664830049
F	-2.207455113	3.991155939	-0.803011074
F	-0.638672862	1.792915230	-0.849562183
H	0.748847507	-1.723454846	0.936089478
C	1.054916021	0.245650267	1.662218084
C	2.466259541	0.082811251	1.681876045
C	0.563676097	1.435990686	2.261870792
C	3.319822880	1.042804579	2.219605810
H	2.884202748	-0.828420441	1.245563906
C	1.416029608	2.397671556	2.795423043
H	-0.513635537	1.586307187	2.324073251
C	2.806182637	2.219769220	2.777543974
H	4.399795976	0.873010127	2.203877700
H	0.989858557	3.298128901	3.245479913
H	3.471377872	2.974942559	3.200201097

Molecular structure Acid H

B	-1.010904113	-0.543366942	0.022649332
C	0.544405299	-0.508015059	0.174691502
C	-1.811522777	0.810988686	-0.028621329
C	-2.914244446	1.046778426	0.804313648
C	-1.447109761	1.868163431	-0.873613200
C	-3.610625417	2.253525966	0.819293979
C	-2.132689488	3.081977260	-0.907598648
C	-3.217778659	3.276001135	-0.048952994

C	-1.803927020	-1.896810395	-0.007691580
C	-3.007876953	-2.048200365	-0.720099350
C	-1.348201675	-3.049589152	0.656659864
C	-3.712241361	-3.248932858	-0.779327262
C	-2.036786148	-4.261290300	0.640909518
C	-3.224838864	-4.360921387	-0.087508532
F	-0.217010455	-3.010507365	1.389031349
F	-1.575772994	-5.327790544	1.311679060
F	-3.891961226	-5.518031745	-0.125276272
F	-4.846044081	-3.351717017	-1.488725884
F	-3.521601997	-1.017146640	-1.421037790
F	-3.326043512	0.088010160	1.663678561
F	-4.647625328	2.445220593	1.649251085
F	-3.880227408	4.438269426	-0.058179343
F	-1.767683295	4.059359254	-1.751782903
F	-0.424942401	1.716795483	-1.744130279
H	1.062793481	-1.374525175	-0.256421890
H	0.998878603	0.419021718	-0.193930818
H	0.752294425	-0.546220955	1.261957277

Molecular structure Base H

B	-0.916203950	-0.539190639	0.000377939
C	0.534186176	-0.539155428	0.005427689
C	-1.753907016	0.822706151	-0.011047395
C	-2.887171905	1.010968302	0.793424072
C	-1.420045292	1.932320996	-0.802863800
C	-3.634842031	2.187645213	0.828868562
C	-2.140784113	3.127668752	-0.800283675
C	-3.258272885	3.261372939	0.023268677
C	-1.753894242	-1.901134405	0.005962318
C	-2.881510043	-2.089454014	-0.806394937
C	-1.425508096	-3.010728076	0.800092927
C	-3.628862559	-3.266164235	-0.847043183
C	-2.146155900	-4.206109279	0.792492623
C	-3.257870523	-4.339869119	-0.038828957
F	-0.380985198	-2.960462631	1.662884091
F	-1.791157656	-5.242214940	1.594165732
F	-3.961465486	-5.497532578	-0.066299438
F	-4.700480818	-3.390176122	-1.669360655
F	-3.294598210	-1.094284130	-1.636683552
F	-3.306001748	0.015776067	1.620804757
F	-4.712162882	2.311607658	1.643705952
F	-3.962092219	4.419006394	0.045849809
F	-1.780255742	4.163793638	-1.599458325
F	-0.369530336	1.882106266	-1.658352979
H	1.133569390	-1.452955208	0.066554429
H	1.133940514	0.374670426	-0.051525441

Molecular structure Acid J

B	8.216949395	10.374038642	18.580639383
C	8.942973458	11.275384962	19.674917832
C	9.470161900	10.424756356	20.578183983
C	9.191999922	8.981276841	20.215195698
C	8.482077466	8.883123992	19.073305407
H	9.028525001	12.359412930	19.749873069
H	10.034597314	10.693587926	21.475136288
H	9.545092797	8.153830747	20.836535071
H	8.175597780	7.932598466	18.636729368
C	7.474498593	10.842225299	17.296593866
H	8.253562599	10.912934295	16.508856642
H	6.726900143	10.131872057	16.916057753
H	7.033816110	11.847014090	17.368280801

Molecular structure Base J

B	8.127057368	10.379319158	18.621757535
C	8.895449914	11.256910186	19.703471542
C	9.495070458	10.398341464	20.584191189
C	9.225244485	8.990188408	20.230264281
C	8.443592784	8.899018358	19.110864967
H	8.981645835	12.345053835	19.801388102
H	10.105699245	10.676672105	21.453779539
H	9.621964743	8.152225230	20.819288743
H	8.136261182	7.933749553	18.692681538
C	7.333181119	10.820300130	17.472246344
H	6.835865472	10.130531510	16.774913915
H	7.170361637	11.876712506	17.213682013

Molecular structure Acid K

B	8.253120605	10.360566964	18.605810273
C	9.035459287	11.272248387	19.636954978
C	9.555520968	10.423631577	20.558041090
C	9.189340188	8.965822843	20.246026926
C	8.432872014	8.875218119	19.124229334
C	7.420974312	10.831246946	17.371351869
H	6.384225583	10.986398145	17.733104612
H	7.738405523	11.795365193	16.947726993
H	7.345040357	10.085234279	16.566302639
C	7.884253238	7.630436165	18.499564871
H	6.785234501	7.677302482	18.417120371
H	8.259246734	7.503254562	17.469762059
H	8.135176827	6.715829093	19.056568642
C	9.662127638	7.860622131	21.133861915
H	9.314559572	6.879670570	20.787294830
H	10.763307812	7.832610710	21.184730833
H	9.310077214	8.000617194	22.169294249
C	10.386769667	10.755062560	21.754871477
H	9.901589719	10.415210484	22.684926027
H	11.364874966	10.247630167	21.715351151
H	10.567739809	11.833400306	21.842074444
C	9.172904410	12.762296536	19.598137967
H	8.185665615	13.253992887	19.621518730
H	9.763531972	13.167729269	20.432707953
H	9.653160239	13.093421091	18.661726317

Molecular structure Base K

B	8.170040428	10.364258964	18.648962920
C	8.932725594	11.251989730	19.728286410
C	9.515615669	10.393668155	20.622038985
C	9.233060310	8.969709030	20.270594108
C	8.461848120	8.879059075	19.142638974
C	7.393107938	10.804084287	17.491677433
H	6.895033445	10.116703443	16.792478584
H	7.240947868	11.859516533	17.222677746
C	9.010338782	12.749684217	19.774780746
H	8.002876627	13.199822178	19.833694994
H	9.595010309	13.142585637	20.626660482
H	9.463812809	13.154927163	18.852070490
C	10.347386443	10.740484195	21.827352944
H	9.906947205	10.352218304	22.765149731
H	11.365505018	10.311021507	21.770481616
H	10.457756212	11.828891679	21.943961429
C	9.772927940	7.845375025	21.112802433
H	9.420552305	7.900995673	22.160145662
H	9.472478289	6.863258836	20.718358651
H	10.878143247	7.854871759	21.164271450
C	7.989659151	7.605549032	18.505085764
H	8.347909408	7.523904695	17.462845337
H	8.313737767	6.691236722	19.035088820
H	6.886764620	7.575055021	18.443852930

Molecular structure Acid L

B	-0.043712393	1.322838231	-0.053298444
C	1.236985123	0.394948424	-0.145812467
C	0.786413192	-0.894624778	-0.077321530
C	-0.738832318	-0.941642709	-0.008094659
C	-1.269030254	0.318942085	0.020523673
C	2.636976358	0.832852704	-0.110772741
C	3.088554245	1.832213601	-0.994810273
H	2.390677093	2.250675029	-1.721719272
C	4.415196425	2.264636093	-0.976844639
H	4.744167421	3.030643316	-1.680957662
C	5.318944033	1.721180045	-0.060481630
H	6.354642945	2.062826895	-0.040777623
C	4.882690075	0.740029460	0.836517535
H	5.578326235	0.316718851	1.562965912
C	3.561115760	0.300677760	0.811133326
H	3.227102362	-0.462149407	1.514882104
C	1.609755610	-2.109351378	-0.043859028
C	2.672426684	-2.272435716	-0.953325347
H	2.863572405	-1.490106091	-1.687811243
C	3.465092679	-3.416711177	-0.923147383
H	4.277934632	-3.530326523	-1.641776398
C	3.222205957	-4.416604267	0.025152329
H	3.846024770	-5.311079769	0.050396465
C	2.173542382	-4.266039678	0.935967241
H	1.977118611	-5.041202534	1.677896605
C	1.365930623	-3.129975898	0.895241119
H	0.545564844	-3.021748048	1.604359945
C	-1.480901217	-2.208324945	0.001929733
C	-1.175135888	-3.239552350	-0.906802386
H	-0.368360232	-3.099175754	-1.625873823
C	-1.905138470	-4.427760928	-0.905251825
H	-1.661930511	-5.211217054	-1.624260912
C	-2.935562243	-4.619046593	0.018612236
H	-3.498225392	-5.553504184	0.026517147
C	-3.238572331	-3.608182567	0.937544205
H	-4.037278066	-3.753061679	1.666338842
C	-2.523724423	-2.413406528	0.925569305
H	-2.761606660	-1.622456427	1.636883541
C	-2.691851933	0.669867055	-0.036615641
C	-3.585162604	0.021905446	-0.914603741
H	-3.208737477	-0.766889801	-1.566125593
C	-4.929741228	0.381590475	-0.964878622
H	-5.599791566	-0.131325706	-1.656823465
C	-5.421522191	1.397873094	-0.138481269
H	-6.475170408	1.677284623	-0.177647689
C	-4.549057262	2.058015718	0.730461025
H	-4.920302888	2.854393262	1.377584178
C	-3.199919125	1.704756004	0.773136173
H	-2.526277390	2.219638586	1.459452189
C	-0.086300606	2.875154447	0.039882088
H	-0.087716191	3.129451420	1.119010313
H	-1.008007539	3.314256695	-0.368786858
H	0.791982782	3.381818947	-0.382856867

Molecular structure Base L

B	-0.043207051	1.368633528	-0.057979645
C	1.223433089	0.420641966	-0.085840003
C	0.766859938	-0.888802379	-0.054768696
C	-0.712912577	-0.933763079	-0.006657923
C	-1.249166913	0.345489877	-0.006656971
C	2.624052942	0.833882253	-0.049676441
C	3.077339484	1.887237507	-0.875628168
H	2.357365349	2.354585915	-1.548572358
C	4.405032437	2.314622393	-0.846923810
H	4.723610461	3.127764487	-1.503949033

C	5.328120970	1.709493844	0.012586337
H	6.367578877	2.043193468	0.034734116
C	4.896268309	0.673795953	0.849877937
H	5.600689248	0.197347451	1.536103651
C	3.570486133	0.246089732	0.820893097
H	3.243613251	-0.553922658	1.485781124
C	1.608602375	-2.096819335	-0.044485806
C	2.666611050	-2.248908797	-0.963469755
H	2.841984995	-1.454853731	-1.689857953
C	3.479110231	-3.381419013	-0.950555527
H	4.292210497	-3.470153480	-1.674478572
C	3.255330039	-4.402671316	-0.020223656
H	3.889251066	-5.291514781	-0.011474808
C	2.206742723	-4.271604252	0.895810818
H	2.018232825	-5.060415250	1.627449928
C	1.395030682	-3.137346824	0.882572056
H	0.580684406	-3.043335501	1.601465447
C	-1.478857224	-2.191086862	0.013017037
C	-1.201456057	-3.238645726	-0.888880825
H	-0.394172522	-3.112198694	-1.610759766
C	-1.941947702	-4.420609353	-0.873783429
H	-1.705059354	-5.213827340	-1.586345374
C	-2.980862730	-4.593622809	0.046301008
H	-3.558977215	-5.519677892	0.059743751
C	-3.267318585	-3.565822931	0.951901490
H	-4.073720447	-3.686779412	1.678644385
C	-2.525886506	-2.385595360	0.936571729
H	-2.749962743	-1.586460799	1.643740602
C	-2.672555386	0.670682377	-0.051797630
C	-3.579992961	0.005494422	-0.908076433
H	-3.203561270	-0.788049327	-1.554235378
C	-4.929626077	0.349687095	-0.946453853
H	-5.602584576	-0.185202995	-1.621092684
C	-5.425461198	1.375907522	-0.133281699
H	-6.483526981	1.644139794	-0.162716054
C	-4.542373923	2.056718996	0.711515613
H	-4.911243434	2.863665429	1.349596106
C	-3.190836898	1.712966263	0.749586475
H	-2.501794810	2.239378364	1.411205398
C	-0.088241777	2.829549450	-0.075146359
H	0.806410166	3.462381132	-0.037120959
H	-1.020200578	3.404933924	-0.127346105

Molecular structure Acid M

B	-2.968434029	-0.562340219	9.569367016
C	-1.688969927	-1.504809011	9.504497776
C	-2.140010699	-2.787542237	9.553406630
C	-3.657645680	-2.834895572	9.578448182
C	-4.187608607	-1.581983326	9.618229971
C	-0.283305207	-1.098489451	9.549536421
C	0.235968443	-0.215067808	8.589137081
C	1.565078419	0.203461858	8.595809493
C	2.425972643	-0.260872504	9.593891426
C	1.945439465	-1.135264968	10.571820362
C	0.611459785	-1.540558277	10.538764646
C	-1.321666222	-4.005681801	9.587190820
C	-0.336679843	-4.259139079	8.619132763
C	0.435807702	-5.419356900	8.639287107
C	0.238567768	-6.363412287	9.651007766
C	-0.727536062	-6.138141198	10.635027649
C	-1.487341008	-4.970957757	10.592192321
C	-4.396937615	-4.102691317	9.557216349
C	-4.168847757	-5.066645590	8.563188970
C	-4.852201307	-6.280552926	8.533460394
C	-5.802448294	-6.556367118	9.520053105
C	-6.060505162	-5.615681155	10.521170045
C	-5.364207961	-4.408079700	10.528184449
C	-5.614871752	-1.262519747	9.566504833
C	-6.481996865	-1.777467466	8.587544427
C	-7.837509703	-1.452145622	8.546771989
C	-8.369145777	-0.585650067	9.504941717
C	-7.537128541	-0.048772532	10.491057724
C	-6.185669136	-0.388048416	10.505794133
C	-3.010332391	0.981289171	9.652373167
H	-2.171524608	1.482500083	9.152063393
H	-3.959825258	1.420557453	9.315788966
H	-2.928291857	1.236681746	10.727997748
F	-0.557449556	0.215527884	7.588450627
F	2.027337428	1.030895395	7.646654559
F	3.705466665	0.132648399	9.614475671
F	2.765857671	-1.567245874	11.541967521
F	0.181399681	-2.352236643	11.525287474
F	-0.147819268	-3.397422819	7.605715074
F	1.352477772	-5.644283950	7.687676581
F	0.976365553	-7.478290822	9.679450043
F	-0.900277611	-7.030425232	11.620368528
F	-2.363185316	-4.750427268	11.591039987
F	-3.308474531	-4.801526189	7.561825817
F	-4.622013045	-7.170863342	7.558148387
F	-6.467108853	-7.716555535	9.504199136
F	-6.961311647	-5.888417068	11.475429739
F	-5.608722656	-3.549119793	11.532051799
F	-6.005254763	-2.582742593	7.617489496
F	-8.630454584	-1.953123994	7.586986850
F	-9.669599456	-0.268656615	9.476861808
F	-8.047368193	0.771649405	11.421634059
F	-5.419370228	0.113061443	11.494441513

Molecular structure Base M

B	-2.966968545	-0.521639773	9.526543845
C	-1.709741485	-1.472383331	9.487559790
C	-2.161906403	-2.776148503	9.534797493
C	-3.632052135	-2.820861441	9.573740152
C	-4.163470710	-1.546542380	9.589690878
C	-0.294652104	-1.100965322	9.517203458
C	0.276951738	-0.292807947	8.517724522
C	1.617947574	0.090647773	8.537469322
C	2.446498906	-0.329045744	9.578417649
C	1.918793427	-1.130918955	10.589824001
C	0.574836289	-1.501594606	10.547360202
C	-1.320692867	-3.982307608	9.570319088
C	-0.344233096	-4.240265131	8.594093328
C	0.465308355	-5.375501392	8.626636976
C	0.309579519	-6.308607444	9.651936522
C	-0.652686451	-6.090118258	10.638021943
C	-1.441099234	-4.941425441	10.589146742
C	-4.397594206	-4.076904029	9.568649119
C	-4.218354355	-5.051440430	8.573358690
C	-4.934880669	-6.247130952	8.553442257
C	-5.882212783	-6.500064784	9.545735670
C	-6.095086834	-5.553534145	10.548193094
C	-5.356654207	-4.370371036	10.552079403
C	-5.598690879	-1.263601117	9.552400966
C	-6.441474802	-1.742155487	8.533369485
C	-7.805715307	-1.456181628	8.483419063
C	-8.382203945	-0.663815867	9.475499833
C	-7.581447414	-0.168451378	10.504882947
C	-6.219373656	-0.467935895	10.532444200
C	-3.011490824	0.934775791	9.508987905
H	-2.116905363	1.565910722	9.466055122
H	-3.942805342	1.511222209	9.537488051
F	-0.459590706	0.098765901	7.462044162
F	2.131227593	0.848601063	7.544452706
F	3.744852161	0.038734382	9.607343783
F	2.711104420	-1.526581604	11.610009713
F	0.111707078	-2.246153038	11.575206179
F	-0.187490816	-3.402514763	7.551359644
F	1.380475230	-5.594743140	7.660319284
F	1.083689672	-7.411202470	9.690563891
F	-0.790681328	-6.977725261	11.644345505
F	-2.313391835	-4.747073805	11.599178618
F	-3.359495924	-4.828627037	7.557708223
F	-4.742462777	-7.148910479	7.568887398
F	-6.587314411	-7.648623514	9.534915060
F	-6.995339095	-5.804793112	11.520699244
F	-5.564721880	-3.518694087	11.574344909
F	-5.933014700	-2.481801972	7.523544905
F	-8.571783193	-1.924963553	7.474221156
F	-9.700764084	-0.377631163	9.439120818
F	-8.140787533	0.580510705	11.479671012
F	-5.508756692	-0.006019765	11.577405651

Molecular structure Acid N

B	8.185110443	10.375912967	18.651399812
C	8.882952237	11.251266510	19.783405808
C	9.590886767	10.404512970	20.562357689
C	9.250131128	8.948978326	20.228256396
C	8.497940952	8.875848113	19.108096826
C	7.425650945	10.873546825	17.408570121
H	6.349873826	10.801032433	17.681813042
H	7.601608110	11.925387721	17.155374664
H	7.540651920	10.232634561	16.526304087
C	7.990316413	7.633169041	18.430338591
C	9.637121116	7.792670499	21.141184166
C	10.675847309	10.791319411	21.559977125
C	8.836928577	12.750386903	19.840666944
F	7.738762023	7.883473261	17.112702526
F	8.864309771	6.598723799	18.458025804
F	6.816925644	7.209198077	18.976181777
F	8.724483228	6.797801720	21.095616577
F	9.687106908	8.208409167	22.432956860
F	10.841486124	7.269201853	20.815157694
F	10.184675594	10.951779536	22.810142768
F	11.284460501	11.943440507	21.199688295
F	11.645342051	9.842258658	21.599772257
F	8.994077890	13.262429449	21.082884494
F	9.770972646	13.317594181	19.027893327
F	7.617345421	13.183175879	19.400054857

Molecular structure Base N

B	8.297480601	10.364545720	18.543652009
C	9.010775670	11.240305685	19.636097661
C	9.570897876	10.388369089	20.568563966
C	9.216956435	8.984269639	20.267551801
C	8.531327799	8.902820412	19.070758631
C	7.592247242	10.800702790	17.338302681
H	7.441450721	11.852605420	17.083537238
H	7.168202316	10.104263125	16.610768932
C	8.065919508	7.651745145	18.412716713
C	9.505358901	7.839734162	21.201410273
C	10.487023361	10.787824098	21.693907850
C	9.096674013	12.726019301	19.645762830
F	6.887212199	7.160497580	18.928754658

F	7.822097864	7.829992156	17.076230984
F	8.967391521	6.614518333	18.466522905
F	8.604797997	6.823898755	21.087494467
F	9.452118989	8.215293782	22.517214400
F	10.740397979	7.272553123	21.011927086
F	9.838119100	10.992711896	22.885484564
F	11.170956660	11.937648013	21.436849297
F	11.452358763	9.847766237	21.938044256
F	8.954727047	13.293419581	20.890467533
F	10.278730940	13.215293470	19.135547994
F	8.114498605	13.305928916	18.886511054

Molecular structure Acid O

C	-1.354245019	-1.456505682	-0.004229593
C	-2.708697771	-1.360171952	0.015981359
C	-2.053610236	0.922293454	-0.024693085
C	-2.262219585	2.293174150	-0.041200857
C	-3.578686255	2.807770211	-0.026264712
C	-4.665515180	1.941483960	0.003820785
C	-4.472004178	0.541946628	0.019968089
C	-3.176646576	0.050402205	0.005417878
H	-1.415344378	2.983715096	-0.066505792
H	-3.743799994	3.886093195	-0.039215882
H	-5.679626653	2.344286176	0.014494650
H	-5.332745841	-0.129954695	0.042987603
B	-0.764832563	0.004193609	-0.032309882
H	-0.837922071	-2.417470300	-0.000897147
H	-3.412836446	-2.197303360	0.037143567
C	0.732435784	0.439837060	-0.013869024
H	1.093738871	0.338455468	1.028220825
H	1.380431646	-0.221522918	-0.609451063
H	0.911625945	1.483036403	-0.309646579

Molecular structure Base O

C	-1.367285055	-1.450767005	-0.081346168
C	-2.731593807	-1.355758414	-0.012471094
C	-2.041852051	0.915587931	-0.076249011
C	-2.279756830	2.291630203	-0.081300787
C	-3.587127359	2.806059983	-0.020722983
C	-4.684245255	1.935904985	0.046426535
C	-4.481242274	0.548045871	0.053406159
C	-3.177559585	0.039955406	-0.007046162
H	-1.434096600	2.986187600	-0.133315556
H	-3.753186340	3.887592137	-0.025740672
H	-5.699543820	2.340152882	0.093403473
H	-5.339940266	-0.130519253	0.105897982
B	-0.738530345	-0.000527991	-0.131460951
H	-0.865108490	-2.425098557	-0.095024929
H	-3.443467816	-2.189392288	0.034400773
C	0.666468658	0.395557308	-0.208465658
H	1.497411315	-0.322349412	-0.242185361
H	1.005860420	1.440195343	-0.239087830

Molecular structure Acid P

C	-5.427722872	-3.578804136	0.637583933
C	-4.032950580	-3.642990713	0.613052994
C	-3.276649942	-2.460024157	0.594655217
C	-3.915796728	-1.220102301	0.601839004
C	-5.338626419	-1.173028493	0.625982611
C	-6.092983353	-2.341116655	0.644098697
H	-6.009987363	-4.501823771	0.651077760
H	-3.535250341	-4.613790382	0.607434490
H	-2.185489914	-2.516332498	0.573999830
H	-7.184215621	-2.310114344	0.662618505
C	-4.689295209	1.122198649	0.602100908
C	-4.914185372	2.498909472	0.595194294
C	-6.226218836	2.998870180	0.613761912
C	-7.308482025	2.116699309	0.638206427
C	-7.105890429	0.726226648	0.644448288
C	-5.804284501	0.237058625	0.626139962
H	-4.071110480	3.193911164	0.574625356
H	-6.404514783	4.075145480	0.608351663
H	-8.325906767	2.511467111	0.651847804
H	-7.964077145	0.051501505	0.662913608
B	-3.379019447	0.256073837	0.590144660
C	-1.897033468	0.745070733	0.626443088
H	-1.603523728	0.835103204	1.690099726
H	-1.183829305	0.040093671	0.174661878
H	-1.744259940	1.740566115	0.184551607

Molecular structure Base P

C	-5.460082699	-3.576370748	0.653183519
C	-4.058562276	-3.647621998	0.595622835
C	-3.292383810	-2.476528829	0.547659422
C	-3.897820638	-1.211868534	0.555703294
C	-5.326797226	-1.161435818	0.614741451
C	-6.096403125	-2.330440477	0.662797981
H	-6.052779027	-4.494189293	0.690423366
H	-3.568115105	-4.625537160	0.588471971
H	-2.200994072	-2.549844626	0.503087415
H	-7.188699564	-2.277397902	0.707667059
C	-4.670292871	1.126809696	0.555994174
C	-4.937243277	2.503275534	0.548323712
C	-6.250215837	2.987594758	0.596624364
C	-7.333553434	2.095561307	0.654170479
C	-7.102565886	0.715744648	0.663389849
C	-5.788089674	0.235147647	0.614960486
H	-4.104248383	3.212240343	0.503799369
H	-6.438738541	4.065240015	0.589770054
H	-8.356315419	2.479768982	0.691708482
H	-7.948345979	0.022512486	0.708280144
B	-3.333915442	0.271310071	0.511872887
C	-1.950054096	0.728376929	0.447611569
H	-1.085848074	0.051258449	0.420632061
H	-1.659152411	1.787019285	0.420771223

Molecular structure Acid Q

C	-0.881702406	-0.000767247	-4.796946254
C	-2.039948298	-0.349817117	-4.096928893
C	0.277214108	0.351335280	-4.103030240
H	-2.943377673	-0.622494898	-4.647116474
H	1.182333169	0.625520165	-4.646541765
C	-2.064022277	-0.355139393	-2.695889351
C	0.267411722	0.352197332	-2.704943469
H	1.167139690	0.628204748	-2.152552255
C	-0.891802302	0.002840394	-2.011470425
H	-0.890554945	0.008107327	-0.918871814
H	-0.886973627	-0.002694634	-5.888140526
C	-3.307969165	-0.744542854	-1.938756398
H	-4.166696909	-0.859291642	-2.613276630
H	-3.167522567	-1.699861386	-1.409345810
H	-3.567911412	0.009469231	-1.181133748

Molecular structure Base Q

C	-0.854394391	-0.030774938	-4.809258549
C	-2.039471014	-0.307000032	-4.145992466
C	0.327032199	0.318582950	-4.120396550
H	-2.929231942	-0.573516802	-4.727090610
H	1.256434674	0.534676960	-4.650254258
C	-2.162555092	-0.260289506	-2.701079391
C	0.246842716	0.374586009	-2.712283447
H	1.139723880	0.641778408	-2.133886641
C	-0.928253486	0.102033160	-2.030012414
H	-0.938274441	0.159334053	-0.935938555
H	-0.840488136	-0.087123880	-5.904613771
C	-3.347618787	-0.535841381	-2.025464084
H	-4.257252064	-0.805365163	-2.566369911
H	-3.400848489	-0.490137464	-0.935632785

Molecular structure Acid R

C	-1.120191063	-0.002946936	-4.226808457
H	-0.238880561	0.554002072	-4.592276449
C	-1.120191043	-1.406358841	-4.838467816
H	-0.239292388	-1.960529594	-4.471701864
H	-2.001089674	-1.960529626	-4.471701856
H	-2.001501575	0.554002053	-4.592276453
C	-1.120191048	-1.395584610	-6.368337897
H	-2.007512960	-0.875656308	-6.760744326
H	-0.232869160	-0.875656270	-6.760744331
H	-1.120191026	-2.414529382	-6.781378800
H	-1.120191044	1.380501878	-0.997616344
C	-1.120191038	1.407229652	-2.096769912
H	-2.007516315	1.974172740	-2.417513971
H	-0.232865734	1.974172702	-2.417513963
C	-1.120191066	-0.000483812	-2.695897207
H	-0.239295004	-0.557533825	-2.333509563
H	-2.001087149	-0.557533794	-2.333509566

Molecular structure Base R

C	-1.120191062	0.021632723	-4.272329379
H	-0.237924396	0.582905042	-4.633963307
C	-1.120191028	-1.394199475	-4.863842270
H	-0.239274167	-1.939971447	-4.476423386
H	-2.001107834	-1.939971507	-4.476423352
H	-2.002457740	0.582905018	-4.633963317
C	-1.120191056	-1.455164997	-6.394341736
H	-2.006057313	-0.947086941	-6.808804943
H	-0.234324866	-0.947086850	-6.808804977
H	-1.120191011	-2.493701951	-6.779753542
C	-1.120191046	1.397357170	-1.980975596
H	-2.016374007	1.989901396	-2.277694605
H	-0.224007987	1.989901300	-2.277694500
C	-1.120191078	0.068601287	-2.703694822
H	-0.237053603	-0.538311308	-2.393562601
H	-2.003328601	-0.538311256	-2.393562631

Molecular structure Acid S

C	5.344538320	-2.494315725	4.000620175
C	6.848694959	-2.461819720	3.667669559
C	7.278708766	-1.053136962	4.100865519
C	6.119542323	-0.167261133	3.610751732
C	4.843632240	-1.036196514	3.788337301
H	4.792920199	-3.222827938	3.390537422
H	5.207637713	-2.790586740	5.051849967
H	7.000470070	-2.574771811	2.581295424
H	7.414241087	-3.263883973	4.162895625
H	7.351547074	-1.009906683	5.200512229
H	8.253774315	-0.749005732	3.694339485
H	6.057479502	0.790000359	4.146669160
H	6.268312973	0.070733546	2.545959806
H	4.251025706	-0.699504965	4.650484866
H	4.189070901	-0.958421758	2.909168430

Molecular structure Base S

C	5.267071084	-2.418182353	3.765189727
C	6.827286362	-2.491926726	3.968886351
C	7.299659435	-1.007165866	3.908738742
C	5.978593948	-0.182008768	3.674208052
C	4.817289239	-1.024973839	4.181908512
H	5.072124496	-2.595269644	2.680997392
H	4.751197856	-3.230689645	4.309203535
H	7.351465160	-3.111691642	3.208684454
H	7.056743580	-2.932960674	4.954374441
H	7.764137062	-0.708296145	4.864397845
H	8.067701560	-0.861327521	3.117770043
H	6.038937897	0.816762762	4.144378165
H	5.896250488	-0.005644865	2.575589871
H	4.793760575	-0.972171373	5.295579188

Molecular structure Acid T

C	-0.147119344	3.333743567	-2.002244678
N	1.206991223	3.382816094	-2.046597820
C	1.990796800	4.612575511	-2.036217747
H	1.344490093	5.487488938	-1.943671201
H	2.694114057	4.608120819	-1.188620310
H	2.573564736	4.705509882	-2.966925353
C	1.972814118	2.141573977	-2.124201897
H	1.759686572	1.503915879	-1.254457576
H	1.690982258	1.571749794	-3.021117840
H	3.040292064	2.391003775	-2.156338405
S	-0.980425385	1.887511129	-2.024860153
C	-0.891530901	4.648957424	-1.930499868
H	-0.626276817	5.217326887	-1.024793383
H	-0.676221358	5.286500030	-2.802659492
H	-1.963629078	4.439429339	-1.907789324

Molecular structure Base T

C	-0.191610701	3.337508361	-2.216059277
N	1.238207565	3.368572758	-2.287297685
C	1.917485744	4.590324236	-1.934858876
H	1.870905557	4.833063434	-0.845677807
H	2.981109346	4.505458825	-2.213228642
H	1.476177426	5.436118860	-2.482458426
C	1.971066404	2.175186504	-1.918650404
H	2.167054630	2.116145215	-0.821543072
H	1.355967296	1.311964893	-2.213013407
H	2.945986376	2.151410990	-2.438368427
S	-0.982091761	1.851188106	-2.684008959
C	-0.866684226	4.473572980	-1.838324957
H	-0.373423330	5.378248788	-1.480140815
H	-1.953979748	4.469793260	-1.869451815

Molecular structure Acid U

C	-3.145270828	-0.985750063	0.482894036
C	-1.623834429	-0.893784266	0.388318268
C	-1.068910739	0.517384682	0.411589459
C	-1.603691111	1.330873124	1.617062171
C	-3.152786583	1.330932289	1.582096928
C	-3.719927531	-0.075431269	1.566047687
H	0.027812836	0.462909725	0.427532367
H	-3.544481991	-0.620595732	-0.481452637
H	-3.465585414	-2.022374550	0.633105336
H	-3.567117865	1.866519826	2.446671900
H	-3.487190846	1.848907543	0.665032471
H	-1.373807292	1.024038001	-0.521939885
O	-0.925444587	-1.889308332	0.328441867
O	-4.548325672	-0.474828180	2.363953163
C	-1.114792167	0.694734484	2.929506874
H	-0.016263665	0.686557181	2.969546290
H	-1.486660511	1.260544552	3.795535312
H	-1.458319610	-0.344532202	3.040165608
C	-1.095959770	2.774559455	1.517680448
H	-1.464483395	3.376189922	2.361601570
H	0.003478688	2.803234423	1.537211798
H	-1.433161228	3.251827907	0.585533349

Molecular structure Base U

C	-2.966483306	-1.136323074	0.905821580
C	-1.671297779	-0.939806862	0.364607547
C	-1.078326782	0.480959197	0.460060676
C	-1.594363527	1.323829073	1.638538997
C	-3.131629295	1.282619882	1.613468085
C	-3.745494480	-0.130000503	1.529918288
H	0.018531704	0.374705203	0.496789920
H	-3.398259772	-2.138635882	0.833822268
H	-3.557598070	1.770993829	2.505535207
H	-3.491788627	1.854161457	0.737082383
H	-1.314364969	1.004111339	-0.486001284
O	-0.974195137	-1.819533557	-0.205541881
O	-4.902353428	-0.285889979	2.001420377
C	-1.075409893	0.743064220	2.966049830
H	0.026413777	0.756482626	2.992870801
H	-1.447253524	1.331668240	3.820564802
H	-1.411839859	-0.295275263	3.088630333
C	-1.103089591	2.773019729	1.506017440
H	-1.473584361	3.395508749	2.338195397
H	-0.000828458	2.820721704	1.511164145
H	-1.456890035	3.221718882	0.564122829

Molecular structure Acid V

C	-2.972002347	-0.995476347	0.798239300
C	-1.665349554	-0.638611439	0.118381712
C	-1.601857799	1.223058468	1.695426901
C	-3.776064508	0.185490473	1.303927623
H	-3.588198294	-1.583927692	0.110229064
H	-2.745236305	-1.649357170	1.656531596
O	-1.161745037	-1.285545499	-0.765873228
O	-4.979429455	0.204963570	1.378622989
C	-1.135296372	0.560596205	2.986438365
H	-0.038688672	0.550613234	3.017675525
H	-1.514076846	1.126699501	3.846481451
H	-1.493061766	-0.473260556	3.068188261
C	-1.123742658	2.653991140	1.552083849
H	-1.504519763	3.257396283	2.385097436
H	-0.027260994	2.680713805	1.555401801
H	-1.491332544	3.071664160	0.607303539
O	-1.070916405	0.516389836	0.546324858
O	-3.049601191	1.288909509	1.657823776

Molecular structure Base V

C	-2.897475057	-1.054699485	0.972178561
C	-1.624308444	-0.841021321	0.395715771
C	-1.612451842	1.214848110	1.682330598
C	-3.686332398	-0.035983696	1.554281894
H	-3.355402687	-2.032145200	0.836340792
O	-0.932505927	-1.610180746	-0.275236805
O	-4.859818638	-0.076820947	1.930632231
C	-1.082776769	0.656246124	3.013502857
H	0.016286949	0.679684928	3.018588714
H	-1.464918515	1.257778291	3.850548393
H	-1.419110593	-0.381105960	3.135510906
C	-1.174523199	2.657744384	1.459091108
H	-1.558281144	3.297427483	2.265933794
H	-0.077688751	2.719382459	1.434203582
H	-1.576327453	3.008741555	0.499884626
O	-1.077365106	0.466679484	0.589438518
O	-3.040762635	1.233222508	1.692362640

Molecular structure Acid W

C	0.324598703	1.234811862	-0.340982908
C	1.719149185	1.238900214	-0.405324123
C	2.418311955	2.447521876	-0.431620552
C	1.718252507	3.655136326	-0.390328225
C	0.323624742	3.657423090	-0.325950193
C	-0.372609436	2.445700563	-0.304041925
H	-0.217680420	0.288819002	-0.306699535
H	2.258617787	0.291594563	-0.428074550
H	3.507825481	2.448206874	-0.476585112
H	2.256990947	4.603066566	-0.401325169
H	-0.219360181	4.602501789	-0.280129124
C	-1.912731598	2.444320013	-0.302300008
H	-2.260764617	2.443480307	-1.353891198
C	-2.479318364	3.648575857	0.322346003
N	-2.912803036	4.618678962	0.790528534
C	-2.476354823	1.239152199	0.323447275
N	-2.907108453	0.268224046	0.792449658

Molecular structure Base W

C	0.366141543	1.235988540	-0.188436857
C	1.737269224	1.244069435	-0.426891143
C	2.445320356	2.447958116	-0.546734230
C	1.735374578	3.650018625	-0.419951020
C	0.364237518	3.654565116	-0.181456345
C	-0.367258274	2.444332624	-0.057551515
H	-0.167400937	0.287635242	-0.098243870
H	2.265208120	0.291139814	-0.521335379
H	3.520603787	2.449344157	-0.733755196
H	2.261811790	4.604307624	-0.508891804
H	-0.170793863	4.601541688	-0.085786127
C	-1.795925631	2.442490213	0.190930212
C	-2.505449175	3.649830362	0.317695061
N	-3.066419398	4.679520966	0.418372935
C	-2.503554267	1.233322631	0.310672615
N	-3.063019509	0.202188817	0.404737004

Molecular structure Acid X

B	8.215043194	10.376475633	18.579106888
C	8.913980685	11.304830098	19.660364479
C	9.517555692	10.312645600	20.630963068
C	9.272426482	9.032262157	20.307041443
C	8.449301866	8.877709542	19.046336127
H	10.091505036	10.615474513	21.510027402
H	9.625435171	8.181044927	20.894146107
C	7.492184037	10.845180455	17.273380524
H	8.266968683	10.896467006	16.483463257
H	6.730891654	10.143960084	16.901401104
H	7.058524083	11.854102390	17.333845674
H	9.663492958	11.987413540	19.215905125
H	8.188175357	11.987011801	20.145138515
H	8.950528962	8.263508656	18.273834356
H	7.491837410	8.349976288	19.225035201

Molecular structure Base X

B	8.108407384	10.395149083	18.573421597
C	8.894852669	11.328310719	19.677820863
C	9.520770665	10.318271429	20.611142912
C	9.274444291	9.034792760	20.288873305
C	8.419736610	8.852766081	19.056241513
H	10.129239064	10.596561088	21.484056241
H	9.668544894	8.196145836	20.881336266

C	7.318340501	10.832975941	17.433627945
H	6.819106729	10.143770706	16.735944763
H	7.154208314	11.889780950	17.174352229
H	9.670083076	11.984368282	19.232134857
H	8.226624340	12.016910198	20.233783288
H	8.953132904	8.248798489	18.294178655
H	7.507231471	8.268603738	19.292636874

Molecular structure Acid Y

B	8.216163855	10.384578530	18.557112908
C	8.892030329	11.328284442	19.639987679
C	9.527058872	10.370489672	20.675162577
C	9.194721059	8.986175168	20.194419361
C	8.480148923	8.936983685	19.042175072
C	7.478950561	10.838115777	17.248510611
H	8.198997725	10.764237929	16.411387352
H	6.640600118	10.179882607	16.973357787
H	7.125049295	11.879069147	17.263438004
H	8.180239356	7.985772742	18.595300252
H	9.524738897	8.107899993	20.761440429
H	10.621234611	10.495602680	20.756911192
H	9.143970822	10.527050753	21.698843379
H	9.635043640	12.008502383	19.190113037
H	8.145472046	11.996524464	20.103078769

Molecular structure Base Y

B	8.081938854	10.408300991	18.582585403
C	8.797389627	11.345713260	19.723891923
C	9.643146191	10.369386505	20.595913560
C	9.198370418	8.974442770	20.194383871
C	8.377603714	8.953823759	19.116069971
C	7.359317098	10.797249721	17.372551910
H	6.906319464	10.066375393	16.686729167
H	7.215805171	11.836687904	17.042089920
H	8.019547876	7.998747249	18.706652675
H	9.573045514	8.091648624	20.732865507
H	10.728681717	10.493344000	20.396311153
H	9.529293997	10.542072693	21.685276669
H	9.413653291	12.168359535	19.322633493
H	8.026124131	11.823626611	20.356121686

Molecular structure N-heterocyclic olefin model

N	3.105828000	10.141896000	15.027494000
N	0.924445000	10.219467000	15.092810000
C	1.319554000	9.066411000	15.777856000
C	2.671529000	9.018343000	15.737391000
C	2.027928000	10.923728000	14.604866000
C	2.047889000	12.083160000	13.894443000
H	2.988908000	12.522565000	13.576931000
H	1.123143000	12.588920000	13.632794000
H	-0.027027000	10.525582000	14.954710000
H	0.606363000	8.389354000	16.228964000
H	3.360552000	8.291432000	16.146529000
H	4.066669000	10.380012000	14.832144000

Molecular structure Borole-NHC-Anion model

B	7.330510000	5.613280000	12.580345000
C	8.465523000	4.639820000	12.135641000
C	8.614452000	4.797646000	10.764018000
C	7.687520000	5.789133000	10.244874000
C	6.894416000	6.314221000	11.256659000
H	9.066098000	3.913417000	12.697410000
H	9.322118000	4.252092000	10.126176000
H	7.650855000	6.073084000	9.185019000
H	6.151502000	7.096111000	11.055009000
C	6.791561000	5.823411000	13.944017000
N	5.767321000	6.727830000	14.347925000
C	5.322640000	6.393543000	15.693421000
H	4.922600000	7.270432000	16.229221000
H	4.561484000	5.580900000	15.728318000
C	6.640879000	5.884799000	16.292781000
H	6.485589000	5.224460000	17.162197000
H	7.261046000	6.752424000	16.614634000
N	7.180704000	5.166607000	15.147079000
H	5.049328000	6.862683000	13.641672000
H	8.167793000	4.926800000	15.172405000

Literature

[1] R. K. Harris, E. D. Becker, S. M. Cabral de Menezes, R. Goodfellow, P. Granger, *Pure Appl. Chem.* **2001**, *73*, 1795-1818.

[2] T. Heitkemper, L. Naß, C. P. Sindlinger, *Dalton Trans.* **2020**, *49*, 2706 - 2714.

[3] M. F. Lappert, M. J. Slade, A. Singh, J. L. Atwood, R. D. Rogers, R. Shakir, *J. Am. Chem. Soc.* **1983**, *105*, 302-304.

[4] SAINTv8.30C, Bruker AXS,Madison, Wisconsin, USA, **2013**.

[5] a) G. M. Sheldrick, SADABS, University of Göttingen, Göttingen, Germany, **2008**; b) L. Krause, R. Herbst-Irmer, G. M. Sheldrick, D. Stalke, *J. Appl. Crystallogr.* **2015**, *48*, 3-10.

[6] G. M. Sheldrick, Bruker, Madison, Wisconsin, USA, **2012**.

[7] G. M. Sheldrick, *Acta Crystallogr.* **2015**, *A71*, 3.

[8] G. M. Sheldrick, *Acta Crystallogr.* **2015**, *C71*, 3.

[9] C. B. Hübschle, G. M. Sheldrick, B. Dittrich, *J. Appl. Crystallogr.* **2011**, *44*, 1281-1284.

[10] D. Kratzert, I. Krossing, *J. Appl. Crystallogr.* **2018**, *51*, 928-934.

[11] a) F. Neese, *Wiley Interdiscip. Rev. Comput. Mol. Sci.* **2012**, *2*, 73-78; b) F. Neese, *Wiley Interdiscip. Rev. Comput. Mol. Sci.* **2018**, *8*, e1327.

[12] S. Grimme, S. Ehrlich, L. Goerigk, *J. Comput. Chem.* **2011**, *32*, 1456-1465.

[13] a) A. D. Becke, *Phys. Rev. A* **1988**, *38*, 3098-3100; b) J. P. Perdew, W. Yue, *Phys. Rev. B* **1986**, *33*, 8800-8802; c) A. Schäfer, C. Huber, R. Ahlrichs, *J. Chem. Phys.* **1994**, *100*, 5829-5835; d) F. Weigend, R. Ahlrichs, *Phys. Chem. Chem. Phys.* **2005**, *7*, 3297-3305; e) K. Eichkorn, F. Weigend, O. Treutler, R. Ahlrichs, *Theor. Chem. Acc.* **1997**, *97*, 119-124.

[14] a) E. D. Glendening, C. R. Landis, F. Weinhold, *Wiley Interdiscip. Rev. Comput. Mol. Sci.* **2012**, *2*, 1-42; b) E. D. Glendening, J. K. Badenhoop, A. E. Reed, J. E. Carpenter, J. A. Bohmann, C. M. Morales, P. Karafiloglou, C. R. Landis, F. Weinhold, NBO7, Theoretical Chemical Institute, University of Wisconsin Madison, **2018**.

[15] a) www.chemcraftprog.com; Version 1.8 **2018**; b) G. Knizia, J. E. M. N. Klein, *Angew. Chem. Int. Ed.* **2015**, *54*, 5518-5522; c) G. Knizia, IBOview v20150427, **2015**; T. A. Keith, AIMAll, TK Gristmill Software, Overland Parks (KS), USA, **2019**

[16] B. Metz, H. Stoll, M. Dolg, *J. Chem. Phys.* **2000**, *113*, 2563-2569.

[17] T. Yanai, D. P. Tew, N. C. Handy, *Chem. Phys. Lett.* **2004**, *393*, 51-57.

[18] P. Moquist, G.-Q. Chen, C. Mück-Lichtenfeld, K. Bussmann, C. G. Daniliuc, G. Kehr, G. Erker, *Chem. Sci.* **2015**, *6*, 816-825.

[19] F. G. Bordwell, G. E. Drucker, H. E. Fried, *J. Org. Chem.* **1981**, *46*, 632-635.

[20] A. Streitwieser, M. R. Granger, F. Mares, R. A. Wolf, *J. Am. Chem. Soc.* **1973**, *95*, 4257-4261.

[21] a) F. G. Bordwell, *Acc. Chem. Res.* **1988**, *21*, 456-463; b) F. G. Bordwell, H. E. Fried, *J. Org. Chem.* **1991**, *56*, 4218-4223.

[22] W. N. Olmstead, F. G. Bordwell, *J. Org. Chem.* **1980**, *45*, 3299-3305.

[23] E. M. Arnett, S. G. Maroldo, S. L. Schilling, J. A. Harrelson, *J. Am. Chem. Soc.* **1984**, *106*, 6759-6767.

[24] Obtained from Hans Reich's compilation of Bordwell pK_a in DMSO (updated 03/16/2021); https://organicchemistrydata.org/hansreich/resources/pka/pka_data/pka-compilation-reich-bordwell.pdf

[25] a) Z. Chen, C. S. Wannere, C. Corminboeuf, R. Puchta, P. v. R. Schleyer, *Chem. Rev.* **2005**, *105*, 3842-3888; b) A. Stanger, *J. Org. Chem.* **2006**, *71*, 883-893.

[26] J. P. Perdew, K. Burke, M. Ernzerhof, *Phys. Rev. Lett.* **1996**, *77*, 3865-3868.

[27] J. O. C. Jiménez-Halla, E. Matito, J. Robles, M. Solà, *J. Organomet. Chem.* **2006**, *691*, 4359-4366.
